# Global mapping of randomised trials related articles published in high-impact-factor medical journals: a cross-sectional analysis

**DOI:** 10.1186/s13063-019-3944-9

**Published:** 2020-01-07

**Authors:** Ferrán Catalá-López, Rafael Aleixandre-Benavent, Lisa Caulley, Brian Hutton, Rafael Tabarés-Seisdedos, David Moher, Adolfo Alonso-Arroyo

**Affiliations:** 1 Department of Health Planning and Economics, National School of Public Health, Institute of Health Carlos III, Madrid, Spain; 2 Department of Medicine, University of Valencia/INCLIVA Health Research Institute and CIBERSAM, Valencia, Spain; 3 Clinical Epidemiology Program, Ottawa Hospital Research Institute (OHRI), Ottawa, Ontario Canada; 4 Ingenio-Spanish National Research Council (CSIC) and Universitat Politècnica de Valencia (UPV), Valencia, Spain; 5 Information and Social and Health Research Unit (UISYS), University of Valencia and Spanish National Research Council (CSIC), Valencia, Spain; 6 Ear, Nose and Throat Department, Guy’s Hospital, London, UK; 7 Department of Clinical Epidemiology, Erasmus University Medical Centre, Rotterdam, The Netherlands; 8 School of Epidemiology and Public Health, University of Ottawa, Ottawa, Ontario Canada; 9 Department of History of Science and Documentation, University of Valencia, Valencia, Spain

**Keywords:** Evidence-based medicine, Randomized controlled trial, Scientific collaboration

## Abstract

**Background:**

Randomised controlled trials (RCTs) provide the most reliable information to inform clinical practice and patient care. We aimed to map global clinical research publication activity through RCT-related articles in high-impact-factor medical journals over the past five decades.

**Methods:**

We conducted a cross-sectional analysis of articles published in the highest ranked medical journals with an impact factor > 10 (according to Journal Citation Reports published in 2017). We searched PubMed/MEDLINE (from inception to December 31, 2017) for all RCT-related articles (e.g. primary RCTs, secondary analyses and methodology papers) published in high-impact-factor medical journals. For each included article, raw metadata were abstracted from the Web of Science. A process of standardization was conducted to unify the different terms and grammatical variants and to remove typographical, transcription and/or indexing errors. Descriptive analyses were conducted (including the number of articles, citations, most prolific authors, countries, journals, funding sources and keywords). Network analyses of collaborations between countries and co-words are presented.

**Results:**

We included 39,305 articles (for the period 1965–2017) published in forty journals. *The Lancet* (*n* = 3593; 9.1%), the *Journal of Clinical Oncology* (*n* = 3343; 8.5%) and *The New England Journal of Medicine* (*n* = 3275 articles; 8.3%) published the largest number of RCTs. A total of 154 countries were involved in the production of articles. The global productivity ranking was led by the United States (*n* = 18,393 articles), followed by the United Kingdom (*n* = 8028 articles), Canada (*n* = 4548 articles) and Germany (*n* = 4415 articles). Seventeen authors who had published 100 or more articles were identified; the most prolific authors were affiliated with Duke University (United States), Harvard University (United States) and McMaster University (Canada). The main funding institutions were the National Institutes of Health (United States), Hoffmann-La Roche (Switzerland), Pfizer (United States), Merck Sharp & Dohme (United States) and Novartis (Switzerland). The 100 most cited RCTs were published in nine journals, led by *The New England Journal of Medicine* (*n* = 78 articles), *The Lancet* (*n* = 9 articles) and *JAMA* (*n* = 7 articles). These landmark contributions focused on novel methodological approaches (e.g. the “Bland-Altman method”) and trials on the management of chronic conditions (e.g. diabetes control, hormone replacement therapy in postmenopausal women, multiple therapies for diverse cancers, cardiovascular therapies such as lipid-lowering statins, antihypertensive medications, and antiplatelet and antithrombotic therapy).

**Conclusions:**

Our analysis identified authors, countries, funding institutions, landmark contributions and high-impact-factor medical journals publishing RCTs. Over the last 50 years, publication production in leading medical journals has increased, with Western countries leading in research but with low- and middle-income countries showing very limited representation.

## Background

Randomised controlled trials (RCTs) are considered one of the simplest and most powerful tools for assessing the safety and effectiveness of treatment interventions [1–3]. When appropriately designed, conducted and reported, RCTs can produce an immediate impact on clinical practice and patient care [4].

The evolution of RCTs has been an enduring and continuing process [5–15]. Since the 1970s the publication landscape for RCTs has exhibited an exponential growth. For example, a 1965–2001 bibliometric analysis of the literature identified 369 articles published in 1970 compared to 11,159 published in 2000 [5]. The development of clinical trial registries (such as clinicaltrials.gov) [9, 10], the exponential increase in journals publishing trial protocols, results and secondary studies, and growing support for data-sharing policies [11, 12] have created an open research environment of transparency and accountability. Furthermore, the publication of reporting guidelines (such as CONSORT and SPIRIT) [4, 13–15] have served to facilitate the transition between research and reporting to ensure standardisation and ease of readability.

RCTs published in major medical journals are highly cited and have an instrumental role in clinical practice and health policy decisions [5, 16, 17]. Previous studies have focused on the quality of the reporting of methods and results of RCTs [18–22] and publication practices [23–28] in selected samples of articles published in high-impact-factor (IF) medical journals. However, to the best of our knowledge, no mapping studies have been conducted on major medical journals to investigate the most common subjects, most productive scientists and countries, most prolific journals and “citation classics” across multiple specialties.

The objective of this study was to describe and characterise the global clinical research publication activity through RCT articles published in high-IF medical journals during the past decades.

## Methods

### Eligibility criteria

This cross-sectional analysis investigated RCT-related articles (that is, primary RCTs, secondary analyses and methodology papers using clinical data) published in major medical journals. We excluded narrative reviews, systematic reviews, meta-analyses, pool-analyses, letters and newspaper articles. All RCT-related articles indexed in PubMed/MEDLINE had to be published in one of the major medical journals with an IF exceeding 10 (2016 IF according to the Journal Citation Reports [JCR] published in June 2017). These medical journals were chosen because they were identified as publishing clinical research with scientific merit and clinical relevance (see Table 1 for a list of the included medical journals).

**Table 1:** Included high-impact-factor medical journals

General medicine journals (with an IF > 10):	
- *The New England Journal of Medicine* (IF = 72.406)	
- *The Lancet* (IF = 47.831)	
- *JAMA* – *the Journal of the American Medical Association* (IF = 44.405)	
- *The BMJ* – *the British Medical Journal* (IF = 20.785)	
- *Annals of Internal Medicine* (IF = 17.202)	
- *JAMA Internal Medicine* – formerly, Archives of Internal Medicine (IF = 16.538)	
- *PLOS Medicine* (IF = 11.862)	
Medical specialty journals (with an IF > 10):	
- *Lancet Oncology* (IF = 33.900)	
- *World Psychiatry* (IF = 26.561)	
- *Lancet Neurology* (IF = 26.284)	
- *Journal of Clinical Oncology* (IF = 24.008)	
- *European Heart Journal* (IF = 20.212)	
- *JACC* – *Journal of the American College of Cardiology* (IF = 19.896)	
- *Lancet Infectious Diseases* (IF = 19.864)	
- *Lancet Diabetes & Endocrinology* (IF = 19.742)	
- *Circulation* (IF = 19.309)	
- *Lancet Respiratory Medicine* (IF = 19.287)	
- *Gastroenterology* (IF = 18.392)	
- *Gut* (IF = 16.658)	
- *JAMA Oncology* (IF = 16.559)	
- *European Urology* (IF = 16.265)	
- *JAMA Psychiatry* – formerly, Archives of General Psychiatry (IF = 15.307)	
- *American Journal of Psychiatry* (IF = 14.176)	
- *Circulation Research* (IF = 13.965)	
- *Hepatology* (IF = 13.246)	
- *American Journal of Respiratory and Critical Care Medicine* (IF = 13.204)	
- *Blood* (IF = 13.164)	
- *Journal of Allergy and Clinical Immunology* (IF = 13.081)	
- *Annals of the Rheumatic Diseases* (IF = 12.811)	
- JNCI – *Journal of the National Cancer Institute* (IF = 12.589)	
- *Journal of Hepatology* (IF = 12.486)	
- *Intensive Care Medicine* (IF = 12.015)	
- *Diabetes Care* (IF = 11.857)	
- *Annals of Oncology* (IF = 11.855)	
- *Leukaemia* (IF = 11.702)	
- *Lancet Psychiatry* (IF = 11.588)	
- *European Respiratory Journal* (IF = 10.569)	
- *Brain* (IF = 10.292)	
- *JAMA Pediatrics* – formerly, Archives of Pediatrics & Adolescent Medicine (IF = 10.251)	
- *JAMA Neurology* – formerly, Archives of Neurology (IF = 10.029)	

### Search

On March 22, 2018, we systematically searched MEDLINE through PubMed (National Library of Medicine, Bethesda, MD, United States) for all RCT-related articles published in high-IF medical journals (from inception to December 31, 2017). A senior information specialist (AA-A) and a clinical epidemiologist (FC-L) designed an electronic literature search using a validated research methodology filter for RCTs (with 97% specificity and 93% sensitivity) [29]. The search was peer reviewed by members of the study team, including a second (senior) information specialist (RA-B). The full search strategy is provided in Additional file 1. On May 7, 2018, we searched the Web of Science (WoS) (Clarivate Analytics, Philadelphia, Penn., United States) by using PubMed IDs (PMIDs) from the PubMed/MEDLINE searches. Merging MEDLINE with other citation indices such as the WoS combines the advantages of MEDLINE (e.g., Medical Subject Headings [MeSH], a comprehensive controlled vocabulary for indexing journal articles) with the relational capabilities and data of the WoS [30].

### Data extraction and normalisation

For each included article, raw (meta) data on the journal and article titles, subject category, the year of publication, keywords, and the authors’ names, institutional affiliation(s), funding source, and country was downloaded online through the WoS by one researcher (A-AA). We also used the WoS to determine the extent to which each article had been cited in the scientific peer-review literature using the “times cited” number (that is, the number of times a publication has been cited by other publications). Two researchers (FC-L, RA-B) independently verified the data to minimise potential information errors. A process of normalisation was conducted by two researchers to bring together the different names of an author or country and the keywords (further details are available in Additional file 2). Specifically, one researcher (AA-A) checked the names by which an individual author appeared in two or more different forms (for example, “John McMurray” or “John J. McMurray” or “John J.V. McMurray”) using coincidence in that author’s place(s) of work as the basic criterion for normalisation (for example, University of Glasgow, Scotland, United Kingdom) [31], and a second researcher (FC-L or RA-B) verified the data. A threshold of 30 articles was applied to review 200 names by which an individual author appeared in two or more different forms.

We extracted both “author keywords” and “keyword plus,” which are automatically assigned by the WoS from the titles of the references of the articles, as topical (also called textural, linguistic or sematic) data [32]. To ensure consistency in the data, one researcher (RA-B) corrected keywords by unifying grammatical variants and using only one keyword developed to name the same concept (for example, “randomized trial” or “randomized clinical trial” or “randomized controlled trial” or “randomised controlled trial”). In addition, the same researcher (RA-B) removed typographical, transcription and/or indexing errors, and a second researcher (FC-L) verified the data. All potential discrepancies were resolved via consensus amongst these investigators. All these data were collected and entered into a Microsoft Access® (Microsoft, Seattle, WA, United States) database between May 7, 2018, and January 9, 2019.

### Data analysis

We analysed data for the number of articles, citations, signatures (or total number of authors included in all the articles of each author), collaboration index (that is the mean number of author’s signatures per article), countries, journals and keywords. Data were summarised as frequencies and percentages for the categorical items. The most prolific authors (>100 articles), countries (>100 articles), funding institutions (>100 articles), and the most cited papers (“top-100 citation classics”) were identified. Network plots were generated for intense scientific collaboration between countries (applying a threshold of 100 articles in collaboration).

We conducted an exploratory analyses of topical data using a set of unique keywords and their frequencies to examine the topic coverage, major topics (“word clouds” of keywords) and their interrelations (“co-words networks”) in RCT articles. The main goal in topical analyses is to understand the topical distribution of a dataset, i.e. what topics are covered and how much of each topic is covered in a scientific discipline [32]. The most frequently used keywords were identified for the most prolific journals (with at least 1000 articles). Based on the most frequently used keywords (with at least 500 articles), a word cloud was created from text that the user provides and more emphasis was placed on words that appear with greater frequency in the source text. A “co-words network” was created to illustrate the co-occurrence of highly frequent words in the articles (applying a threshold of 100 articles in collaboration). The network analysis was carried out with the use of PAJEK (University of Ljubljana, Slovenia) [33], a software package for large network analysis that is free for non-commercial use to construct network graphs. The PRISMA checklist [34] (http://www.prisma-statement.org/) guided the reporting of the present analysis (and is available in Additional file 3).

## Results

**Fig. 1:**
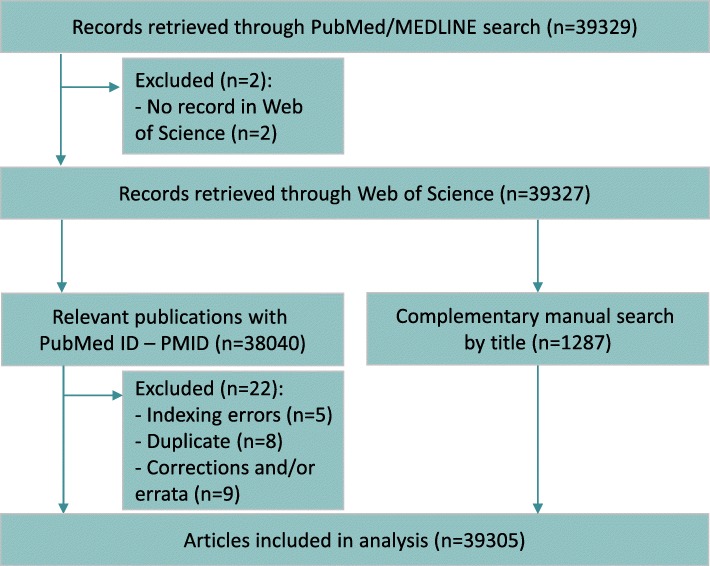
Flow diagram with selection of articles

A total of 39,329 records were identified by the PubMed/MEDLINE search (Fig. 1), and 39,305 articles met the study inclusion criteria (Additional file 4) after 24 records had been excluded (Additional file 5). Table 2 details the general characteristics of the articles.

**Table 2:** General characteristics of the study sample

Characteristic	Number	Percent
Total number of articles	39,305	100.0
Journal (top-10)		
The Lancet	3593	9.1
Journal of Clinical Oncology	3343	8.5
The New England Journal of Medicine	3275	8.3
The BMJ	2516	6.4
Circulation	2331	5.9
JACC Journal of the American College of Cardiology	2133	5.4
JAMA	1904	4.8
Diabetes Care	1885	4.8
Journal of Allergy and Clinical Immunology	1345	3.4
European Heart Journal	1315	3.3
Year of publication		
Before 1980	2004	5.1
1980–1989	4040	10.3
1990–1999	9626	24.5
2000–2009	12,574	32.0
2010–2017	11,061	28.1
Journal impact factor (2016)		
10.0–15.0	12,150	30.9
15.1–20.0	10,388	26.4
20.1–25.0	7174	18.3
25.1–30.0	238	0.6
>30.0	9355	23.8
Main subject category^a^		
Medicine, General & Internal	13,688	30.7
Cardiac & Cardiovascular Systems	5828	13.1
Oncology	5760	12.9
Gastroenterology & Hepatology	3023	6.8
Psychiatry	2380	5.3
Number of citations		
0–50	15,449	39.3
51–100	8714	22.2
101–500	13,056	33.2
501–1000	1445	3.7
>1000	641	1.6
Number of authors		
1	1064	2.7
2–3	3405	8.7
4–6	10,340	26.3
7–10	11,142	28.3
>10	13,354	34.0
Country of first author (top-10)		
United States	14,508	36.9
United Kingdom	4924	12.5
The Netherlands	1874	4.8
Germany	1862	4.7
Canada	1847	4.7
France	1732	4.4
Italy	1720	4.4
Australia	999	2.5
Sweden	755	1.9
Denmark	660	1.7
Source of funding		
Reported	16,485	41.9
None/not reported	22,820	58.1

^a^Subject category according to Journal Citation Reports (JCR)

### Publication trend

**Fig. 2:**
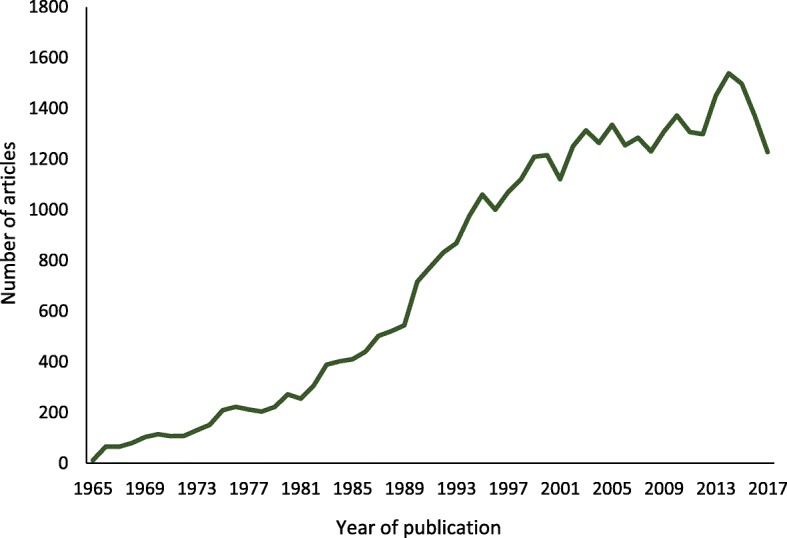
Number of articles by year of publication

The number of articles increased exponentially over the period 1965–2017 (Fig. 2). Approximately 60% (*n* = 23,635) of the articles have been published since 2000.

### Journals and subject category

Forty journals published 39,305 articles, and 23.8% of them (*n* = 9355) were published by four journals with an IF > 30. *The Lancet* (9.1%; *n* = 3593), the *Journal of Clinical Oncology* (8.5%; *n* = 3343) and *The New England Journal of Medicine* (8.3%; *n* = 3275) published the largest number of articles, followed by *The BMJ* (6.4%; *n* = 2516) and *Circulation* (5.9%; *n* = 2331). Most articles were classified as “medicine, general & internal” (30.7%; *n* = 13,688); “cardiac & cardiovascular systems” (13.1%; *n* = 5828); or “oncology” (12.9%; *n* = 5760) according to the WoS journal categorisations (Table 2).

### Authors, institutions and countries

Most articles (62.3%; *n* = 24,496) were written by seven or more authors, and only 11.4% (*n* = 4469) of the articles were written by three or fewer authors. The first authors of the articles were based most commonly in North America and Western Europe; first authors from the United States were responsible for 36.9% (*n* = 14,508) of the articles (Table 2). We identified 17 authors who published 100 or more articles (Table 3). All of the most productive authors were male. The most prolific authors were Robert M. Califf, with 239 articles (from Duke University, United States); Eugene Braunwald, with 218 (from Harvard University, United States); Salim Yusuf, with 217 (from McMaster University, Canada); Eric J. Topol, with 212 (from Scripps Translational Science Institute, United States); Harvey D. White, with 186 (from University of Auckland, New Zealand); Lars Wallentin, with 144 (Uppsala University, Sweden); and Christopher B. Granger, with 140 (from Duke University, United States).

**Table 3:** Most productive authors and their institutions

Author	Affiliation and country	Articles	Citations	Citations per article	Articles in collaboration	Total signatures	Collaboration index (signatures per article)
Califf, Robert M.	Duke Clinical Research Institute, Duke University, United States	239	56,742	237.4	239	7919	33.1
Braunwald, Eugene	Brigham and Women’s Hospital, Harvard University, United States	218	63,764	292.5	218	8296	38.1
Yusuf, Salim	McMaster University, Canada	217	79,270	365.3	216	9163	42.4
Topol, Eric J.	Scripps Translational Science Institute, United States	212	48,523	228.9	212	6229	29.4
White, Harvey D.	Auckland City Hospital, University of Auckland, New Zealand	186	38,540	207.2	185	9133	49.4
Wallentin, Lars	Uppsala Clinical Research Centre, Uppsala University, Sweden	144	32,741	227.4	142	2958	20.8
Granger, Christopher B.	Duke Clinical Research Institute, Duke University, United States	140	29,668	211.9	140	5025	35.9
Stone, Gregg W.	New York-Presbyterian Hospital, Columbia University, United States	135	24,601	182.2	135	1928	14.3
Serruys, Patrick W.	Imperial College London, United Kingdom and Erasmus University, The Netherlands	133	27,302	205.3	133	2253	16.9
Armstrong, Paul W.	University of Alberta Hospital, University of Alberta, Canada	125	25,992	207.9	124	4573	36.9
McMurray, John J.V.	University of Glasgow, Scotland, United Kingdom	116	30,470	262.7	116	3425	29.5
Pfeffer, Marc A.	Brigham and Women’s Hospital, Harvard University, United States	116	46,032	396.8	115	4070	35.4
Gelber, Richard D.	Dana-Farber Cancer Institute, Harvard University, United States	107	20,898	195.3	107	3311	30.9
Van de Werf, Frans	Catholic University of Leuven, University Hospital Leuven, Belgium	107	21,918	204.8	105	4492	42.8
Harrington, Robert A.	Stanford University, United States	105	20,436	194.6	103	4971	48.3
Cannon, Christopher P.	Brigham and Women’s Hospital, Harvard University, United States	103	26,192	254.3	103	2241	21.8
Goldhirsch, Aron	European Institute of Oncology, Italy	103	16,254	157.8	98	3279	33.5

Note: Top authors with at least 100 articles

**Fig. 3:**
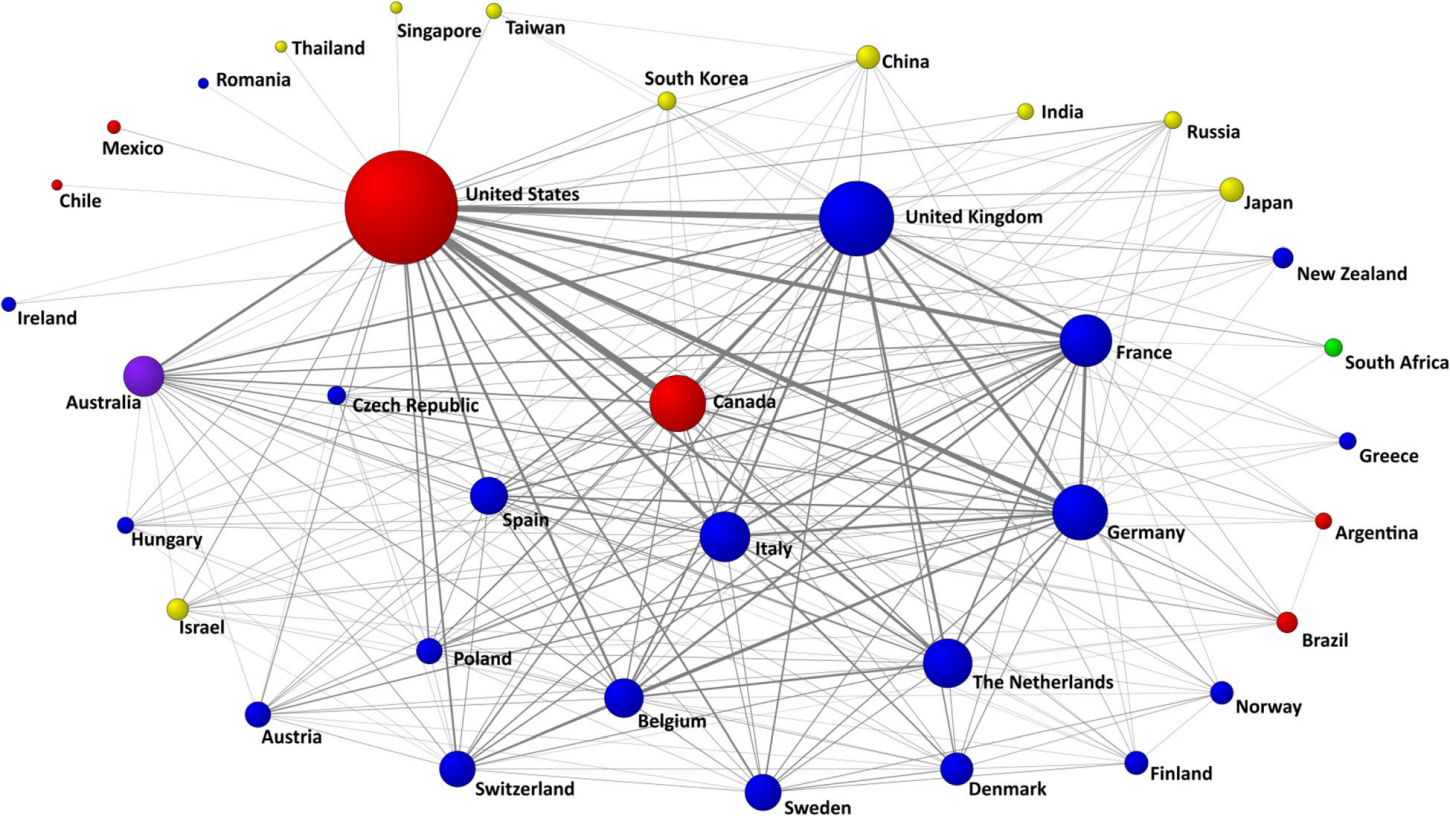
Global collaborative network between countries. Note: Most productive cluster of countries applying a threshold of 100 or more papers signed in co-authorship. Node sizes are proportional to the number of papers, and line thicknesses are proportional to the number of collaborations. Node colours: America = red; Asia = yellow; Africa = green; Europe = blue; Oceania = purple

Overall, 154 countries worldwide contributed to the analysed articles. The publication productivity ranking for countries (Table 4) was led by the United States (*n* = 18,393 articles, with 3.4 million citations), followed by the United Kingdom (*n* = 8028 articles, with 1.3 million citations), Canada (*n* = 4548 articles, with 1.0 million citations) and Germany (*n* = 4415 articles, with 0.9 million citations). A total of 37 countries had at least 100 articles in co-authorship. Figure 3 shows a visual representation of the most intense collaborative network between these 37 countries, in which we can see the relationships of some countries with respect to others and the position that each occupies in the network.

**Table 4:** Productivity and patterns of collaboration by top countries

Country	Total articles	Articles per million inhabitants	Total collaborations	Total citations	Citations per article	Articles in collaboration (distinct country)	Distinct countries of collaboration	Main collaborator (and number of collaborations)
United States	18,393	56.5	25,308	3,364,015	182.9	7895	130	Canada (2892)
United Kingdom	8028	121.6	18,529	1,345,597	167.6	4534	128	United States (2528)
Canada	4548	123.9	13,024	1,017,912	223.8	3552	108	United States (2892)
Germany	4415	53.4	16,463	894,026	202.5	3416	111	United States (2034)
France	3900	58.1	15,093	826,172	211.8	2928	119	United States (1759)
Italy	3608	59.6	13,005	674,367	186.9	2432	96	United States (1446)
The Netherlands	3453	201.5	10,578	619,589	179.4	2331	99	United States (1172)
Australia	2354	95.7	8787	469,341	199.4	1750	103	United States (1114)
Belgium	2197	193.2	10,685	482,974	219.8	2016	107	United States (1068)
Spain	2020	43.4	9747	417,291	206.6	1581	99	United States (1017)
Sweden	1909	189.6	7034	376,159	197.0	1483	91	United States (794)
Switzerland	1862	219.9	7840	348,734	187.3	1621	117	United States (818)
Denmark	1523	264.0	5572	297,936	195.6	1090	84	United States (582)
Poland	953	25.1	6577	222,301	233.3	916	80	United States (650)
Austria	945	107.3	4673	192,799	204.0	832	85	Germany (555)
Japan	820	6.5	2232	132,758	161.9	374	66	United States (289)
Finland	797	144.6	2950	195,500	245.3	556	81	United States (264)
China	771	0.6	3292	141,741	183.8	550	87	United States (391)
Norway	756	143.1	3076	152,184	201.3	605	70	Sweden (310)
Israel	661	75.9	3364	159,264	240.9	547	71	United States (382)
Brazil	626	3.0	4102	154,296	246.5	567	98	United States (440)
New Zealand	602	125.6	2229	106,318	176.6	480	80	United States (293)
Czech Republic	486	45.9	3757	107,543	221.3	475	82	United States (315)
South Korea	474	9.2	2748	80,338	169.5	383	78	United States (322)
South Africa	463	8.2	2468	89,382	193.0	422	95	United States (311)
Russia	425	2.9	3449	109,224	257.0	424	75	United States (336)
Greece	419	38.9	2114	59,496	142.0	285	75	United States (168)
Hungary	392	40.1	3192	93,590	238.7	383	74	United States (249)
Argentina	390	8.8	2736	117,289	300.7	357	91	United States (290)
India	370	0.3	2016	63,646	172.0	299	101	United States (214)
Taiwan	341	14.5	1803	77,089	226.1	242	63	United States (194)
Ireland	297	61.7	1366	60,867	204.9	263	79	United Kingdom (170)
Mexico	253	2.0	1754	56,256	222.4	230	84	United States (195)
Singapore	194	34.6	1210	42,265	217.9	176	80	United States (120)
Turkey	189	2.3	1288	34,928	184.8	149	79	United States (92)
Thailand	185	2.7	1268	55,360	299.2	177	93	United States (120)
Portugal	168	16.3	1218	24,177	143.9	160	61	Belgium (89), France (89)
Chile	157	8.7	1265	37,556	239.2	149	81	United States (112)
Romania	149	7.6	1406	31,392	210.7	148	77	United States (110)
Ukraine	113	2.5	1007	29,880	264.4	113	61	United States (84)
Uganda	112	2.6	405	14,931	133.3	108	72	United States (78)
Kenya	107	2.2	508	18,737	175.1	103	83	United States (68)

Note: Top countries with at least 100 articles. Country inhabitants (year 2017) obtained from the World Bank (http://data.worldbank.org/)

### Funding source

A total of 16,485 articles (41.9%) reported sources of funding. The 40 most frequent funding institutions (with 100 or more articles) are listed in Table 5. The main funders were the National Institutes of Health (NIH), with 7422 articles; Hoffmann-La Roche (*n* = 1188), Pfizer (*n* = 1139), Merck Sharp & Dohme (*n* = 1097) and Novartis (*n* = 1052).

**Table 5:** Most frequent funding institutions

Funding institution and country	Funding type	Articles	Citations	Citations per article	Articles in collaboration	Journals	Main journals (number of articles)
National Institutes of Health (NIH), United States	Non-industry	7422	1,312,297	176.8	2187	39	Journal of Clinical Oncology (1096), The New England Journal of Medicine (869), JAMA (614)
Hoffmann-La Roche, Switzerland	Industry	1188	218,428	183.9	997	36	The New England Journal of Medicine (159), Journal of Clinical Oncology (155), Lancet Oncology (96)
Pfizer, Inc., United States	Industry	1139	197,275	173.2	1001	36	The New England Journal of Medicine (187), JACC (112), Journal of Clinical Oncology (82)
Merck Sharp & Dohme (MSD), United States	Industry	1097	189,047	172.3	978	35	The New England Journal of Medicine (156), JACC (145), Diabetes Care (116)
Novartis, Switzerland	Industry	1052	192,229	182.7	900	34	The New England Journal of Medicine (157), JACC (107), Journal of Clinical Oncology (81)
Sanofi, France	Industry	987	170,078	172.3	920	34	JACC (152), Diabetes Care (142), The New England Journal of Medicine (135)
AstraZeneca, United Kingdom	Industry	938	168,667	179.8	841	33	JACC (166), The New England Journal of Medicine (131), Diabetes Care (101)
GlaxoSmithKline, United Kingdom	Industry	937	174,377	186.1	832	35	The New England Journal of Medicine (154), JACC (95), Diabetes Care (88)
Bristol-Myers Squibb (BMS), United States	Industry	924	186,731	202.1	843	33	The New England Journal of Medicine (141), JACC (121), Diabetes Care (119)
Johnson & Johnson, United States	Industry	873	153,063	175.3	791	34	The New England Journal of Medicine (136), JACC (113), Diabetes Care (90)
Abbott Laboratories, United States	Industry	843	141,475	167.8	758	33	JACC (218), The New England Journal of Medicine (129), Diabetes Care (71)
Eli Lilly and Company, United States	Industry	796	118,087	148.4	727	29	Diabetes Care (144), JACC (133), The New England Journal of Medicine (88)
Boehringer Ingelheim, Germany	Industry	600	125,626	209.4	537	31	The New England Journal of Medicine (115), Diabetes Care (89), JACC (78)
Medtronic, United States	Industry	565	95,746	169.5	525	25	JACC (186), The New England Journal of Medicine (84), Diabetes Care (65)
National Institute of Health Research (NIHR), United Kingdom	Non-industry	502	42,358	84.4	409	40	The Lancet (76), The BMJ (66), The New England Journal of Medicine (35)
Amgen Inc., United States	Industry	488	83,923	172.0	424	25	The New England Journal of Medicine (88), Journal of Clinical Oncology (65), JACC (64)
Bayer AG, Germany	Industry	487	107,327	220.4	453	33	The New England Journal of Medicine (89), JACC (84), Circulation (42)
Medical Research Council (MRC), United Kingdom	Non-industry	426	67,256	157.9	304	39	The Lancet (84), The BMJ (47), The New England Journal of Medicine (29)
Takeda Pharmaceutical Company, Japan	Industry	392	83,259	212.4	354	28	Diabetes Care (71), The New England Journal of Medicine (70), JACC (44)
National Health and Medical Research Council (NHMRC), Australia	Non-industry	338	35,116	103.9	276	38	Diabetes Care (34), The New England Journal of Medicine (34), The Lancet (34)
Daiichi Sankyo Company, Japan	Industry	336	52,054	154.9	325	18	JACC (105), Diabetes Care (46), The New England Journal of Medicine (43)
Boston Scientific Corporation, United States	Industry	317	45,609	143.9	291	14	JACC (140), Circulation (58), European Heart Journal (38)
Novo Nordisk, Denmark	Industry	306	44,994	147.0	273	26	Diabetes Care (162), The New England Journal of Medicine (38), The Lancet (24)
Gilead Sciences, United States	Industry	301	53,978	179.3	244	22	The New England Journal of Medicine (67), JACC (49), Hepatology (26)
Wellcome Trust, United Kingdom	Non-industry	276	32,785	118.8	142	36	The Lancet (67), The BMJ (34), PLOS Medicine (26)
Canadian Institutes of Health Research (CIHR), Canada	Non-industry	266	27,300	102.6	216	30	The New England Journal of Medicine (46), JAMA (29), The Lancet (22)
Cancer Research UK, United Kingdom	Non-industry	229	23,766	103.8	202	21	Lancet Oncology (64), Journal of Clinical Oncology (45), Annals of Oncology (27)
Allergan plc, Ireland	Industry	203	32,028	157.8	195	26	American Journal of Psychiatry (39), The New England Journal of Medicine (27), JACC (18)
Servier, France	Industry	199	33,106	166.4	194	20	JACC (51), The New England Journal of Medicine (29), European Heart Journal (24)
Astellas Pharma Inc., Japan	Industry	193	38,282	198.4	178	29	The New England Journal of Medicine (38), European Urology (18), JACC (16)
Teva Pharmaceutical Industries Ltd., Israel	Industry	175	29,128	166.4	167	24	The New England Journal of Medicine (29), Lancet Neurology (20), Journal of Allergy and Clinical Immunology (18)
The Medicines Company, United States	Industry	175	26,314	150.4	165	10	JACC (76), Circulation (29), The New England Journal of Medicine (28)
Eisai Co., Ltd., Japan	Industry	161	36,321	225.6	150	22	The New England Journal of Medicine (34), JACC (30), Circulation (16)
Merck KGaA, Germany	Industry	156	35,146	225.3	134	20	The New England Journal of Medicine (24), Annals of Oncology (21), Lancet Oncology (20)
Otsuka Pharmaceutical Co., Ltd., Japan	Industry	138	21,029	152.4	130	20	JACC (31), American Journal of Psychiatry (31), The New England Journal of Medicine (14)
Bill & Melinda Gates Foundation, United States	Non-industry	135	15,839	117.3	95	15	The Lancet (33), PLOS Medicine (27), The New England Journal of Medicine (24)
Celgene Corporation, United States	Industry	134	26,369	196.8	102	19	Journal of Clinical Oncology (31), The New England Journal of Medicine (26), Blood (20)
Federal Ministry of Education and Research (BMBF), Germany	Non-industry	119	11,409	95.9	104	28	Journal of Clinical Oncology (14), Blood (10), Diabetes Care (9)
UCB, Belgium	Industry	118	16,085	136.3	100	14	Annals of the Rheumatic Diseases (47), Lancet Neurology (14), The New England Journal of Medicine (13)
Biotronik, Germany	Industry	104	15,554	149.6	96	7	JACC (44), European Heart Journal (22), The New England Journal of Medicine (19)

### Most cited articles

Overall, included articles received 5.9 million citations, of which 83.1% of the citations (*n* = 4,950,604) corresponded to 15,142 (38.5%) articles with more than 100 citations. In addition, 641 (1.63%) articles with more than 1000 citations accounted for 20.7% of the total citations (*n* = 1,234,462). The most cited articles by number of citations (“100 citation classics”) are listed in Table 6. All of the most cited papers were published in English. These most cited articles were published in nine journals, led by *The New England Journal of Medicine*, with 78 articles, followed by *The Lancet* (*n* = 9) and *JAMA* (*n* = 7). The list of most cited papers contained innovative research methodologies. For example, the most cited article was a method paper published in *The Lancet* (“Bland-Altman method”) [35]. This seminal paper changed how method comparison studies are performed in clinical research. The list of the most cited papers also reflected important studies examining the health effects of pharmacological interventions on patients with chronic diseases. Common themes in major advances in health interventions included diabetes control [36–41]; the effects of hormone replacement therapy in postmenopausal women [42, 43]; therapies for diverse cancers such as glioblastoma, colorectal cancer, breast cancer, melanoma and hepatocellular carcinoma [44–50]; important interventional studies in the field of clinical cardiology, such as lipid-lowering statin therapy trials, antihypertensive trials, and antiplatelet and/or antithrombotic trials [51–63].

**Table 6:** Most cited articles

Rank	Articles	Article type	Total citations	Citations per year
1.	Bland JM, Altman DG. Statistical methods for assessing agreement between two methods of clinical measurement. Lancet. 1986;1(8476):307–10. PubMed PMID: 2868172.	Methods	30,217	974.7
2.	Diabetes Control and Complications Trial Research Group, Nathan DM, Genuth S, Lachin J, Cleary P, Crofford O, Davis M, Rand L, Siebert C. The effect of intensive treatment of diabetes on the development and progression of long-term complications in insulin-dependent diabetes mellitus. N Engl J Med. 1993;329(14):977–86. PubMed PMID: 8366922.	Original research	11,618	484.1
3.	UK Prospective Diabetes Study (UKPDS) Group, Turner RC, Holman RR, Cull CA, Stratton IM, Matthews DR, Frighi V, Manley SE, Neil A, McElroy K, Wright D, Kohner E, Fox C, Hadden D, Mehta Z, Smith A, Nugent Z, Peto R. Intensive blood-glucose control with sulphonylureas or insulin compared with conventional treatment and risk of complications in patients with type 2 diabetes (UKPDS 33). Lancet. 1998;352(9131):837–53. PubMed PMID: 9742976.	Original research	9895	520.8
4.	Rossouw JE, Anderson GL, Prentice RL, LaCroix AZ, Kooperberg C, Stefanick ML, Jackson RD, Beresford SA, Howard BV, Johnson KC, Kotchen JM, Ockene J; Writing Group for the Women’s Health Initiative Investigators. Risks and benefits of estrogen plus progestin in healthy postmenopausal women: principal results from the Women’s Health Initiative randomized controlled trial. JAMA. 2002;288(3):321–33. PubMed PMID: 12117397.	Original research	8962	597.5
5.	Knowler WC, Barrett-Connor E, Fowler SE, Hamman RF, Lachin JM, Walker EA, Nathan DM; Diabetes Prevention Program Research Group. Reduction in the incidence of type 2 diabetes with lifestyle intervention or metformin. N Engl J Med. 2002;346(6):393–403. PubMed PMID: 11832527.	Original research	8878	591.9
6.	Stupp R, Mason WP, van den Bent MJ, Weller M, Fisher B, Taphoorn MJ, Belanger K, Brandes AA, Marosi C, Bogdahn U, Curschmann J, Janzer RC, Ludwin SK, Gorlia T, Allgeier A, Lacombe D, Cairncross JG, Eisenhauer E, Mirimanoff RO; European Organisation for Research and Treatment of Cancer Brain Tumor and Radiotherapy Groups; National Cancer Institute of Canada Clinical Trials Group. Radiotherapy plus concomitant and adjuvant temozolomide for glioblastoma. N Engl J Med. 2005;352(10):987–96. PubMed PMID: 15758009.	Original research	8017	668.1
7.	Pedersen TR, Kjekshus J, Berg K, Haghfelt T, Faergeman O, Thorgeirsson G, Pyorala K, Miettinen T, Wilhelmsen L, Olsson AG, Wedel H, Kristianson K, Thomsen H, Nordero E, Thosen B, Lyngborg K. Randomised trial of cholesterol lowering in 4444 patients with coronary heart disease: the Scandinavian Simvastatin Survival Study (4S). Lancet. 1994;344(8934):1383–9. PubMed PMID: 7968073.	Original research	7924	344.5
8.	Hurwitz H, Fehrenbacher L, Novotny W, Cartwright T, Hainsworth J, Heim W, Berlin J, Baron A, Griffing S, Holmgren E, Ferrara N, Fyfe G, Rogers B, Ross R, Kabbinavar F. Bevacizumab plus irinotecan, fluorouracil, and leucovorin for metastatic colorectal cancer. N Engl J Med. 2004;350(23):2335–42. PubMed PMID: 15175435.	Original research	6835	525.8
9.	Slamon DJ, Leyland-Jones B, Shak S, Fuchs H, Paton V, Bajamonde A, Fleming T, Eiermann W, Wolter J, Pegram M, Baselga J, Norton L. Use of chemotherapy plus a monoclonal antibody against HER2 for metastatic breast cancer that overexpresses HER2. N Engl J Med. 2001;344(11):783–92. PubMed PMID: 11248153.	Original research	6507	406.7
10.	Shepherd J, Cobbe SM, Ford I, Isles CG, Lorimer AR, MacFarlane PW, McKillop JH, Packard CJ; West of Scotland Coronary Prevention Study Group. Prevention of coronary heart disease with pravastatin in men with hypercholesterolemia. N Engl J Med. 1995;333(20):1301–7. PubMed PMID: 7566020.	Original research	5933	269.7
11.	Hodi FS, O’Day SJ, McDermott DF, Weber RW, Sosman JA, Haanen JB, Gonzalez R, Robert C, Schadendorf D, Hassel JC, Akerley W, van den Eertwegh AJ, Lutzky J, Lorigan P, Vaubel JM, Linette GP, Hogg D, Ottensmeier CH, Lebbé C, Peschel C, Quirt I, Clark JI, Wolchok JD, Weber JS, Tian J, Yellin MJ, Nichol GM, Hoos A, Urba WJ. Improved survival with ipilimumab in patients with metastatic melanoma. N Engl J Med. 2010;363(8):711–23. PubMed PMID: 20525992.	Original research	5884	840.6
12.	Heart Outcomes Prevention Evaluation Study Investigators, Yusuf S, Sleight P, Pogue J, Bosch J, Davies R, Dagenais G. Effects of an angiotensin-converting-enzyme inhibitor, ramipril, on cardiovascular events in high-risk patients. N Engl J Med. 2000;342(3):145–53. PubMed PMID: 10639539.	Original research	5761	338.9
13.	National Institute of Neurological Disorders and Stroke rt-PA Stroke Study Group, Marler JR, Brott T, Broderick J, kothari R, Odonoghue M, Barsan W, Tomsick T, Spilker J, Miller R, Sauerbeck L, Jarrell J, Kelly J, Perkins T, McDonald T, Rorick M, Hickey C, Armitage J. Tissue plasminogen activator for acute ischemic stroke. N Engl J Med. 1995;333(24):1581–7. PubMed PMID: 7477192.	Original research	5533	251.5
14.	Tuomilehto J, Lindström J, Eriksson JG, Valle TT, Hämäläinen H, Ilanne-Parikka P, Keinänen-Kiukaanniemi S, Laakso M, Louheranta A, Rastas M, Salminen V, Uusitupa M; Finnish Diabetes Prevention Study Group. Prevention of type 2 diabetes mellitus by changes in lifestyle among subjects with impaired glucose tolerance. N Engl J Med. 2001;344(18):1343–50. PubMed PMID: 11333990.	Original research	5519	344.9
15.	Rivers E, Nguyen B, Havstad S, Ressler J, Muzzin A, Knoblich B, Peterson E, Tomlanovich M; Early Goal-Directed Therapy Collaborative Group. Early goal-directed therapy in the treatment of severe sepsis and septic shock. N Engl J Med. 2001;345(19):1368–77. PubMed PMID: 11794169.	Original research	5285	330.3
16.	van den Berghe G, Wouters P, Weekers F, Verwaest C, Bruyninckx F, Schetz M, Vlasselaers D, Ferdinande P, Lauwers P, Bouillon R. Intensive insulin therapy in critically ill patients. N Engl J Med. 2001;345(19):1359–67. PubMed PMID: 11,794,168.	Original research	5258	328.6
17.	Llovet JM, Ricci S, Mazzaferro V, Hilgard P, Gane E, Blanc JF, de Oliveira AC, Santoro A, Raoul JL, Forner A, Schwartz M, Porta C, Zeuzem S, Bolondi L, Greten TF, Galle PR, Seitz JF, Borbath I, Häussinger D, Giannaris T, Shan M, Moscovici M, Voliotis D, Bruix J; SHARP Investigators Study Group. Sorafenib in advanced hepatocellular carcinoma. N Engl J Med. 2008;359(4):378–90. PubMed PMID: 18650514.	Original research	5228	580.0
18.	Sacks FM, Pfeffer MA, Moye LA, Rouleau JL, Rutherford JD, Cole TG, Brown L, Warnica JW, Arnold JM, Wun CC, Davis BR, Braunwald E. The effect of pravastatin on coronary events after myocardial infarction in patients with average cholesterol levels. Cholesterol and Recurrent Events Trial investigators. N Engl J Med. 1996;335(14):1001–9. PubMed PMID: 8801446.	Original research	5205	247.9
19.	Collins R, Armitage J, Parish S, Sleight P, Peto R; Heart Protection Study Collaborative Group. MRC/BHF Heart Protection Study of cholesterol lowering with simvastatin in 20.536 high-risk individuals: a randomised placebo-controlled trial. Lancet. 2002;360(9326):7–22. PubMed PMID: 12114036.	Original research	5041	336.1
20.	Connolly SJ, Ezekowitz MD, Yusuf S, Eikelboom J, Oldgren J, Parekh A, Pogue J, Reilly PA, Themeles E, Varrone J, Wang S, Alings M, Xavier D, Zhu J, Diaz R, Lewis BS, Darius H, Diener HC, Joyner CD, Wallentin L; RE-LY Steering Committee and Investigators. Dabigatran versus warfarin in patients with atrial fibrillation. N Engl J Med. 2009;361(12):1139–51. PubMed PMID: 19717844.	Original research	4975	621.9
21.	Pitt B, Zannad F, Remme WJ, Cody R, Castaigne A, Perez A, Palensky J, Wittes J. The effect of spironolactone on morbidity and mortality in patients with severe heart failure. Randomized Aldactone Evaluation Study Investigators. N Engl J Med. 1999;341(10):709–17. PubMed PMID: 10471456.	Original research	4948	274.9
22.	Manns MP, McHutchison JG, Gordon SC, Rustgi VK, Shiffman M, Reindollar R, Goodman ZD, Koury K, Ling M, Albrecht JK. Peginterferon alfa-2b plus ribavirin compared with interferon alfa-2b plus ribavirin for initial treatment of chronic hepatitis C: a randomised trial. Lancet. 2001;358(9286):958–65. PubMed PMID: 11583749.	Original research	4757	297.3
23.	Fried MW, Shiffman ML, Reddy KR, Smith C, Marinos G, Gonçales FL Jr., Häussinger D, Diago M, Carosi G, Dhumeaux D, Craxi A, Lin A, Hoffman J, Yu J. Peginterferon alfa-2a plus ribavirin for chronic hepatitis C virus infection. N Engl J Med. 2002;347(13):975–82. PubMed PMID: 12324553.	Original research	4740	316.0
24.	Acute Respiratory Distress Syndrome Network, Brower RG, Matthay MA, Morris A, Schoenfeld D, Thompson BT, Wheeler A. Ventilation with lower tidal volumes as compared with traditional tidal volumes for acute lung injury and the acute respiratory distress syndrome. N Engl J Med. 2000;342(18):1301–8. PubMed PMID: 10793162.	Original research	4633	272.5
25.	Topalian SL, Hodi FS, Brahmer JR, Gettinger SN, Smith DC, McDermott DF, Powderly JD, Carvajal RD, Sosman JA, Atkins MB, Leming PD, Spigel DR, Antonia SJ, Horn L, Drake CG, Pardoll DM, Chen L, Sharfman WH, Anders RA, Taube JM, McMiller TL, Xu H, Korman AJ, Jure-Kunkel M, Agrawal S, McDonald D, Kollia GD, Gupta A, Wigginton JM, Sznol M. Safety, activity, and immune correlates of anti-PD-1 antibody in cancer. N Engl J Med. 2012;366(26):2443–54. PubMed PMID: 22658127.	Original research	4512	902.4
26.	Pfeffer MA, Braunwald E, Moyé LA, Basta L, Brown EJ Jr., Cuddy TE, Davis BR, Geltman EM, Goldman S, Flaker GC, Klein M, Lamas GA, Packer M, Rouleau J, Rouleau JL, Rutherford J, Wertheimer JH; The SAVE Investigators. Effect of captopril on mortality and morbidity in patients with left ventricular dysfunction after myocardial infarction. Results of the survival and ventricular enlargement trial. N Engl J Med. 1992;327(10):669–77. PubMed PMID: 1386652.	Original research	4437	177.5
27.	Hulley S, Grady D, Bush T, Furberg C, Herrington D, Riggs B, Vittinghoff E. Randomized trial of estrogen plus progestin for secondary prevention of coronary heart disease in postmenopausal women. Heart and Estrogen/progestin Replacement Study (HERS) Research Group. JAMA. 1998;280(7):605–13. PubMed PMID: 9718051.	Original research	4325	227.6
28.	North American Symptomatic Carotid Endarterectomy Trial Collaborators, Barnett HJM, Taylor DW, Haynes RB, Sackett DL, Peerless SJ, Ferguson GG, Fox AJ, Rankin RN, Hachinski VC, Wiebers DO, Eliasziw M. Beneficial effect of carotid endarterectomy in symptomatic patients with high-grade carotid stenosis. N Engl J Med. 1991;325(7):445–53. PubMed PMID: 1852179.	Original research	4315	166.0
29.	Mok TS, Wu YL, Thongprasert S, Yang CH, Chu DT, Saijo N, Sunpaweravong P, Han B, Margono B, Ichinose Y, Nishiwaki Y, Ohe Y, Yang JJ, Chewaskulyong B, Jiang H, Duffield EL, Watkins CL, Armour AA, Fukuoka M. Gefitinib or carboplatin-paclitaxel in pulmonary adenocarcinoma. N Engl J Med. 2009;361(10):947–57. PubMed PMID: 19692680.	Original research	4261	532.6
30.	Yusuf S, Zhao F, Mehta SR, Chrolavicius S, Tognoni G, Fox KK; Clopidogrel in Unstable Angina to Prevent Recurrent Events Trial Investigators. Effects of clopidogrel in addition to aspirin in patients with acute coronary syndromes without ST-segment elevation. N Engl J Med. 2001;345(7):494–502. PubMed PMID: 11519503.	Original research	4176	261.0
31.	Brenner BM, Cooper ME, de Zeeuw D, Keane WF, Mitch WE, Parving HH, Remuzzi G, Snapinn SM, Zhang Z, Shahinfar S; RENAAL Study Investigators. Effects of losartan on renal and cardiovascular outcomes in patients with type 2 diabetes and nephropathy. N Engl J Med. 2001;345(12):861–9. PubMed PMID: 11565518.	Original research	4164	260.3
32.	Lewis EJ, Hunsicker LG, Bain RP, Rohde RD. The effect of angiotensin-converting-enzyme inhibition on diabetic nephropathy. The Collaborative Study Group. N Engl J Med. 1993;329(20):1456–62. PubMed PMID: 8413456.	Original research	3965	165.2
33.	Chapman PB, Hauschild A, Robert C, Haanen JB, Ascierto P, Larkin J, Dummer R, Garbe C, Testori A, Maio M, Hogg D, Lorigan P, Lebbe C, Jouary T, Schadendorf D, Ribas A, O’Day SJ, Sosman JA, Kirkwood JM, Eggermont AM, Dreno B, Nolop K, Li J, Nelson B, Hou J, Lee RJ, Flaherty KT, McArthur GA; BRIM-3 Study Group. Improved survival with vemurafenib in melanoma with BRAF V600E mutation. N Engl J Med. 2011;364(26):2507–16. PubMed PMID: 21639808	Original research	3952	658.7
34.	Shepherd FA, Rodrigues Pereira J, Ciuleanu T, Tan EH, Hirsh V, Thongprasert S, Campos D, Maoleekoonpiroj S, Smylie M, Martins R, van Kooten M, Dediu M, Findlay B, Tu D, Johnston D, Bezjak A, Clark G, Santabárbara P, Seymour L; National Cancer Institute of Canada Clinical Trials Group. Erlotinib in previously treated non-small-cell lung cancer. N Engl J Med. 2005;353(2):123–32. PubMed PMID: 16014882.	Original research	3923	326.9
35.	CAPRIE Steering Committee, Gent M, Beaumont D, Blanchard J, Bousser MG, Coffman J, Easton JD, Hampton JR, Harker LA, Janzon L, Kusmierek JJ, Panak E, Roberts RS, Shannon JS, Sicurella J, Tognoni G, Topol EJ, Verstraet M, Warlow C, Cairns JA, Chesebro JH, von der Lippe G, Ross Russell RW, Wolf PA, Boissel JP, Friedman L, Fuster V, Harrison MG, Pocock S, et al. A randomised, blinded, trial of clopidogrel versus aspirin in patients at risk of ischaemic events (CAPRIE). Lancet. 1996;348(9038):1329–39. PubMed PMID: 8918275.	Original research	3902	185.8
36.	Ridker PM, Cushman M, Stampfer MJ, Tracy RP, Hennekens CH. Inflammation, aspirin, and the risk of cardiovascular disease in apparently healthy men. N Engl J Med. 1997;336(14):973–9. PubMed PMID: 9077376.	Original research	3874	193.7
37.	UK Prospective Diabetes Study (UKPDS) Group, Turner RC, Holman RR, Stratton IM, Cull CA, Matthews DR, Frighi V, Wright D, Neil A, Kohner E, McElroy K, Fox C, Hadden D, et al. Effect of intensive blood-glucose control with metformin on complications in overweight patients with type 2 diabetes (UKPDS 34). Lancet. 1998;352(9131):854–65. PubMed PMID: 9742977.	Original research	3861	203.2
38.	Moss AJ, Zareba W, Hall WJ, Klein H, Wilber DJ, Cannom DS, Daubert JP, Higgins SL, Brown MW, Andrews ML; Multicenter Automatic Defibrillator Implantation Trial II Investigators. Prophylactic implantation of a defibrillator in patients with myocardial infarction and reduced ejection fraction. N Engl J Med. 2002;346(12):877–83. PubMed PMID: 11907286.	Original research	3822	254.8
39.	Action to Control Cardiovascular Risk in Diabetes Study Group, Gerstein HC, Miller ME, Byington RP, Goff DC Jr., Bigger JT, Buse JB, Cushman WC, Genuth S, Ismail-Beigi F, Grimm RH Jr., Probstfield JL, Simons-Morton DG, Friedewald WT. Effects of intensive glucose lowering in type 2 diabetes. N Engl J Med. 2008;358(24):2545–59. PubMed PMID: 18539917.	Original research	3791	421.2
40.	Hansson L, Zanchetti A, Carruthers SG, Dahlöf B, Elmfeldt D, Julius S, Ménard J, Rahn KH, Wedel H, Westerling S. Effects of intensive blood-pressure lowering and low-dose aspirin in patients with hypertension: principal results of the Hypertension Optimal Treatment (HOT) randomised trial. HOT Study Group. Lancet. 1998;351(9118):1755–62. PubMed PMID: 9635947.	Original research	3773	198.6
41.	Sandler A, Gray R, Perry MC, Brahmer J, Schiller JH, Dowlati A, Lilenbaum R, Johnson DH. Paclitaxel carboplatin alone or with bevacizumab for non-small-cell lung cancer. N Engl J Med. 2006;355(24):2542–50. PubMed PMID: 17167137.	Original research	3772	342.9
42.	Fisher B, Costantino JP, Wickerham DL, Redmond CK, Kavanah M, Cronin WM, Vogel V, Robidoux A, Dimitrov N, Atkins J, Daly M, Wieand S, Tan-Chiu E, Ford L, Wolmark N. Tamoxifen for prevention of breast cancer: report of the National Surgical Adjuvant Breast and Bowel Project P-1 Study. J Natl Cancer Inst. 1998;90(18):1371–88. PubMed PMID: 9747868.	Original research	3757	197.7
43.	Bernard GR, Vincent JL, Laterre PF, LaRosa SP, Dhainaut JF, Lopez-Rodriguez A, Steingrub JS, Garber GE, Helterbrand JD, Ely EW, Fisher CJ Jr.; Recombinant human protein C Worldwide Evaluation in Severe Sepsis (PROWESS) study group. Efficacy and safety of recombinant human activated protein C for severe sepsis. N Engl J Med. 2001;344(10):699–709. PubMed PMID: 11236773.	Original research	3757	234.8
44.	Downs JR, Clearfield M, Weis S, Whitney E, Shapiro DR, Beere PA, Langendorfer A, Stein EA, Kruyer W, Gotto AM Jr. Primary prevention of acute coronary events with lovastatin in men and women with average cholesterol levels: results of AFCAPS/TexCAPS. Air Force/Texas Coronary Atherosclerosis Prevention Study. JAMA. 1998;279(20):1615–22. PubMed PMID: 9613910.	Original research	3720	195.8
45.	Long-Term Intervention with Pravastatin in Ischaemic Disease (LIPID) Study Group. Prevention of cardiovascular events and death with pravastatin in patients with coronary heart disease and a broad range of initial cholesterol levels. N Engl J Med. 1998;339(19):1349–57. PubMed PMID: 9841303.	Original research	3696	194.5
46.	Le Gall JR, Lemeshow S, Saulnier F. A new Simplified Acute Physiology Score (SAPS II) based on a European/North American multicenter study. JAMA. 1993;270(24):2957–63. PubMed PMID: 8254858.	Methods	3691	153.8
47.	Motzer RJ, Hutson TE, Tomczak P, Michaelson MD, Bukowski RM, Rixe O, Oudard S, Negrier S, Szczylik C, Kim ST, Chen I, Bycott PW, Baum CM, Figlin RA. Sunitinib versus interferon alfa in metastatic renal-cell carcinoma. N Engl J Med. 2007;356(2):115–24. PubMed PMID: 17215529.	Original research	3625	362.5
48.	Burris HA 3rd, Moore MJ, Andersen J, Green MR, Rothenberg ML, Modiano MR, Cripps MC, Portenoy RK, Storniolo AM, Tarassoff P, Nelson R, Dorr FA, Stephens CD, Von Hoff DD. Improvements in survival and clinical benefit with gemcitabine as first-line therapy for patients with advanced pancreas cancer: a randomized trial. J Clin Oncol. 1997;15(6):2403–13. PubMed PMID: 9196156.	Original research	3613	180.65
49	Patel MR, Mahaffey KW, Garg J, Pan G, Singer DE, Hacke W, Breithardt G, Halperin JL, Hankey GJ, Piccini JP, Becker RC, Nessel CC, Paolini JF, Berkowitz SD, Fox KA, Califf RM; ROCKET AF Investigators. Rivaroxaban versus warfarin in nonvalvular atrial fibrillation. N Engl J Med. 2011;365(10):883–91. PubMed PMID: 21830957.	Original research	3564	594.0
50.	Ridker PM, Danielson E, Fonseca FA, Genest J, Gotto AM Jr., Kastelein JJ, Koenig W, Libby P, Lorenzatti AJ, MacFadyen JG, Nordestgaard BG, Shepherd J, Willerson JT, Glynn RJ; JUPITER Study Group. Rosuvastatin to prevent vascular events in men and women with elevated C-reactive protein. N Engl J Med. 2008;359(21):2195–207. PubMed PMID: 18997196.	Original research	3488	387.6
51.	Serruys PW, de Jaegere P, Kiemeneij F, Macaya C, Rutsch W, Heyndrickx G, Emanuelsson H, Marco J, Legrand V, Materne P, et al. A comparison of balloon-expandable-stent implantation with balloon angioplasty in patients with coronary artery disease. Benestent Study Group. N Engl J Med. 1994;331(8):489–95. PubMed PMID: 8041413.	Original research	3473	151.0
52.	Cleland JG, Daubert JC, Erdmann E, Freemantle N, Gras D, Kappenberger L, Tavazzi L; Cardiac Resynchronization-Heart Failure (CARE-HF) Study Investigators. The effect of cardiac resynchronization on morbidity and mortality in heart failure. N Engl J Med. 2005;352(15):1539–49. PubMed PMID: 15753115.	Original research	3461	288.4
53.	Bardy GH, Lee KL, Mark DB, Poole JE, Packer DL, Boineau R, Domanski M, Troutman C, Anderson J, Johnson G, McNulty SE, Clapp-Channing N, Davidson-Ray LD, Fraulo ES, Fishbein DP, Luceri RM, Ip JH; Sudden Cardiac Death in Heart Failure Trial (SCD-HeFT) Investigators. Amiodarone or an implantable cardioverter-defibrillator for congestive heart failure. N Engl J Med. 2005;352(3):225–37. PubMed PMID: 15659722.	Original research	3448	290.7
54.	Wiviott SD, Braunwald E, McCabe CH, Montalescot G, Ruzyllo W, Gottlieb S, Neumann FJ, Ardissino D, De Servi S, Murphy SA, Riesmeyer J, Weerakkody G, Gibson CM, Antman EM; TRITON-TIMI 38 Investigators. Prasugrel versus clopidogrel in patients with acute coronary syndromes. N Engl J Med. 2007;357(20):2001–15. PubMed PMID: 17982182.	Original research	3439	343.9
55.	Lewis EJ, Hunsicker LG, Clarke WR, Berl T, Pohl MA, Lewis JB, Ritz E, Atkins RC, Rohde R, Raz I; Collaborative Study Group. Renoprotective effect of the angiotensin-receptor antagonist irbesartan in patients with nephropathy due to type 2 diabetes. N Engl J Med. 2001;345(12):851–60. PubMed PMID: 11565517.	Original research	3438	214.9
56.	SOLVD Investigators, Yusuf S, Pitt B, Davis CE, Hood WB, Cohn JN. Effect of enalapril on survival in patients with reduced left ventricular ejection fractions and congestive heart failure. N Engl J Med. 1991;325(5):293–302. PubMed PMID: 2057034.	Original research	3438	132.2
57.	Lieberman JA, Stroup TS, McEvoy JP, Swartz MS, Rosenheck RA, Perkins DO, Keefe RS, Davis SM, Davis CE, Lebowitz BD, Severe J, Hsiao JK; Clinical Antipsychotic Trials of Intervention Effectiveness (CATIE) Investigators. Effectiveness of antipsychotic drugs in patients with chronic schizophrenia. N Engl J Med. 2005;353(12):1209–23. PubMed PMID: 16172203.	Original research	3436	286.3
58.	Cunningham D, Humblet Y, Siena S, Khayat D, Bleiberg H, Santoro A, Bets D, Mueser M, Harstrick A, Verslype C, Chau I, Van Cutsem E. Cetuximab monotherapy and cetuximab plus irinotecan in irinotecan-refractory metastatic colorectal cancer. N Engl J Med. 2004;351(4):337–45. PubMed PMID: 15269313.	Original research	3430	263.8
59.	Dahlöf B, Devereux RB, Kjeldsen SE, Julius S, Beevers G, de Faire U, Fyhrquist F, Ibsen H, Kristiansson K, Lederballe-Pedersen O, Lindholm LH, Nieminen MS, Omvik P, Oparil S, Wedel H; LIFE Study Group. Cardiovascular morbidity and mortality in the Losartan Intervention For Endpoint reduction in hypertension study (LIFE): a randomised trial against atenolol. Lancet. 2002;359(9311):995–1003. PubMed PMID: 11937178.	Original research	3421	228.1
60.	Schiller JH, Harrington D, Belani CP, Langer C, Sandler A, Krook J, Zhu J, Johnson DH; Eastern Cooperative Oncology Group. Comparison of four chemotherapy regimens for advanced non-small-cell lung cancer. N Engl J Med. 2002;346(2):92–8. PubMed PMID: 11784875.	Original research	3413	227.5
61.	Cohen MS, Chen YQ, McCauley M, Gamble T, Hosseinipour MC, Kumarasamy N, Hakim JG, Kumwenda J, Grinsztejn B, Pilotto JH, Godbole SV, Mehendale S, Chariyalertsak S, Santos BR, Mayer KH, Hoffman IF, Eshleman SH, Piwowar-Manning E, Wang L, Makhema J, Mills LA, de Bruyn G, Sanne I, Eron J, Gallant J, Havlir D, Swindells S, Ribaudo H, Elharrar V, Burns D, Taha TE, Nielsen-Saines K, Celentano D, Essex M, Fleming TR; HPTN 052 Study Team. Prevention of HIV-1 infection with early antiretroviral therapy. N Engl J Med. 2011;365(6):493–505. PubMed PMID: 21767103.	Original research	3409	568.2
62.	Stearne MR, Palmer SL, Hammersley MS, Franklin SL, Spivey RS, Levy JC, Tidy CR, Bell NJ, Steemson J, Barrow BA, Coster R, Waring K, Nolan J, Truscott E, Walravens N, Cook L, Lampard H, Merle C, Parker P, McVittie J, et al. UK Prospective Diabetes Study Group. Tight blood pressure control and risk of macrovascular and microvascular complications in type 2 diabetes: UKPDS 38. BMJ. 1998;317(7160):703–13. PubMed PMID: 9732337.	Original research	3356	176.6
63.	Holman RR, Paul SK, Bethel MA, Matthews DR. Neil HA. 10-year follow-up of intensive glucose control in type 2 diabetes. N Engl J Med. 2008;359(15):1577–89. PubMed PMID: 18784090.	Secondary analysis	3349	372.1
64.	Escudier B, Eisen T, Stadler WM, Szczylik C, Oudard S, Siebels M, Negrier S, Chevreau C, Solska E, Desai AA, Rolland F, Demkow T, Hutson TE, Gore M, Freeman S, Schwartz B, Shan M, Simantov R, Bukowski RM; TARGET Study Group. Sorafenib in advanced clear-cell renal-cell carcinoma. N Engl J Med. 2007;356(2):125–34. PubMed PMID: 17215530.	Original research	3313	331.3
65.	Tannock IF, de Wit R, Berry WR, Horti J, Pluzanska A, Chi KN, Oudard S, Théodore C, James ND, Turesson I, Rosenthal MA, Eisenberger MA; TAX 327 Investigators. Docetaxel plus prednisone or mitoxantrone plus prednisone for advanced prostate cancer. N Engl J Med. 2004;351(15):1502–12. PubMed PMID: 15470213.	Original research	3286	252.8
66.	Romond EH, Perez EA, Bryant J, Suman VJ, Geyer CE Jr., Davidson NE, Tan-Chiu E, Martino S, Paik S, Kaufman PA, Swain SM, Pisansky TM, Fehrenbacher L, Kutteh LA, Vogel VG, Visscher DW, Yothers G, Jenkins RB, Brown AM, Dakhil SR, Mamounas EP, Lingle WL, Klein PM, Ingle JN, Wolmark N. Trastuzumab plus adjuvant chemotherapy for operable HER2-positive breast cancer. N Engl J Med. 2005;353(16):1673–84. PubMed PMID: 16236738.	Original research	3282	273.5
67.	Fischman DL, Leon MB, Baim DS, Schatz RA, Savage MP, Penn I, Detre K, Veltri L, Ricci D, Nobuyoshi M, Cleman M, Heuser R, Almond D, Teirstein PS, Fish RD, Colombo A, Brinker J, Moses J. A randomized comparison of coronary-stent placement and balloon angioplasty in the treatment of coronary artery disease. Stent Restenosis Study Investigators. N Engl J Med. 1994;331(8):496–501. PubMed PMID: 8041414.	Original research	3263	141.9
68.	Paik S, Shak S, Tang G, Kim C, Baker J, Cronin M, Baehner FL, Walker MG, Watson D, Park T, Hiller W, Fisher ER, Wickerham DL, Bryant J, Wolmark N. A multigene assay to predict recurrence of tamoxifen-treated, node negative breast cancer. N Engl J Med. 2004;351(27):2817–26. PubMed PMID: 15591335.	Original research	3249	249.9
69.	Bristow MR, Saxon LA, Boehmer J, Krueger S, Kass DA, De Marco T, Carson P, DiCarlo L, DeMets D, White BG, DeVries DW. Feldman AM; Comparison of Medical Therapy, Pacing, and Defibrillation in Heart Failure (COMPANION) Investigators. Cardiac-resynchronization therapy with or without an implantable defibrillator in advanced chronic heart failure. N Engl J Med. 2004;350(21):2140–50. PubMed PMID: 15152059.	Original research	3248	249.8
70.	ADVANCE Collaborative Group, Patel A, MacMahon S, Chalmers J, Neal B, Billot L, Woodward M, Marre M, Cooper M, Glasziou P, Grobbee D, Hamet P, Harrap S, Heller S, Liu L, Mancia G, Mogensen CE, Pan C, Poulter N, Rodgers A, Williams B, Bompoint S, de Galan BE, Joshi R, Travert F. Intensive blood glucose control and vascular outcomes in patients with type 2 diabetes. N Engl J Med. 2008;358(24):2560–72. PubMed PMID: 18539916.	Original research	3215	357.2
71.	Granger CB, Alexander JH, McMurray JJ, Lopes RD, Hylek EM, Hanna M, Al-Khalidi HR, Ansell J, Atar D, Avezum A, Bahit MC, Diaz R, Easton JD, Ezekowitz JA, Flaker G, Garcia D, Geraldes M, Gersh BJ, Golitsyn S, Goto S, Hermosillo AG, Hohnloser SH, Horowitz J, Mohan P, Jansky P, Lewis BS, Lopez-Sendon JL, Pais P, Parkhomenko A, Verheugt FW, Zhu J, Wallentin L; ARISTOTLE Committees and Investigators. Apixaban versus warfarin in patients with atrial fibrillation. N Engl J Med. 2011;365(11):981–92. PubMed PMID: 21870978.	Original research	3195	532.5
72.	Frick MH, Elo O, Haapa K, Heinonen OP, Heinsalmi P, Helo P, Huttunen JK, Kaitaniemi P, Koskinen P, Manninen V, Maenpaa H, Malkonen M, Manttari M, Norola S, Pasternack A, Pikkarainen J, Romo M, Sjöblom T, Nikiilä EA. Helsinki Heart Study: primary-prevention trial with gemfibrozil in middle-aged men with dyslipidemia. Safety of treatment, changes in risk factors, and incidence of coronary heart disease. N Engl J Med. 1987;317(20):1237–45. PubMed PMID: 3313041.	Original research	3109	103.6
73.	Kane J, Honigfeld G, Singer J, Meltzer H. Clozapine for the treatment-resistant schizophrenic. A double-blind comparison with chlorpromazine. Arch Gen Psychiatry. 1988;45(9):789–96. PubMed PMID: 3046553.	Original research	3098	106.8
74.	Leon MB, Smith CR, Mack M, Miller DC, Moses JW, Svensson LG, Tuzcu EM, Webb JG, Fontana GP, Makkar RR, Brown DL, Block PC, Guyton RA, Pichard AD, Bavaria JE, Herrmann HC, Douglas PS, Petersen JL, Akin JJ, Anderson WN, Wang D, Pocock S; PARTNER Trial Investigators. Transcatheter aortic-valve implantation for aortic stenosis in patients who cannot undergo surgery. N Engl J Med. 2010;363(17):1597–607. PubMed PMID: 20961243.	Original research	3079	439.9
75.	Coiffier B, Lepage E, Briere J, Herbrecht R, Tilly H, Bouabdallah R, Morel P, Van Den Neste E, Salles G, Gaulard P, Reyes F, Lederlin P, Gisselbrecht C. CHOP chemotherapy plus rituximab compared with CHOP alone in elderly patients with diffuse large-B-cell lymphoma. N Engl J Med. 2002;346(4):235–42. PubMed PMID: 11807147.	Original research	3077	205.1
76.	Packer M, Bristow MR, Cohn JN, Colucci WS, Fowler MB, Gilbert EM, Shusterman NH. The effect of carvedilol on morbidity and mortality in patients with chronic heart failure. U.S. Carvedilol Heart Failure Study Group. N Engl J Med. 1996;334(21):1349–55. PubMed PMID: 8614419.	Original research	3040	144.8
77.	Sauer R, Becker H, Hohenberger W, Rödel C, Wittekind C, Fietkau R, Martus P, Tschmelitsch J, Hager E, Hess CF, Karstens JH, Liersch T, Schmidberger H, Raab R; German Rectal Cancer Study Group. Preoperative versus postoperative chemoradiotherapy for rectal cancer. N Engl J Med. 2004;351(17):1731–40. PubMed PMID: 15496622.	Original research	3031	233.2
78.	Rosenfeld PJ, Brown DM, Heier JS, Boyer DS, Kaiser PK, Chung CY, Kim RY; MARINA Study Group. Ranibizumab for neovascular age-related macular degeneration. N Engl J Med. 2006;355(14):1419–31. PubMed PMID: 17021318.	Original research	3013	273.9
79.	Piccart-Gebhart MJ, Procter M, Leyland-Jones B, Goldhirsch A, Untch M, Smith I, Gianni L, Baselga J, Bell R, Jackisch C, Cameron D, Dowsett M, Barrios CH, Steger G, Huang CS, Andersson M, Inbar M, Lichinitser M, Láng I, Nitz U, Iwata H, Thomssen C, Lohrisch C, Suter TM, Rüschoff J, Suto T, Greatorex V, Ward C, Straehle C, McFadden E, Dolci MS, Gelber RD; Herceptin Adjuvant (HERA) Trial Study Team. Trastuzumab after adjuvant chemotherapy in HER2-positive breast cancer. N Engl J Med. 2005;353(16):1659–72. PubMed PMID: 16236737.	Original research	2993	249.4
80.	Moses JW, Leon MB, Popma JJ, Fitzgerald PJ, Holmes DR, O’Shaughnessy C, Caputo RP, Kereiakes DJ, Williams DO, Teirstein PS, Jaeger JL, Kuntz RE; SIRIUS Investigators. Sirolimus-eluting stents versus standard stents in patients with stenosis in a native coronary artery. N Engl J Med. 2003;349(14):1315–23. PubMed PMID: 14523139.	Original research	2992	213.7
81.	Bernard SA, Gray TW, Buist MD, Jones BM, Silvester W, Gutteridge G, Smith K. Treatment of comatose survivors of out-of-hospital cardiac arrest with induced hypothermia. N Engl J Med. 2002 Feb 21;346(8):557–63. PubMed PMID: 11856794.	Original research	2983	198.9
82.	Abraham WT, Fisher WG, Smith AL, Delurgio DB, Leon AR, Loh E, Kocovic DZ, Packer M, Clavell AL, Hayes DL, Ellestad M, Trupp RJ, Underwood J, Pickering F, Truex C, McAtee P, Messenger J; MIRACLE Study Group. Multicenter InSync Randomized Clinical Evaluation, Cardiac resynchronization in chronic heart failure. N Engl J Med. 2002;346(24):1845–53. PubMed PMID: 12063368.	Original research	2978	198.5
83.	Wallentin L, Becker RC, Budaj A, Cannon CP, Emanuelsson H, Held C, Horrow J, Husted S, James S, Katus H, Mahaffey KW, Scirica BM, Skene A, Steg PG, Storey RF, Harrington RA; PLATO Investigators, Freij A, Thorsén M. Ticagrelor versus clopidogrel in patients with acute coronary syndromes. N Engl J Med. 2009;361(11):1045–57. PubMed PMID: 19717846.	Original research	2961	370.1
84.	Walker M, Marler JR, Goldstein M, Grady PA, Toole JF, Baker WH, Castaldo JE, Chambless LE, Moore WS, Robertson JT, Young B, Howard VJ, Purvis S, Vernon DD, Needham K, Beck P, Celani VJ, Sauerbeck L, von Rajcs JA. Atkins D. Endarterectomy for asymptomatic carotid artery stenosis. Executive Committee for the Asymptomatic Carotid Atherosclerosis Study. JAMA. 1995;273(18):1421–8. PubMed PMID: 7723155.	Original research	2921	132.8
85.	Cannon CP, Braunwald E, McCabe CH, Rader DJ, Rouleau JL, Belder R, Joyal SV, Hill KA, Pfeffer MA, Skene AM; Pravastatin or Atorvastatin Evaluation and Infection Therapy-Thrombolysis in Myocardial Infarction 22 Investigators. Intensive versus moderate lipid lowering with statins after acute coronary syndromes. N Engl J Med. 2004;350(15):1495–504. PubMed PMID: 15007110.	Original research	2901	223.2
86.	Hacke W, Kaste M, Bluhmki E, Brozman M, Dávalos A, Guidetti D, Larrue V, Lees KR, Medeghri Z, Machnig T, Schneider D, von Kummer R, Wahlgren N, Toni D; ECASS Investigators. Thrombolysis with alteplase 3 to 4.5 h after acute ischemic stroke. N Engl J Med. 2008;359(13):1317–29. PubMed PMID: 18815396.	Original research	2896	321.8
87.	Anderson GL, Limacher M, Assaf AR, Bassford T, Beresford SA, Black H, Bonds D, Brunner R, Brzyski R, Caan B, Chlebowski R, Curb D, Gass M, Hays J, Heiss G, Hendrix S, Howard BV, Hsia J, Hubbell A, Jackson R, Johnson KC, Judd H, Kotchen JM, Kuller L, LaCroix AZ, Lane D, Langer RD, Lasser N, Lewis CE, Manson J, Margolis K, Ockene J, O’Sullivan MJ, Phillips L, Prentice RL, Ritenbaugh C, Robbins J, Rossouw JE, Sarto G, Stefanick ML, Van Horn L, Wactawski-Wende J, Wallace R, Wassertheil-Smoller S; Women’s Health Initiative Steering Committee. Effects of conjugated equine estrogen in postmenopausal women with hysterectomy: the Women’s Health Initiative randomized controlled trial. JAMA. 2004;291(14):1701–12. PubMed PMID: 15082697.	Original research	2836	218.2
88.	Bonner JA, Harari PM, Giralt J, Azarnia N, Shin DM, Cohen RB, Jones CU, Sur R, Raben D, Jassem J, Ove R, Kies MS, Baselga J, Youssoufian H, Amellal N, Rowinsky EK, Ang KK. Radiotherapy plus cetuximab for squamous-cell carcinoma of the head and neck. N Engl J Med. 2006;354(6):567–78. PubMed PMID: 16467544.	Original research	2836	257.8
89.	Stupp R, Hegi ME, Mason WP, van den Bent MJ, Taphoorn MJ, Janzer RC, Ludwin SK, Allgeier A, Fisher B, Belanger K, Hau P, Brandes AA, Gijtenbeek J, Marosi C, Vecht CJ, Mokhtari K, Wesseling P, Villa S, Eisenhauer E, Gorlia T, Weller M, Lacombe D, Cairncross JG, Mirimanoff RO; European Organisation for Research and Treatment of Cancer Brain Tumour and Radiation Oncology Groups; National Cancer Institute of Canada Clinical Trials Group. Effects of radiotherapy with concomitant and adjuvant temozolomide versus radiotherapy alone on survival in glioblastoma in a randomised phase III study: 5-year analysis of the EORTC-NCIC trial. Lancet Oncol. 2009 May;10(5):459–66. PubMed PMID: 19269895.	Secondary analysis	2799	349.9
90.	Morice MC, Serruys PW, Sousa JE, Fajadet J, Ban Hayashi E, Perin M, Colombo A, Schuler G, Barragan P, Guagliumi G, Molnàr F, Falotico R; RAVEL Study Group. Randomized Study with the Sirolimus-Coated Bx Velocity Balloon-Expandable Stent in the Treatment of Patients with de Novo Native Coronary Artery Lesions. A randomized comparison of a sirolimus-eluting stent with a standard stent for coronary revascularization. N Engl J Med. 2002;346(23):1773–80. PubMed PMID: 12050336.	Original research	2780	185.3
91.	Furberg CD, Wright JT, Davis BR, Cutler JA, Alderman M, Black H, Cushman W, Grimm R, Haywood LJ, Leenen F, Oparil S, Probstfield J, Whelton P, Nwachuku C, Gordon D, Proschan M, Einhorn P, et al. ALLHAT Officers and Coordinators for the ALLHAT Collaborative Research Group. The Antihypertensive and Lipid-Lowering Treatment to Prevent Heart Attack Trial. Major outcomes in high-risk hypertensive patients randomized to angiotensin-converting enzyme inhibitor or calcium channel blocker vs diuretic: The Antihypertensive and Lipid-Lowering Treatment to Prevent Heart Attack Trial (ALLHAT). JAMA. 2002;288(23):2981–97. PubMed PMID: 12479763.	Original research	2752	183.5
92.	Appel LJ, Moore TJ, Obarzanek E, Vollmer WM, Svetkey LP, Sacks FM, Bray GA, Vogt TM, Cutler JA, Windhauser MM, Lin PH, Karanja N. A clinical trial of the effects of dietary patterns on blood pressure. DASH Collaborative Research Group. N Engl J Med. 1997;336(16):1117–24. PubMed PMID: 9099655.	Original research	2741	137.1
93.	National Lung Screening Trial Research Team, Aberle DR, Adams AM, Berg CD, Black WC, Clapp JD, Fagerstrom RM, Gareen IF, Gatsonis C, Marcus PM, Sicks JD. Reduced lung-cancer mortality with low-dose computed tomographic screening. N Engl J Med. 2011 Aug 4;365(5):395–409. PubMed PMID: 21714641.	Original research	2726	454.3
94.	McHutchison JG, Gordon SC, Schiff ER, Shiffman ML, Lee WM, Rustgi VK, Goodman ZD, Ling MH, Cort S, Albrecht JK. Interferon alfa-2b alone or in combination with ribavirin as initial treatment for chronic hepatitis C. Hepatitis Interventional Therapy Group. N Engl J Med. 1998;339(21):1485–92. PubMed PMID: 9819446.	Original research	2725	143.4
95.	Pitt B, Remme W, Zannad F, Neaton J, Martinez F, Roniker B, Bittman R, Hurley S, Kleiman J, Gatlin M; Eplerenone Post-Acute Myocardial Infarction Heart Failure Efficacy and Survival Study Investigators. Eplerenone, a selective aldosterone blocker, in patients with left ventricular dysfunction after myocardial infarction. N Engl J Med. 2003;348(14):1309–21. PubMed PMID: 12668699.	Original research	2713	193.8
96.	CONSENSUS Trial Study Group. Effects of enalapril on mortality in severe congestive heart failure. Results of the Cooperative North Scandinavian Enalapril Survival Study (CONSENSUS). N Engl J Med. 1987 Jun 4;316(23):1429–35. PubMed PMID: 2883575.	Original research	2693	89.8
97.	Hébert PC, Wells G, Blajchman MA, Marshall J, Martin C, Pagliarello G, Tweeddale M, Schweitzer I, Yetisir E. A multicenter, randomized, controlled clinical trial of transfusion requirements in critical care. Transfusion Requirements in Critical Care Investigators. Canadian Critical Care Trials Group. N Engl J Med. 1999;340(6):409–17. PubMed PMID: 9971864.	Original research	2687	149.3
98.	Cardiac Arrhythmia Suppression Trial (CAST) Investigators. Preliminary report: effect of encainide and flecainide on mortality in a randomized trial of arrhythmia suppression after myocardial infarction. N Engl J Med. 1989;321(6):406–12. PubMed PMID: 2473403.	Secondary analysis	2683	95.8
99.	Demetri GD, von Mehren M, Blanke CD, Van den Abbeele AD, Eisenberg B, Roberts PJ, Heinrich MC, Tuveson DA, Singer S, Janicek M, Fletcher JA, Silverman SG, Silberman SL, Capdeville R, Kiese B, Peng B, Dimitrijevic S, Druker BJ, Corless C, Fletcher CD, Joensuu H. Efficacy and safety of imatinib mesylate in advanced gastrointestinal stromal tumors. N Engl J Med. 2002;347(7):472–80. PubMed PMID: 12181401.	Original research	2653	176.9
100.	Levey AS, Coresh J, Greene T, Stevens LA, Zhang YL, Hendriksen S, Kusek JW, Van Lente F; Chronic Kidney Disease Epidemiology Collaboration. Using standardized serum creatinine values in the modification of diet in renal disease study equation for estimating glomerular filtration rate. Ann Intern Med. 2006;145(4):247–54. PubMed PMID: 16908915.	Methods	2650	240.9

Note: Most cited (top-100) articles

### Common keywords

The most commonly used article keywords were “clinical trial” (16.1%; *n* = 6332 papers), followed by “therapy” (10.8%; *n* = 4267), “randomised controlled trial” (6.6%; *n* = 2587), “chemotherapy” (5.6%; *n* = 2224), “risk” (5.1%; *n* = 2026), “efficacy” (4.9%; *n* = 1933) and “double-blind” (4.9%; *n* = 1929). The most frequently used keywords in the most prolific journals are shown in Table 7. In addition, exploratory analyses of word clouds and networks based on keywords (co-words) showed the broad range of the topics covered (see Additional file 6).

**Table 7:** Most prolific journals and most commonly used keywords per journal

Journal subject category	Total articles	Journal name	Total articles	Keywords	Total articles
General medical journals	13,688	*The Lancet*	3593	Clinical trial	331
				Therapy	310
				Efficacy	175
				Management	167
				Risk	164
		*The New England Journal of Medicine*	3275	Clinical trial	875
				Randomised controlled trial	511
				Therapy	385
				Double-blind	238
				Risk	201
		*The BMJ*	2516	Clinical trial	151
				Management	109
				Intervention	96
				Therapy	88
				Risk	77
		*JAMA*	1904	Clinical trial	237
				Therapy	153
				Risk	136
				Meta-analysis	130
				Double-blind	127
		*JAMA Internal Medicine*	1122	Clinical trial	151
				Risk	98
				Randomised controlled trial	93
				Intervention	84
				Cardiovascular disease	81
		*Annals of Internal Medicine*	1097	Clinical trial	140
				Therapy	96
				Risk	75
				Disease	62
				Efficacy	55
Specialised medical journals	25,617	*Journal of Clinical Oncology*	3343	Chemotherapy	1028
				Clinical trial	772
				Therapy	646
				Survival	442
				Randomised controlled trial	385
		*Circulation*	2331	Myocardial infarction	690
				Clinical trial	618
				Cardiovascular disease	578
				Therapy	466
				Coronary heart disease	454
		*JAAC – Journal of the American College of Cardiology*	2133	Myocardial infarction	355
				Clinical trial	345
				Cardiovascular disease	281
				Mortality	258
				Randomised controlled trial	247
		*Diabetes Care*	1885	Diabetes mellitus	779
				Glycaemic control	305
				Clinical trial	272
				Therapy	225
				Risk	189
		*Journal of Allergy and Clinical Immunology*	1354	Asthma	446
				Children	364
				Double-blind	238
				Clinical trial	232
				Histamine	222
		*European Heart Journal*	1315	Clinical trial	231
				Myocardial infarction	228
				Mortality	159
				Therapy	159
				Cardiovascular disease	153
		*Annals of Oncology*	1119	Chemotherapy	448
				Clinical trial	353
				Therapy	236
				Survival	169
				Carcinoma	130
		*American Journal of Respiratory and Critical Care Medicine*	1011	Asthma	316
				Clinical trial	270
				Therapy	182
				Chronic obstructive pulmonary disease	178
				Bronchial hyperresponsiveness	148
		*Gastroenterology*	1008	Clinical trial	158
				Therapy	122
				Randomised controlled trial	86
				Inflammatory bowel disease	75
				Cirrhosis	63

Note: Journals with at least 1000 articles. Keyword data refer to the period 1990–2017

## Discussion

In this cross-sectional analysis, we presented a global mapping of RCT-related articles published in high-IF medical journals for the period 1965–2017. We identified the most prolific scientists, institutions and countries, main funding sources, most common subjects and topics, “citation classics” and most prolific high-IF medical journals from multiple specialties over the last 50 years.

In general, we found a strong clustering of articles published in British and American medical journals (*The Lancet, Journal of Clinical Oncology, The New England Journal of Medicine, The BMJ*, *Circulation, JAMA, JACC* and *Diabetes Care* accounted for 53% of the RCT-related articles). Many of these journals have been developed by active medical associations, both nationally and internationally. We hypothesize that different publishing patterns between journals may potentially reflect editorial policies and/or preferences, with some general medicine journals (such as *The Lancet and The New England Journal of Medicine*) and specialty journals (such as *Journal of Clinical Oncology* and *Circulation*), being more interested in and/or promoting the publication of RCTs. In contrast, a substantial number of these articles are behind publication paywalls (very few of the medical journals in our study sample are Open Access), and thus, research results may not be accessible to a large fraction of the scientific community and society as a whole, including clinicians (and patients) who may want them to help inform their clinical practice.

The results of this study highlight the expanding collaborative networks between countries in multiple regions, revealing a discernible scientific community, with the most productive countries having an important number of collaborations. Publication activity efforts were global during the study period, with articles from scientists and institutions in more than 150 different countries. However, the scientific community is centred on a nucleus of scientists from Western countries, with the most intense global collaborations taking place among the United States, United Kingdom and Canada. The presence and influence that these countries have on biomedical research [64–66] may be due to their large multi-stakeholder research partnerships, greater financial investment in clinical research, and high population of active scientists and research centres compared to other countries.

Publication activity worldwide shows that low- and middle-income countries have low levels of articles in high-IF medical journals. Difficulties in healthcare, education and research systems, information access and communication, language barriers and economic and institutional instability all represent challenges (and clear disadvantages) for productivity in low- and middle-income regions. In addition, restrictions and difficulties in conducting clinical research in resource-poor situations result in the exclusion of many of these countries from the planning, conduct and publication of RCTs [67–69]. As might be expected, our results support previous findings that low- and middle-income countries [31, 70, 71] had minimal contributions in articles published in major medical journals. For example, a previous study [70] showed that most of the authors of original papers published in five high-impact general medical journals (including *The New England Journal of Medicine*, *The Lancet*, *JAMA, The BMJ* and *Annals of Internal Medicine*) were more frequently affiliated with institutions in the same country as the journal. To address some of these problems, scientists, institutions and funders should promote collaborations (beyond historical, cultural and political factors) to share knowledge, expertise and innovative methodologies for clinical research. This may involve partnerships with Western countries to support capacity and resource development and research training.

RCT-related articles were published most often in high IF medical journals devoted to general and internal medicine, cardiology and oncology (nearly 57% of all articles). Similarly, the lists of the most cited articles identified topics which reflect major advances in the management of chronic conditions (such diabetes, cardiovascular disorders and cancer). The large relative productivity in general internal medicine, cardiology and oncology may be explained by the important role of randomised evidence to novel treatments and preventive strategies for these chronic diseases. In line with previous research [72–75], most of these highly cited RCTs addressed interventions for burdensome conditions that are health priorities in Western countries [76, 77]. Funding of (international, collaborative) RCTs may come from varying sources including commercial and non-commercial sponsors. However, previous analyses of RCT-related articles published in high-IF journals have suggested that study sponsors may influence how RCTs are designed, conducted and reported, sometimes serving financial rather than public interests [78]. Given that research funding is often restricted, the scientific community is responsible for using the available resources most efficiently when exploring research priorities to afford knowledge users and population health needs [76, 77, 79, 80].

Our findings suggest that women are vastly underrepresented in the group of most prolific scientists publishing in high-impact medical journals. This is in direct contrast to recent studies that have identified a gender gap in research publications [81–84]. For example, a previous study [84] showed that women in first authorship positions increased from 27% in 1994 to 37% in 2014 in leading medical journals (including *Annals of Internal Medicine*, *JAMA Internal Medicine*, *The BMJ*, *JAMA*, *The Lancet* and *The New England Journal of Medicine*), but progress has plateaued or declined since 2009. An urgent need exists to investigate the underlying causes of the potential gender gap to help identify publication practices and strategies to increase women’s influence [82, 84].

Several limitations exist in our study. First, we characterised the knowledge structures generated by a large number of articles published in major medical journals that are included in the WoS database. However, our results are limited to a subset of all clinical-trial-related articles published in 40 leading medical journals. We suspect that these articles represent those that have great implications for clinical practice and that are relevant to clinical practice guidelines and healthcare regulators. Although the publication production analysed has been drawn from an exhaustive analysis of the biomedical literature, possibly, the search missed some relevant articles (and journals). Some reports may be published in journals without being indexed as RCTs, making them difficult to identify. Second, as in many bibliometric analyses, the normalisation of the different names of an author, country and funding sources is fundamentally important to avoiding potential errors. We conducted a careful manual validation of the references and textual data to avoid typographical, transcription and/or indexing errors. However, we recognize this procedure does not assure complete certainty. Third, the affiliation addresses of authors do not necessarily reflect the country where the research was conducted or the research funding source. Fourth, topical analysis that extracts a set of unique keywords, word profiles and co-words may indicate intellectual organization in publication production, albeit with inherent limitations [85, 86]. Fifth, the use of citation analysis carries some problems [87–91]. A potential length time-effect bias exists, which puts the more recent articles at a disadvantage. In addition, the biomedical literature is rich in barriers and motivations for publication and citation preferences [87], including self-citation (bias towards one’s own work) [88], language bias (bias towards publishing and citing English articles), omission bias (bias whereby competitors are purposely not cited), and selective reporting and publication bias (bias in which “negative” results are withheld from publication and citation) [89–92]. In addition, citations are also treated as equal regardless of whether research is being cited for its positive contribution to the field or because it is being criticized. Finally, our methods represent only a mapping approach, which could be complemented further by more detailed analyses such as by examining the content (e.g. differences in journal or author characteristics between publicly funded and industry-funded studies, designs/methodology, etc.), the reporting and the reproducible research practices through research of research (“meta-research”) studies [92–98].

## Conclusion

The global analysis presented in this study provides evidence of the scientific growth of RCT- related articles published in high-IF medical journals. Over the last 50 years, publication activity in leading medical journals has increased, with Western countries (most notably, the United States) leading but with low- and middle-income countries showing very limited representation. Our analysis contributes to a better conceptualization and understanding of RCT articles and identified the main areas of research, the most influential publication sources chosen for their scientific dissemination and the major scientific leaders. Given the dynamic nature of the field, whether the growth trend remains the same in the coming years and how the characteristics of the field change over time will be interesting to see.

## Supplementary information

**Additional file 1.** Full strategy in PubMed/MEDLINE.

**Additional file 2.** Data extraction and normalisation processes.

**Additional file 3.** Reporting checklist.

**Additional file 4.** List of PMID for included articles.

**Additional file 5.** List of excluded articles.

**Additional file 6.** Exploratory analysis of topical data.

## Data Availability

With the publication of this manuscript, the full dataset will be freely available online in the Open Science Framework (https://osf.io/r2vw5/), a secure online repository for research data.
