# Supplementary material for: Global mapping of randomised trials related articles published in high-impact-factor medical journals: a cross-sectional analysis
Source: Trials. 2020 Jan 7;21:34. doi: 10.1186/s13063-019-3944-9 (PMC6947860; doi:10.1186/s13063-019-3944-9)
Supplement: Supplementary file 1 — Additional file 1. Full strategy in PubMed/MEDLINE. [file 13063_2019_3944_MOESM1_ESM.docx]

**Additional file 1. Full strategy in PubMed/MEDLINE.**

| **Search** | **Query** | **Items** |
| --- | --- | --- |
| #1 | "N Engl J Med"[Journal] OR "Lancet"[Journal] OR "JAMA"[Journal] OR "J Am Med Assoc"[Journal] OR "BMJ"[Journal] OR "Br Med J"[Journal] OR "Br Med J (Clin Res Ed)"[Journal] OR "Ann Intern Med"[Journal] OR "JAMA Intern Med"[Journal] OR "Arch Intern Med"[Journal] OR "PLoS Med"[Journal] OR "Lancet Oncol"[Journal] OR "World Psychiatry"[Journal] OR "Lancet Neurol"[Journal] OR "J Clin Oncol"[Journal] OR "Eur Heart J"[Journal] OR "J Am Coll Cardiol"[Journal] OR "Lancet Infect Dis"[Journal] OR "Lancet Diabetes Endocrinol"[Journal] OR "Circulation"[Journal] OR "Lancet Respir Med"[Journal] OR "Gastroenterology"[Journal] OR "Gut"[Journal] OR "JAMA Oncol"[Journal] OR "Eur Urol"[Journal] OR "JAMA Psychiatry"[Journal] OR "Arch Gen Psychiatry"[Journal] OR "Am J Psychiatry"[Journal] OR "Circ Res"[Journal] OR "Hepatology"[Journal] OR "Am J Respir Crit Care Med"[Journal] OR "Blood"[Journal] OR "J Allergy Clin Immunol"[Journal] OR "Ann Rheum Dis"[Journal] OR "J Natl Cancer Inst"[Journal] OR "J Hepatol"[Journal] OR "Intensive Care Med"[Journal] OR "Diabetes Care"[Journal] OR "Ann Oncol"[Journal] OR "Leukemia"[Journal] OR "Lancet Psychiatry"[Journal] OR "Eur Respir J"[Journal] OR "Brain"[Journal] OR "JAMA Pediatr"[Journal] OR "Arch Pediatr Adolesc Med"[Journal] OR "JAMA Neurol"[Journal] OR "Arch Neurol"[Journal] | 1001087 |
| #2 | (randomized controlled trial[Publication Type] OR (randomized[Title/Abstract] AND controlled[Title/Abstract] AND trial[Title/Abstract])) | 494058 |
| #3 | ("0001/01/01"[PDAT] : "2017/12/31"[PDAT]) | 27980439 |
| #4 | #1 AND #2 AND #3 | 41585 |
| #5 | (systematic review[title] OR review[publication type] OR meta-analy*[title] OR meta-analysis[publication type] OR pool analys*[title] OR pooled data [text word] OR letter [publication type] OR newspaper article [publication type]) | 3419286 |
| **#6** | **#4 NOT #5** | **39329** |
