# Supplementary material for: Global mapping of randomised trials related articles published in high-impact-factor medical journals: a cross-sectional analysis
Source: Trials. 2020 Jan 7;21:34. doi: 10.1186/s13063-019-3944-9 (PMC6947860; doi:10.1186/s13063-019-3944-9)
Supplement: Supplementary file 2 — Additional file 2. Data extraction and normalisation processes. [file 13063_2019_3944_MOESM2_ESM.docx]

**Additional file 2. Data extraction and normalisation processes**

| *Supplementary information on data extraction and normalisation*  For each included article, raw (meta)data on the journal and article titles, subject category, the year of publication, keywords, and the authors’ names, institutional affiliation(s), and country was downloaded online through the Web of Science (WoS) by a senior information specialist (A-AA). We also used the WoS to determine the extent to which each article had been cited in the scientific peer-review literature using the “times cited” number (that is, the number of times a publication has been cited by other publications). All these data were collected and entered into a Microsoft Access® (Microsoft, Seattle, WA, United States) database between May 7, 2018 and January 9, 2019 (see a brief overview of the database design below).  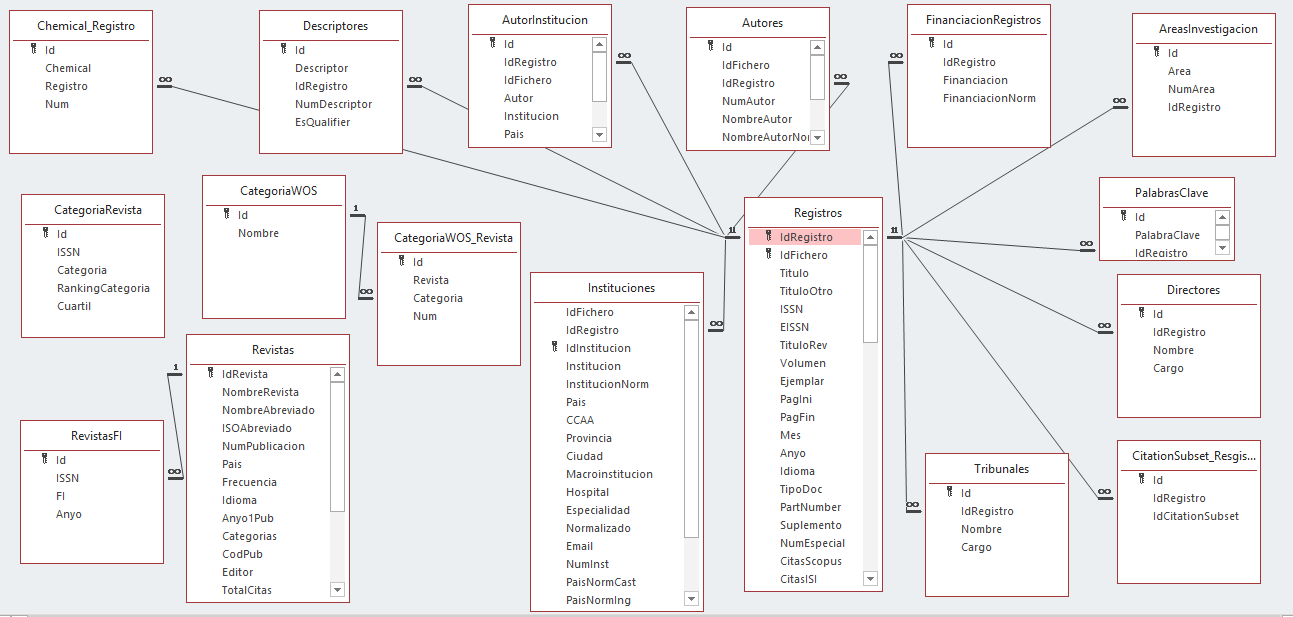  A process of normalisation was conducted by two researchers (AA-A, and FC-L or RA-B) to bring together the different names of an author, country, keywords and funding source.  *Author names*  One researcher (AA-A) checked the names by which an individual author appeared in two or more different forms (for example, “John McMurray “or “John J. McMurray” or “John J.V. McMurray”) using coincidence in that author’s place(s) of work as the basic criterion for normalisation (for example, University of Glasgow, Scotland, United Kingdom). A threshold of 30 articles was applied to review 200 names by which an author appeared in two or more different forms. Only authors with at least 100 articles were normalised. A second researcher (FC-L or RA-B) verified data. A full list of is provided for most productive authors:      Some authors reached the threshold of 30 articles with at least a name variant or form, but did not reach 100 articles in order to be categorised among the most productive authors. In those cases, the authorship information was not normalised. See some examples of checking with no normalisation are presented below.    *Country*  The normalisation of the countries was made based on the institutional affiliation of the authors. The WoS indexes the institutional addresses of all authors, not just first authors as is done in other databases (e.g. MEDLINE). This facilitated the construction of collaboration indicators and networks, as one could identify, at scale, collaborations patterns between affiliations at multiple levels of aggregation (countries, cities). If an article was published by several authors from the same country, it was counted as a single country. Missing data such as missing country information of an affiliated institution was substituted and completed by manual web search in some isolated cases (missing country data was observed in less than 200 out of a total of 232 000 institutional affiliations).  *Keywords*  We used both ‘‘author keywords’’ and ‘‘keyword plus,’’ which are automatically assigned by the WoS from the titles of the references of the articles because this approach has proven to be highly effective in representing the conceptual content of articles. A threshold of 100 articles was applied to review and normalise 359 most frequently keywords by which a keyword appeared in two or more different forms. Keywords in at least 500 articles were used for word cloud analyses. A second researcher (FC-L or RA-B) verified data.  To ensure consistency in the data, one researcher (RA-B) corrected keywords unifying grammatical variants and using only one keyword developed names of the same concept (for example, “randomized trial” or “randomized clinical trial” or “randomized controlled trial” or “randomised controlled trial”). In addition, the same researcher (RA-B) removed typographical, transcription and/or indexing errors, and a second researcher (FC-L) verified data. All potential discrepancies were resolved via consensus amongst these investigators.  Examples of keywords normalisation are presented below:        *Funding source*  During peer-review, reviewers suggested to further explore funding source. For this requested analysis, WoS metadata was downloaded. The WoS platform and therefore, the Science Citation Index database, systematically collects the information on funding source since 2008, so that we proceeded to normalise the funding institutions from the recovered data (a total of 59 423 institutional signatures). First, we downloaded data on funding source from WoS. After obtaining (meta)data, we needed to preprocess the data (e.g. lowercase letters, removal of typographical, transcription and/or indexing errors). We ordered main funding institutions by participation (publication productivity). Funding institutions having more than 50 participations were normalised, looking for all possible name variants. Overall, 35 012 different signatures have been normalised.  Given that funding institution names in many records included two or more institutions (for example, collaborations, partnerships, multiple sponsors), we proceeded to distinguish between these names by recording all variations of any individual institution as could be identified for each bibliographic record (for example, for “Eli Lilly & Co, GlaxoSmithKline” the standardization approach was to present “GlaxoSmithKline” separately from “Eli Lilly”).  When conducting normalisation, we used lists of major commercial and non-commercial sponsors of RCTs by using the European Union Trials Tracker (<https://eu.trialstracker.net/>). This information was supplemented by lists of major noncommercial sponsors of RCTs evaluated by DeVito et al. (reference: *DeVito NJ, French L, Goldacre B. Noncommercial funders’ policies on trial registration, access to summary results, and individual patient data availability .JAMA. 2018;319(16):1721-1723. doi:10.1001/jama.2018.2841)*  Examples of normalisation for funding institutions are presented below:   - GlaxoSmithKline (commercial funder)  \| Funding institution: GlaxoSmithKline \| Number or articles \| \| --- \| --- \| \| GlaxoSmithKline \| 773 \| \| GSK \| 46 \| \| GlaxoSmithKline (GSK) \| 9 \| \| GlaxoSmithKline Biologicals SA \| 8 \| \| GlaxoSmithKline Biologicals \| 7 \| \| Glaxo \| 6 \| \| GlaxoSmith Kline \| 5 \| \| Glaxo Smith Kline \| 4 \| \| Glaxo-SmithKline \| 4 \| \| GlaxoSmithKline Pharmaceuticals \| 4 \| \| GlaxoSmithKline Vaccines \| 4 \| \| GlaxoSmithKline, Collegeville, PA \| 4 \| \| Glaxo Smith Kline \| 3 \| \| GlaxoSmithKline Biologicals [NCT00122681/580299/008] \| 3 \| \| GlaxoSmithKline Pharma Denmark \| 3 \| \| GalxoSmithKline \| 2 \| \| Glaxo Smith Kline \| 2 \| \| Glaxo SmithKline \| 2 \| \| Glaxo Wellcome \| 2 \| \| GlaxoSmithKline [SCO30003] \| 2 \| \| GlaxoSmithKline Australia \| 2 \| \| GlaxoSmithKline Biologicals (GSK) \| 2 \| \| GlaxoSmithKline Pharmaceuticals, Philadelphia, PA \| 2 \| \| GlaxoSmithKline plc \| 2 \| \| GlaxoSmithKline, Uxbridge, UK \| 2 \| \| GlaxoSmithKline, plc \| 2 \| \| 2007 GlaxoSmithKline International Competitive Grant Award for Young Investigators (GlaxoSmithKline Research & Education Foundation for Cardiovascular Disease) \| 1 \| \| AAAAI/GlaxoSmithKline \| 1 \| \| Boehringer Ingelheim, GlaxoSmithKline \| 1 \| \| Cephalon, and GlaxoSmithKline \| 1 \| \| Eli Lilly & Co, GlaxoSmithKline \| 1 \| \| Endotis GlaxoSmithKline \| 1 \| \| Fausto Roila, GlaxoSmithKline, Merck \| 1 \| \| Forest Laboratories GlaxoSmithKline \| 1 \| \| GalaxoSmithKline \| 1 \| \| Genentech, GlaxoSmithKline \| 1 \| \| Gilead, GlaxoSmithKline \| 1 \| \| Glaxo Smith Klein \| 1 \| \| Glaxo-Smith Kline \| 1 \| \| GlaxoSmith Kline Biologicals \| 1 \| \| GlaxoSmithKIine \| 1 \| \| GlaxoSmithKiine (New Zealand) \| 1 \| \| GlaxoSmithKine \| 1 \| \| Glaxo-Smith-Kline \| 1 \| \| GlaxoSmith-Kline \| 1 \| \| GlaxoSmithKline and Sanofi-Aventis \| 1 \| \| GlaxoSmithKline Biologicals, Belgium \| 1 \| \| GlaxoSmithKline Nutrition \| 1 \| \| GlaxoSmithKline ( GSK), Greenford, UK [MEE103226] \| 1 \| \| GlaxoSmithKline (GSK) [HZC116601] \| 1 \| \| GlaxoSmithKline (GSK) [SAM106689] \| 1 \| \| GlaxoSmithKline (GSK) Biologicals SA \| 1 \| \| GlaxoSmithKline (Inst) \| 1 \| \| GlaxoSmithKline (King of Prussia, PA) \| 1 \| \| GlaxoSmithKline (Munich, Germany) \| 1 \| \| GlaxoSmithKline (Research Triangle Park, NC, USA) \| 1 \| \| GlaxoSmithKline (The Netherlands) \| 1 \| \| GlaxoSmithKline (the Netherlands) [SCO107656] \| 1 \| \| GlaxoSmithKline [109928] \| 1 \| \| GlaxoSmithKline [111782/NCT01362244] \| 1 \| \| GlaxoSmithKline [115921] \| 1 \| \| GlaxoSmithKline [201595] \| 1 \| \| GlaxoSmithKline [AC4115408] \| 1 \| \| GlaxoSmithKline [B2C109575] \| 1 \| \| GlaxoSmithKline [CRT110178] \| 1 \| \| GlaxoSmithKline [EGF106988] \| 1 \| \| GlaxoSmithKline [HZA106829] \| 1 \| \| GlaxoSmithKline [MEE103219] \| 1 \| \| GlaxoSmithKline [NCT00976391] \| 1 \| \| GlaxoSmithKline [NCT01227889] \| 1 \| \| GlaxoSmithKline [NCT01362296] \| 1 \| \| GlaxoSmithKline [SCO30003, NCT00268216] \| 1 \| \| GlaxoSmithKline and Development Limited \| 1 \| \| GlaxoSmithKline Biologicals (GSK) (US Food and Drug Administration) [BB-IND 7920] \| 1 \| \| GlaxoSmithKline Biologicals [ZOE-50, ZOE-70] \| 1 \| \| GlaxoSmithKline Biologicals SA (PATRICIA) \| 1 \| \| GlaxoSmithKline Biologicals, Belgium \| 1 \| \| GlaxoSmithKline Europe \| 1 \| \| GlaxoSmithKline Expert Testimony: Kathleen I. Pritchard \| 1 \| \| GlaxoSmithKline GmbH Co. \| 1 \| \| GlaxoSmithKline group of companies \| 1 \| \| GlaxoSmithKline in industry \| 1 \| \| GlaxoSmithKline of The Netherlands \| 1 \| \| GlaxoSmithKline Pharma Europe \| 1 \| \| GlaxoSmithKline Pharmaceuticals, Philadelphia, Pennsylvania \| 1 \| \| GlaxoSmithKline plc, UK. \| 1 \| \| GlaxoSmithKline PLC. \| 1 \| \| GlaxoSmithKline Research and Development \| 1 \| \| GlaxoSmithKline research grant \| 1 \| \| GlaxoSmithKline USA \| 1 \| \| GlaxoSmithKline, Collegeville, PA \| 1 \| \| GlaxoSmithKline, London, UK \| 1 \| \| GlaxoSmithKline, Medicines for Malaria Venture \| 1 \| \| GlaxoSmithKline, Verona, Italy \| 1 \| \| GlaxoSmithKline-Human Genome Sciences \| 1 \| \| GlaxoSmithKlineWellcome, Belgium \| 1 \| \| GlaxoSmithlKline \| 1 \| \| GlaxoSmithRline Biologicals SA \| 1 \| \| GlaxoWellcome \| 1 \| \| GSK [116935] \| 1 \| \| GSK Biologicals \| 1 \| \| GSK Biologicals SA \| 1 \| \| GSK Canada \| 1 \| \| GSK/Novartis \| 1 \| \| McMaster University/GlaxoSmithKline Chair in Lung Immunology at St Joseph´s Healthcare \| 1 \| \| Novartis/GlaxoSmithKline \| 1 \| \| Reliant Pharmaceuticals (acquired by GlaxoSmithKline) \| 1 \| \| SmithKline \| 1 \| \| SmithKline Beecham \| 1 \| \| ViiV Healthcare/GlaxoSmithKline \| 1 \|  - Medical Research Council (MRC) (non-commercial funder)  \| Funding institution: Medical Research Council (MRC) \| Number or articles \| \| --- \| --- \| \| Medical Research Council \| 57 \| \| UK Medical Research Council \| 56 \| \| MRC \| 17 \| \| United Kingdom Medical Research Council \| 10 \| \| Medical Research Council (UK) \| 8 \| \| UK MRC \| 8 \| \| Medical Research Council \| 7 \| \| Medical Research Council [G0700837] \| 6 \| \| UK Medical Research Council \| 5 \| \| Medical Research Council \| 4 \| \| Medical Research Council (MRC) \| 4 \| \| Medical Research Council [G9615910] \| 4 \| \| Medical Research Council [MC_U137686853] \| 4 \| \| Medical Research Council, UK \| 4 \| \| Medical Research Council [G0001164] \| 3 \| \| Medical Research Council [G0601303] \| 3 \| \| Medical Research Council [G9700808] \| 3 \| \| Medical Research Council [G9827821, G9827821(62595)] \| 3 \| \| Medical Research Council [MC_U122861331] \| 3 \| \| Medical Research Council [MC_U137686856] \| 3 \| \| U.K. Medical Research Council \| 3 \| \| Medical Research Council (MRC) \| 2 \| \| Medical Research Council (MRC) \| 2 \| \| Medical Research Council (United Kingdom) \| 2 \| \| Medical Research Council [G0300130] \| 2 \| \| Medical Research Council [G7900510] \| 2 \| \| Medical Research Council [G9600656] \| 2 \| \| Medical Research Council [G9900264] \| 2 \| \| Medical Research Council [MC_U122797165, MC_U122785831] \| 2 \| \| Medical Research Council [MC_U122797165] \| 2 \| \| Medical Research Council Clinical Trials Unit at University College London \| 2 \| \| Medical Research Council of the UK [G0200531] \| 2 \| \| Medical Research Council UK \| 2 \| \| MRC [G0701918] \| 2 \| \| MRC Clinical Trials Unit \| 2 \| \| UK Medical Research Council (via CTSU) \| 2 \| \| UK Medical Research Council \| 2 \| \| UK Medical Research Council [MR/K015338/1] \| 2 \| \| BHF, UK MRC \| 1 \| \| core MRC CTU funding \| 1 \| \| Efficacy and Mechanism Evaluation (EME) Programme, a Medical Research Council (MRC) \| 1 \| \| Efficacy and Mechanism Evaluation (EME) Programme, an MRC \| 1 \| \| Efficacy and Mechanism Evaluation Programme - Medical Research Council (MRC) \| 1 \| \| Efficacy and Mechanisms Evaluation programme of the Medical Research Council (MRC) \| 1 \| \| Medical Research Council (MRC) \| 1 \| \| Medical Research Council (STOP HCV) [MR/K01532X/1] \| 1 \| \| Medical Research Council [MC_U122861379] \| 1 \| \| Medical Research Council Bioinformatics Initiative \| 1 \| \| Medical Research Council Biomarkers grant \| 1 \| \| Medical Research Council Clinician Scientist Fellowship \| 1 \| \| Medical Research Council New Investigator Grant [G1002113] \| 1 \| \| Medical Research Council of Great Britain \| 1 \| \| Medical Research Council UK \| 1 \| \| Medical Research Council, UK \| 1 \| \| Medical Research Council (MRC) Clinical Trials Unit, London, UK \| 1 \| \| Medical Research Council (MRC) through the MRC Clinical Trials Unit \| 1 \| \| Medical Research Council [G0001164] \| 1 \| \| Medical Research Council [G0700837] \| 1 \| \| Medical Research Council in the UK \| 1 \| \| Medical Research Council UK (MRC) \| 1 \| \| Medical Research Council/Economic and Social Research Council \| 1 \| \| Medical Research Council/Economic and Social Research Council [G0800055] \| 1 \| \| Medical Research Council & Asthma UK Centre \| 1 \| \| Medical Research Council [G0001164, MC_U106179474] \| 1 \| \| Medical Research Council [G0200390] \| 1 \| \| Medical Research Council [G0501033] \| 1 \| \| Medical Research Council [G0600705] \| 1 \| \| Medical Research Council [G0900453] \| 1 \| \| Medical Research Council [MC_EX_G0400248, MC_EX_UU_G0400248, MC_UU_12023/11] \| 1 \| \| Medical Research Council [MC_U117588499] \| 1 \| \| Medical Research Council [MC_U122861330, MC_UU_12023/6] \| 1 \| \| Medical Research Council [MC_U122861383] \| 1 \| \| Medical Research Council [MC_U122886352] \| 1 \| \| Medical Research Council [MR/K010468/1] \| 1 \| \| Medical Research Council [MR/M003132/1] \| 1 \| \| Medical Research Council [MR/N002121/1] \| 1 \| \| Medical Research Council Clinical Trials Unit \| 1 \| \| Medical Research Council (London, UK) \| 1 \| \| Medical Research Council (MRC) [G9721265] \| 1 \| \| Medical Research Council (UK) [G0701592] \| 1 \| \| Medical Research Council [G0000340] \| 1 \| \| Medical Research Council [G0000934] \| 1 \| \| Medical Research Council [G0001078, MC_U122797164] \| 1 \| \| Medical Research Council [G0001104] \| 1 \| \| Medical Research Council [G0001164, MC_U106179474] \| 1 \| \| Medical Research Council [G0001354] \| 1 \| \| Medical Research Council [G0100070] \| 1 \| \| Medical Research Council [G0100496] \| 1 \| \| Medical Research Council [G0200585, MC_U122886352] \| 1 \| \| Medical Research Council [G0300133] \| 1 \| \| Medical Research Council [G0300195] \| 1 \| \| Medical Research Council [G0300411] \| 1 \| \| Medical Research Council [G0300497] \| 1 \| \| Medical Research Council [G0300653] \| 1 \| \| Medical Research Council [G0301005] \| 1 \| \| Medical Research Council [G0400426] \| 1 \| \| Medical Research Council [G0400491, MC_UP_A620_1014] \| 1 \| \| Medical Research Council [G0401546] \| 1 \| \| Medical Research Council [G0500264] \| 1 \| \| Medical Research Council [G0500300] \| 1 \| \| Medical Research Council [G0500495] \| 1 \| \| Medical Research Council [G0500783] \| 1 \| \| Medical Research Council [G0500966] \| 1 \| \| Medical Research Council [G0501019, MC_U122861330] \| 1 \| \| Medical Research Council [G0501019] \| 1 \| \| Medical Research Council [G0600989] \| 1 \| \| Medical Research Council [G0600998] \| 1 \| \| Medical Research Council [G0601031, U.1052.00.001] \| 1 \| \| Medical Research Council [G0601846] \| 1 \| \| Medical Research Council [G0700837, G0400456, G0501954] \| 1 \| \| Medical Research Council [G0701706] \| 1 \| \| Medical Research Council [G0800860] \| 1 \| \| Medical Research Council [G0900871] \| 1 \| \| Medical Research Council [G0901786] \| 1 \| \| Medical Research Council [G0902037, G8802774, G19/35, G0100222] \| 1 \| \| Medical Research Council [G1000183] \| 1 \| \| Medical Research Council [G1000469] \| 1 \| \| Medical Research Council [G1002046] \| 1 \| \| Medical Research Council [G106/1173, G0502028] \| 1 \| \| Medical Research Council [G116/172] \| 1 \| \| Medical Research Council [G8223452] \| 1 \| \| Medical Research Council [G84/5610] \| 1 \| \| Medical Research Council [G9000793] \| 1 \| \| Medical Research Council [G9401611] \| 1 \| \| Medical Research Council [G9439390, G0001237, G0001354] \| 1 \| \| Medical Research Council [G9439390] \| 1 \| \| Medical Research Council [G9533539] \| 1 \| \| Medical Research Council [G9533930] \| 1 \| \| Medical Research Council [G9534799] \| 1 \| \| Medical Research Council [G9626797] \| 1 \| \| Medical Research Council [G9810900] \| 1 \| \| Medical Research Council [G9827821(62595), G0700349, G9827821] \| 1 \| \| Medical Research Council [GO601295, MRC G0700288] \| 1 \| \| Medical Research Council [ISRCTN 26416991] \| 1 \| \| Medical Research Council [MC US A060 0016, RG62761] \| 1 \| \| Medical Research Council [MC_U105261167] \| 1 \| \| Medical Research Council [MC_U105960396] \| 1 \| \| Medical Research Council [MC_U106179474, G0001164] \| 1 \| \| Medical Research Council [MC_U106179474, MC_U106179471, G0200391, MC_U105260557, MC_U106179473] \| 1 \| \| Medical Research Council [MC_U106179474] \| 1 \| \| Medical Research Council [MC_U117588499] \| 1 \| \| Medical Research Council [MC_U120081323] \| 1 \| \| Medical Research Council [MC_U120084164] \| 1 \| \| Medical Research Council [MC_U122797165, G0701113] \| 1 \| \| Medical Research Council [MC_U122861325] \| 1 \| \| Medical Research Council [MC_U122861327] \| 1 \| \| Medical Research Council [MC_U122861330, G0501019] \| 1 \| \| Medical Research Council [MC_U122861375] \| 1 \| \| Medical Research Council [MC_U122861381] \| 1 \| \| Medical Research Council [MC_U122861384] \| 1 \| \| Medical Research Council [MC_U122861386] \| 1 \| \| Medical Research Council [MC_U122888468] \| 1 \| \| Medical Research Council [MC_U122888469] \| 1 \| \| Medical Research Council [MC_U130031238] \| 1 \| \| Medical Research Council [MC_U130059811] \| 1 \| \| Medical Research Council [MC_U132670597] \| 1 \| \| Medical Research Council [MC_U137686853, MC_EX_G0801669] \| 1 \| \| Medical Research Council [MC_U137686856, G0001160] \| 1 \| \| Medical Research Council [MC_U137686856, MC_U132670597] \| 1 \| \| Medical Research Council [MC_U137686857] \| 1 \| \| Medical Research Council [MC_U950770497] \| 1 \| \| Medical Research Council [MC_UP_A620_1014, G0400491, U1475000001] \| 1 \| \| Medical Research Council [MC_US_A060_0016, RG62761] \| 1 \| \| Medical Research Council [MC_UU_12023/6, MC_U122861330] \| 1 \| \| Medical Research Council [MC-A060-5PQ30, RG62761] \| 1 \| \| Medical Research Council [MR/J000620/1] \| 1 \| \| Medical Research Council [MR/L002515/1] \| 1 \| \| Medical Research Council [RG35746] \| 1 \| \| Medical Research Council Biostatistics Clinical Trials Methodology Hub \| 1 \| \| Medical Research Council Centre for Obesity and Related Metabolic Diseases \| 1 \| \| Medical Research Council Centre for Drug Safety Science \| 1 \| \| Medical Research Council Centre for Obesity and Related Metabolic Diseases \| 1 \| \| Medical Research Council Centre for Obesity and Related Metabolic Diseases \| 1 \| \| Medical Research Council ConDuCT Hub \| 1 \| \| Medical Research Council ConDuCT Hub \| 1 \| \| Medical Research Council Epidemiology Unit [MC_UU_12015/1, MC_UU_12015/5] \| 1 \| \| Medical research Council GB \| 1 \| \| Medical Research Council Human Nutrition Research [MC_UD99999906] \| 1 \| \| Medical Research Council of Great Britain [G0700392] \| 1 \| \| Medical Research Council of the United Kingdom \| 1 \| \| Medical Research Council through the Clinical Trials Unit \| 1 \| \| Medical Research Council through the MRC Clinical Trials Unit \| 1 \| \| Medical Research Council U. K. \| 1 \| \| Medical Research Council UK [G0701659/1] \| 1 \| \| Medical Research Council, British Heart Foundation \| 1 \| \| Medical Research Council, Cancer Research UK \| 1 \| \| Medical Research Council, Respiratory and Meningeal Pathogens Research Unit \| 1 \| \| Medical Research Council, UK [G0500274] \| 1 \| \| Medical Research Council, United Kingdom \| 1 \| \| Medical Research Council, United Kingdom [G0801439] \| 1 \| \| Medical Research Council/National Institute for Health Research Efficacy and Mechanism Evaluation Program \| 1 \| \| Medical Research Council/Wellcome Trust [WT089698] \| 1 \| \| Medical Research Council-UK [12/YH/0206] \| 1 \| \| MRC [G108/613] \| 1 \| \| MRC [MC_UU_12023/26] \| 1 \| \| MRC [08/99/08] \| 1 \| \| MRC [545926, 1010613, 1069985] \| 1 \| \| MRC [E743] \| 1 \| \| MRC [G001354] \| 1 \| \| MRC [G1002046, MR/K013386/1] \| 1 \| \| MRC [MR/K006185/1] \| 1 \| \| MRC [MR/K012924/1] \| 1 \| \| MRC [U.1052.00.006] \| 1 \| \| MRC [U105960371] \| 1 \| \| MRC British Thoracic Society/Morriston Davies Trust Capacity Building PhD Studentship \| 1 \| \| MRC Centenary Fellowship \| 1 \| \| MRC Centre grant [G1000758] \| 1 \| \| MRC Clinical Trials Unit at University College London \| 1 \| \| MRC Clinical Trials Unit at University College London \| 1 \| \| MRC ConDuCT Hub for trials methodology research \| 1 \| \| MRC CTU \| 1 \| \| MRC Myeloma IX trial \| 1 \| \| MRC National Prevention Research Initiative [G0802030] \| 1 \| \| MRC Partnership Grant [MR/L009242/1] \| 1 \| \| MRC Population Health Scientist Fellowship \| 1 \| \| MRC, United Kingdom \| 1 \| \| MRC/NCRI Adult Leukaemia Working Party \| 1 \| \| MRC-Asthma UK \| 1 \| \| MRC-NIHR \| 1 \| \| Pathfinder Award of the UK Medical Research Council [MRC] [G0401099] \| 1 \| \| The Medical Research Council \| 1 \| \| The UK Medical Research Council \| 1 \| \| U. K. Medical Research Council \| 1 \| \| U. K. Medical Research Council [G0100496] \| 1 \| \| U.K. Medical Research Council Epidemiology Unit Core Support [MC_UU_12015/5] \| 1 \| \| UK Medical Research Council \| 1 \| \| UK Medical Research Council (MRC) \| 1 \| \| UK Medical Research Council (MRC) [MC_UU_12013/5] \| 1 \| \| UK Medical Research Council [WM/3306381] \| 1 \| \| UK Department for International Development (DFID) (DMG) under the MRC/DFID Concordat agreement \| 1 \| \| UK Medical Research Council Clinical Research Training Fellowship [MR/K002406/1] \| 1 \| \| UK Medical Research Council (MRC) \| 1 \| \| UK Medical Research Council [G9600656] \| 1 \| \| UK Medical Research Council [MR/J003999/1] \| 1 \| \| UK Medical Research Council [RT01] \| 1 \| \| UK Medical Research Council [U1175.02.002.00014.01] \| 1 \| \| UK Medical Research Council (MRC) [G0300400, G1001190] \| 1 \| \| UK Medical Research Council (MRC) [G0500247] \| 1 \| \| UK Medical Research Council (MRC) [G0701013] \| 1 \| \| UK Medical Research Council (MRC) Epidemiology Unit [MC_UU_12015/1, MC_UU_12015/5] \| 1 \| \| UK Medical Research Council [84730] \| 1 \| \| UK Medical Research Council [G0500956] \| 1 \| \| UK Medical Research Council [G0600475] \| 1 \| \| UK Medical Research Council [G108/613] \| 1 \| \| UK Medical Research Council [G200212] \| 1 \| \| UK Medical Research Council [G9805643] \| 1 \| \| UK Medical Research Council [G9900306] \| 1 \| \| UK Medical Research Council [GO200471] \| 1 \| \| UK Medical Research Council [ISRCTN48489393] \| 1 \| \| UK Medical Research Council [MR/J015903/1] \| 1 \| \| UK Medical Research Council [MRC G0200434] \| 1 \| \| UK Medical Research Council Experimental Medicine [G0502131] \| 1 \| \| UK Medical Research Council, Primary Care Research Networks \| 1 \| \| UK MRC [G0400069, EME 09-800-15] \| 1 \| \| UK MRC [G0400074] \| 1 \| \| UK MRC [G1002375] \| 1 \| \| UK MRC Senior Clinical Fellowship [G0902308] \| 1 \| \| UK National Institute for Health Research Oxford Biomedical Research Centre Programme \| 1 \| \| United Kingdom Department for International Development (DFID) under the MRC/DFID Concordat agreement [MC-A760-5QX00] \| 1 \| \| United Kingdom Medical Research Council \| 1 \| \| United Kingdom Medical Research Council [AML15] \| 1 \| \| United Kingdom Medical Research Council [G9901427] \| 1 \| \| United Kingdom Medical Research Council [ISRCTN82375366] \| 1 \| \| University College London Medical Research Council Centre [G0601943] \| 1 \| |
| --- | --- | --- | --- | --- | --- | --- | --- | --- | --- | --- | --- | --- | --- | --- | --- | --- | --- | --- | --- | --- | --- | --- | --- | --- | --- | --- | --- | --- | --- | --- | --- | --- | --- | --- | --- | --- | --- | --- | --- | --- | --- | --- | --- | --- | --- | --- | --- | --- | --- | --- | --- | --- | --- | --- | --- | --- | --- | --- | --- | --- | --- | --- | --- | --- | --- | --- | --- | --- | --- | --- | --- | --- | --- | --- | --- | --- | --- | --- | --- | --- | --- | --- | --- | --- | --- | --- | --- | --- | --- | --- | --- | --- | --- | --- | --- | --- | --- | --- | --- | --- | --- | --- | --- | --- | --- | --- | --- | --- | --- | --- | --- | --- | --- | --- | --- | --- | --- | --- | --- | --- | --- | --- | --- | --- | --- | --- | --- | --- | --- | --- | --- | --- | --- | --- | --- | --- | --- | --- | --- | --- | --- | --- | --- | --- | --- | --- | --- | --- | --- | --- | --- | --- | --- | --- | --- | --- | --- | --- | --- | --- | --- | --- | --- | --- | --- | --- | --- | --- | --- | --- | --- | --- | --- | --- | --- | --- | --- | --- | --- | --- | --- | --- | --- | --- | --- | --- | --- | --- | --- | --- | --- | --- | --- | --- | --- | --- | --- | --- | --- | --- | --- | --- | --- | --- | --- | --- | --- | --- | --- | --- | --- | --- | --- | --- | --- | --- | --- | --- | --- | --- | --- | --- | --- | --- | --- | --- | --- | --- | --- | --- | --- | --- | --- | --- | --- | --- | --- | --- | --- | --- | --- | --- | --- | --- | --- | --- | --- | --- | --- | --- | --- | --- | --- | --- | --- | --- | --- | --- | --- | --- | --- | --- | --- | --- | --- | --- | --- | --- | --- | --- | --- | --- | --- | --- | --- | --- | --- | --- | --- | --- | --- | --- | --- | --- | --- | --- | --- | --- | --- | --- | --- | --- | --- | --- | --- | --- | --- | --- | --- | --- | --- | --- | --- | --- | --- | --- | --- | --- | --- | --- | --- | --- | --- | --- | --- | --- | --- | --- | --- | --- | --- | --- | --- | --- | --- | --- | --- | --- | --- | --- | --- | --- | --- | --- | --- | --- | --- | --- | --- | --- | --- | --- | --- | --- | --- | --- | --- | --- | --- | --- | --- | --- | --- | --- | --- | --- | --- | --- | --- | --- | --- | --- | --- | --- | --- | --- | --- | --- | --- | --- | --- | --- | --- | --- | --- | --- | --- | --- | --- | --- | --- | --- | --- | --- | --- | --- | --- | --- | --- | --- | --- | --- | --- | --- | --- | --- | --- | --- | --- | --- | --- | --- | --- | --- | --- | --- | --- | --- | --- | --- | --- | --- | --- | --- | --- | --- | --- | --- | --- | --- | --- | --- | --- | --- | --- | --- | --- | --- | --- | --- | --- | --- | --- | --- | --- | --- | --- | --- | --- | --- | --- | --- | --- | --- | --- | --- | --- | --- | --- | --- | --- | --- | --- | --- | --- | --- | --- | --- | --- | --- | --- | --- | --- | --- | --- | --- | --- | --- | --- | --- | --- | --- | --- | --- | --- | --- | --- | --- | --- | --- | --- | --- | --- | --- | --- | --- | --- | --- | --- | --- | --- | --- | --- | --- | --- | --- | --- | --- | --- | --- | --- | --- | --- | --- | --- | --- | --- | --- | --- | --- | --- | --- | --- | --- | --- | --- | --- | --- | --- | --- | --- | --- | --- | --- | --- | --- | --- | --- | --- | --- | --- | --- | --- | --- | --- | --- | --- | --- | --- | --- | --- | --- | --- | --- | --- | --- | --- | --- | --- | --- | --- | --- | --- | --- | --- | --- | --- | --- | --- | --- | --- | --- | --- | --- | --- | --- | --- | --- | --- | --- | --- | --- | --- | --- | --- | --- | --- | --- | --- | --- | --- | --- | --- | --- | --- | --- | --- | --- | --- | --- | --- | --- | --- | --- | --- | --- | --- | --- | --- | --- | --- | --- | --- | --- | --- | --- | --- | --- | --- | --- | --- | --- | --- | --- | --- | --- | --- | --- | --- | --- | --- | --- | --- | --- | --- | --- | --- | --- | --- | --- | --- | --- | --- | --- | --- | --- | --- | --- | --- | --- | --- | --- | --- | --- | --- | --- | --- | --- | --- | --- | --- | --- | --- | --- | --- | --- | --- | --- | --- | --- | --- | --- | --- | --- | --- | --- | --- | --- | --- | --- | --- | --- | --- | --- | --- | --- | --- | --- | --- | --- | --- | --- | --- | --- | --- | --- | --- | --- | --- | --- | --- | --- | --- | --- | --- | --- | --- | --- | --- | --- | --- | --- | --- | --- | --- | --- | --- | --- | --- | --- | --- | --- | --- | --- | --- | --- | --- | --- | --- | --- | --- | --- | --- | --- | --- | --- | --- | --- | --- | --- | --- | --- | --- | --- | --- | --- | --- | --- | --- | --- | --- | --- | --- | --- | --- | --- | --- | --- | --- | --- | --- | --- |
