# Supplementary material for: Global mapping of randomised trials related articles published in high-impact-factor medical journals: a cross-sectional analysis
Source: Trials. 2020 Jan 7;21:34. doi: 10.1186/s13063-019-3944-9 (PMC6947860; doi:10.1186/s13063-019-3944-9)
Supplement: Supplementary file 3 — Additional file 3. Reporting checklist. [file 13063_2019_3944_MOESM3_ESM.docx]

**Additional file 3. Reporting checklist**

Note of the authors: We are not aware of any reporting guideline for bibliometric/mapping analysis of the biomedical literature. Instead, we have reported the present (systematic) cross-sectional analysis using the PRISMA statement for systematic reviews.

| **Section/Topic** | **Item #** | **Checklist Item** | **Reported on Page #** |
| --- | --- | --- | --- |
| **TITLE** |  |  |  |
| Title | 1 | Identify the report as a systematic review, meta-analysis (or related form of meta-analysis). | **p.1 (cross-sectional, mapping)** |
| **ABSTRACT** |  |  |  |
| Structured summary | 2 | Provide a structured summary including, as applicable:  **Background:** main objectives  **Methods:** data sources; study eligibility criteria, participants, and interventions; study appraisal; and *synthesis methods*  **Results:** number of studies and participants identified; summary estimates with corresponding confidence/credible intervals; *…*  **Discussion/Conclusions:** limitations; conclusions and implications of findings.  **Other:** primary source of funding; systematic review registration number with registry name. | **p.2** |
| **INTRODUCTION** |  |  |  |
| Rationale | 3 | Describe the rationale for the review in the context of what is already known | **p.3** |
| Objectives | 4 | Provide an explicit statement of questions being addressed, with reference to participants, interventions, comparisons, outcomes, and study design (PICOS). | **p.3** |
| **METHODS** |  |  |  |
| Protocol and registration | 5 | Indicate whether a review protocol exists and if and where it can be accessed (e.g., Web address); and, if available, provide registration information, including registration number. | **NA** |
| Eligibility criteria | 6 | Specify study characteristics (e.g., PICOS, length of follow-up) and report characteristics (e.g., years considered, language, publication status) used as criteria for eligibility, giving rationale. | **p.3 and 4** |
| Information sources | 7 | Describe all information sources (e.g., databases with dates of coverage, contact with study authors to identify additional studies) in the search and date last searched. | **p.3** |
| Search | 8 | Present full electronic search strategy for at least one database, including any limits used, such that it could be repeated. | **p.3 and S1 Table** |
| Study selection | 9 | State the process for selecting studies (i.e., screening, eligibility, included in systematic review, and, if applicable, included in the meta-analysis). | **p.4** |
| Data collection process | 10 | Describe method of data extraction from reports (e.g., piloted forms, independently, in duplicate) and any processes for obtaining and confirming data from investigators. | **p.4 and S2 Table** |
| Data items | 11 | List and define all variables for which data were sought (e.g., PICOS, funding sources) and any assumptions and simplifications made. | **p.4 and 5. Tables 1-5** |
| Risk of bias within individual studies | 12 | Describe methods used for assessing risk of bias of individual studies (including specification of whether this was done at the study or outcome level), and how this information is to be used in any data synthesis. | **NA** |
| Summary measures | 13 | State the principal summary measures (e.g., risk ratio, difference in means). | **NA** |
| Planned methods of analysis | 14 | Describe the methods of handling data and combining results of studies for each meta-analysis. … | **p.4 and 5** |
| Risk of bias across studies | 15 | Specify any assessment of risk of bias that may affect the cumulative evidence (e.g., publication bias, selective reporting within studies). | **NA** |
| Additional analyses | 16 | Describe methods of additional analyses if done, indicating which were pre-specified. This may include, but not be limited to, the following:   - Sensitivity or subgroup analyses; - Meta-regression analyses; | **NA** |
| **RESULTS** |  |  |  |
| Study selection | 17 | Give numbers of studies screened, assessed for eligibility, and included in the review, with reasons for exclusions at each stage, ideally with a flow diagram. | **p.5 and Fig 1** |
| Study characteristics | 18 | For each study, present characteristics for which data were extracted (e.g., study size, PICOS, follow-up period) and provide the citations. | **p.5, 6 and 7, Table 1** |
| Risk of bias within studies | 19 | Present data on risk of bias of each study and, if available, any outcome level assessment. | **NA** |
| Results of individual studies | 20 | For all outcomes considered (benefits or harms), present, for each study: 1) simple summary data for each intervention group, and 2) effect estimates and confidence intervals. | **p. 5-7 Tables, Fig** |
| Synthesis of results | 21 | Present results of each meta-analysis done, including confidence intervals. | **p. 5-7 Tables, Fig** |
| Risk of bias across studies | 22 | Present results of any assessment of risk of bias across studies for the evidence base being studied. | **NA** |
| Results of additional analyses | 23 | Give results of additional analyses, if done (e.g., sensitivity or subgroup analyses, meta-regression analyses*,…* | **NA** |
|  |  |  |  |
| **DISCUSSION** |  |  |  |
| Summary of evidence | 24 | Summarize the main findings, including the strength of evidence for each main outcome; consider their relevance to key groups (e.g., healthcare providers, users, and policy-makers). | **p. 7-9** |
| Limitations | 25 | Discuss limitations at study and outcome level (e.g., risk of bias), and at review level (e.g., incomplete retrieval of identified research, reporting bias). | **p.8** |
| Conclusions | 26 | Provide a general interpretation of the results in the context of other evidence, and implications for future research. | **p.9** |
| **FUNDING** |  |  |  |
| Funding | 27 | Describe sources of funding for the systematic review and other support (e.g., supply of data); role of funders for the systematic review. … | **p.9** |
