# Supplementary material for: Global mapping of randomised trials related articles published in high-impact-factor medical journals: a cross-sectional analysis
Source: Trials. 2020 Jan 7;21:34. doi: 10.1186/s13063-019-3944-9 (PMC6947860; doi:10.1186/s13063-019-3944-9)
Supplement: Supplementary file 4 — Additional file 4. List of PMID for included articles. [file 13063_2019_3944_MOESM4_ESM.docx]

**Additional file 4. List of PMID for included articles.**

| **Num. PMID** |
| --- |
| 5891291, 5849145, 4158821, 4159214, 5321112, 4158171, 4165407, 5318612, 4157501, 4158801, 5321594, 5849135, 4159504, 4159700, 5326281, 4163541, 4162365, 5933438, 4161161, 4162366, 4159070, 4159071, 5331178, 4378175, 5908884, 5323069, 4160846, 4222374, 5900912, 5917939, 5912512, 4160958, 5910109, 5951811, 4160515, 5909480, 5952173, 5951838, 4161592, 4162526, 4158993, 5952104, 5956365, 5951878, 5327222, 4160268, 5952290, 5331424, 5322596, 4161966, 5926130, 5926263, 5331179, 4161718, 5917835, 5926260, 5331176, 4161606, 4158954, 5323049, 5322503, 4285721, 5332983, 5332542, 4163441, 4159007, 5951795, 5917405, 4159956, 4162503, 4143638, 4379597, 4162550, 5951890, 5322863, 4163901, 4163915, 4164601, 4163309, 4166895, 6079807, 4163308, 4293374, 4964192, 4165041, 5334748, 5333307, 6072348, 6054003, 4952167, 6081001, 4167518, 6020856, 6038339, 5339171, 5340622, 5340332, 4165154, 4167854, 4166108, 6038318, 4865575, 4165134, 5338679, 4163975, 4862069, 4952779, 4964135, 4167249, 5333306, 4164367, 4964132, 6016060, 6016061, 4166100, 6027134, 6072705, 6015520, 6058613, 4164175, 6066689, 6053945, 4964853, 5335042, 6020993, 4170088, 6038338, 4165918, 4163895, 6072493, 4166904, 6017526, 6059259, 4165466, 4163741, 6072816, 4166889, 4163775, 6036431, 4166334, 4177467, 5656294, 5695575, 4175157, 4174153, 4887691, 5656325, 4174507, 4170432, 4173263, 5655030, 5639627, 4872151, 5649002, 4175156, 4867447, 4169080, 4170945, 4969551, 4870069, 4301534, 4865235, 4971442, 4873660, 5655196, 4176868, 4171584, 4175085, 4173259, 4879059, 4173262, 4877801, 5661963, 5670905, 4173404, 5694257, 5683581, 4170163, 5647535, 5658018, 4871610, 4301207, 4881421, 4170164, 4174530, 4174488, 4865234, 5667468, 4176294, 4971563, 4174535, 4171333, 5717967, 4171838, 4170656, 4298260, 5640926, 5238810, 4170275, 4867936, 5640925, 4867934, 5634718, 4969991, 4870075, 5673964, 4868098, 4294840, 4881341, 4872152, 4872154, 4184834, 4899454, 4179897, 4186598, 4902196, 4180817, 4389442, 5354851, 5386267, 4182654, 5764843, 5782719, 4178422, 5774314, 5258858, 4885307, 4886627, 4884653, 5800365, 4894463, 5762643, 4888873, 4186452, 4186450, 4178243, 4888353, 4885615, 4180816, 4179565, 4178418, 4896164, 4178789, 4904222, 4186281, 4895341, 4186992, 4897366, 4186168, 5781136, 5784612, 4889121, 4897625, 4179885, 4185887, 5354877, 4181730, 4183130, 4308796, 4306322, 4897812, 5819227, 4185613, 4884671, 4895340, 5350103, 4187998, 4185445, 4388775, 4182413, 4179167, 4311188, 4883649, 5800368, 4179194, 4179353, 4887601, 5774077, 4186975, 4180215, 4916870, 5818187, 4307139, 4885975, 4895339, 4895338, 4183971, 4890207, 4885972, 4187544, 4180900, 4185593, 4975605, 4187541, 5818242, 4901344, 4184502, 4900147, 4976123, 5818682, 4237796, 5394364, 4896497, 4886628, 4885026, 4974510, 4885025, 4981625, 5792611, 5811680, 5780438, 4912656, 4916211, 4909817, 4097794, 4192093, 4192092, 4191960, 4098468, 4190543, 4097954, 4919405, 5205234, 4392983, 4909818, 4911774, 5448798, 4194690, 4195220, 4919118, 4920226, 5411599, 4905889, 4194359, 4393346, 4913784, 4097650, 4913961, 4922276, 4189290, 4194935, 4097285, 4394369, 4916790, 4907811, 4910188, 4909450, 4911365, 4189753, 4904935, 4947943, 4097904, 4903499, 4911775, 4392503, 4098907, 5473608, 5472756, 4914365, 5441886, 4905733, 4913624, 4913623, 4191513, 5467290, 11515025, 4193567, 4904254, 4916196, 4195578, 4915361, 4188667, 4922754, 4191287, 5472804, 4194364, 4192129, 5468016, 4190177, 4394735, 4098045, 4393178, 5470432, 5467158, 4098455, 4378136, 4191962, 4913962, 5478574, 4913785, 4914612, 4394650, 5470431, 4392984, 5536561, 5507464, 4194835, 4194439, 4391702, 4919024, 5410582, 5460838, 4921234, 4190651, 4190632, 4916796, 5536112, 4922770, 5454335, 5447373, 4097595, 4919554, 4904162, 4906702, 4925077, 4929276, 5536580, 4919119, 4192525, 5100621, 5558187, 4105209, 4100153, 4106379, 4398980, 4938243, 4103980, 4100348, 4321407, 4939600, 4322483, 5002115, 4925514, 4939602, 4933388, 4934374, 5571451, 4106909, 4938522, 4396186, 4397655, 5556052, 4101425, 5125683, 4107900, 4143532, 4943034, 4931488, 4106657, 4930603, 4928171, 4104337, 4992425, 4929685, 4102858, 4100078, 4107905, 4106866, 4099215, 4929896, 4397168, 4941698, 4397167, 4099313, 5575973, 4934740, 4102290, 4940158, 4100347, 5100498, 5098326, 5109411, 4099217, 4923653, 5106102, 4101228, 4936439, 5539185, 4926948, 4928161, 4939601, 4104721, 4104846, 4938245, 4931099, 4106911, 5090761, 4396083, 4941924, 5090825, 4107395, 4104974, 5090759, 5124439, 4937690, 5576438, 5107910, 4397654, 4100152, 4943607, 5579197, 4107763, 4105008, 5002114, 4100167, 4328640, 4104457, 4938244, 5538679, 5569563, 4104498, 4935627, 4106925, 5101285, 4943605, 4933135, 4936538, 4922776, 5100420, 5539179, 4259217, 5068267, 4112366, 5027121, 4404939, 4112100, 5027584, 4117261, 5009427, 4404700, 4556020, 4552464, 4111577, 4115598, 4550872, 4621418, 4343318, 5072717, 4557030, 4538724, 5071699, 4562098, 4653876, 4558306, 5042825, 4551257, 5067511, 4627050, 4112569, 4627420, 4113052, 4108980, 4117705, 4646510, 4554123, 4113531, 5058728, 4118203, 4556021, 4568356, 4624443, 4115602, 4555654, 5031712, 4623522, 4566958, 5036880, 4401696, 4552431, 4113891, 4551861, 4552131, 4110641, 5077919, 5071698, 4560728, 5039779, 4110088, 4342116, 4561828, 4555651, 4569549, 4554243, 4678681, 5060669, 4125162, 5067346, 4561258, 4627093, 4116776, 5058731, 4561559, 4629980, 4506615, 4564764, 4627811, 5059434, 4115814, 4551047, 4550552, 4552130, 5015048, 5067322, 4112185, 4112183, 4623950, 4113076, 4117813, 5051622, 4338548, 4550352, 5052446, 4116227, 4116600, 4258564, 4556543, 4553818, 4624222, 4621449, 4623288, 4555826, 4551010, 4111579, 4566727, 4117605, 4120641, 4148614, 4354007, 4120491, 4584411, 4578167, 4200476, 4567104, 4150000, 4589810, 4586085, 4712485, 4118646, 4706914, 4118816, 4758424, 4684293, 4202265, 4688037, 4688038, 4578640, 4748254, 4688040, 4121848, 4584707, 4516522, 4145234, 4758487, 4127641, 4122108, 4763409, 4564720, 4150435, 4148469, 4123775, 4118558, 4121073, 4126917, 4124043, 4405284, 4356453, 4753236, 4707989, 4583184, 4584640, 4571680, 4126560, 4570291, 4741512, 4127370, 4573122, 4122998, 4586043, 4733248, 4124253, 4689832, 4706912, 4573205, 4200875, 4564983, 4577015, 4748311, 4119946, 4684283, 4512003, 4631743, 4776883, 4681896, 4748315, 4571186, 4685619, 4125793, 4755182, 4579507, 4573417, 4586044, 4699959, 4122739, 4513355, 4123619, 4684295, 4582051, 4695709, 4740983, 4707988, 4569673, 4685631, 4571196, 4123586, 4347382, 4123863, 4751740, 4119373, 4569674, 4593192, 4126507, 4127340, 4630706, 4200477, 4201225, 4739191, 4575166, 4688631, 4716429, 4122006, 4568221, 4357135, 4119747, 4128579, 4758573, 4197069, 4122561, 4591000, 4577839, 4121256, 4581080, 4199744, 4700326, 4716523, 4758698, 4692674, 4583183, 4124271, 4129310, 4593555, 4215536, 4811017, 4130309, 4207965, 4441240, 4588285, 4441827, 4140344, 4154124, 4425916, 4808929, 4811789, 4832250, 4407133, 4528982, 4521468, 4412419, 4596046, 4139630, 4448416, 4608355, 4143007, 4594580, 4606370, 4139447, 4609545, 4606109, 4820642, 4831660, 4369891, 4609544, 4589352, 4521467, 4609380, 4587069, 4412826, 4610196, 4817159, 4817160, 4133645, 4140005, 4611579, 4139622, 4130307, 4374156, 4813140, 4134406, 4843577, 4131170, 4599385, 4140421, 4137638, 4604408, 4594525, 4138297, 4592898, 4136735, 4138905, 4599766, 4129028, 4131353, 4135488, 4138107, 4134700, 4202683, 4606594, 4594581, 4825114, 4130658, 4818172, 4153372, 4367916, 4139406, 4852505, 4604627, 4610392, 4610393, 4547724, 4136143, 4357042, 4853118, 4593554, 4152590, 4606810, 4143012, 4600765, 4425917, 4602899, 4424007, 4604874, 4414996, 4135612, 4422691, 4154716, 4134404, 4545269, 4137115, 4138248, 4853767, 4594582, 4135502, 4136980, 4604977, 4441241, 4415356, 4148936, 4378132, 4434974, 4140992, 4139587, 4599149, 4595181, 4597119, 4816852, 4813139, 4421738, 4594690, 4148660, 4590668, 4153093, 4598852, 4132359, 4137322, 4416717, 4832185, 4430473, 4597093, 4215352, 4131217, 4813131, 4590669, 4133735, 4430106, 4832189, 4140268, 4136162, 1105174, 1329, 46963, 1092284, 46503, 1089400, 1101843, 125133, 1205281, 1089399, 1092283, 1099449, 241306, 163386, 1104047, 1106277, 1056801, 50828, 241307, 52107, 47081, 164128, 49736, 49734, 1080405, 1088963, 1100188, 53553, 1107172, 1092585, 808359, 51141, 1095121, 1092420, 51964, 1090337, 1101847, 1125586, 1101844, 1113762, 236806, 1106817, 168120, 48894, 1113783, 46951, 1095122, 1107173, 168121, 48053, 1140671, 1097295, 53330, 46959, 53659, 48054, 1095453, 1104059, 46511, 1093947, 236810, 1106278, 1093503, 1137255, 1128571, 1092419, 1090222, 1054609, 1203713, 808445, 1092285, 804952, 1097046, 47949, 163625, 1095132, 1102398, 1112949, 1174906, 236209, 1123173, 1140623, 238716, 1193329, 1091532, 1055858, 46384, 172181, 1125699, 1097921, 1088916, 53428, 48000, 1099263, 1098726, 170521, 1089885, 1095114, 1205270, 1158187, 49746, 1182489, 1101066, 51188, 805264, 1097035, 1095794, 51285, 1140690, 1137256, 1095133, 166586, 51172, 1054222, 49745, 239346, 1148776, 1166982, 1092282, 1107174, 803910, 1131575, 1139193, 1107179, 1203699, 51380, 168493, 1106350, 1104063, 236805, 1096704, 1095117, 1104060, 1091856, 1173830, 123815, 805623, 237234, 810719, 1093506, 1104072, 1102987, 237804, 806018, 1078803, 52767, 1148741, 1172753, 48162, 1104411, 1107171, 53548, 1092411, 1120220, 1169086, 1089401, 48832, 49737, 1205299, 236804, 1111463, 165796, 1117912, 1098808, 47417, 1111465, 1119589, 1119588, 51386, 234488, 47421, 1078711, 1102976, 1117460, 46071, 53351, 1089875, 53654, 1111464, 53429, 1137786, 46049, 51664, 51331, 52007, 1097747, 53394, 1106281, 1095116, 1089884, 1098721, 1101842, 1148732, 786204, 773499, 55647, 769630, 787050, 793552, 177866, 985152, 764916, 766907, 782310, 947448, 786807, 781336, 54740, 11859, 773500, 780407, 3259, 770551, 55567, 55288, 59277, 773493, 937570, 59850, 816411, 956557, 953529, 1064434, 958261, 792477, 73797, 73792, 773498, 795498, 59234, 1252882, 971031, 62992, 1276742, 1106810, 7342, 1252821, 1276696, 73849, 770226, 8187, 776348, 793956, 974569, 773506, 57451, 5066, 54634, 776751, 1256483, 773507, 73637, 60623, 782651, 820882, 814956, 937890, 63748, 7585, 58198, 773736, 62161, 55586, 59855, 56650, 73744, 8188, 4361, 779561, 63744, 54579, 779926, 765184, 786772, 131139, 6161, 60566, 58199, 820228, 55838, 829223, 1252886, 62998, 56591, 766905, 10874, 61439, 59882, 779497, 788855, 177474, 971011, 54725, 60633, 793562, 786429, 780406, 823433, 73694, 62991, 1000228, 54631, 54550, 5342, 56589, 59039, 946886, 55648, 55877, 769721, 178191, 1108832, 55890, 955495, 1247716, 58269, 765528, 773735, 824468, 773497, 1106289, 1267035, 55650, 1260401, 764913, 779705, 776350, 938189, 769722, 764935, 793680, 1268490, 127945, 1107612, 974610, 773494, 57334, 54578, 60622, 776441, 56576, 59013, 985049, 9919, 56648, 937882, 1268656, 773495, 946507, 1069570, 58143, 1083313, 74457, 776351, 54619, 1086117, 770224, 1276739, 55891, 55761, 177873, 57340, 765824, 829222, 786428, 1252881, 989091, 950094, 1252864, 55646, 17642108, 764929, 1260337, 990784, 764914, 946844, 1248676, 938185, 1086428, 779871, 178190, 813604, 60565, 813809, 1068646, 765822, 765515, 791442, 769891, 985045, 55583, 59189, 789186, 769864, 821577, 57507, 180938, 816410, 821986, 789436, 58258, 55715, 54611, 773486, 765518, 766901, 73796, 768533, 11760, 787788, 62111, 323118, 871702, 329940, 589308, 338105, 406299, 329949, 318577, 851821, 321147, 326347, 838992, 335940, 328109, 318900, 837099, 338109, 322794, 334336, 14982, 319143, 319271, 337916, 577889, 68285, 324570, 141471, 16806, 901996, 137922, 65508, 336116, 408522, 319876, 14049, 319140, 860894, 892352, 192405, 329948, 319355, 122450, 321067, 338110, 67338, 319356, 320109, 324863, 831655, 402406, 67348, 835924, 589019, 71495, 320478, 406561, 576887, 70585, 321502, 330353, 342360, 326847, 326642, 137921, 73903, 71492, 323326, 330302, 73949, 870369, 201203, 830372, 320956, 320957, 72290, 73014, 263816, 893875, 328108, 884446, 319724, 74730, 68331, 64803, 884449, 876327, 199128, 909566, 72239, 589314, 846510, 189724, 199129, 299896, 66425, 876324, 406098, 68228, 64653, 319141, 836131, 870376, 69058, 322634, 318894, 895764, 921082, 576296, 332100, 71604, 324566, 869668, 332099, 861533, 69828, 322635, 68383, 411434, 71605, 11144, 64807, 322793, 922420, 19514, 319871, 318710, 843178, 320930, 321076, 330354, 95728, 576328, 911218, 63673, 72237, 72192, 901134, 882110, 406875, 326215, 329786, 842986, 843177, 831965, 843794, 321823, 64746, 72953, 263815, 861375, 577226, 69126, 324571, 71397, 68272, 922326, 22305, 21973, 589349, 69191, 69192, 320477, 318825, 326339, 407377, 320955, 865585, 843871, 72240, 326333, 326635, 68044, 326337, 145265, 405408, 68225, 412099, 320476, 923067, 401690, 843186, 330307, 190970, 301797, 318715, 14602, 73062, 405998, 336135, 331107, 95729, 140029, 326332, 335903, 66471, 334337, 922328, 65606, 70639, 843188, 320111, 208439, 351394, 273143, 74607, 101843, 680588, 698606, 25384, 210379, 655282, 215095, 361165, 363229, 359091, 686545, 98128, 31847, 348260, 350932, 340945, 80528, 686922, 209710, 362204, 678048, 363232, 340331, 348251, 340325, 340330, 620911, 340326, 340327, 340332, 361512, 80526, 78297, 205566, 33875, 727903, 339993, 79080, 80525, 638249, 349379, 719418, 340328, 105970, 81927, 78446, 152663, 80682, 78382, 81412, 727887, 352788, 79761, 340335, 355663, 80633, 361156, 638545, 647304, 619829, 75438, 76748, 347112, 348553, 81986, 211983, 82086, 344157, 361155, 346252, 337144, 346629, 344156, 339084, 619839, 659728, 728739, 646248, 355879, 340949, 23495, 361485, 336453, 671684, 344125, 75392, 357764, 620898, 76223, 417248, 737474, 642973, 148936, 637427, 82735, 347294, 215096, 74660, 346628, 355881, 74662, 692548, 340006, 25830, 351401, 272207, 638577, 698650, 81361, 98127, 340329, 670607, 76798, 683224, 709092, 355071, 75439, 76750, 647213, 76797, 80579, 305379, 79709, 712981, 365123, 343743, 351232, 306577, 340005, 346430, 77367, 209263, 619817, 365289, 363236, 645948, 76166, 634316, 344914, 356916, 344898, 346153, 354746, 342962, 346161, 77413, 580623, 81925, 31123, 367509, 28810, 638627, 76794, 340334, 639229, 76842, 339995, 656778, 350346, 340492, 28102, 620902, 647254, 80632, 647262, 365125, 619842, 100624, 709173, 336450, 624031, 361152, 367510, 75397, 340319, 74014, 362197, 309095, 78260, 75391, 343678, 652005, 666189, 346257, 74569, 76857, 82680, 678886, 77993, 476300, 508127, 373841, 383033, 368364, 92668, 83475, 38178, 367501, 369646, 428710, 85937, 444024, 444025, 85162, 448844, 111119, 84147, 387542, 481481, 386125, 376390, 90761, 381092, 385100, 381093, 84260, 36865, 381094, 435016, 375865, 369472, 91023, 382878, 475542, 466329, 368368, 86666, 376572, 449951, 391321, 84148, 369647, 85052, 373852, 380765, 581782, 373560, 220536, 376054, 313353, 86727, 391657, 376043, 381127, 160266, 376042, 391342, 310511, 38180, 91027, 223950, 224831, 517882, 293141, 759664, 219269, 534865, 92623, 518245, 375849, 389121, 574523, 519355, 428086, 372290, 372063, 90214, 385458, 385457, 93180, 105777, 430774, 36422, 503151, 90964, 90965, 387170, 85053, 85872, 375793, 378164, 380768, 227618, 374176, 365391, 758131, 363773, 490852, 380492, 226735, 393356, 369470, 84900, 92624, 376894, 219802, 435953, 445714, 490805, 386118, 384241, 380773, 227340, 439317, 90270, 86003, 466336, 380434, 497671, 436200, 90217, 444661, 369471, 761334, 519269, 445053, 370586, 86158, 84901, 288486, 32776, 761001, 86051, 427406, 38177, 467943, 38176, 38175, 449946, 435882, 374194, 375858, 431635, 380427, 466335, 519476, 485782, 221812, 368354, 112475, 37142, 374178, 90859, 758493, 84951, 372593, 389346, 427400, 90280, 466138, 383195, 312708, 378317, 384858, 91836, 759879, 519357, 90768, 427401, 508019, 87965, 105656, 434682, 375088, 365920, 435014, 371421, 384251, 219267, 758132, 372609, 89390, 760861, 374177, 378754, 375881, 365665, 32949, 369653, 107908, 104761, 38179, 526820, 760698, 215338, 112265, 387171, 376875, 376036, 421317, 368370, 384248, 86677, 91901, 7000260, 6159384, 6987291, 7406654, 6999349, 6107409, 7351282, 7004560, 6105336, 6108449, 7377911, 6986027, 7000250, 6254456, 6985702, 6992677, 7000243, 7427409, 6991353, 6986827, 7349932, 6107164, 7387335, 7409383, 6107548, 7400475, 7372069, 6101847, 7191754, 6986185, 7436160, 7351447, 7004559, 7350036, 6107154, 7400477, 7350046, 6107722, 7436691, 7436690, 6159865, 6928104, 7435677, 6153164, 7377854, 6773725, 7370603, 6770952, 6107406, 6104083, 6254602, 6770731, 6103299, 6106739, 7002250, 6775752, 6777233, 7372965, 6102231, 6102235, 6892984, 6259981, 7437865, 7406657, 7430504, 6988713, 6101631, 6985881, 7002706, 7000029, 6107592, 6101791, 6105436, 6104172, 6103906, 7373798, 7380201, 7429342, 7370606, 7192170, 6106098, 7351906, 6101337, 7372061, 7419011, 7358942, 6104255, 7400476, 7412801, 6765920, 6777070, 6444241, 6998597, 6996784, 7410745, 7437749, 7006539, 6773727, 6771653, 7387018, 7431552, 7000282, 6986948, 6996635, 6107159, 6448650, 7352844, 7437747, 7411761, 7387340, 6989437, 6106828, 6997743, 6997738, 6108392, 7370526, 7362427, 6105520, 6153233, 7435720, 7364321, 6102878, 7353128, 7436817, 7436639, 7381123, 6107725, 6993163, 7407516, 7350203, 6101353, 6107587, 7212475, 6102677, 6102487, 7437781, 6444445, 6988304, 6247521, 6104295, 6989434, 6996779, 7370651, 6103450, 7362377, 6991941, 6997516, 6986940, 7440855, 6773613, 6105118, 7398008, 6775094, 6109149, 6102284, 6776300, 6106740, 6106110, 6160172, 7406656, 6103440, 7370643, 6102458, 7420656, 6106735, 7350204, 6101843, 6102637, 7427334, 7410746, 7000631, 7352836, 6985681, 6989432, 6101459, 6105342, 7447618, 6968705, 6106736, 6992927, 7419830, 7351286, 7427625, 6102180, 6986552, 7001230, 7000628, 6107452, 6992916, 7427301, 7000292, 7407477, 7357339, 7427085, 6104131, 7004292, 7002707, 7436164, 7396657, 7420722, 6775742, 6106099, 7352850, 6101676, 6107768, 7000239, 6105519, 6997522, 6103997, 7363435, 6993706, 7388541, 6103209, 6252466, 7353752, 7000264, 7402234, 7363436, 7398005, 6109147, 6107853, 6989436, 6766360, 6444678, 6243714, 6105569, 6998304, 6156074, 6101535, 6108901, 6255844, 7001056, 6995221, 6102739, 7002739, 7352840, 6101677, 7000296, 6109150, 6103255, 6777263, 6986945, 6989438, 6153260, 6782146, 7010161, 6109741, 6782486, 7030902, 6790985, 6116728, 6782480, 6112526, 6109047, 7451775, 7202950, 7004995, 7014349, 7019703, 7005682, 6794709, 6116951, 6113352, 7017177, 6118579, 6109990, 7294546, 6118631, 7024807, 6119130, 6118758, 7023742, 6263396, 7026343, 6788226, 6786688, 7011250, 7027988, 6110954, 6793182, 6109118, 6109798, 6794796, 6944548, 6110089, 7006708, 6794821, 6785336, 6781657, 7024383, 6115246, 6450001, 7025719, 6110965, 6796618, 7025784, 6783231, 7469623, 6113483, 6118524, 7015141, 7005474, 6113192, 6114900, 6112538, 6113353, 6780025, 6118717, 6788167, 6790048, 7471334, 7452872, 6110956, 7005678, 7006558, 6109102, 6110953, 7204788, 7270721, 7026401, 6113485, 6118757, 6786536, 6115247, 6112602, 6788319, 7262517, 6786497, 7008711, 6971716, 6118715, 7023308, 6116097, 7310012, 6118666, 7004999, 7438385, 6788323, 7229228, 6257941, 7286585, 6116950, 7247635, 6783232, 6264825, 7299000, 7346529, 6109991, 7447225, 6109938, 7294222, 6780021, 6116952, 6458250, 6789205, 6784854, 6109059, 6778548, 6938259, 6114325, 7235853, 6115159, 7026400, 6165750, 6109801, 6793183, 7005681, 7451778, 7240596, 7227770, 6110093, 6256422, 7019706, 7014353, 6940466, 6786543, 6784850, 6794746, 7346530, 6112411, 6110819, 6113428, 6451257, 6781390, 6110957, 7239110, 6788133, 6789959, 7240598, 6972401, 6117684, 6113313, 6781586, 7025777, 7247587, 6778552, 6112444, 6784849, 6788244, 7028181, 7285309, 7026817, 6266576, 6112324, 6788318, 6109989, 6110818, 7299001, 6792379, 7007473, 6116084, 6794767, 6110863, 6118384, 7026344, 7007474, 6110090, 6794406, 6114279, 7007666, 6111670, 6263139, 6789793, 7025721, 7029276, 6112329, 6111672, 7305144, 7018336, 7447218, 7001242, 6110088, 6111671, 6110955, 7014392, 6112386, 7011249, 6118759, 6780105, 7009309, 7029275, 6117726, 6970272, 7009311, 7010164, 6116855, 6779890, 6113821, 6786493, 7005293, 6794774, 6453240, 6788185, 7031112, 7007668, 6113021, 6797571, 7469210, 7018438, 7235851, 6751736, 7025778, 7022214, 7259838, 7003383, 7025776, 7271091, 6117681, 7239116, 6108895, 6118716, 6165428, 6799067, 7247638, 6781588, 6946847, 6794832, 7024580, 7006544, 7010167, 6114321, 6794831, 6112564, 7447203, 6114776, 6955542, 6805625, 7076116, 6124473, 6124474, 7056949, 7050702, 6124671, 7046990, 7042799, 6752712, 7045659, 7068113, 7033789, 7035950, 7050717, 7057858, 6177981, 6280052, 7050705, 7042111, 7055871, 7045433, 6752720, 6123837, 6802408, 7033782, 6122483, 6815252, 7069070, 6214234, 6174865, 7046543, 6814568, 6814605, 6123675, 6761121, 7092002, 7061773, 6805588, 7060245, 6121181, 6128673, 6805647, 6121605, 7119300, 6124760, 6123021, 6803871, 6749604, 7045440, 7069074, 6816599, 6123810, 6751182, 6800539, 7073415, 7042486, 6176357, 6459262, 7060912, 6797661, 6807439, 6804108, 6125678, 7108070, 6765542, 6804109, 7118065, 6895626, 6123363, 6181113, 6803977, 6806155, 7035299, 6128596, 6802327, 6122937, 6123808, 6122770, 7044881, 6816598, 6800507, 6119443, 6119459, 6283982, 6953986, 6814569, 6329613, 6819029, 6755255, 6123718, 6749634, 6797663, 6802260, 6802362, 6279224, 7040158, 6124759, 6123836, 6819034, 6179975, 6119512, 7037524, 7046702, 7082112, 7044880, 6122100, 7035953, 6122099, 6127360, 6282381, 6811645, 6122890, 6750403, 7060247, 7149870, 7053291, 7040956, 6126423, 7040955, 6805791, 7069076, 6127172, 7043008, 6122098, 7042110, 7074794, 6123884, 6125728, 6814669, 6956376, 6123760, 6816343, 7053334, 6120350, 7053889, 6800440, 6123079, 6122939, 6121965, 6275811, 6128447, 7056952, 7044913, 6120738, 7125768, 7038483, 6807469, 6755247, 6754131, 6799081, 6979980, 6174864, 6805621, 7115012, 7059257, 6119510, 7073151, 7047788, 7119301, 6812820, 7036816, 6122003, 7060255, 6122057, 6124708, 7044883, 6803890, 6810839, 6120389, 6800465, 6127137, 6178915, 7065559, 7076005, 6281143, 6125727, 6982085, 7040528, 6462116, 6800869, 6122848, 6122767, 7038491, 6803911, 7038244, 6122970, 6799077, 6128449, 7040950, 7065555, 6800536, 6809108, 7054041, 7129028, 6120966, 6802095, 6214228, 6215008, 6128086, 6211296, 6805841, 7141388, 6126752, 6119492, 6177991, 7053286, 7042113, 6807679, 6125811, 6804113, 6981577, 7067965, 6805715, 6122892, 6799558, 6750396, 6177374, 6291439, 6125636, 6809161, 6128451, 6286033, 6123647, 7149895, 7046680, 6802331, 6808048, 7052009, 6121090, 6802391, 6765541, 6811068, 7074777, 7092487, 6279230, 6179976, 6120311, 7149897, 7141389, 7053283, 6805618, 7032378, 7130553, 6807435, 6796289, 6814610, 6123821, 6802413, 6811037, 7076989, 7074752, 6981575, 7074750, 6796288, 6952022, 7092423, 6810736, 6210726, 7119271, 7044606, 6751183, 7038165, 7049029, 7074797, 6805714, 6129179, 7103635, 6125676, 6752708, 7035298, 6807568, 7050714, 6765526, 7038182, 7044608, 6127542, 7141392, 6122004, 7040157, 6124805, 7047770, 7121527, 7065831, 7050711, 6580491, 6184493, 6343175, 6352272, 6337083, 6347578, 6355247, 6828092, 6347108, 6847336, 6352448, 6861819, 6341159, 6341015, 6409446, 6366133, 6341843, 6349715, 6343656, 6134093, 6133645, 6604081, 6873608, 6343861, 6359995, 6307464, 6223819, 6621623, 6366125, 6403101, 6321674, 6886255, 6843620, 6343860, 6401351, 6601670, 6133637, 6138377, 6366141, 6135830, 6219156, 6402096, 6347109, 6359996, 6300213, 6349719, 6336819, 6411944, 6355542, 6343871, 6679229, 6580492, 6135725, 6633618, 6133104, 6402612, 6416514, 6633598, 6336641, 6837786, 6129380, 6336708, 6403172, 6358329, 6352267, 6411270, 6134039, 6338383, 6338381, 6416513, 6407606, 6343868, 6339595, 6838068, 6872170, 6821904, 6401230, 6315259, 6354826, 6416399, 6549746, 6134959, 6138116, 6130246, 6401257, 6575836, 6576814, 6303624, 6340575, 6130195, 6416510, 6404471, 6416401, 6571946, 6360688, 6872169, 6409208, 6416575, 6401458, 6132179, 6196006, 6337741, 6403142, 6134136, 6134884, 6129459, 6412934, 6361553, 6132218, 6129364, 6344715, 6136745, 6600417, 6606576, 6305758, 6185553, 6404385, 6139671, 6401193, 6871470, 6134885, 6293939, 6621660, 6825993, 6407644, 6401551, 6870483, 6299612, 6402199, 6133166, 6355851, 6133072, 6354828, 6352386, 6227361, 6131148, 6136690, 6401668, 6130243, 6358552, 6140494, 6679239, 6404382, 6404404, 6618436, 6401837, 6848406, 6407598, 6826111, 6412896, 6404344, 6412795, 6321673, 6133059, 6350409, 6826108, 6352208, 6304360, 6131178, 6304513, 6823253, 6132852, 6336794, 6338856, 6357964, 6404440, 6337580, 6139668, 6339349, 6363078, 6832707, 6130330, 6131914, 6343041, 6824247, 6133987, 6861303, 6139669, 6354030, 6134048, 6135828, 6828120, 6403140, 6414387, 6134180, 6337323, 6848932, 6341844, 6337581, 6132082, 6129077, 6404341, 6366124, 6416511, 6336824, 6129914, 6130752, 6342564, 6341435, 6632131, 6337742, 6406886, 6401172, 6187790, 6403171, 6423045, 6366135, 6833677, 6294517, 6404377, 6130329, 6341842, 6402201, 6305244, 6401229, 6345330, 6137694, 6859300, 6407642, 6601463, 6405876, 6337322, 6861770, 6345023, 6353233, 6418109, 6639234, 6337198, 6406198, 6639224, 6130197, 6348314, 6360689, 6347119, 6854869, 6134565, 6409316, 6135489, 6345281, 6132178, 6347117, 6343089, 6343462, 6347110, 6402091, 6350411, 6401442, 6130335, 6342562, 6352209, 6341847, 6349453, 6600418, 6416707, 6832623, 6139529, 6131164, 6339939, 6217921, 6354122, 6407641, 6138593, 6134981, 6405881, 6301320, 6130244, 6138642, 6336711, 6336616, 6134137, 6405877, 6633596, 6833676, 6412833, 6136841, 6136794, 6404472, 6352081, 6138597, 6366132, 6407714, 6135989, 6401510, 6625781, 6293321, 6847332, 6339312, 6407638, 6354904, 6366137, 6686849, 6129368, 6133153, 6338082, 6134979, 6350097, 6352967, 6366138, 6337285, 6140541, 6132220, 6186329, 6350408, 6411208, 6619453, 6848810, 6341642, 6341640, 6639290, 6305762, 6409272, 6341632, 6139628, 6199470, 6341017, 6825998, 6402540, 6851041, 6342563, 6401550, 6343878, 6139139, 6129458, 6403151, 6402168, 6337196, 6132229, 6405939, 6138117, 6188770, 6855871, 6848905, 6138009, 6872168, 6615094, 6352080, 6131223, 6337075, 6337074, 6342565, 6338385, 6689424, 6347581, 6412929, 6339923, 6139100, 6136793, 6337578, 6130751, 6617676, 6321684, 6133103, 6405914, 6222257, 6347118, 6832557, 6139572, 6363079, 6355533, 6138551, 6358886, 6368355, 6419809, 6391877, 6363731, 6747142, 6470361, 6366150, 6717541, 6328298, 6206207, 6387059, 6723011, 6321686, 6608419, 6332178, 6201211, 6143195, 6373547, 6472365, 6379122, 6704545, 6381770, 6383654, 6376722, 6374059, 6332274, 6141436, 6376596, 6148470, 6421391, 6143042, 6365017, 6142202, 6439836, 6363930, 6426616, 6383836, 6388452, 6379459, 6379455, 6143965, 6546371, 6689662, 6381570, 6368649, 6715723, 6428687, 6363735, 6145024, 6089632, 6428371, 6320945, 6142775, 6145480, 6432119, 6418295, 6143083, 6143969, 6385797, 6466017, 6429229, 6235889, 6375889, 6389142, 6365015, 6363727, 6089633, 6147703, 6714795, 6087702, 6092554, 6366561, 6148621, 6382004, 6208231, 6427609, 6428555, 6712366, 6231897, 6428372, 6691783, 6491703, 6327927, 6434024, 6437310, 6360413, 6324710, 6428655, 6421438, 6421344, 6360815, 6439835, 6437579, 6706302, 6488496, 6144625, 6322906, 6428513, 6539812, 6479685, 6745850, 6693665, 6439298, 6481927, 6381006, 6389273, 6230296, 6146861, 6142208, 6150275, 6366560, 6143188, 6363219, 6388898, 6209324, 6437365, 6230174, 6437487, 6489694, 6434122, 6367691, 6423186, 6428553, 6146875, 6423133, 6703818, 6539363, 6088819, 6141335, 6712032, 6427416, 6437366, 6367689, 6377071, 6699312, 6088192, 6419896, 6361561, 6388521, 6715724, 6229314, 6147293, 6387207, 6148991, 6365352, 6389060, 6421918, 6489697, 6363935, 6377070, 6390207, 6202993, 6595350, 6438504, 6421395, 6689676, 6142748, 6714792, 6146752, 6361565, 6360414, 6378423, 6375760, 6140906, 6322710, 6361287, 6418300, 6428577, 6712393, 6329547, 6334234, 6423162, 6434367, 6140202, 6365007, 6383655, 6437815, 6378733, 6230173, 6706305, 6379120, 6389648, 6333298, 6376016, 6497566, 6233921, 6235453, 6209513, 6145932, 6147454, 6478567, 6150050, 6421424, 6378117, 6366276, 6362597, 6206105, 6146045, 6144975, 6142254, 6419934, 6321689, 6087753, 6385894, 6439286, 6142218, 6142348, 6363216, 6150370, 6607784, 6386936, 6142752, 6149992, 6434117, 6208968, 6140091, 6143138, 6437509, 6374058, 6364909, 6428290, 6707342, 6697460, 6371535, 6381007, 6608483, 6091840, 6141424, 6388523, 6387197, 6373050, 6439364, 6381775, 6143187, 6432200, 6383937, 6428582, 6202851, 6327926, 6387208, 6365349, 6365350, 6388897, 6141377, 6469079, 6424827, 6326632, 6386932, 6430142, 6086466, 6147551, 6140340, 6421359, 6469080, 6707391, 6375621, 6434084, 6148522, 6439360, 6147549, 6140355, 6418303, 6374055, 6700643, 6368889, 6426646, 6143868, 6419851, 6148617, 6580926, 6143981, 6713092, 6724511, 6366151, 6202437, 6146006, 6363740, 6365008, 6376291, 6150320, 6143964, 6698373, 6389647, 6749228, 6143039, 6143970, 6326932, 6150365, 6196251, 6150180, 6434058, 6146762, 6376292, 6428590, 6584561, 6374050, 6199481, 6512582, 6726981, 6147640, 6374057, 6690963, 6233398, 6092555, 6427057, 6146821, 6147693, 6712031, 6143968, 6144933, 6421429, 6691639, 6372563, 6705802, 6362905, 6706301, 6482920, 6364908, 6434081, 6732403, 6378118, 6581756, 6693039, 6712367, 6486249, 6390194, 6150276, 6512581, 6146378, 6145027, 6732409, 6238136, 6432205, 6466047, 6146033, 6092557, 6328297, 6357536, 6144348, 6151044, 6705172, 6428619, 6147691, 6726307, 6384785, 6381003, 6376718, 6699655, 6360415, 6691784, 6366148, 6140351, 6142252, 6712030, 6364997, 6388524, 6090280, 6145832, 6363220, 6385893, 6145483, 6432198, 6368877, 6419958, 6700072, 6424854, 6435772, 6363488, 6690375, 6378422, 6370492, 6595452, 6096517, 6432201, 6319244, 6365351, 6748177, 2866388, 3883168, 2862464, 3931806, 3883167, 2864634, 3889396, 2579257, 4040552, 3839263, 3899049, 2933492, 3905922, 3894474, 3890789, 3897345, 3904643, 3899843, 2861219, 3905561, 3859587, 2857103, 3971528, 3894200, 3897471, 4029895, 3882061, 3882812, 2415436, 3889230, 3903501, 3873614, 3885891, 4006661, 3926040, 2933119, 2989349, 4019949, 3921097, 3933617, 3881561, 3896085, 2857210, 3894481, 4019951, 2858187, 2863676, 2990631, 2859077, 3966746, 2857276, 4043661, 4040553, 3929874, 3926591, 4056843, 3902420, 3903504, 3974051, 4045047, 3884469, 2856949, 3899841, 2982694, 2859931, 3155548, 2861365, 2857910, 3904559, 3882509, 3919076, 3922535, 3155982, 2857025, 3965335, 2861218, 3888810, 4056250, 3880810, 3900301, 3924221, 2863674, 2863675, 4047104, 3874573, 2865677, 3903062, 3924285, 3900303, 3881496, 3924984, 3882371, 3935203, 2931153, 3977557, 2863679, 4007415, 2865930, 3921583, 2991478, 3884746, 2578421, 4006126, 3901956, 3910523, 3931773, 2865417, 3894587, 3925335, 3874932, 4003987, 3881497, 3892293, 2865614, 3894707, 3921142, 2860494, 2860441, 3891346, 4018499, 2578610, 2860453, 3902590, 3926169, 3933645, 3932509, 2857900, 3921146, 3921173, 4006145, 2981292, 2862371, 3932499, 3838343, 3888312, 3933677, 3881068, 3881608, 3933736, 4005428, 3921279, 3918688, 3881323, 3910524, 2865502, 2860386, 3929943, 2862370, 2857803, 4054701, 2860284, 3896964, 3935205, 3881501, 3880521, 2866047, 3883162, 3894143, 3849459, 2985675, 3922471, 4029558, 4054521, 3926141, 4075944, 3918684, 4043663, 3924719, 2861526, 3883182, 2410320, 2413097, 3917968, 2861220, 2862419, 3926074, 2866291, 3919803, 2856908, 3882526, 2981088, 2991475, 3933397, 2857265, 4018502, 3882270, 3880714, 2861527, 3884690, 3965336, 3929876, 3883172, 2866960, 3883868, 2863443, 3894199, 3891497, 3973309, 3970647, 3977198, 3838188, 3964713, 3926067, 2864032, 2412829, 3880715, 3996846, 3965343, 3885813, 3933739, 4076202, 3894971, 3966742, 2859412, 3968773, 3919831, 4038511, 4040550, 3880867, 3890786, 3903063, 3924286, 3886943, 4056240, 2864027, 2857361, 3893343, 3977491, 3881331, 4056259, 3874009, 2863596, 2993147, 3893793, 4074031, 3902588, 3899840, 3990734, 3924216, 3884688, 4008815, 2578493, 2988687, 3906048, 3928422, 3900727, 3922505, 2863492, 4029717, 3918652, 2579868, 3926135, 3896179, 3880670, 3886743, 3906049, 4073304, 3935242, 4054135, 3886191, 3999311, 3889100, 2996991, 3871597, 2862933, 3891264, 3899046, 3917752, 3917702, 3839262, 3885889, 3920301, 3888133, 2861311, 4046137, 2996690, 2861315, 3917316, 2863541, 3922510, 2862315, 4063010, 3929906, 4054138, 3933675, 3880557, 2986508, 2867396, 3873389, 2857949, 3966851, 4009215, 3897470, 2866336, 3891348, 3989142, 3936866, 3883170, 2861523, 2999455, 2858710, 3924191, 4046122, 3838425, 2858664, 4067613, 4042800, 3917264, 3890657, 3921102, 2863754, 2410328, 2858619, 2866289, 2857677, 3894589, 2981982, 3926113, 3918691, 2858711, 3919804, 4038754, 4029186, 3887165, 2858707, 3926042, 2857901, 2865422, 3926848, 2857212, 2859421, 2865518, 3883918, 3996172, 4051686, 2857213, 3894482, 2866338, 3924720, 3918190, 3881309, 2857036, 3917751, 3928054, 3935204, 3964710, 3989147, 4056254, 4008807, 3884747, 4033706, 2861223, 4056256, 3891826, 3921727, 4067134, 4056246, 3985516, 2864530, 4067130, 4019953, 3927165, 4042306, 3883815, 2857739, 3918734, 3881327, 3919832, 3982468, 3887159, 4004503, 3974116, 3882269, 2858708, 2412521, 3973648, 3885033, 3921124, 2858743, 4043659, 3924197, 2865615, 3973293, 2862514, 3896811, 3924196, 3965341, 3899404, 4037555, 2860942, 3919805, 3931802, 3537787, 3094463, 3516257, 3517241, 3531560, 3298406, 3298404, 3510384, 3789208, 3081188, 2423337, 3783203, 3527086, 3516447, 2936956, 3517243, 3537784, 3531852, 3082733, 3511311, 3528402, 3531851, 2439568, 3298411, 3516704, 3023556, 3698783, 3526155, 3519886, 3514804, 3735627, 3510278, 3086204, 3517396, 2868173, 3940480, 3525337, 3009050, 3511376, 3771955, 3732901, 3510333, 3530897, 2856857, 3754572, 3461193, 3093556, 2422994, 3769163, 3528404, 3089465, 3511877, 3510393, 3729755, 3510285, 3522818, 3521523, 3511308, 3081081, 3711963, 3089361, 2878275, 3092712, 2871819, 2870956, 3792350, 3096181, 3956939, 3516097, 3528278, 3944373, 3511125, 3946979, 3521531, 3530769, 3092967, 3753116, 3946944, 3539618, 3099944, 3093305, 3485068, 3941214, 3456079, 3711553, 3514806, 3513684, 3081148, 3079781, 3773141, 3018566, 3524513, 2871294, 3958345, 3944976, 3525055, 3782679, 3522372, 3080511, 3512663, 3511127, 3018561, 3519879, 3719282, 3531862, 3081622, 2878274, 3084344, 3513867, 2870223, 3536677, 2871378, 2869208, 3086203, 3097099, 3023712, 2873396, 3007984, 3804028, 3022664, 2936958, 3081085, 3512121, 3526151, 3095410, 3081153, 3528541, 3085844, 3524744, 3941533, 3089521, 3094627, 2869745, 3098341, 3083995, 3005384, 2868162, 2874278, 3539708, 3081221, 3098194, 3081211, 2937825, 2878175, 2871285, 3512783, 3007683, 3087310, 2878859, 3092938, 3539714, 2876183, 3091164, 2875235, 2877327, 3742049, 3089408, 3946946, 3792347, 3949246, 3080056, 3940258, 3092972, 3089466, 2942407, 3092974, 3096429, 2946740, 3096460, 2871333, 3948357, 3510232, 2873863, 3510764, 2872516, 3520315, 3519416, 3794304, 3098340, 3956940, 3946953, 3519731, 3734264, 2937707, 2873440, 3793001, 3518980, 2875812, 3092934, 2877129, 3005138, 3517644, 3017804, 3804025, 3080510, 2875317, 3782654, 3534568, 2868030, 2870352, 3711547, 3732891, 3083913, 3709586, 3781322, 2424759, 3511821, 3944997, 3537092, 2872870, 2873397, 3542451, 3963953, 3080177, 3516606, 3512382, 3530865, 3011583, 2878131, 3539617, 3527712, 3080050, 2426419, 2868413, 2945109, 3080173, 3089490, 3521526, 3949238, 3510601, 3510602, 3794300, 2419384, 3950252, 2871418, 3525768, 3094804, 2431027, 2938849, 3022694, 3089491, 3089433, 3519225, 3533315, 3516096, 3531860, 3036936, 3080101, 3521522, 3535601, 2868254, 3767146, 3956521, 2869346, 2876326, 3092968, 3513009, 3782678, 3549312, 3525317, 3095501, 3089500, 2867260, 2421120, 3769719, 3521761, 2874411, 3537216, 3740675, 3783868, 3513012, 2878272, 2878174, 2428852, 2873161, 2868812, 3729666, 3943691, 2868207, 3729636, 3094717, 3021407, 3089407, 3790968, 3099943, 3521518, 2430180, 3091138, 2872514, 3517643, 2943780, 2868029, 3712681, 2868255, 3511382, 2876325, 3782653, 2878867, 3539037, 3080048, 3514727, 2872822, 3520319, 3099950, 3001519, 2943781, 2878130, 2419524, 3745702, 3521532, 3528853, 3086721, 3539741, 2417075, 3510282, 2869304, 3084975, 3535990, 3722633, 3003259, 2940091, 3087488, 3088219, 3094776, 3092976, 2423826, 3298409, 3514224, 3534570, 3511184, 3533699, 2876244, 3089411, 3081212, 3519885, 3094811, 3519348, 2870315, 3298408, 3537785, 3782677, 2874366, 2877644, 3087456, 3527853, 3018564, 2878173, 3089360, 3522371, 3081155, 3771951, 3095503, 3089439, 3525336, 3954551, 3758947, 3528403, 2872429, 3095641, 2870233, 3523240, 2868172, 2876744, 3009726, 2873324, 2876233, 3535752, 3510947, 3713748, 3948638, 3520317, 3734289, 2935737, 3520318, 3510088, 2878016, 2423333, 3773181, 3088085, 3950253, 3090127, 3083921, 2426590, 3097109, 3540097, 3771949, 2439577, 3958384, 2431021, 3511372, 3711534, 3092933, 3535714, 2868294, 3011180, 3094657, 2875321, 3298410, 3298405, 3080123, 3521427, 3729199, 3940501, 3087550, 3001523, 3517650, 3096431, 3521428, 3525168, 3525369, 2935222, 2428948, 3026552, 3516608, 3001520, 3779921, 3578568, 3631784, 2892047, 3625243, 3103778, 3113566, 3569765, 3543246, 3312658, 2890307, 3584744, 2967184, 3110248, 3552680, 3306374, 3316518, 3655305, 2885650, 2447998, 3294977, 3318723, 2885357, 3103580, 3624680, 3310941, 2877912, 2953770, 2439856, 3302705, 3320181, 2443619, 3546618, 3309199, 3320747, 2824707, 3560381, 3119786, 3555650, 3027267, 3669768, 2956516, 2960897, 3105913, 2948735, 2820329, 3115445, 3301614, 3108346, 3492950, 3106656, 2441633, 2888651, 3555386, 3307582, 3543236, 3806169, 3310792, 3330524, 3305794, 3310952, 3619219, 2433406, 3305795, 3318754, 2890021, 3298360, 3781194, 3309658, 2881132, 3318609, 3492164, 3543234, 3109670, 3119024, 3631319, 2955955, 3546751, 3295315, 3304706, 3311755, 3307584, 2967185, 2897305, 2887951, 3038552, 3820493, 3117554, 2890019, 3040923, 3325289, 3319629, 2882868, 2892130, 2878741, 3655856, 3032033, 3040918, 2442317, 3655855, 3029592, 3106448, 3310942, 3541724, 2824710, 3115236, 3451888, 2953318, 3300587, 3317057, 3295129, 3584749, 3322955, 2442323, 3106449, 3570029, 3443128, 3552076, 3115394, 3330526, 2967187, 3105986, 2882234, 3579683, 3553277, 2882420, 3309002, 3297907, 3675008, 3571773, 2897300, 3631792, 2957416, 3622203, 2822203, 3101790, 2890900, 3036291, 3300841, 2891992, 3101821, 2887723, 3546611, 3038254, 3546612, 3565967, 2820359, 2881131, 3120928, 2881130, 3827456, 3653128, 3540329, 3693764, 3548521, 3300457, 3038722, 3624669, 3105748, 2888942, 2882083, 2960473, 2880161, 2880214, 2434815, 2887726, 3546466, 3668124, 2887152, 2883488, 3450521, 3549990, 3555126, 3101865, 2882180, 3121055, 3115449, 2880069, 3538964, 2882288, 2446091, 3570035, 2885354, 3300375, 3119687, 2885355, 3552906, 3781184, 3107663, 3121334, 3552925, 2880218, 3028545, 2880114, 3119086, 3306380, 2886716, 3574424, 2888018, 3816835, 3549842, 3480374, 3105747, 3477659, 3103730, 2880885, 3033339, 3683478, 2882339, 3106450, 2821953, 2897303, 3297578, 3574383, 3314538, 3034372, 3556337, 3119785, 2890820, 3599344, 2967186, 2880294, 3597990, 3678756, 3491317, 3107660, 3118061, 3819804, 3117864, 3316514, 2882294, 2967183, 3579066, 3545965, 3120989, 3550162, 3675128, 3108064, 2950015, 3614304, 3109581, 3312371, 2890903, 3322728, 2890501, 3119689, 3313041, 3301517, 3542775, 3113957, 2882183, 2958400, 2824706, 3317054, 3543241, 2883406, 3553627, 3669757, 3296979, 2889416, 3541830, 2446132, 2882184, 2880213, 3632149, 3101839, 2885638, 3622543, 3632163, 3683485, 2879970, 3113764, 3791601, 3466563, 3104433, 3109602, 3592880, 2959719, 2438553, 2434546, 3584748, 3109661, 3572463, 2890808, 3122887, 2885572, 3819198, 2957943, 3579676, 3305185, 3556983, 2886820, 3024542, 3030904, 3330028, 3113538, 3548626, 3598162, 3295020, 3315932, 2883393, 3295548, 2824708, 3541824, 3113599, 3295019, 3119087, 3309198, 3543093, 2433409, 3317042, 3546465, 2885573, 3662274, 3555385, 3549420, 3655310, 3107487, 3624684, 3103764, 3579659, 3113576, 3298559, 3103761, 3653755, 3312372, 3571768, 3031226, 3295547, 3622539, 3296982, 3500688, 3294978, 3330031, 3032537, 3310925, 3540070, 2891032, 3546747, 2890550, 2880066, 3301985, 3538962, 2882736, 3101843, 3298379, 2445932, 3598611, 3691557, 3105681, 2887941, 2889081, 2885450, 2958105, 3675133, 2886110, 2885599, 3806894, 3547126, 3627208, 3033040, 2442610, 3315513, 3111615, 3549507, 3502542, 3820443, 3657877, 2439559, 2826311, 3549043, 3552697, 2825571, 3500684, 3101895, 3306385, 3315824, 3103731, 2888509, 2442322, 3114506, 3539691, 2882289, 2890805, 3585441, 3543239, 3109575, 3297940, 3499456, 2954800, 3549473, 3114434, 2439854, 20928962, 2879174, 3295018, 3297575, 3556338, 3689065, 3307670, 3543247, 3622197, 2889957, 2948466, 2887885, 2882082, 3680816, 3805550, 3821824, 3655306, 3315677, 3121335, 3632148, 2885505, 3552920, 3566457, 3675134, 3605397, 3806876, 3311904, 3583073, 3791604, 3330528, 3109574, 3495732, 3109663, 3107721, 3557014, 2820289, 3295553, 3315825, 3318751, 3653758, 3309196, 3558996, 3553279, 3571772, 3549843, 3103950, 3038987, 3584746, 3805547, 3573270, 3299089, 3632375, 3322826, 3436330, 3119340, 3586248, 3027153, 3552921, 3301986, 3031225, 3669756, 3827416, 3450522, 3297912, 3806896, 3558936, 2959716, 3109764, 2880718, 3552679, 3121022, 3101804, 3115421, 3114351, 2437417, 3311905, 3299095, 2437455, 3585444, 2823955, 2884455, 3552696, 2880017, 3552694, 3294976, 2821397, 3826424, 3305136, 2879101, 3101849, 2881038, 3119022, 3819810, 2953870, 3392363, 3260659, 2904394, 3278942, 3261651, 3288680, 3372910, 3289931, 3277450, 3275894, 3041924, 2893189, 3281637, 3128589, 3058228, 3234516, 3135880, 3282033, 3185621, 2455778, 3046383, 3048121, 2826714, 3047334, 3281014, 3142949, 3136831, 3057036, 3280316, 3049953, 3373260, 3137073, 2458438, 3411737, 2457657, 3280741, 2453619, 2898963, 3137061, 3417989, 3422091, 3046537, 2846791, 3047339, 3047335, 3286830, 2903322, 2963213, 3288682, 3358644, 3282478, 3282479, 3146369, 3276281, 3284974, 2459174, 3047336, 3422548, 2893930, 3065157, 3294081, 3131041, 3356852, 3276282, 3385910, 2967433, 3050522, 2448358, 3418376, 3065156, 2450113, 3277562, 3047337, 3410238, 2895829, 3122919, 3411756, 3173455, 2891797, 2833577, 3277558, 3178370, 2901609, 2832549, 3403868, 3356847, 2578012, 2904582, 3418730, 3282481, 3046553, 3123538, 3287167, 3276824, 3286041, 3044508, 3288029, 2451712, 2897515, 2973531, 3384075, 3192871, 3285215, 3045553, 3339175, 2899536, 2896139, 3224543, 3416147, 3063541, 2974463, 3139179, 3044515, 2897559, 3276279, 3276752, 2897388, 3339821, 3277569, 3286384, 3282034, 3137065, 2901610, 3143625, 3044506, 2837300, 2897556, 3126890, 2902491, 3280388, 2845011, 3190040, 3276360, 3371619, 3361022, 3042309, 3261612, 3166107, 2891886, 3046554, 3280158, 3289932, 3286383, 3056820, 3281620, 2837304, 3286039, 3128362, 2895840, 3415422, 3345774, 3286722, 3147048, 3289523, 2903110, 3294079, 3123540, 2965719, 3123539, 3134266, 2903876, 3385897, 3045264, 3136832, 2971565, 2899188, 3403863, 2967917, 2902494, 3192843, 3362176, 2962695, 2901569, 3294080, 3197978, 3136828, 2902114, 3336380, 3042398, 3278955, 2901611, 3139728, 2897987, 3135013, 3356357, 3178373, 2971708, 3367001, 2892988, 3263098, 2893187, 3124899, 3143436, 3042310, 2896828, 3246246, 2901565, 2900977, 2904053, 3131406, 3046542, 3200265, 3170993, 3139730, 3281724, 2899425, 3379743, 3135047, 2969930, 3286360, 3061819, 2838318, 3372915, 2900980, 3125915, 3044509, 3404638, 3044507, 3129065, 2963059, 3282463, 3124685, 2465180, 2893877, 2897986, 3044912, 3367452, 3128465, 2826285, 3403825, 3124680, 2901818, 3292711, 2457043, 3277579, 2968248, 3045545, 2462581, 3050179, 2898052, 3041937, 2900410, 3339790, 2896866, 3190503, 3042317, 2839632, 2972542, 3171001, 3192861, 3280157, 2970337, 3196080, 2895319, 3338379, 3131404, 2899102, 3277578, 3393023, 2902379, 3291657, 3165298, 3139326, 3042835, 3396164, 3146370, 3139138, 3204250, 3248129, 3409907, 3288683, 3069466, 2902319, 3203571, 3053887, 2849492, 3248127, 3142943, 3131040, 3164788, 3339788, 3290517, 2899719, 3129114, 2895993, 3289791, 3422105, 3053312, 3126956, 2451549, 3275750, 3282032, 3292363, 2899140, 3130927, 3142562, 3292710, 3422262, 3286831, 3278232, 3058088, 2901251, 2891940, 3045351, 3281016, 3284943, 3057041, 3048224, 3337484, 3276591, 3053888, 3229484, 3336177, 2448428, 3049213, 3137075, 3058555, 3350281, 3122967, 3147046, 3367455, 3134082, 3132275, 3350283, 2457710, 3169489, 2894547, 3044932, 3276280, 3282482, 2896868, 3169499, 2893865, 3276901, 3276900, 2893867, 3057924, 3042041, 3289433, 3275748, 3042826, 3136953, 2848604, 2899239, 2903955, 3142593, 3416079, 3288866, 2897517, 3184334, 3282572, 2892989, 3346979, 3383875, 3258578, 3054555, 3288871, 3056821, 3281001, 3292630, 3141491, 3057063, 2904568, 3143450, 2836565, 2895214, 3410217, 3170985, 3292634, 2842677, 3137469, 3058874, 2898636, 3284975, 3360270, 2897516, 3052668, 3053176, 3169044, 2899718, 3289861, 2899663, 3278754, 3282078, 2970555, 3184196, 3047333, 3125882, 2902486, 3049216, 2901566, 3139325, 2898622, 3199881, 2840172, 2894492, 3145064, 2844356, 2842680, 3136331, 2897005, 3144333, 3041936, 3289961, 3046854, 3132244, 2899773, 3257532, 3282480, 3343463, 2451713, 3276283, 3281619, 3279101, 3422261, 3122983, 2898696, 3130925, 3210286, 2904523, 3207375, 3270328, 3144330, 3162515, 3371882, 3141194, 3284501, 3289930, 3144366, 3044503, 3281618, 3170995, 3041940, 3281997, 2901252, 3192867, 2844876, 3204249, 3280999, 3230200, 2904931, 2899186, 2899234, 3350031, 3340118, 3257915, 3276409, 3131405, 3079582, 3121710, 3060511, 3336176, 3144329, 3358568, 2452940, 2897008, 3277506, 2893080, 3275568, 3398891, 2902263, 2902264, 2900919, 3260759, 3257670, 3357006, 3292338, 2894232, 3393193, 3169214, 2904392, 3050517, 3243304, 3280640, 2462025, 3047338, 3259410, 2898268, 3142566, 3127710, 2915238, 2536803, 2645385, 2661734, 2927449, 2674262, 2496240, 2912419, 2760359, 2671289, 2646536, 2788816, 2536538, 2521997, 2671740, 2547858, 2657423, 2668148, 2503724, 2648150, 2573572, 2647578, 2785610, 2777203, 2673129, 2671739, 2926468, 2768721, 2745867, 2703617, 2677721, 2644531, 2644532, 2674333, 2492454, 2643046, 2792117, 2651924, 2594037, 2761587, 2547030, 2654329, 2530317, 2644534, 2650646, 2642605, 2927479, 2549204, 2926945, 2498658, 2498657, 2541335, 2682243, 2691278, 2649413, 2642691, 2930837, 2564109, 2674336, 2671283, 2563046, 2569105, 2712744, 2919938, 2645345, 2647819, 2645950, 2672925, 2506966, 2702966, 2669536, 2679483, 2507028, 2656368, 2511136, 2643934, 2565517, 2566779, 2563985, 2492011, 2681331, 2492166, 2918332, 2531618, 2913914, 2513047, 2680795, 2645845, 2654435, 2684107, 2916880, 2573757, 2571627, 2547470, 2724486, 2530318, 2536257, 2547028, 2574108, 2721343, 2663610, 2910220, 2563951, 2782470, 2553879, 2913493, 2525897, 2523921, 2565105, 2911205, 2572902, 2783678, 2494249, 2572899, 2566736, 2564563, 2643772, 2544688, 2467711, 2810673, 2506965, 2570958, 2731750, 2564110, 2811970, 2495050, 2691252, 2789169, 2688972, 2643042, 2521816, 2492252, 2566827, 2502256, 2563045, 2665687, 2642536, 2515967, 2697567, 2731461, 2642538, 2493868, 2492832, 2674460, 2784648, 2492422, 2537349, 2716162, 2687688, 2547617, 2596943, 2802434, 2513939, 2570799, 2670580, 2495983, 2649324, 2680793, 2643356, 2653972, 2677092, 2573761, 2566048, 2651576, 2563744, 2767502, 2511939, 2685179, 2570234, 2644150, 2642284, 2513050, 2604760, 2707638, 2507061, 2469708, 2680794, 2754147, 2656362, 2571860, 2506969, 2715550, 2494454, 2664523, 2496815, 2543596, 2541193, 2570233, 2496789, 2563409, 2680583, 2571815, 2507027, 2681320, 2715552, 2710173, 2680584, 2564505, 2564011, 2549261, 2739017, 2643357, 2571009, 2910758, 2733125, 2911170, 2565997, 2500361, 2792664, 2669667, 2649330, 2730280, 2572617, 2915651, 2492354, 2507265, 2686478, 2656266, 2491940, 2687687, 2930095, 2659995, 2724504, 2754149, 2538577, 2654406, 2465391, 2563743, 2713970, 2645848, 2673080, 2683921, 2737271, 2574732, 2571021, 2792658, 2503197, 2523957, 2648143, 2570956, 2767075, 2648144, 2668006, 2504796, 2469711, 2685180, 2802431, 2495079, 2567896, 2707117, 2563454, 2681554, 2643933, 2759221, 2654256, 2913138, 2702970, 2563741, 2809002, 2643773, 2788085, 2683917, 2624617, 2677725, 2563797, 2493409, 2913132, 2504375, 2753406, 2806497, 2754150, 2503553, 2712613, 2513024, 2732449, 2512162, 2494240, 2572857, 2513966, 2655521, 2736749, 2657424, 2918157, 2688586, 2500195, 2495051, 2759546, 2571759, 2737602, 2654285, 2651226, 2500472, 2720925, 2492322, 2504944, 2684076, 2474057, 2504377, 2500196, 2532229, 2691568, 2564458, 2529059, 2778240, 2723246, 2680157, 2653461, 2648927, 2495779, 2491827, 2714570, 2792112, 2715803, 2697568, 2647817, 2647014, 2684085, 2663695, 2564901, 2567431, 2513931, 2565841, 2508813, 2674713, 2644465, 2651509, 2563787, 2506998, 2702897, 2647495, 2567266, 2684804, 2475589, 2916889, 2574262, 2684078, 2916871, 2502251, 2514824, 2685599, 2570959, 2564912, 2586565, 2539286, 2564763, 2493044, 2521184, 2673130, 2774849, 2503179, 2563096, 2739019, 2491867, 2556978, 2707399, 2503148, 2596940, 2777211, 2519530, 2473403, 2647494, 2645846, 2563788, 2567372, 2574764, 2513143, 2503723, 2566781, 2778913, 2497864, 2669998, 2584565, 2570798, 2677429, 2764657, 2666282, 2920999, 2599441, 2738261, 2658897, 2504359, 2681556, 2797066, 2785872, 2522475, 2494246, 2468745, 2566824, 2474480, 2670693, 2493302, 2508850, 2671116, 2915441, 2553993, 2567903, 2674459, 2504357, 2926470, 2475392, 2568519, 2681856, 2566735, 2567873, 2657428, 2684802, 2492325, 2693231, 2679473, 2808984, 2564058, 2909202, 2509917, 2513026, 2574566, 2733733, 2503114, 2465099, 2686575, 2540724, 2571864, 2567793, 2569550, 2802896, 2680158, 2471718, 2563465, 2787477, 2689493, 2914634, 2500169, 2666251, 2509103, 2744731, 2574304, 2539403, 2493897, 2682242, 2642543, 2478849, 2503150, 2742440, 2493869, 2564834, 2573768, 2788116, 2642875, 2600323, 2570730, 2645063, 2778478, 2598950, 2679288, 2742247, 2910264, 2680581, 2566778, 2776872, 2930092, 2538499, 2817624, 2468702, 2512486, 2811958, 2930328, 2913139, 2687691, 2719503, 2643038, 2681858, 2572616, 2572618, 2674331, 2478604, 2674335, 2702967, 2809024, 2688505, 2664522, 2509528, 2497168, 2918184, 2498414, 2659299, 2462943, 2930094, 2567893, 2915410, 2666484, 2927483, 2551537, 2691569, 2509916, 2563952, 2572797, 2684077, 2686576, 2789272, 2570971, 2572205, 2567190, 2567792, 2569600, 2566790, 2564948, 2471472, 2551765, 2811943, 2495053, 2510877, 2565999, 2506263, 2680866, 2735626, 2510845, 2232019, 1977531, 2225367, 2202791, 2244793, 2083186, 2256740, 2155958, 2138186, 2202901, 2122251, 2403654, 2078484, 2406328, 2200382, 2405806, 2404204, 2200966, 2184355, 2233917, 2181312, 2179096, 2203964, 2140432, 2200821, 2262649, 2203662, 2192563, 2107242, 1977781, 2374564, 2191991, 2172349, 2179479, 2157983, 1977080, 1699985, 2189954, 2404087, 2146370, 2242521, 2331203, 2153765, 1977079, 2127787, 2196954, 2118853, 2141247, 2140854, 2167952, 2185298, 2167953, 1964073, 2127690, 2193119, 2199622, 2174933, 2169587, 2299367, 2405271, 2109729, 2200380, 2186276, 2195346, 2278545, 2194128, 2247104, 2127786, 2115976, 2233904, 2186951, 2143062, 2332564, 2190865, 2121114, 2203494, 1975780, 1971590, 2179067, 2193560, 2179478, 2230895, 2407812, 2181311, 2181310, 2202789, 2179477, 2207401, 2144819, 1975426, 2262643, 2153719, 1977780, 2185293, 2224134, 2079568, 2179366, 1978873, 2407872, 2212369, 2152339, 2123904, 1967720, 2145400, 2254559, 2221162, 2194694, 2107898, 2212409, 2227289, 2242456, 2145347, 1975334, 2222132, 2127054, 2243429, 2404633, 2156475, 2375564, 2306061, 2174464, 2195106, 1979363, 2090472, 1978872, 2183632, 2153475, 1973470, 2383162, 2407813, 1978660, 2317911, 2131793, 2179481, 2199618, 2198165, 2391738, 2203556, 2144018, 1978771, 1969244, 2350209, 2390547, 2405822, 2213104, 2202903, 2115444, 2182697, 2311727, 1976147, 1976928, 2110033, 1967767, 2403867, 2146040, 2210463, 1978115, 2322723, 1973768, 1967425, 2122926, 1975333, 1967666, 2337698, 2376254, 2148176, 2262646, 2107241, 2191990, 2229765, 2151272, 2183735, 2403473, 2123902, 1975324, 2294326, 1974743, 2337713, 2344923, 1968126, 2222131, 1976938, 2184945, 1979376, 2193166, 1969560, 2157637, 2209307, 2226538, 2117622, 2229750, 2225384, 2152944, 2233916, 2190515, 2126475, 2111784, 2112906, 2166142, 2110198, 1971318, 2222103, 2244794, 2184790, 2229783, 1698846, 1699835, 1980218, 2162867, 1970070, 2294222, 2179363, 2148177, 1974942, 2212378, 2191636, 2116773, 1972487, 2190864, 2202458, 2406327, 2406323, 2404480, 1969870, 2188543, 2271852, 1972430, 2186825, 2110064, 1967717, 2311729, 2282112, 2254558, 1970108, 1969982, 2227819, 2186982, 2152777, 2374566, 1970027, 2137901, 2173646, 2407957, 2179066, 1697646, 2253903, 2376891, 2109197, 2105779, 2229775, 2215560, 1967418, 1978164, 1971320, 2205799, 2202790, 1977980, 2105988, 2351301, 2231756, 2271853, 2195345, 2105256, 1969509, 1971670, 2182917, 1971034, 2318105, 1968178, 2106402, 2240922, 2159250, 2406602, 2297284, 2138449, 2209315, 1699989, 2123907, 2293600, 1968919, 1967416, 2252453, 2244639, 2109002, 2289995, 2405804, 2342129, 2199681, 2141313, 1973969, 1968871, 2180591, 2181158, 2185340, 1689807, 2207419, 2178965, 2123819, 2407533, 2222100, 2178582, 2261927, 2404448, 1974941, 2241443, 2265137, 2190516, 2403818, 1967667, 2332569, 2148702, 1969869, 1695877, 2384645, 2332919, 2105990, 2225383, 2196466, 2199290, 2195108, 2222242, 2125513, 2180659, 2282920, 1967730, 2201495, 2107112, 2197908, 2171449, 2378547, 2200894, 2405042, 2180658, 2156476, 1971872, 2178535, 1975321, 2351011, 2197095, 2196207, 2210246, 2185299, 2294819, 2183055, 2230868, 2225374, 2184711, 2111786, 1978880, 1688616, 2110430, 2200384, 2304505, 1699861, 2167452, 2229837, 2170088, 2209318, 2244769, 2404046, 2107237, 2197208, 2331204, 2320062, 2205418, 2149704, 2139442, 2194047, 2184183, 1975851, 2199209, 1976576, 2174121, 1969060, 2224231, 1975322, 1693175, 2258142, 2079571, 1971503, 1971504, 2113611, 2194894, 2214100, 2197911, 2115407, 1698845, 2112184, 1976664, 1972218, 2179594, 2209305, 1974561, 2110199, 1977012, 2121490, 2300087, 2233906, 2118403, 2189951, 2090474, 2374273, 2123914, 2079574, 2197314, 2204661, 2323591, 2186832, 2222129, 2222130, 2182401, 2178939, 2179367, 2338194, 2143092, 2204739, 1970370, 2299370, 2179362, 2296053, 2209320, 2165831, 2182891, 2192562, 2184356, 2090478, 2301656, 2105795, 2142611, 2209306, 2209343, 2404483, 2322085, 2403775, 2144216, 2099751, 2213103, 2210238, 2350206, 2374275, 2255026, 2191020, 2210673, 2191867, 2246421, 2403952, 2146911, 2183633, 2188871, 2392131, 2151274, 2183732, 2256974, 2230890, 2111249, 2403655, 1706610, 2343912, 2224121, 2107240, 1967210, 2244643, 2348469, 2242106, 2378982, 2262647, 2191018, 2364165, 1706741, 2197450, 2224266, 2189950, 1694236, 1971166, 2123903, 2404631, 2365189, 2215592, 2226540, 1969066, 2199295, 1696309, 2202902, 2109195, 2405040, 2202792, 2152972, 2144019, 2203555, 1975893, 2169216, 2258157, 2142081, 2232062, 2079570, 2382916, 2407808, 1694235, 1688495, 2405920, 1696310, 2229749, 2127790, 2403428, 2407810, 1691125, 1978163, 2314446, 2242455, 2197909, 2153424, 2159054, 2182370, 2120310, 2157982, 2188696, 2209300, 2196962, 2179638, 2403824, 2351027, 2405107, 2261379, 1693065, 1973472, 1976563, 2184796, 2145212, 2127791, 2079567, 2186273, 2292255, 2105793, 1701029, 2205798, 2254557, 2210464, 2304504, 2190840, 2243428, 2215615, 1978871, 2115538, 1977779, 2344503, 2182696, 2404478, 2196946, 1695232, 2183665, 2212408, 2213107, 2344111, 2405805, 2183052, 1971861, 2179027, 1976875, 2183733, 2297824, 2179591, 1967729, 1972207, 1980220, 2229840, 2364508, 2354297, 1975891, 2403593, 1969068, 2227816, 2184795, 2167951, 2202451, 2107239, 2119263, 2172351, 2360750, 2242519, 1976937, 2112374, 2405108, 2191992, 2199349, 2196208, 2180388, 2405274, 2121486, 1971586, 1969017, 1975487, 2107926, 2190839, 1967134, 2183056, 2384652, 2191993, 2324409, 2233931, 2299096, 2178941, 2102668, 2215608, 2194126, 2186274, 2202445, 2247106, 1976665, 2139175, 2304216, 2178930, 2090473, 1979946, 1693173, 2104884, 2127257, 2407875, 2406601, 2261364, 2403953, 2138182, 2297206, 2295911, 2184180, 2152844, 2121487, 1968329, 1970466, 2184789, 2212256, 2124540, 2115755, 2123909, 2098016, 2127789, 2161633, 2265248, 2123901, 2185294, 2226542, 2151276, 2354706, 1975045, 1970032, 2079572, 2127785, 2358837, 2184794, 2153194, 2153193, 2221645, 2407208, 2407209, 2107780, 2183734, 1978161, 2184359, 1700077, 2403603, 1688617, 2282421, 2300106, 1689791, 1974940, 1705274, 2166758, 1975819, 1979796, 2155569, 2114350, 2178534, 1969247, 2199619, 2203481, 2178011, 1971596, 1969115, 2151275, 2151277, 1999025, 1992318, 2021299, 1722167, 1674853, 1878583, 1995917, 2013676, 1824249, 1922220, 2039377, 2057035, 1988573, 1922235, 2000109, 1778191, 1865542, 1649971, 1988829, 1944438, 1904551, 1832209, 1985041, 1944470, 1912915, 1994867, 1929762, 1828420, 2057036, 1722967, 1674105, 1674589, 1985163, 1894861, 1992319, 1928474, 2072138, 1680893, 1907228, 2033413, 1742480, 1902404, 1960557, 1991894, 2050321, 2051002, 1912556, 1888248, 1993790, 2051004, 1906532, 2054316, 1825097, 1960551, 2049321, 1760601, 1648597, 1646292, 1649267, 1805818, 2045855, 1709686, 1702148, 1922207, 2016616, 1870651, 1808221, 1656826, 1848679, 1742224, 1922243, 1709446, 1826739, 1797487, 1860640, 2056774, 1678705, 1944468, 1912887, 1904134, 1678800, 1960555, 1919626, 2043778, 1900575, 1755876, 2024862, 1710750, 2015394, 1832944, 1710921, 1992306, 1854108, 2012462, 1890264, 2026848, 1653901, 1683682, 1826506, 1883258, 1911460, 1773691, 1999634, 1683673, 1860682, 1960301, 1888931, 1744356, 1778190, 1702795, 2066075, 1918709, 1904288, 1747577, 1683602, 1676696, 1952471, 1941055, 2044734, 1837744, 1920701, 2021719, 2012456, 1747643, 2069491, 1922250, 1984891, 1958248, 1747614, 1856403, 1929761, 1670617, 2021297, 2066762, 1902418, 1960828, 1899682, 2070108, 1773686, 1998792, 2066757, 1714951, 1681265, 1991182, 1955122, 2066763, 1987229, 2005316, 2033429, 1860215, 2066755, 1856404, 1773685, 1683882, 1768163, 2016622, 2009034, 1773154, 1744360, 1984711, 1929764, 1845222, 1848996, 1941057, 1909218, 2019357, 1801880, 2065925, 1992957, 1906028, 2062329, 2052056, 1648488, 2016618, 1993815, 1704089, 1649265, 1678453, 1679736, 1915427, 1985164, 1714532, 2062330, 1671471, 2012448, 1853956, 1901072, 2050329, 1995100, 2016451, 1671434, 1856405, 1793725, 2013951, 1886157, 1984696, 2006699, 1773955, 1681264, 2060447, 1903404, 1744962, 1904989, 1907241, 2050913, 1955633, 2026847, 1648598, 2045854, 1986287, 1656828, 1674014, 1894864, 1720796, 1955637, 1960302, 2060433, 2013934, 1877851, 1954418, 2071784, 1936012, 1678031, 1987818, 1932973, 1890863, 1984898, 1782932, 1671884, 1837743, 1989572, 2054319, 1915428, 2060439, 2029996, 1677063, 1959195, 1670840, 1672967, 1658652, 1997850, 1825100, 2044550, 1905621, 2003706, 1991906, 1673134, 1673975, 2040466, 1672176, 1712835, 1905592, 2057034, 1673976, 1711001, 1856429, 1676776, 1880318, 1993796, 1954814, 1772296, 1929038, 1944425, 1682541, 1959859, 1653711, 1984187, 1830277, 2013669, 1848053, 2012458, 1854110, 1883119, 2070058, 2045613, 1997835, 1989570, 2041051, 2064484, 1680616, 1984710, 1683196, 1991366, 1797509, 1918724, 2013680, 2040042, 1744422, 1936001, 1675329, 1922206, 1954179, 1830551, 1682543, 1952472, 1671665, 1942422, 1677368, 1959863, 1888345, 1987410, 1860008, 1671993, 1884058, 1845438, 1681355, 1676467, 1860210, 1898585, 1960303, 1855676, 1955161, 2069490, 1905672, 1955630, 1988576, 1992877, 1682739, 1902405, 1914803, 2010166, 1939943, 1904892, 1904891, 1768158, 1823530, 1985608, 1671885, 2045618, 1960558, 1898630, 1920698, 2044736, 1746996, 1912833, 1898643, 1898640, 1742223, 1890860, 1880885, 1878641, 1752455, 2013954, 1985031, 1899080, 1906692, 1934382, 1679154, 1987817, 1985161, 1955002, 2021388, 2043493, 1899852, 1903663, 1936788, 2029988, 1922241, 1706666, 1993805, 2012351, 1678798, 1782954, 2013140, 1657248, 1814749, 2022014, 1671346, 1994794, 2071783, 1960560, 1875221, 1953218, 1670723, 2072144, 2016615, 1676779, 2060435, 1678039, 2072407, 2012358, 2009366, 1929022, 1824790, 1681343, 1658407, 1894852, 1900223, 1883123, 1932903, 2029111, 1678033, 1783090, 1756844, 1687129, 1664346, 1682593, 2071787, 1672841, 1999715, 1983848, 1944430, 1959860, 1773148, 1671113, 1993497, 2049311, 2064488, 1672588, 1922236, 1768630, 1855018, 1899364, 1674295, 1683981, 1710483, 1999883, 1954453, 1675763, 1778192, 1960304, 1743226, 2044551, 1827810, 1832382, 1992876, 1682159, 1770553, 1999012, 1860196, 1681275, 1672878, 1681224, 1900313, 1904477, 1959887, 1870247, 1882999, 1929687, 1863026, 1992214, 2014943, 1824248, 1673495, 1716338, 1782952, 1864532, 1889706, 1710922, 1888238, 1941060, 1901203, 1880321, 1752280, 1905501, 1890269, 2026339, 2033414, 1682683, 1864539, 1900101, 1674060, 1984877, 1999714, 1899433, 1824621, 1676392, 1995974, 1672921, 2064495, 1899365, 1682685, 1676083, 1649269, 1955631, 1993488, 1984743, 1889718, 1941065, 1890859, 2013952, 1883257, 1885075, 1783082, 1681365, 1863024, 1847315, 1681162, 2023353, 1850208, 1875966, 2043500, 1853989, 1895471, 2013161, 2013139, 2040325, 1655867, 2044903, 1912554, 2072147, 1834808, 1670725, 2067035, 2033963, 2043017, 2065688, 1831059, 1715501, 1713930, 1658648, 2024858, 1944428, 2001806, 1860199, 2058867, 2013416, 1898694, 1955634, 2045853, 2012511, 1953230, 1856402, 1679870, 1829757, 1848054, 1655136, 1675315, 2016630, 1922223, 1901528, 1922205, 1940256, 1833088, 1682684, 1683420, 1954178, 1742488, 1868024, 1954180, 1990191, 1904351, 1716340, 1716334, 2061981, 2001087, 1988333, 1889702, 2014947, 1675316, 2029994, 1710919, 1677698, 2050319, 1674294, 1797888, 1677894, 1914801, 1898644, 2003707, 1898641, 2033427, 1847977, 2016621, 1914097, 1954424, 1826281, 1789830, 1672001, 1710655, 1934379, 1954811, 1648142, 1875415, 1678797, 1678445, 1726639, 2013419, 1846784, 1898951, 1903847, 1900254, 1742482, 2041549, 1902417, 1672392, 2016833, 1711156, 2040324, 1993808, 1940267, 1960549, 1901527, 1709185, 1681268, 1804665, 2009033, 1676251, 1869725, 2015395, 1889697, 1883120, 1960313, 1992304, 2014945, 1671951, 1671890, 2050326, 1673505, 1671940, 1687131, 1774408, 2061979, 1985045, 1912918, 1992960, 2044924, 2034245, 1991925, 2013674, 2016625, 1991923, 2019378, 1991922, 2050997, 1671347, 1677695, 1710631, 1929035, 1889499, 1992187, 1672539, 1682643, 1797498, 2012514, 1860197, 1797506, 1671631, 1650315, 1722697, 1653363, 1702144, 1891022, 1714948, 1888251, 1984762, 1673209, 2060415, 1651994, 2039897, 2018160, 2027357, 1860625, 2071786, 2005734, 1744358, 1652604, 1673977, 1675368, 1953228, 1991926, 1828336, 1778193, 1941059, 1852180, 1903846, 2005733, 1822966, 1746997, 1929681, 1999706, 1874490, 1712803, 2033418, 1889493, 1984386, 1702796, 2065685, 1954810, 1656827, 1903405, 1874487, 1806376, 1782951, 1889719, 1726637, 1912917, 1992843, 1929765, 1671241, 2053632, 2025147, 1851894, 1712836, 2017225, 2009895, 1988827, 1960378, 1900410, 1929683, 1673953, 1961221, 2012354, 1847976, 1984893, 1670734, 1747675, 2050342, 1648976, 1998791, 1672389, 1744368, 1984763, 1904028, 1988575, 2016626, 1991440, 1678028, 1859951, 1890866, 1960829, 2012453, 1727744, 1606981, 1417428, 1315294, 1426215, 1346794, 1629507, 1607550, 1403038, 1345754, 1355595, 1739355, 1732770, 1357554, 1522841, 1396839, 1298221, 1435883, 1512910, 1353607, 1734249, 1320740, 1732771, 1325036, 1360145, 1435930, 1578593, 1346337, 1538722, 1542320, 1313549, 1587429, 1569973, 1447500, 1421390, 1522842, 1404819, 1448121, 1590494, 1728157, 1550410, 1505560, 1406817, 1460211, 1503326, 1732427, 1607916, 1329697, 1362432, 1618486, 1453208, 1452083, 1451242, 1433686, 1607913, 1423954, 1640952, 1487608, 1551634, 1575253, 1732429, 1318742, 1537492, 1552913, 1608408, 1591828, 1303639, 1734247, 1530798, 1559584, 1597230, 1727754, 1406880, 1739236, 1740861, 1740585, 1583761, 1423946, 1517787, 1534287, 1499708, 1401622, 1403034, 1333518, 1472184, 1453199, 1403033, 1499947, 1578927, 1552109, 1370510, 1370511, 1545092, 1586710, 1578909, 1738379, 1499461, 1348161, 1415824, 1359322, 1520082, 1527255, 1644087, 1527286, 1512329, 1443986, 1552919, 1500686, 1346819, 1739360, 1532940, 1546860, 1336735, 1350596, 1578912, 1499480, 1537130, 1737544, 1352375, 1347278, 1430701, 1555628, 1734985, 1728438, 1355153, 1404448, 1358674, 1351703, 1358932, 1577155, 1628722, 1612362, 1392755, 1575250, 1289091, 1571644, 1599353, 1332634, 1547683, 1350809, 1644089, 1740589, 1512911, 1383821, 1286044, 1739794, 1587408, 1456855, 1733381, 1376735, 1602805, 1468283, 1358037, 1330079, 1569444, 1567096, 1558466, 1558439, 1318765, 1346564, 1417429, 1547684, 1345755, 1584260, 1346171, 1434838, 1443245, 1354616, 1727915, 1396829, 1596562, 1383819, 1727236, 1346408, 1637021, 1499473, 1458144, 1427365, 1520045, 1740587, 1613206, 1538721, 1426218, 1426224, 1359318, 1361594, 1331484, 1633517, 1346817, 1460202, 1357349, 1583760, 1458074, 1507376, 1730830, 1357453, 1613201, 1734740, 1535495, 1348805, 1349376, 1616322, 1591831, 1472183, 1356175, 1582601, 1516498, 1348226, 1597214, 1357450, 1398229, 1512327, 1734254, 1577026, 1452929, 1417430, 1554040, 1578922, 1370236, 1606067, 1730080, 1346414, 1444727, 1735142, 1555618, 1469179, 1312317, 1486457, 1602793, 1417433, 1536276, 1361175, 1735925, 1578953, 1644325, 1535419, 1597911, 1398497, 1430705, 1644341, 1383099, 1729317, 1330676, 1333181, 1351127, 1444880, 1531328, 1360557, 1644335, 1616359, 1323324, 1537131, 1351620, 1623866, 1578935, 1349990, 1360087, 1447502, 1351196, 1392754, 1547419, 1356776, 1351183, 1401602, 1560168, 1444723, 1505563, 1739365, 1612357, 1386652, 1737539, 1560166, 1527296, 1545080, 1359258, 1443951, 1572445, 1330678, 1423942, 1406879, 1446868, 1644095, 1397878, 1360558, 1386825, 1352564, 1740678, 1393035, 1325762, 1592346, 1583246, 1577028, 1558340, 1467719, 1359211, 1738173, 1537493, 1560167, 1390306, 1545086, 1535083, 1624154, 1427379, 1552095, 1356117, 1531993, 1348481, 1310160, 1560801, 1593034, 1512341, 1486900, 1582603, 1737529, 1347866, 1732347, 1580727, 1317694, 1524334, 1354276, 1349062, 1321177, 1634662, 1588377, 1542321, 1638712, 1495520, 1592345, 1347091, 1396838, 1600993, 1346008, 1540019, 1357350, 1309390, 1320133, 1351600, 1456861, 1587405, 1477566, 1360793, 1505562, 1616214, 1371323, 1584258, 1582586, 1568574, 1579136, 1355209, 1578910, 1449552, 1486974, 1370509, 1281103, 1505554, 1730841, 1555273, 1740677, 1337465, 1569442, 1500694, 1558344, 1548519, 1622852, 1337466, 1545094, 1401644, 1308663, 1616890, 1392887, 1527313, 1575571, 1538013, 1638713, 1597229, 1346170, 1728934, 1580724, 1308130, 1624171, 1381626, 1728202, 1345803, 1567097, 1586270, 1554036, 1530752, 1313369, 1506656, 1357295, 1607535, 1296590, 1398493, 1548522, 1310584, 1522840, 1423948, 1571681, 1343207, 1568727, 1591822, 1727913, 1430700, 1351126, 1516491, 1346818, 1586708, 1740583, 1552746, 1731134, 1517791, 1569443, 1552106, 1588979, 1512330, 1534001, 1346283, 1584259, 1593057, 1356779, 1345826, 1348751, 1426223, 1736766, 1392795, 1426202, 1398238, 1354225, 1560798, 1578942, 1536941, 1578949, 1578945, 1406835, 1568716, 1583243, 1555284, 1451240, 1537519, 1616360, 1333267, 1614462, 1351547, 1503142, 1452916, 1535082, 1386857, 1730079, 1729575, 1347801, 1469180, 1320131, 1608407, 1348515, 1625490, 1545097, 1551541, 1532970, 1323587, 1622858, 1729573, 1623868, 1629506, 1357550, 1434838, 1392827, 1644101, 1533595, 1452917, 1738183, 1634914, 1588379, 1352567, 1383434, 1404796, 1516489, 1463951, 1281177, 1588378, 1443954, 1430679, 1348750, 1516506, 1549149, 1640953, 1468285, 1522839, 1638252, 1569602, 1516500, 1350803, 1345799, 1578921, 1586706, 1346172, 1739372, 1739366, 1396832, 1555623, 1505561, 1617789, 1444726, 1345133, 1500629, 1737543, 1314192, 1321893, 1390316, 1285740, 1599343, 1397884, 1730831, 1489388, 1427656, 1416572, 1737157, 1735149, 1497392, 1568660, 1357297, 1634054, 1548825, 1612492, 1624155, 1729625, 1321894, 1512328, 1732133, 1380024, 1360855, 1430680, 1385147, 1423955, 1370924, 1728190, 1396808, 1444728, 1586709, 1558463, 1289367, 1586105, 1352012, 1559090, 1356176, 1290475, 1537129, 1345953, 1450043, 1459154, 1464341, 1503133, 1546915, 1555269, 1572037, 1618211, 1547673, 1352011, 1586733, 1524328, 1469177, 1578042, 1354274, 1307234, 1728443, 1351538, 1464336, 1453197, 1727614, 1348797, 1327082, 1347280, 1352363, 1347336, 1568586, 1357462, 1612344, 1350192, 1430708, 1351489, 1486963, 1636805, 1535321, 1530193, 1543307, 1359260, 1586104, 1740582, 1607520, 1532760, 1314910, 1359317, 1634913, 1737542, 1349676, 1349112, 1628013, 1354275, 1581716, 1638191, 1586788, 1586782, 1357242, 1346182, 1360032, 1349049, 1483075, 1359210, 1353803, 1392995, 1599348, 1468291, 1353804, 1310103, 1578954, 1571645, 1498061, 1347854, 1353134, 1357306, 1498073, 1423952, 1281903, 1313340, 1423953, 1406862, 1349458, 1357451, 1396833, 1608405, 1536959, 1515637, 1417133, 1390305, 1607528, 1617763, 1607532, 1347803, 1416576, 1638251, 1543310, 1629503, 1404449, 1632658, 1633520, 1617762, 1545849, 1540729, 1433739, 1538559, 1628727, 1730835, 1361435, 1281180, 1346877, 1732370, 1460207, 1586618, 1445500, 1503329, 1355211, 1597912, 1588374, 1568575, 1345870, 1732769, 1401645, 1355798, 1393034, 1486965, 1346793, 1283370, 1727801, 1531994, 1498074, 1426214, 1728249, 1627030, 1728930, 1392793, 1599352, 1730840, 1618465, 1447501, 1545091, 1453211, 1586272, 1543305, 1426866, 1727961, 1537516, 1555625, 1527304, 1384364, 1547677, 1545464, 1732434, 1505329, 1560172, 1350562, 1460210, 1586821, 1552108, 1359221, 1499482, 1486964, 1600983, 1520049, 1732652, 1383816, 1545089, 1560164, 1331217, 1289369, 1568584, 1352789, 1426204, 1360991, 1578941, 1430709, 1731135, 1569453, 1572446, 1598096, 1396820, 1450064, 1728731, 1622856, 1329698, 1728908, 1560163, 1573749, 1388181, 1578940, 1427652, 1578934, 1623865, 1617766, 1728444, 1386823, 1357551, 1737545, 1737537, 1583252, 1729619, 1334465, 1359846, 1346413, 1417380, 1577025, 1578948, 1323943, 1302472, 1322075, 1532198, 1732426, 1611329, 1596044, 1734739, 1635566, 1606429, 1520054, 1548525, 1500630, 1729617, 1534476, 1620169, 1371211, 1380989, 1727803, 1416574, 1350428, 1727093, 1353742, 1450046, 1346410, 1458072, 1503330, 1505304, 1322118, 1464342, 1551535, 1435880, 1505913, 1398498, 1350804, 1468295, 1545087, 1464343, 1349666, 1562720, 1612153, 1463945, 1644080, 1728160, 1353192, 1346951, 1347582, 1406859, 1393074, 1506638, 1355797, 8179648, 8245326, 8282251, 8408940, 8103820, 8410127, 8409054, 8406348, 8332150, 8018138, 8336751, 8316267, 8316266, 8332149, 8339809, 8422221, 8433725, 8433726, 8472719, 8469251, 8413431, 8413432, 8095240, 8371727, 8445223, 8454793, 7692297, 8487823, 8510706, 8123097, 8474201, 8314497, 8247075, 8350884, 8239849, 7794289, 8450166, 8370371, 8498878, 8370370, 8422085, 8417553, 8257205, 8437304, 8496536, 8349933, 8454796, 8363165, 8227779, 8458362, 8501499, 8357287, 8254858, 8392957, 8489327, 8245337, 8335049, 8350885, 8417382, 8323420, 8417049, 8471778, 8504501, 8301064, 8418403, 8418228, 7907668, 8490166, 8400255, 8251808, 8353072, 8328559, 8332152, 8445418, 8338511, 8215728, 8445225, 8410114, 8491010, 7690462, 8477961, 8093291, 8480823, 8174950, 8349942, 8101532, 8227809, 8458364, 8444426, 8102669, 8229121, 8445413, 8518219, 8381187, 8311536, 8484698, 8445420, 8418238, 8391063, 8410124, 8416406, 8315263, 8241913, 8339808, 8095941, 8315260, 8340603, 8498881, 8094768, 8094279, 8501503, 8333815, 8406147, 8315425, 8394849, 8508863, 8274892, 8227722, 8215814, 8323473, 8335858, 8409041, 8241852, 8409113, 8491007, 8222097, 8473644, 8355039, 8262084, 8315433, 8335191, 8339412, 8509579, 7901637, 8101254, 8319334, 7902479, 8252662, 8416325, 8103146, 8471555, 8292113, 8422091, 8363210, 8352671, 8096941, 8112444, 8100913, 8418302, 8340983, 8339411, 8417077, 8487062, 8335833, 8350363, 8435030, 8096373, 8376696, 8478672, 8260695, 8432448, 8477962, 8287864, 8219947, 8409337, 8388286, 8452323, 8387607, 8240996, 8104273, 8436744, 8227822, 8192751, 7693881, 8433469, 8509532, 8185728, 8394880, 8391064, 8379600, 8518642, 8425987, 8386921, 8103876, 8293756, 8227867, 8123120, 7680764, 8487326, 8443900, 8479489, 8104966, 8504970, 8417578, 8404944, 8416267, 8401094, 8299441, 8472827, 8339409, 8387933, 8461812, 8227844, 8443481, 8401048, 8473686, 8238640, 8315261, 8214993, 8094826, 8094834, 8257887, 8432296, 8519379, 8245325, 8413388, 8425695, 8214995, 8299431, 8479488, 8417552, 8230266, 8440429, 8454794, 8335048, 8102428, 8123056, 8461472, 8379551, 8496526, 7901531, 8103027, 8426213, 8280213, 8239245, 8426205, 8096958, 8504499, 8244135, 8229120, 8498759, 8315422, 8336198, 8182272, 8443479, 8418243, 7901256, 8299637, 8335855, 8432442, 8094111, 8328566, 8509565, 8480832, 8328732, 8360399, 8432299, 8285927, 8386652, 8281056, 8418402, 8093705, 8459057, 8174967, 8241496, 8360391, 8374373, 8379601, 8421475, 8099593, 8388285, 8252691, 8410128, 8350886, 8502270, 8257472, 8436780, 8174949, 8093267, 8314512, 8339807, 8379564, 8436777, 8101302, 8422824, 8229122, 8258611, 8112439, 7693785, 8333807, 8491012, 8355740, 8385040, 8104270, 8419816, 8472980, 8490471, 8473654, 8389421, 8409072, 8110249, 8328575, 8491304, 8478958, 8462137, 7678844, 8425683, 8343034, 8499815, 8449203, 8366227, 8105165, 8462384, 8422814, 8301062, 8252687, 8097170, 8459060, 7691911, 8101772, 8425702, 8495604, 8227787, 8317948, 8462390, 8393028, 8151104, 8100289, 8343668, 8428718, 8174947, 8314468, 8441428, 8244130, 8299455, 8222122, 8131770, 8151095, 8515787, 8357360, 8117360, 8473676, 8100928, 8480959, 8497284, 8418401, 8314480, 8325315, 8439113, 8489326, 8376682, 8363164, 8244132, 8102430, 8472981, 8498757, 8174966, 7694148, 8360397, 8335047, 8357283, 8435601, 8509530, 8387576, 8101589, 8375421, 8403306, 8405850, 8392297, 7505008, 8487048, 8227791, 8375437, 8444427, 8490411, 8335849, 8504976, 8359644, 8500745, 8245359, 7682581, 8442620, 8436755, 8417806, 8465871, 8462805, 8406169, 8380243, 8380242, 8491393, 8229143, 8098788, 8375242, 8262083, 8214179, 8096956, 8403290, 8403307, 8509566, 8246027, 7689519, 8482458, 7686120, 8314475, 8489107, 8270721, 8507125, 8095127, 7512110, 8329917, 7903034, 8239224, 8323383, 8336190, 8494079, 8433727, 8230265, 8099637, 8501502, 8445429, 8229130, 8519378, 8381164, 8269792, 8445428, 8244121, 8229139, 8422839, 8420442, 8404433, 8245327, 8227803, 8419003, 8117604, 8100910, 8228122, 8365423, 8417381, 8245329, 8381091, 8436775, 8401013, 8445422, 8325299, 7693506, 8406151, 8393019, 8247036, 8406153, 7688335, 8096416, 8315435, 8391041, 8314476, 8314485, 8444411, 8382969, 8244259, 8258615, 8227863, 8508864, 8489114, 8093495, 8459068, 8406150, 8314495, 8482456, 8299437, 8436740, 8370428, 8103144, 8103874, 8426198, 8404935, 8455298, 8370356, 8096945, 7687978, 8250678, 8465885, 8101583, 8491949, 8096228, 8299622, 8432221, 8461475, 8501488, 7694894, 8478660, 8245335, 8452322, 8315258, 7689971, 8465870, 8321259, 8314499, 8331682, 8246033, 8105737, 8339398, 8252676, 8381758, 8093915, 8353925, 8292119, 8391065, 8422223, 8422222, 8357293, 8102048, 8482443, 8315426, 8359098, 8404438, 8269798, 8498758, 8472829, 8495154, 8484675, 8230622, 8510705, 8314483, 8292945, 8410110, 8374375, 8287870, 8315423, 8349938, 8453092, 8250683, 8388007, 8496540, 8404942, 8417242, 8425711, 8215749, 8287938, 8491302, 8095507, 8294627, 8442622, 8360932, 8360931, 8400973, 8103145, 8418225, 8094114, 8509578, 8455665, 8455664, 8246028, 8355037, 8380540, 8425685, 8353089, 8326972, 8445231, 8214177, 8450161, 8427515, 7680374, 8486876, 8425703, 8422080, 8482455, 7905413, 7901342, 8480824, 8487053, 8094467, 8315428, 8478668, 8459063, 8418345, 8406350, 8340055, 8487059, 8099640, 8357297, 8097802, 8252685, 8314510, 8101304, 8400933, 8215734, 8355383, 8100911, 8510708, 8315437, 8445427, 8324432, 7684512, 8101300, 8413387, 8338943, 8280653, 8299450, 8386650, 8245333, 8098392, 8461815, 8413475, 8352668, 8301065, 8224655, 8501490, 7901254, 8428715, 8468720, 8227846, 8411242, 8498876, 8446137, 8262115, 8403296, 8333805, 7684456, 8323380, 8387637, 8487046, 8329700, 7683942, 8418246, 8315424, 8446136, 8280654, 8473650, 8225214, 7901585, 8099639, 8251842, 8102427, 8101584, 8105213, 8478959, 8435362, 8336195, 8478661, 8518220, 8410122, 8166777, 8445432, 8409069, 8335807, 8386751, 8426211, 8515495, 8391042, 8244149, 8101900, 7691256, 8095573, 8232429, 8383088, 8487324, 8343737, 8492320, 8474513, 8093742, 8333814, 8498761, 8377777, 8225212, 8445234, 8227798, 8104272, 8349939, 8349935, 8223740, 8462386, 8105098, 8422204, 8408933, 8314474, 8388918, 7678046, 8338289, 8102665, 8269790, 7688253, 8354807, 8222099, 8182270, 7901110, 8227788, 7901582, 8098238, 8477964, 8490414, 8387940, 7678047, 8335810, 8352673, 8454792, 8468765, 8099380, 8446138, 8227859, 8470851, 8490338, 8491934, 8391792, 8215003, 8094485, 8419248, 8319361, 7687608, 7509619, 7901636, 8487050, 8436747, 8409070, 8380185, 8518572, 8325622, 8410113, 8311533, 7693504, 8335859, 8409112, 8413456, 8473679, 8232485, 8099592, 8239223, 8258620, 8147960, 8388006, 8404943, 8441426, 8472720, 7901049, 8131769, 8385230, 8437596, 8464431, 8454791, 8487051, 8445814, 7686216, 8325311, 8449202, 8396534, 8390232, 8472828, 8250653, 8512162, 8404931, 8336377, 8299434, 8363987, 8466376, 8099549, 8214998, 8112441, 8250615, 8098084, 8245328, 8375422, 8504495, 8477979, 8416161, 8340985, 8101534, 8479462, 8262130, 8287887, 8425995, 8498768, 7683989, 7686065, 7901473, 8495155, 8364194, 8423275, 8509640, 8151105, 8314479, 8314481, 8426204, 8385741, 8380765, 8251778, 8427957, 8377779, 8336373, 8418327, 8101048, 8214997, 8499823, 8380295, 8361220, 8509627, 8389710, 8432481, 8443480, 8253362, 8329922, 8117608, 8315262, 8151107, 8376688, 8095622, 8335816, 8102720, 8102511, 8466377, 8417634, 8425992, 8461646, 8505940, 8097082, 8100611, 7848882, 8005129, 7980110, 8174987, 8139083, 8174881, 8042889, 8147544, 7906384, 8186159, 8041413, 8078530, 7915824, 8025741, 7955195, 8281660, 7926495, 7523946, 8309029, 7935657, 8133853, 7949088, 8302340, 8166318, 8284002, 7956629, 7798545, 8299916, 7512838, 7890905, 7963150, 8267111, 8083452, 8162989, 8031229, 7521313, 7506225, 7737257, 7921099, 8078528, 8018001, 7916540, 7737253, 7967730, 8144799, 8149522, 8194687, 8109654, 7910231, 7913907, 8201376, 7951808, 8045513, 8041414, 8021731, 8124798, 7966901, 7890900, 8129455, 7836705, 7918123, 8289481, 7509381, 8090162, 7514046, 8289849, 8267489, 8167351, 8090163, 7935701, 8158698, 8043059, 8021734, 8114860, 8277068, 8301766, 8274110, 8075732, 7930473, 8120559, 7520770, 7537666, 8077527, 7956628, 7819986, 7698136, 8080985, 7995864, 8076659, 7995270, 8076917, 8006266, 7926899, 8049831, 7925510, 8057661, 7913155, 8070481, 7993148, 7955182, 8026054, 7931477, 8201375, 8164049, 7910275, 8026055, 8306050, 8118539, 7989932, 8037259, 7977882, 8277075, 8037263, 8193351, 8173763, 8195536, 7930276, 7983993, 8306021, 7698130, 7910120, 8014294, 7963155, 8281674, 7994817, 7699154, 8074600, 8147677, 8005121, 7698138, 7909049, 8147452, 7909098, 8013509, 7888849, 8312783, 8180400, 7882815, 8006397, 8106700, 8106695, 7959185, 8143994, 7914959, 8270984, 8113836, 8083709, 8042824, 7875267, 8267120, 8080349, 8256988, 8112186, 8005126, 8135446, 7993161, 7994822, 7835373, 8154643, 7908410, 7909874, 7927247, 8120917, 8111604, 7875272, 8129501, 8087928, 8270968, 7949185, 7924775, 8122946, 8053615, 7932825, 8311368, 8294678, 8201372, 7912347, 7978715, 7906809, 8193690, 8291819, 8026290, 8008027, 7983959, 8179463, 8067483, 8077535, 7905549, 8281698, 8294680, 7964957, 8276350, 7956639, 8200567, 7696164, 8031206, 8117177, 7882817, 7907073, 7977878, 8062601, 8040678, 7821310, 7828688, 8005124, 8083715, 7921460, 8180641, 8020806, 7527453, 8006262, 8111572, 8010359, 7914564, 8135447, 7916398, 7805711, 7963104, 7726876, 7921452, 8022438, 8014286, 8083453, 7875275, 8006281, 7930301, 7873457, 7984193, 8185149, 7916401, 7706568, 8113849, 8150348, 7935655, 7930468, 7997000, 7911175, 7959241, 8196726, 7963418, 8138256, 7873461, 8143996, 7914620, 7930223, 8124803, 7944874, 7905722, 7517720, 7523607, 7829013, 7798537, 8159193, 8080392, 8294092, 7819938, 7907732, 8276209, 7955181, 7698137, 8056000, 7818631, 8184997, 8086868, 7772114, 7875268, 7934434, 7905097, 8080512, 8273981, 7848884, 7989942, 7713100, 7908610, 8297216, 8297217, 8044941, 7908358, 7912296, 7964949, 7906759, 7925519, 8294090, 8056004, 7930273, 7933370, 7921429, 8026287, 8174988, 8145782, 7508827, 7503821, 8182225, 8050541, 8013602, 7835362, 8176095, 7913906, 8295285, 7933324, 7921428, 7906330, 7997002, 8174979, 7882821, 8057515, 7866080, 8007079, 7908501, 8151855, 8049846, 7957835, 7828695, 8173753, 8151854, 8026000, 8174879, 7734010, 8043878, 7982415, 8167489, 7930255, 8167511, 7979853, 7996960, 8020815, 8136666, 8185424, 8147547, 7952551, 8164029, 7930199, 8077542, 7864934, 8267134, 7905550, 7979855, 8307445, 7509383, 8201224, 7912249, 7988607, 7913157, 7934538, 7921095, 8185007, 8087328, 8112187, 7914260, 8113548, 8203993, 7508828, 7923656, 8006284, 8180536, 7519567, 8267129, 7713205, 8025984, 8277950, 7958682, 8119527, 7990242, 8311557, 7836708, 8124838, 8076768, 7524759, 7699233, 8062609, 7930237, 7943463, 7818648, 8124836, 7925518, 7836713, 7930028, 8049826, 8005128, 8058076, 7969280, 7963115, 8049833, 7966841, 7932809, 7910323, 8112185, 8044943, 8164026, 8044945, 8006252, 7848401, 8124787, 8017740, 7931490, 7979841, 8082525, 8205676, 8120960, 8163757, 7910230, 7982429, 8195519, 8006396, 8173365, 7925509, 7993413, 7980747, 8087329, 8133536, 7699226, 8200551, 8133537, 7829006, 8201379, 7978718, 7989941, 7532501, 8176802, 8113560, 8082522, 8113850, 7916020, 7906386, 8056005, 8190152, 8034872, 8055995, 8050544, 8291822, 7930274, 8200556, 7988298, 7934445, 7520676, 7931476, 8142791, 7509652, 7986180, 8276366, 7989945, 8161288, 7930300, 8143995, 8082533, 7959186, 8068956, 7989928, 7737255, 7507504, 8004326, 7921469, 7910229, 7925882, 7958669, 7910322, 7904978, 8125392, 8161290, 8277066, 7934351, 8004320, 8014289, 8294085, 8139084, 8304654, 7995386, 7713201, 8107739, 7519005, 7995861, 7923644, 7875277, 8295321, 7957830, 7988301, 8159194, 8144761, 7910089, 7912766, 7890902, 8068949, 7907729, 7819847, 7944835, 7959187, 8018041, 8176099, 8164027, 7935656, 8082521, 8041415, 8067649, 8031230, 7512679, 7713197, 8200552, 8049448, 8277547, 8078162, 8046805, 7950558, 7523271, 7908246, 7957836, 7930025, 8025759, 8025768, 7930243, 7848404, 8078551, 8044953, 7994829, 8107741, 7516951, 8053616, 7977884, 7944639, 7993153, 8025985, 7506013, 7934431, 8203989, 8044938, 7916073, 8064075, 8127332, 7935702, 8093132, 7904002, 7910272, 8076657, 8143016, 8044944, 7511106, 8137684, 8139085, 8067495, 8112194, 8004312, 7979840, 7925517, 8018006, 7931475, 8124800, 8111257, 7908076, 8281696, 8112188, 7826905, 8014444, 7989934, 8093139, 8196727, 8172436, 7914307, 8044962, 7934346, 7828694, 8087332, 7517442, 7905256, 7505563, 8148710, 8019248, 8081005, 7989943, 8124176, 7911176, 8026024, 8028614, 8053613, 7794302, 7924783, 7523739, 7819846, 7826913, 8027495, 8151058, 8080345, 7925507, 8313548, 7994805, 8151323, 8015129, 7950736, 8092339, 7930304, 7524736, 8022437, 8004299, 8111606, 8018037, 8087360, 7913731, 7930246, 7930480, 8301791, 8087361, 8058074, 7875274, 7925877, 8049819, 8080348, 8135449, 8181115, 7698134, 8173750, 7963427, 7926494, 8273984, 8200548, 7848395, 8174569, 8186174, 7979888, 7964961, 7836706, 7959238, 7926910, 7880228, 8078529, 8005127, 7993831, 8013600, 8289475, 8186157, 7944845, 8118531, 8075030, 8042927, 7952624, 8150356, 7508396, 7532991, 7969342, 8076921, 8067497, 7919326, 8111576, 8040679, 8013609, 8186168, 8074599, 8163780, 8084354, 7828971, 7969324, 8083697, 8040674, 8113843, 7964942, 8158680, 7964947, 8010369, 7988302, 7516250, 7521308, 8025745, 7510813, 7989940, 8201374, 7915348, 7911177, 8149523, 8045501, 8020904, 8004297, 8045490, 7950564, 8205670, 7934344, 7509355, 8301790, 7966415, 8119688, 7888847, 7977894, 8077528, 8291821, 8004292, 7515643, 7956634, 8201222, 8042821, 7906329, 7921461, 8172437, 8271298, 7829015, 8151900, 7963426, 7890893, 7993833, 8049437, 7915733, 8123837, 8152445, 7952623, 7964948, 8085688, 8270987, 7982419, 8113835, 7931470, 7983951, 7927227, 8124794, 8158817, 7905041, 7967990, 8122955, 8083708, 8299896, 8294693, 7526096, 8162979, 7988608, 8143833, 8188164, 7925516, 8083699, 8174448, 7931478, 8276370, 8306022, 7677801, 7968119, 8113557, 8172793, 7522527, 7993835, 8040669, 7994025, 7512131, 8113846, 7955184, 7914960, 7950612, 7950723, 8136665, 8055999, 7968075, 7916399, 7934491, 7964960, 8201373, 8164045, 7916403, 8201735, 7930242, 7519132, 7512124, 7521907, 8113844, 8083704, 7989938, 7508826, 7968073, 7908357, 7934537, 7996958, 8172789, 7772106, 7909099, 7915773, 7914611, 7912767, 7914962, 7911921, 7983954, 7526098, 7931474, 8201089, 7835358, 7505809, 8149521, 8196140, 8181121, 8045488, 8164030, 8142663, 8193698, 8093148, 7920126, 8026057, 8093138, 8276367, 7935654, 8113547, 8004298, 7910880, 8270982, 8181124, 7980764, 7909822, 8020690, 7908268, 8111598, 7994827, 7808029, 8056007, 8087352, 7514366, 7509244, 8040666, 8308183, 7921131, 8196120, 7933425, 7923646, 7528662, 7909853, 8040676, 8158702, 8270988, 8174993, 8267110, 8149518, 7534320, 8136670, 8296887, 8124790, 7755720, 7982430, 7963156, 7930305, 8135445, 8143997, 8027497, 8015121, 7916274, 7866082, 8122957, 7518480, 8307468, 7923674, 7978695, 8090164, 8179465, 8020902, 8164053, 8040675, 8142829, 7699155, 7995387, 8006401, 7814794, 7910321, 8006249, 8175136, 8291826, 7933396, 7913730, 8004311, 7911136, 8139627, 7512101, 8173754, 8015127, 8311374, 8114857, 7956631, 7713200, 7734015, 7966893, 8174444, 7836711, 7826904, 8162986, 8192551, 8064072, 8051381, 8005130, 8116990, 8052271, 7989939, 8201393, 8148935, 7993155, 7821128, 7989704, 8049820, 8201388, 8250455, 7828692, 7537664, 7516361, 8270972, 8114833, 8006274, 7994812, 8010365, 8074597, 8151317, 7931484, 7931483, 8006395, 7506223, 8188163, 8259188, 7977879, 8039612, 8046120, 7815836, 7964952, 7815837, 8198341, 8026027, 8161291, 8040671, 8076658, 7737258, 7955188, 7957464, 7957468, 8174890, 8152446, 8121459, 8018004, 7706571, 8044950, 8026282, 8304656, 8143828, 7994860, 8062610, 8289855, 7930196, 8087930, 8025983, 7531487, 8172790, 7907677, 7907731, 8148707, 7751892, 7877647, 7799039, 7496238, 7875666, 7744087, 8529731, 7849295, 7643880, 7596370, 7751528, 7477219, 7739681, 7823995, 7565948, 7760866, 7791837, 7675045, 7532074, 7579469, 7698053, 7643882, 7616988, 7791836, 7555513, 7671352, 7776987, 7586249, 7842188, 7527493, 8541559, 8549944, 7560468, 7838190, 7616989, 7544439, 7672685, 7539109, 7723545, 7751531, 7555551, 7791258, 7771913, 7843707, 7849875, 7625469, 8543745, 7554207, 7895354, 7565975, 8589024, 7675047, 7729640, 7477217, 7477168, 7723385, 7805224, 7586263, 7635414, 7702447, 7829792, 7715038, 8536775, 7563531, 7891486, 7605984, 7854376, 7620184, 7744141, 7854375, 7580707, 7715644, 7723543, 7715645, 7554180, 7633701, 7657280, 7755223, 7852669, 7477167, 7565974, 7675044, 7872852, 7843696, 7594076, 8682055, 7579435, 8881849, 7789372, 7671898, 7767151, 7608428, 7641363, 7722125, 8523049, 7752269, 8624291, 8575494, 8722064, 7860911, 7895356, 7888887, 7589378, 7718899, 8529737, 7776775, 8589025, 7656956, 7539890, 7884951, 8869018, 8542870, 8746902, 7860900, 7560624, 7498208, 8620948, 7866213, 7800005, 7486464, 7694905, 7622767, 8582392, 8869017, 7827549, 7767417, 7557852, 7897120, 7594028, 7594027, 7697837, 7769900, 7827548, 7602348, 8573537, 7842190, 7805221, 7738616, 7842191, 7852670, 7642849, 7642847, 7642848, 8881840, 7707809, 7477169, 7808487, 7646670, 7629982, 7642860, 7746058, 7830728, 7898178, 7657279, 8520744, 7648654, 8582390, 7594019, 7796496, 7664425, 7586343, 7574193, 7848024, 7666093, 7843710, 7795772, 7697252, 7812559, 7616798, 7822661, 7658483, 7658482, 7533825, 7580638, 7869560, 7897117, 7737631, 7722114, 7848120, 7751511, 7531732, 7664863, 7674504, 7759725, 7615288, 7602352, 7619749, 7698703, 7797785, 7557868, 7799026, 8746907, 7729302, 7595712, 7602351, 7751901, 7622763, 7487244, 7664856, 7697242, 7791257, 7595698, 7496281, 7698589, 7897786, 7985892, 8720288, 7751891, 7661492, 7791814, 7738628, 7636050, 7560477, 8586132, 7539233, 7897108, 7795774, 8575495, 7828296, 7587856, 7898521, 7595704, 7697845, 7768494, 7555472, 7589374, 7729018, 7729019, 7636054, 7608448, 8636523, 8582387, 8555803, 7751881, 7586339, 7541660, 7875472, 7632972, 7642857, 7810935, 7557093, 7842204, 7881645, 7588925, 7582300, 7666090, 7672690, 7711623, 7759696, 7890238, 7671347, 7867179, 7829793, 7850943, 7560618, 7767419, 7560617, 7542021, 7697275, 7884950, 7895002, 7560663, 7560662, 7615269, 7828316, 7877646, 7767538, 7799030, 7748020, 7759715, 7767525, 8536777, 7812004, 8543750, 7838188, 7840362, 7677296, 7555486, 7797125, 7850941, 7829780, 7722137, 7608434, 7833755, 8520339, 7594031, 7717597, 8595479, 8682006, 7805235, 7586313, 7710545, 7733838, 7702445, 7729633, 7640544, 7726322, 7664433, 7755127, 7793450, 7707121, 7602342, 7802127, 7560625, 7564670, 7743615, 7658819, 7528712, 7778957, 7786823, 7864267, 7666075, 7704169, 7875477, 7663802, 7499684, 7663801, 7735582, 7698688, 7898228, 8520793, 7755228, 7677827, 7789477, 7860905, 7663799, 7473835, 7557571, 7726308, 7551371, 8612440, 7805231, 7608485, 7590426, 7599807, 7759712, 7694914, 7475819, 7537116, 7595716, 7715295, 7737560, 7829789, 7503838, 7613277, 7715639, 7648653, 7767551, 7594041, 7852667, 7664861, 7853952, 7738626, 7697236, 7694913, 7477143, 7666103, 7737228, 7637722, 7633746, 7789488, 8575573, 7560661, 7698575, 7897151, 7586303, 8586126, 7698050, 7767531, 7654110, 7694917, 7633545, 7475592, 7722124, 7575051, 8612437, 7758568, 7551370, 7887757, 7702230, 7587848, 7654129, 8682007, 7564674, 7601400, 8636519, 7586338, 8586009, 7582291, 7599825, 7671364, 7707420, 7790604, 8521898, 7608438, 7743614, 8665975, 7743619, 7500507, 7486485, 7598296, 7633695, 7735607, 7744192, 7741366, 7845114, 7477146, 7767193, 7759705, 8549941, 7677298, 8881860, 7544732, 7603218, 7555507, 8568984, 7560608, 7588895, 7664435, 7884952, 7677557, 7595744, 7789484, 7595717, 8549952, 7623902, 7771914, 7500752, 7842186, 7637479, 7738618, 8522669, 7805238, 7722140, 7890212, 7887759, 7985893, 7499675, 7656906, 7575066, 7666085, 7895345, 7657105, 7494233, 8521562, 7594035, 7707626, 7658874, 8521551, 7758184, 7489796, 7582304, 7815886, 7557846, 8521893, 7594100, 7555530, 7599840, 7650334, 7763203, 7654128, 7738622, 7499781, 7778840, 7898227, 7810934, 7555553, 8881845, 7723797, 7595735, 8624292, 8521886, 7615283, 7847642, 7739706, 7726317, 7582278, 8682023, 7798505, 7625467, 8624293, 8682011, 7625474, 7744190, 7593899, 8750162, 8520738, 7845026, 7799013, 7795451, 7538555, 7760868, 7767512, 8555805, 7815656, 7560628, 7540062, 7538556, 7715641, 7565947, 7744189, 7595697, 7664857, 7563559, 7641350, 7700286, 7729296, 7697840, 7726306, 7593897, 7642863, 7767539, 7530507, 7544759, 7490991, 7778963, 7823670, 7658778, 7666089, 7723546, 7797777, 7531223, 7842192, 8636525, 7601182, 7475282, 7658780, 7802104, 7695460, 7677554, 7750526, 7540696, 7544368, 7637747, 8522666, 7580706, 7573591, 7496234, 7797783, 7885426, 7705775, 8847426, 7550814, 7564786, 7656936, 7633717, 7664860, 7589376, 7799699, 7656935, 7780131, 7545133, 7586348, 7759711, 7823668, 7582281, 7869554, 8583134, 8682086, 7843700, 7806038, 7669711, 7669712, 8523058, 7850984, 7867175, 7717598, 7794044, 7672677, 7664428, 7862180, 7563451, 7751885, 7723402, 7799707, 7575052, 7661937, 7637723, 7671358, 7802126, 7540996, 7574219, 7793451, 7832598, 7564509, 7887549, 7541448, 7602344, 7833483, 7844595, 7733839, 7985890, 7798528, 7895348, 7603217, 8520751, 7657286, 7882475, 8530808, 7833735, 7743622, 7715343, 7697842, 7633711, 7542675, 7828295, 8520759, 7477193, 7608429, 7666088, 7864705, 7661729, 7812552, 8721941, 7560489, 7663789, 7697277, 7598297, 7815657, 7798508, 7835575, 7849885, 8620970, 7816064, 7848119, 7872882, 7993000, 7625622, 7737555, 7611589, 7799016, 7864266, 7758177, 7655889, 7837862, 7768380, 8526243, 7697843, 7528271, 7632121, 7555487, 7798511, 7651473, 7586252, 7881669, 7633693, 7633704, 7697245, 7615287, 7588920, 7799029, 7848124, 7707423, 7717792, 7759706, 7806173, 7651472, 7799018, 7595718, 7853417, 7806146, 7737552, 7489931, 7744191, 7697844, 7594098, 8536780, 7895344, 7776995, 7885123, 8529732, 7637721, 7538285, 7560599, 7666101, 7787537, 7822659, 8620951, 7790606, 7735583, 7602349, 8589023, 7555554, 7707112, 7697233, 7595749, 7560619, 7474246, 7595746, 7663782, 8589028, 7799021, 7751898, 7751890, 7751875, 7595709, 7751888, 7541003, 7890239, 7722116, 7722161, 7555557, 7551381, 7752752, 7885125, 7864265, 7723496, 7671893, 7495344, 7575103, 8682010, 8682000, 7670117, 7890211, 7601430, 7751894, 7653695, 7554206, 7663809, 7565999, 7528310, 7633535, 7594023, 7641351, 7882472, 7829790, 7793470, 7798494, 7762909, 7557850, 7665863, 7541358, 7760608, 7566020, 7783536, 7636533, 7594088, 7744150, 7640591, 7640592, 7751527, 7897158, 7602359, 7738625, 7658777, 7560615, 7586247, 7487242, 7698046, 7707405, 7616634, 7615189, 7755224, 7594063, 8575497, 8551824, 7861873, 7789486, 8575575, 7788917, 7498641, 7653685, 7862181, 7536028, 7812574, 7897161, 7707407, 8750152, 8551821, 7549636, 7728058, 7654268, 7498216, 7640538, 7760614, 7603215, 7891484, 7707126, 7595707, 7595708, 7580663, 7873952, 7833752, 7897784, 7580639, 7861879, 7491044, 7613410, 7595729, 7697841, 7580551, 7666098, 7799011, 7602354, 7844610, 7475658, 7727768, 7697849, 7657272, 7473820, 7884414, 7564784, 7844607, 7844608, 7776772, 7797776, 7473832, 7666094, 7888933, 7751897, 7586312, 7623530, 7763202, 7752258, 7540705, 7767523, 7595738, 7759724, 7477145, 7594097, 7589382, 7799046, 7884433, 7489965, 8541533, 7867177, 8620950, 7491136, 7752754, 8682016, 7566021, 7767541, 7551366, 7622762, 7634447, 7722117, 7797790, 7840365, 7489976, 7722120, 7543344, 8722072, 7665750, 7654109, 8541750, 7670096, 7666097, 7755126, 7671894, 7486483, 7489937, 7828294, 8523056, 7707099, 8589013, 7751874, 7715294, 7719181, 8682031, 7715293, 7767515, 8575574, 7636529, 7737638, 7623904, 7543434, 7545059, 7759701, 7881655, 7875466, 7551400, 7835571, 7837388, 7776501, 7653696, 7737632, 7475716, 7864704, 7707108, 7895357, 7554199, 7767194, 7582277, 7594045, 7853949, 7671361, 7664854, 7842199, 7843703, 7647644, 7586262, 7751513, 7797748, 7594026, 7586246, 7474192, 7551385, 9110940, 7760888, 7711582, 7799019, 7751882, 7635420, 7540071, 8523053, 7602364, 7897109, 8536776, 7758179, 7811163, 7787595, 7767427, 7557866, 7797786, 7498201, 7789487, 7830729, 7845426, 7791815, 8720287, 7822663, 8520750, 7990904, 7629954, 7564725, 7666874, 7842184, 7748048, 7842195, 7657130, 7768495, 7758556, 7656958, 7799033, 8682090, 8575493, 7609260, 7742677, 7654113, 7744136, 7767514, 7594092, 8682088, 7625470, 7767416, 8543748, 7589375, 7582283, 7872876, 8520743, 7599850, 7560622, 7884423, 7823996, 7722163, 7852666, 7849886, 7798503, 7616802, 7608436, 7537110, 7637478, 7707111, 7477166, 7897129, 8575576, 7497865, 7697239, 8636524, 8589027, 7595711, 7665860, 7885427, 7756663, 7723158, 7536871, 8682002, 7594099, 8542889, 8666093, 7812579, 7768497, 7657122, 7555484, 7698708, 7726307, 7601403, 7639438, 7661494, 7589392, 7643883, 7477218, 8526244, 7555478, 7842205, 7887554, 7882486, 7744151, 7599866, 7598298, 8624301, 7842185, 7760609, 7488893, 7737655, 7697230, 7602060, 7580666, 7705801, 7745771, 7563558, 7799040, 7587846, 7627043, 7575227, 8750168, 7769899, 7698030, 7537797, 7726705, 7856987, 7651474, 8917264, 8879370, 8944560, 8638999, 8742572, 8973228, 8532025, 8765815, 8703169, 8594425, 8672151, 8778585, 8732710, 8781824, 8676931, 8793447, 8624183, 8538705, 8717471, 8951297, 8621850, 8538704, 8842603, 8866594, 8639069, 8561204, 8911223, 8598592, 8940320, 9007710, 8694673, 8828533, 9006746, 8857869, 8630576, 8621174, 8876546, 8757209, 8756801, 8792754, 8839908, 8642852, 8821690, 8938163, 8649495, 8683634, 8595286, 8973231, 8879372, 8555849, 8839900, 8801202, 8905028, 8598866, 8548891, 8855175, 8880241, 8874180, 8839911, 8605328, 8611684, 8634416, 8777169, 8903382, 9037359, 8663871, 8839839, 8626947, 8634730, 8622092, 8839906, 8626981, 8813038, 8649492, 8839865, 8939149, 8565164, 8908387, 8675164, 8702088, 8819441, 8941460, 8648356, 8558219, 8839902, 8558217, 8791049, 8648368, 8896392, 8622041, 8827017, 8905421, 8665055, 8618579, 8873656, 8774204, 8634560, 8946925, 8558939, 8907566, 8901854, 8821692, 8622055, 8912772, 8683243, 8733086, 8962558, 8886549, 8752791, 8970342, 8892674, 8622045, 8683230, 8622389, 8917263, 8962549, 8869860, 8759064, 8636753, 8620234, 8737216, 8970358, 8837563, 8759066, 8905434, 8774203, 8890679, 8901659, 8949637, 8836654, 8566840, 8538349, 8840841, 8684102, 8828539, 8781317, 8618581, 8810613, 8610947, 8881809, 8759069, 8880096, 8564101, 8813983, 8688776, 8725871, 8881815, 8707089, 8664609, 8967706, 8836653, 8666352, 8733437, 8781825, 7503472, 8857016, 8636546, 8594265, 8653874, 8941105, 8941106, 8943865, 8604962, 8712867, 8772687, 8887586, 8804929, 8898646, 8626941, 8791042, 8879373, 8656243, 8839903, 8956674, 8635258, 8639015, 8615705, 8742566, 8970354, 8905437, 8624220, 8814468, 8684103, 8555852, 8695837, 8557899, 8905026, 8621847, 8756806, 8676626, 8630590, 8880109, 8941458, 8956701, 8566836, 8977503, 8976635, 8901660, 8866596, 8569363, 8941477, 8774567, 8778601, 8953998, 8618369, 8610948, 8613630, 8598594, 8555470, 8611831, 8793448, 8727433, 8646314, 8687263, 8622072, 8940325, 8733090, 8557891, 8942719, 8857018, 8756814, 8609325, 8622327, 8639012, 8565166, 8634616, 8542129, 8797769, 8611877, 7494563, 8558203, 8916975, 8636748, 8678176, 8678177, 8687515, 8823335, 8941114, 8709687, 8890680, 8925577, 8610833, 8790024, 8633697, 8759075, 8630585, 8557897, 8921778, 8800113, 8694682, 8618584, 8844228, 8630595, 8752793, 8646142, 8725851, 8648369, 8704533, 8531541, 8942464, 8568124, 8684155, 8806290, 8898643, 8564089, 8941104, 8708713, 8708714, 8757207, 8912488, 8772681, 8620236, 8890801, 8648387, 8880107, 8622022, 8707080, 8925581, 8813041, 8628335, 8967704, 8929262, 8859144, 8866584, 8815751, 8968014, 8691967, 8953999, 8597736, 8564107, 8596222, 8702086, 8961992, 8596381, 8665036, 8821686, 8675093, 8962564, 8918858, 8793451, 8834029, 8690200, 8774205, 8880110, 8526698, 8564129, 8984939, 8939178, 8730003, 8880102, 8688760, 8892717, 8772751, 8921775, 8558225, 8790074, 8596594, 8678934, 8598621, 8859063, 8912148, 8675159, 8594266, 8665047, 8616303, 8948561, 8610914, 8636558, 8678385, 8732700, 8967707, 8931612, 8640968, 8842601, 8602180, 8918852, 8557900, 8781823, 8834030, 8740782, 8606384, 8940324, 8684101, 8642959, 8737105, 8640980, 8909907, 8772679, 8675080, 8986484, 8640997, 8562960, 8569016, 8912734, 8880018, 8901655, 8554246, 8738728, 8778198, 8941092, 8648360, 8682134, 8828530, 8621845, 8837569, 8772759, 8800119, 8709402, 8967668, 8540776, 8839837, 8971064, 8790131, 8866593, 8718436, 8911222, 8869864, 8613058, 8531308, 8596569, 8840852, 8912765, 8607723, 8688774, 8564134, 8678182, 8718440, 8752805, 8738724, 8765813, 8965890, 8559201, 8572841, 8620226, 8973433, 8610836, 8667868, 8616576, 8680689, 8657212, 8780566, 8938192, 8765824, 8630596, 8640987, 8683232, 8704237, 8886552, 8622048, 8977350, 8980985, 8941467, 8869876, 8921786, 8633818, 8561215, 8678175, 8618582, 8629878, 8759084, 8691923, 8769587, 9006748, 8839901, 8908382, 8840843, 8901851, 8609658, 8895005, 8964406, 8898629, 8676619, 8874327, 8942462, 8947070, 8630574, 8887604, 8678393, 8659620, 8786777, 8678170, 8908379, 8725855, 8875098, 8683249, 8633830, 8596268, 8938531, 8616543, 8989127, 8938545, 8707278, 8890674, 8636768, 8737101, 8649056, 8636743, 8790025, 8630607, 8733444, 8822914, 8648358, 8842608, 8637342, 8611060, 8622502, 8861991, 8726925, 8622020, 8918486, 8923079, 8780630, 8960472, 8707264, 8572836, 8626963, 8757208, 8664720, 9120105, 8800106, 8896396, 8837559, 8757204, 8874334, 8626983, 8598837, 8805732, 8923073, 8840849, 8630579, 8742561, 8947088, 9038665, 8630581, 8542158, 8564100, 8630575, 8648870, 8941480, 8780431, 8665039, 8941095, 8790035, 8608290, 8558223, 8923090, 8756812, 8876562, 8712886, 8774159, 8566580, 8931970, 8673189, 8621843, 8656545, 8960474, 8522712, 8609324, 8813271, 8887580, 8608285, 8621147, 8694672, 8602179, 8531998, 8546552, 8686973, 8616947, 8656441, 8905031, 8732376, 8626939, 8635262, 8941101, 8607727, 8653868, 8859136, 8683231, 8704887, 8568135, 8646094, 8890677, 8908381, 8616547, 8622245, 8898645, 8606267, 8572838, 8622034, 8598695, 8648371, 8572837, 8962554, 8707096, 8660109, 8980974, 8917255, 8730004, 8703168, 8596317, 8616946, 8708719, 8666320, 8550052, 8563585, 8792753, 8639017, 8609686, 8965861, 8683244, 8944004, 8834027, 8608877, 8675100, 8908377, 8665038, 8613046, 8815753, 8569362, 8606276, 8922196, 8648018, 8708716, 8964113, 8594267, 8737211, 8708166, 8836663, 8780429, 8921795, 8955656, 8656254, 8791050, 8905424, 8628357, 8633815, 8737099, 8804944, 8989129, 8947084, 8791051, 8836642, 8566861, 8757203, 8822974, 8522716, 8740792, 8536887, 8964395, 8618603, 8879380, 8542008, 8655877, 8558222, 8598593, 8634728, 9120111, 8708171, 8626978, 8912740, 8566592, 8667538, 8630564, 8887606, 8726951, 8929263, 8606715, 8636540, 8955653, 8656678, 8555493, 8912147, 8890804, 8874326, 8627643, 8938176, 8938179, 8779455, 8822986, 8640969, 8813270, 8790023, 8961972, 8526700, 8905428, 8606613, 8622076, 8842602, 8598090, 8752795, 8665064, 8656680, 8564096, 9007706, 7494588, 8596376, 8663853, 8614421, 8563533, 8628336, 8607589, 8916958, 8547651, 8772689, 8800102, 8800103, 8752790, 8621136, 8616937, 8609343, 8887578, 8676932, 8738729, 8991863, 8822997, 8704534, 8630380, 8626951, 8780576, 8564099, 9120123, 8978226, 8608288, 8627642, 8674171, 8874325, 8828525, 8686975, 8613615, 8978218, 8656444, 8559203, 8601999, 8605463, 8826808, 8709688, 8709734, 8806289, 8918277, 8898601, 8942775, 8620132, 8609746, 8782754, 8888166, 8622332, 8950882, 8709736, 8806288, 8688757, 8598699, 8855856, 8813987, 8616414, 8942774, 8611783, 8950879, 8937280, 8598755, 8538345, 8598700, 8950881, 8973428, 8544547, 8531545, 8611872, 8973430, 8684197, 8637340, 8760738, 8622391, 8606612, 8931610, 8790022, 8566601, 8558188, 8918495, 8977984, 8558208, 8708728, 8636779, 8815853, 8922195, 8708718, 8831595, 8708726, 8874336, 8622038, 8636745, 8874339, 8558199, 8831596, 8606262, 8925582, 8683240, 8537982, 8684201, 8908384, 8942777, 8542165, 8592524, 8869862, 8639883, 8967667, 8967666, 8873662, 8778189, 8866582, 8890678, 8904862, 8597317, 8840836, 8694680, 8653870, 8967669, 8633687, 8918498, 8921782, 8796387, 8709689, 8565153, 8917260, 8942454, 8903371, 8522681, 8606561, 11823998, 8923071, 8977237, 8938532, 8901853, 8684199, 8956699, 8740786, 8912486, 8566605, 9081395, 8823332, 8916695, 8707128, 8811760, 8656503, 8656502, 8544269, 8678389, 8622225, 8862101, 8759983, 8756833, 8568137, 8977514, 8902473, 8866595, 8707127, 8630539, 8683228, 8540598, 8921777, 8831438, 8622042, 8542001, 8756815, 8595287, 8687262, 8565163, 9007717, 8611058, 8780417, 8880031, 8973432, 8664717, 8917256, 8805730, 8752794, 8542136, 8603220, 8805928, 7503497, 8630559, 8964410, 8683251, 8624184, 8773636, 8855179, 8635265, 8647140, 8622050, 8772758, 8639011, 8908380, 8873668, 8960447, 8609337, 8727432, 8675160, 8892714, 8970355, 8908378, 8740787, 8614419, 8842609, 8757206, 8777954, 8616558, 8621132, 8737213, 8875083, 8542163, 8801446, 8542164, 8793454, 8790077, 8905024, 8633829, 8782638, 8680672, 8611064, 8639070, 8684198, 8792758, 8720537, 8708167, 8621857, 8857005, 8922914, 8637535, 8526696, 8684407, 8554205, 8651843, 8732386, 8844237, 8630614, 8931972, 8611059, 8618367, 8923067, 8977062, 8922912, 8554251, 8813039, 8791047, 8781321, 8621137, 8943160, 8614420, 8682138, 8596231, 8648019, 8757200, 8986493, 8634735, 8622066, 8609339, 8938538, 8909381, 8558194, 8550048, 8630624, 8636549, 8652810, 8813984, 8977510, 8646143, 8682116, 8598756, 8773908, 8857435, 8608893, 8878773, 8557919, 8857868, 8970345, 8836892, 8628358, 8598838, 8806245, 8922197, 8636761, 8609945, 8637514, 8594426, 8831436, 8831437, 8626986, 8637536, 8546549, 8558187, 8942455, 8828528, 8554206, 8908388, 8675168, 8675165, 8729158, 8912492, 8732374, 8542002, 8554248, 8708717, 8597320, 8752803, 8916749, 8813040, 8622226, 8678384, 9358213, 9296229, 9294380, 9173270, 9099655, 9382660, 9385125, 9295240, 9340504, 9062089, 9311358, 9183229, 9358138, 9278462, 9250846, 9091801, 9358126, 9337378, 9402455, 9389286, 9099116, 9233870, 9288004, 9011784, 9233868, 9110909, 9287227, 9037645, 9302957, 9230252, 9314339, 9152656, 9236562, 9366660, 9096985, 9160657, 9391254, 9296217, 9362378, 9343463, 9402173, 9247455, 9373242, 9096584, 9402453, 9180105, 9180103, 9284664, 9040328, 9233326, 8988115, 9328304, 9137223, 9412795, 9232045, 9314625, 9130688, 9496386, 8980206, 9153366, 9362350, 9268276, 9129894, 9366661, 9235493, 9209661, 9096687, 9398007, 9215245, 9389686, 9097988, 9321530, 9295241, 9006528, 9167106, 9110907, 9295239, 9041232, 9003206, 9051366, 9314127, 9150326, 9129038, 9060898, 9196140, 9286191, 9402450, 9042638, 9093709, 9215818, 9164215, 9309100, 9412558, 9168289, 9250445, 9155575, 9192919, 9321529, 9164811, 9351617, 9150318, 9192926, 9389288, 9120173, 9062091, 9316514, 8996294, 9193243, 9416896, 9120155, 9250230, 9307346, 9315764, 9337187, 9247510, 9180113, 9076381, 9286179, 9286194, 9049526, 9355902, 9329515, 9264477, 9269786, 9192924, 9133513, 9247512, 9355890, 9105094, 9230243, 9323063, 9002942, 9458425, 9350914, 9236433, 9054757, 9060555, 9352888, 9303505, 9103344, 9207647, 9060533, 9135227, 9350925, 9286184, 9111435, 9076466, 9310811, 9040574, 9105072, 9091691, 9283506, 9193210, 9361543, 9093710, 9193323, 9396419, 9009975, 9027270, 9180087, 9290542, 9233865, 9080938, 9215249, 9314346, 9265425, 9060896, 9182470, 9193325, 9219702, 8996152, 9057634, 9203932, 9402446, 9096989, 9416884, 9028694, 9207470, 9397979, 9400342, 9054279, 9230725, 9350935, 9264489, 9054791, 9142004, 8978338, 9054741, 9196086, 9264494, 9135928, 9316528, 9272926, 9458423, 9193328, 9060552, 9005747, 9405898, 9493656, 9376578, 9236570, 9164809, 9385891, 9426082, 9054795, 9236418, 9083230, 9022435, 9022434, 9158469, 9417009, 9412548, 9042043, 9117023, 9363872, 9032502, 9164216, 9250182, 9049193, 8996149, 9351621, 8998184, 9055716, 9167505, 9129896, 9182975, 9014973, 8996291, 9294478, 9161310, 9054781, 9428251, 9388086, 9372670, 9236451, 9040570, 9059953, 9164200, 9160697, 9164318, 9049196, 9264478, 9069004, 9350938, 9193324, 9006398, 9203448, 9353612, 9385883, 9389306, 9060881, 9386141, 8996417, 9426081, 9142006, 9286937, 9365037, 9163650, 9075669, 9032494, 9274477, 9167507, 9385303, 9274470, 9389293, 9129047, 8996142, 9215819, 9164221, 9200635, 9351634, 9196141, 9203934, 9054788, 9310603, 9111538, 9196163, 9283510, 9093724, 9269788, 9051390, 9015001, 9388396, 8995085, 9193433, 8996118, 9387942, 9042633, 9051367, 9101212, 9388088, 9071936, 9278463, 9301505, 9008444, 9051387, 9228961, 9283534, 9036799, 9413466, 9227167, 9105077, 9133516, 9412304, 9322500, 9120151, 9180124, 9073002, 9353508, 9358137, 9001307, 9176069, 9167559, 9096993, 9458419, 9372677, 9396433, 9057731, 9291905, 8988956, 9316510, 9167520, 9215816, 9049514, 9164214, 9412549, 9310023, 9163637, 9230234, 9196087, 9133508, 9274466, 9236571, 9416888, 9347278, 9264493, 8994421, 9392698, 9120179, 9008458, 9426097, 9314638, 9168290, 9316509, 9075649, 9027267, 9086005, 9230719, 9345021, 9356001, 9412546, 9314634, 9043840, 9412550, 9096977, 9355889, 8996307, 9051395, 9120161, 9098014, 9150334, 9041098, 9470077, 9493667, 9196108, 9316524, 9075462, 9352866, 9042628, 9118516, 9107154, 9311486, 9315535, 9236443, 9010146, 9385122, 9244210, 9266231, 9493655, 8970936, 9051389, 9109749, 9345173, 9110906, 9438487, 9314641, 9076465, 9302301, 9414975, 9352869, 9531235, 9338537, 9232040, 9341056, 9309988, 9355894, 9091692, 9236561, 9155573, 9337376, 9096986, 9256139, 9244203, 9396960, 9141424, 9315539, 9470081, 9352867, 9142587, 9163286, 9186834, 9185751, 8985267, 9279213, 9236425, 9257791, 9345020, 9168185, 9032543, 9167113, 9392696, 9171065, 9271481, 9207281, 9154031, 9192927, 9133884, 9345174, 9350924, 8996134, 9250456, 9405902, 9207636, 9252087, 9369422, 9338554, 9279224, 9152658, 9032190, 9366659, 9178671, 9006472, 9028341, 9170400, 9236420, 9106647, 9311511, 9203950, 9060527, 9400343, 9283504, 9412794, 9338539, 9105061, 9040326, 9167457, 9322512, 9244208, 9313019, 9438485, 9203944, 9201522, 9164223, 8996292, 9283792, 9167112, 9405910, 9261526, 9226158, 9060560, 9187067, 9193361, 9382964, 9352864, 9167105, 9137213, 9310806, 8985273, 9118762, 9314121, 9365036, 9323056, 9126791, 9093726, 9113932, 9028311, 9329513, 9150331, 9193192, 8996144, 9262240, 9232049, 9187068, 9256140, 9385910, 9054841, 9137231, 9040320, 9059944, 9236426, 9309995, 9118772, 9200633, 9164193, 9137798, 9233866, 9107182, 9310813, 9196156, 9374887, 9402779, 9164196, 9250453, 9027276, 9077376, 9032510, 9207639, 9083228, 9307357, 9154850, 9426083, 9196111, 9032045, 9001297, 9230720, 9252131, 9287967, 9155828, 9196083, 9361576, 9060525, 9072981, 9314644, 9135961, 9040329, 9215828, 9227927, 9108387, 9133510, 9255651, 9241127, 9308510, 9361539, 9001327, 9036797, 9014993, 9148651, 9154869, 9387944, 9227164, 9247524, 9060551, 8996129, 9387952, 9247406, 8985298, 9322520, 9227926, 9120160, 9105060, 9041233, 9028689, 9059138, 9090346, 9274547, 9126802, 9247511, 9363971, 9272895, 9332684, 9215826, 9076457, 9652562, 9352870, 9256222, 9294377, 9187435, 9049515, 9171066, 9531230, 9053465, 9215844, 9142590, 9272925, 9247517, 9133509, 9310809, 9118763, 9183237, 9279225, 8988886, 9093249, 9090340, 9294477, 9182474, 9215817, 9332682, 9182472, 9353602, 9250454, 9470082, 9390050, 9400511, 9051392, 8980207, 9314631, 9269759, 9182469, 9396958, 9243145, 9028693, 9252084, 9356566, 9247454, 9337185, 9283509, 9389292, 9230767, 9294378, 9210608, 9396394, 9311490, 9279219, 9275135, 9230237, 9090332, 9152114, 8996143, 9426079, 9405909, 9117021, 9107166, 9314645, 9187908, 9283511, 9041875, 9395429, 9009974, 9105070, 9343462, 9272896, 9090331, 9462204, 9309980, 9116551, 9301500, 9040303, 9322521, 9103129, 9493664, 9153367, 9458416, 9054848, 9214246, 9347370, 9187443, 9370502, 9134943, 9163658, 9136830, 9302302, 9180111, 9087467, 9103126, 9187434, 8996159, 8996137, 9196161, 9060567, 9256141, 9256142, 9060535, 9060541, 9337186, 9305603, 9286186, 9060568, 9193426, 9387969, 9022429, 9279217, 9107153, 9075465, 9347368, 9395428, 9137794, 9184580, 9214247, 9207646, 9323077, 9402168, 9247476, 9219700, 9043841, 9400034, 9250457, 9164228, 9265424, 9218667, 9337181, 9078199, 9042042, 9283508, 9241125, 9398000, 9164203, 9416883, 9337190, 8996153, 9247506, 9438490, 9252074, 9060894, 8988955, 9371853, 9169397, 9053491, 9362356, 9126795, 9024142, 9385898, 9363861, 9257790, 9154884, 9361574, 9032500, 9247526, 9137123, 9462166, 9358933, 9105068, 9350912, 9275141, 9193196, 9135526, 9158465, 9251634, 9001476, 9048792, 9400510, 9284777, 9366737, 9113011, 9022487, 9048788, 9224079, 9180065, 9048790, 9357405, 9428250, 9393335, 9366727, 9274549, 9055715, 9074573, 9297994, 9142061, 9186385, 9113009, 9291901, 9274584, 9310604, 9652560, 10213547, 9310602, 9057729, 9652561, 9100622, 9284774, 9291904, 9193380, 9652559, 9078197, 9074574, 9228962, 9413463, 9113010, 9365449, 9329511, 9130939, 9274583, 9024371, 9074572, 9357406, 9402774, 9093250, 9365450, 9078198, 9259655, 9291903, 9297998, 9107241, 9314869, 9388397, 9133891, 9251545, 9329303, 9174562, 9093725, 9236422, 9116274, 9120174, 9267791, 9215845, 9060545, 9363868, 9043839, 9209440, 9294465, 9256118, 9060557, 9332680, 8996151, 9053508, 9137222, 9256127, 9207435, 9164194, 9060957, 9363869, 8996138, 9215837, 9196144, 9316525, 9382669, 9164179, 9166835, 9129014, 9337184, 9193370, 9142008, 9355892, 9301496, 9054789, 9203933, 9270458, 9129016, 9405901, 9405913, 9245924, 9416886, 9152099, 9093253, 9531224, 9338535, 9193208, 9496390, 9080918, 9247389, 9083239, 9434916, 9244213, 8990337, 9405908, 9042045, 9292541, 9470078, 9394721, 9137791, 9152646, 9060534, 9207618, 9152654, 9201523, 9136839, 9310807, 9259653, 9058684, 9236555, 9176076, 9230003, 9386146, 9257783, 9366582, 9230238, 9193290, 9315523, 9054840, 9261520, 9201012, 9046892, 9149711, 9416890, 9164180, 9287229, 9396449, 9243149, 9342998, 9116271, 9051391, 9040296, 9060538, 9090338, 9286938, 9387945, 9236446, 9307345, 9230722, 9390536, 9372661, 8999265, 9154767, 9252088, 9332683, 9341573, 9010144, 9310011, 9310010, 9117020, 9196082, 9250849, 9099660, 9262495, 9032509, 9105062, 9091670, 9438480, 9032515, 9250226, 9043838, 9117012, 9058681, 9032501, 9193198, 9070471, 9072985, 9135927, 9358940, 9210750, 8980210, 9426331, 9073005, 9361572, 9167099, 9279218, 9412547, 9185746, 9215242, 9283507, 9250233, 9362354, 9118764, 9412301, 9093728, 9051365, 9407153, 9059137, 9032531, 9437275, 9126796, 9185752, 9313020, 9356557, 9356558, 9217714, 9152116, 9058689, 9075670, 9252161, 9176086, 9196130, 9400355, 9192929, 9054280, 9163285, 9352865, 9164377, 9193364, 9207282, 9345074, 9303508, 9021962, 9203426, 9211677, 9283776, 9224082, 9412311, 8970933, 9351614, 9345019, 9116275, 9372647, 9040295, 9219699, 9020273, 9139555, 9023090, 9531231, 9332685, 9287228, 9211675, 9154882, 8996293, 9135531, 9313000, 8994413, 9142578, 9369411, 9266230, 9453428, 9041099, 9142583, 9257795, 9283530, 9376575, 9187896, 9036801, 9326833, 9396403, 9347277, 9059950, 9328328, 9134944, 9164205, 9139554, 9356569, 9210746, 9284668, 9182213, 9659858, 9932161, 9462523, 9473217, 9581713, 9834303, 9459643, 9701099, 9761802, 9599104, 9435326, 9691103, 9738086, 9709042, 9700176, 9494148, 9761803, 9545358, 9637805, 9828247, 9504939, 9845708, 9709043, 9783557, 9854116, 9643668, 9572995, 9716578, 9672054, 9508182, 9545359, 9564793, 9740569, 9654535, 9541686, 9476862, 9551672, 9700123, 9857924, 9531581, 9609749, 9679847, 9570178, 9505879, 9437379, 9676408, 9741499, 9440664, 9596598, 9794856, 9862051, 9448552, 9716583, 9740347, 9701130, 9802740, 9581698, 9555553, 9476870, 9778204, 9791142, 9701104, 9544767, 9723669, 9812111, 9802373, 9862047, 9746769, 9673299, 9827783, 9521334, 9777836, 9605896, 9653611, 9809934, 9554677, 9504513, 9820261, 9799205, 9727545, 9773720, 9586883, 9609408, 9655732, 9676671, 9839521, 9552047, 9704725, 9734883, 9697878, 9809958, 9777834, 9449495, 9777817, 9517592, 9682041, 9471927, 9655713, 9616311, 9561981, 9653595, 9731088, 9519774, 9864001, 9521221, 9697880, 9537847, 9680346, 9495294, 9822086, 9468465, 9865797, 9690406, 9655734, 9462588, 9564795, 9609411, 9552906, 9498533, 9556464, 9737514, 9704239, 9568442, 9808595, 9799202, 9741504, 9862944, 9741514, 9751681, 9822094, 9614257, 9428213, 9717065, 9552949, 9850026, 9653594, 9863850, 9787142, 9494026, 9824265, 9589245, 9811916, 9563738, 9525449, 9828235, 9751675, 9761805, 9609412, 9776411, 9833910, 9783558, 9863994, 9701432, 9648959, 9648960, 9564797, 9721148, 9753485, 9676677, 9886723, 9817266, 9843456, 9571357, 9541692, 10326538, 9600501, 9681774, 9589246, 9552031, 9647875, 9834301, 9869669, 9593789, 9500689, 9440719, 9540025, 9786811, 9715861, 9626844, 9551737, 9864004, 9552063, 9741505, 9625686, 9862943, 9464200, 9459471, 9589236, 9786808, 9862558, 9655733, 9672165, 9850014, 9537454, 9570192, 9552064, 9555556, 9562007, 9577354, 9797393, 9665188, 9440748, 9768574, 9585709, 9531251, 9779696, 9589247, 9450712, 9581739, 9469367, 9847429, 9778203, 9822097, 9502647, 9443427, 9491040, 9704737, 9667257, 9529260, 9681082, 9834279, 9468466, 9581743, 9857862, 9834304, 9582132, 9570170, 9596049, 9672056, 9581674, 9605644, 9538976, 9561984, 9610529, 9501711, 10070271, 9701431, 9552059, 9510219, 9462573, 9743440, 9817163, 9609084, 9702441, 9855386, 9820992, 9435325, 9860785, 9841603, 9676682, 9645829, 9449500, 9768710, 9546003, 9426014, 9676668, 9672052, 9717036, 9877478, 9588436, 9679718, 9636831, 9719081, 9819068, 9554431, 9819291, 9779697, 9449871, 9734938, 9433336, 9667247, 9801184, 9620322, 9794929, 9711933, 9515999, 9746766, 9586877, 9591764, 9756807, 9626231, 9605803, 9634262, 9649456, 9616313, 9623685, 9449727, 9847430, 9464213, 9875874, 9719082, 9768596, 9872248, 9771399, 9773732, 9676666, 9791144, 9643792, 9701409, 9721113, 9773721, 9704236, 9551739, 9832483, 9773734, 9850017, 9641933, 9581728, 9669786, 9585710, 9641928, 9723660, 9536950, 9851955, 9581724, 9852885, 9577336, 9543266, 9566827, 9855387, 9518395, 9824602, 9433426, 9582043, 9728984, 9500317, 9664089, 9657561, 9589229, 9761804, 9817160, 9651717, 9766765, 9773716, 9529266, 9841599, 9669262, 9704717, 9669790, 9503217, 9679720, 9648963, 9839524, 9819288, 9518909, 9577350, 9508155, 9516220, 9839106, 9741525, 9521232, 9802752, 9517604, 9603112, 9676667, 9469324, 9625405, 9694707, 9731025, 9585725, 9441569, 9804720, 9551726, 9712594, 9657569, 9596112, 9605777, 9597417, 9652995, 9525445, 9464212, 9771208, 9647874, 9445291, 9701429, 9657576, 9469353, 9539069, 9833917, 9832490, 9635947, 9802367, 9538979, 9603532, 9494025, 9657565, 9640129, 9564804, 9886730, 9571355, 9551748, 9731558, 9623694, 9626221, 9600478, 9564799, 9626821, 9566830, 9566835, 9731006, 9822096, 9768705, 9477931, 9633671, 9516221, 9589248, 9839094, 9839093, 9482363, 9819296, 9701421, 9433339, 9462572, 9651263, 9672169, 9801179, 9777816, 9581695, 9537342, 9758574, 9514542, 9503181, 9654538, 9672057, 9449498, 9469326, 9536937, 9747866, 9440758, 9824569, 9743505, 9571333, 9449494, 9449728, 9815865, 9492666, 9469323, 9839522, 9427706, 9717012, 9655746, 9657583, 9722212, 9768576, 9525448, 9593559, 9886725, 9571254, 9424977, 9862555, 9740348, 9508173, 9469362, 9468205, 9681077, 9679792, 10189849, 9665357, 9695999, 9842788, 9846777, 9846778, 9820262, 9811286, 9625398, 9803327, 9757919, 9440666, 10326533, 9571328, 9653596, 9773742, 9738560, 9641712, 9659862, 9433338, 9809729, 9529267, 9826308, 9566833, 9809953, 9537429, 9657119, 9857856, 9787148, 9817174, 9487169, 9510218, 9624074, 9565457, 9510221, 9702436, 9723661, 9632446, 9799203, 9571359, 9609409, 9691919, 9477929, 9519951, 9802364, 9708456, 9476845, 9462678, 9847435, 9802741, 9665186, 9857882, 9614617, 9517603, 9763554, 9740345, 9697820, 9762777, 9626770, 9424040, 9648697, 9832000, 9495299, 9707384, 9738637, 9462565, 9648966, 9554680, 9808602, 9437382, 9752816, 9812110, 9817153, 9517578, 9679845, 9730996, 9603127, 9603111, 9705684, 9721156, 9812933, 9596594, 9847269, 9667263, 9469359, 9863851, 9808599, 9558288, 9500717, 9819446, 9819447, 9820292, 9562580, 9508170, 9672274, 9428817, 9440749, 9770556, 9517609, 9620901, 9783563, 9741502, 9717924, 9583926, 9727896, 9721149, 9550954, 9716993, 9563981, 9839101, 9593411, 9862942, 9654537, 9834260, 9603794, 9469328, 9681078, 9694705, 9540015, 9771414, 9576425, 9840235, 9832481, 9566819, 9786373, 9469360, 9626172, 9702445, 9426022, 9736592, 9469347, 9626201, 9807988, 9540024, 9797360, 9562003, 9519318, 9616309, 9558411, 9494145, 9757853, 9850033, 9571346, 9628709, 9469327, 9877488, 9537453, 9791141, 9440721, 9831997, 9683207, 9665202, 9551752, 9586911, 9834207, 9519355, 9519356, 9555559, 9817708, 9554895, 9625397, 9659155, 9699707, 9454525, 9867443, 9537864, 9508168, 9589230, 9626829, 9851956, 9757855, 9414326, 9586892, 9555757, 9664090, 9625400, 9657560, 9508171, 9440718, 9817288, 9677215, 9691922, 9820259, 9653597, 9519948, 9820298, 9600500, 9802369, 9613912, 9727782, 9886714, 9529467, 9586884, 9818070, 9648957, 9773719, 9556465, 9490238, 9615209, 9764990, 9707377, 9786807, 9679794, 9708455, 9794903, 9659857, 9494149, 9824598, 9552948, 9738562, 9809947, 9734550, 9773735, 9653613, 9649455, 9586668, 9618650, 9778325, 9739972, 9625322, 9580646, 9841585, 9554444, 9885888, 9812120, 9571349, 9632444, 9700115, 9700132, 9469330, 9552068, 9834280, 9728642, 9738579, 9667262, 9462525, 9751674, 9591761, 9469350, 9852907, 9667249, 9552042, 9746768, 9626212, 9586887, 9508190, 9817278, 9845709, 9820990, 9505892, 9490235, 9439492, 9552905, 9596107, 9665902, 9440742, 9619146, 9809728, 9571344, 9570205, 9731027, 9614619, 9760291, 9635948, 9505889, 9508151, 9546566, 9433337, 9768715, 9872247, 9625399, 9647873, 9443925, 9672273, 9841303, 9802273, 9757854, 9508225, 9736008, 9490236, 9449497, 9809926, 9696733, 9613910, 9817690, 9557622, 9817281, 9738557, 9649466, 9748684, 9469333, 9425919, 9808550, 9778328, 9586886, 9440756, 9521168, 9875644, 9599191, 9586915, 9746023, 9545357, 9701410, 9790546, 9543270, 9794849, 9449870, 9643663, 9504514, 9716056, 9552954, 9728982, 9643685, 9843104, 9514531, 9624070, 9624068, 9624069, 9583927, 9482294, 9804712, 9759744, 9685273, 9657784, 9500318, 9831574, 9794859, 9546505, 9802271, 9552953, 9462315, 9433424, 9788453, 9660578, 9652613, 9504515, 9683204, 9492772, 9853438, 9457094, 9482293, 9798586, 9831573, 9774287, 9752814, 9605804, 9737282, 9734940, 9651260, 9716055, 9482291, 9563983, 9759745, 9807989, 9654256, 9807987, 9807986, 9519950, 9449869, 9652612, 9737283, 9798584, 10326536, 9798587, 9643742, 9439491, 9469358, 9552027, 9657469, 9626825, 9817289, 9704709, 9769262, 9727786, 9833916, 9443710, 9815866, 9778323, 9626225, 9552036, 9716580, 9440727, 9440743, 9626213, 9839098, 9667264, 9739438, 9818805, 9508188, 9468207, 9667260, 9718051, 9545998, 9704715, 9779710, 9469336, 9727544, 9502624, 9779695, 9521337, 9809925, 9581727, 9708959, 9501747, 9727542, 9508204, 9834259, 9586908, 9564798, 9633672, 9809930, 9651262, 9462527, 9602258, 9562581, 9626838, 9529460, 9546002, 9822087, 9691101, 9535664, 9760288, 9562008, 9851957, 9722203, 9420337, 9498541, 9701417, 9576424, 9727900, 9651731, 9788454, 9701406, 9732194, 9508174, 9571345, 9809945, 9537455, 9802743, 9620898, 9678314, 9581697, 9498539, 9817157, 9545995, 9731049, 9426019, 9580385, 9702435, 9605897, 9740115, 9561995, 9774295, 9517598, 9694753, 9500750, 9817273, 9500751, 9769277, 9672184, 9552998, 9572758, 9501748, 9653491, 9717015, 9443715, 9695995, 9842950, 9819070, 9734541, 9699704, 9503220, 9500334, 9766764, 9614605, 9826312, 9842795, 9517606, 9552032, 9660361, 9492664, 9552067, 9545990, 9669245, 9667255, 9766780, 9699698, 9515998, 9811284, 9820258, 9843465, 9433341, 9655717, 9669254, 9741495, 9718376, 9701103, 9679846, 9648699, 9762783, 9588435, 9741512, 9665189, 9603132, 9817265, 9473250, 9708454, 9843464, 9732187, 9862941, 9639377, 9747868, 9709173, 9856424, 9500719, 9851950, 9499327, 9445311, 9764980, 9839100, 9696002, 9539998, 9717038, 9649457, 9648698, 9626173, 9504938, 9475762, 9679843, 9809932, 9565804, 9486993, 9538962, 9652994, 9620921, 9538967, 9701408, 9441572, 9603138, 9589227, 9648703, 9718377, 9549449, 9596110, 9500747, 9580647, 9731580, 9796841, 9653495, 9519354, 9824573, 9808548, 9549518, 9681776, 9450719, 9639500, 9449726, 9420339, 9471928, 9553664, 9773737, 9773717, 9857880, 9667265, 9502626, 9843458, 9443711, 9847275, 9625308, 9669255, 9779700, 9589244, 9860780, 9491047, 9762782, 9648961, 9549526, 9620330, 9812128, 9610531, 9425916, 9612677, 9741509, 9822092, 9639501, 9727794, 9596048, 9577356, 9571327, 9820260, 9554427, 9696501, 9803001, 9600495, 9741513, 9833911, 9589243, 9727892, 9727891, 9732190, 9820995, 9529407, 9651714, 9551683, 9472506, 9616321, 9764988, 9625683, 9508205, 9625684, 9551674, 9536943, 9554429, 9669244, 9673298, 9704732, 9850489, 9565008, 9659868, 9808597, 9822095, 9503219, 9525542, 9620907, 9693241, 9428212, 9820263, 9847434, 9643859, 10561294, 10371567, 10459959, 10097930, 10589000, 10207809, 10596714, 9872816, 10451461, 10477777, 10395629, 10329812, 10547405, 10089183, 9878638, 10448773, 9920951, 10053177, 10390406, 10400847, 10369709, 10423464, 10332683, 10228117, 10435606, 10550755, 10197835, 9893190, 9933794, 10362188, 9971864, 10510990, 10424293, 10463708, 10094976, 10588407, 10065682, 10543267, 9892252, 10528036, 9927343, 10403729, 10051081, 10214857, 10193737, 10535881, 10547170, 10355573, 10531077, 10369711, 10556107, 10401508, 10438706, 10208786, 9892256, 10069877, 10547171, 10586347, 10442197, 10372249, 10643541, 10383377, 10194427, 10199338, 10075142, 10051239, 10450748, 10601507, 9892329, 10588209, 10510993, 10500058, 9973019, 10483953, 10493326, 10596681, 10424277, 9887160, 10535880, 10547166, 10445600, 10572081, 10586340, 9917117, 9920948, 10351964, 10511608, 10419911, 10543928, 10480522, 10556126, 10359410, 10543929, 10470699, 9950438, 10483950, 10190874, 10588398, 10556125, 10069787, 10051289, 10401948, 10485679, 10577561, 10334442, 10081699, 10459961, 10440302, 10400838, 10334417, 10346773, 10051269, 10543927, 10339471, 10395630, 10581221, 10065667, 9927354, 10207805, 10610644, 10375295, 10588611, 10393475, 10555659, 10486418, 10226092, 10091823, 10556111, 9949315, 10228111, 10332671, 10208142, 10201714, 10510053, 10520779, 10500060, 10413640, 10580457, 10445623, 9929021, 10448774, 10362194, 10065670, 10561314, 10359738, 10400840, 10588404, 10588212, 10596687, 10665890, 10573242, 10440146, 10330387, 10591286, 10071299, 10338459, 10071295, 9989959, 10482985, 10498560, 10466664, 10075949, 10086433, 10514155, 10406361, 10217646, 10341274, 10091816, 10551702, 10440140, 10601381, 10561254, 10347103, 10386508, 10388961, 9927369, 9895383, 10483942, 10527297, 10654204, 10471602, 10030327, 9887161, 10458256, 10200750, 10194151, 10588412, 10584723, 10562262, 10075141, 10533859, 10385642, 10459902, 10609815, 10445599, 10450256, 10484952, 9890856, 10561258, 10235156, 10458232, 10610628, 10385495, 10446101, 10572606, 10421669, 10334540, 10202166, 10190722, 10527293, 10550740, 10487997, 10450010, 10194144, 10450255, 9935014, 9880280, 9862847, 10359733, 10550154, 10565503, 9989566, 10514156, 10231256, 10493319, 10070942, 10364900, 10506597, 9917121, 10080557, 10588987, 10587334, 10092314, 10561192, 10200006, 9895382, 10551711, 10205211, 10068412, 10477530, 10334255, 10099140, 10414394, 10556117, 10359889, 9929085, 10584722, 10051257, 9929018, 10206383, 10401799, 10371571, 10501749, 9933795, 10386509, 10359881, 10390407, 10382693, 10365809, 10502592, 10484948, 10423466, 10029122, 10202165, 10520620, 10475174, 10388979, 10193543, 9933789, 10332684, 10028980, 10480777, 10581142, 10607811, 9950660, 10093981, 10094949, 10450717, 10359466, 10359474, 9862844, 10189536, 9892589, 10430720, 10097932, 10561255, 10475183, 9892294, 10216122, 10359467, 10099907, 10561203, 10502590, 10069782, 10065691, 10190878, 12884887, 10381854, 10430767, 9923768, 10480502, 10587835, 10573278, 9989567, 10208787, 9918905, 10194170, 10351919, 10450271, 10530636, 10588408, 10090111, 10465163, 10551700, 10582932, 10588606, 10521195, 10225813, 10094253, 10486417, 10323627, 10193713, 10458219, 10460188, 10037634, 10205099, 10361108, 10360138, 10591309, 10452766, 10351970, 10553738, 10200751, 10550734, 10338458, 10355575, 9989538, 10392984, 10553752, 10232311, 10562261, 10480773, 10099142, 10470576, 10068095, 10460813, 10459904, 10093686, 10591720, 10547169, 10520791, 9935003, 9935008, 9935012, 10197829, 10601505, 10359886, 10406358, 10400014, 10520785, 10471635, 10390392, 9973021, 10450266, 10400003, 10030325, 10473477, 10485722, 10400007, 10526739, 10561336, 10199880, 10610626, 9892314, 10073514, 10199429, 10086969, 10551687, 10021470, 10556133, 10588224, 9872813, 10228127, 9890849, 9929022, 10432325, 9895384, 10329814, 10588635, 10075964, 10404912, 10332676, 10383370, 10543667, 9872878, 10359391, 10368116, 10218753, 10356008, 10449693, 10323814, 10333912, 10546006, 9927366, 10379017, 10399995, 10526722, 9935011, 10372240, 10421302, 10636366, 9973031, 10334750, 10508797, 9989713, 10343535, 10189539, 10078487, 10411194, 10518167, 10332663, 10509499, 10333947, 10368114, 10332666, 10053176, 9989541, 10379016, 9935020, 10193541, 10208788, 10068383, 10547175, 10200011, 10414411, 10493320, 10543284, 10546695, 10190873, 10082152, 9929086, 10411862, 10487800, 10428734, 10364907, 10362186, 10097934, 10421591, 10193720, 10480500, 9884376, 10527181, 10596705, 9971866, 10381905, 10636368, 10208146, 10334256, 10612317, 10421596, 9929017, 10622297, 9917116, 10440307, 10573224, 10500056, 10066203, 10489947, 10518846, 10209977, 9892253, 10217083, 10498559, 10385666, 10359475, 10051494, 10440305, 9895397, 10340366, 10232312, 10589004, 10545434, 10561190, 10220494, 10486353, 10072409, 10199874, 10488709, 10573277, 10518177, 10517729, 10561249, 10228142, 10092259, 10561191, 10334534, 10075615, 10551714, 10508816, 10573241, 10362203, 10487792, 10208998, 9927363, 9862828, 10430734, 10051277, 10089184, 10551494, 10369847, 10665894, 9973029, 10342514, 10329078, 10520789, 10458254, 10480826, 10385499, 10562581, 10586336, 9892304, 10359887, 9949318, 10327910, 10588216, 9927098, 10543286, 10419902, 10587340, 10489821, 10550131, 10475175, 10543285, 10068382, 10550150, 10438259, 10199877, 10484538, 9974458, 9862830, 10359389, 10338454, 10450749, 10550137, 10201721, 10533862, 10377079, 10561339, 10421667, 10561201, 10337542, 10075140, 10463897, 10577572, 10440160, 10604885, 10615075, 10488711, 10480765, 10065662, 10329808, 10094939, 10351973, 10501359, 10340369, 10359888, 10588620, 10561316, 10591711, 10362215, 10546008, 10588962, 10227322, 10200009, 10393676, 9892302, 10323634, 10071280, 10023946, 10471608, 10393682, 10555719, 10340903, 10334550, 10218756, 10520778, 10091835, 10199878, 10367822, 10458255, 10080566, 10333897, 10096932, 10493846, 10480767, 10332685, 10334415, 10577995, 10334416, 10200742, 10454944, 10480825, 10329815, 10381531, 10401453, 10489823, 10402447, 10477776, 10458713, 10090927, 10388983, 10091821, 10362210, 10622295, 10228190, 10091827, 10334254, 10468521, 9935018, 10233878, 9989957, 9973009, 10343521, 10561302, 10501745, 10367823, 10452757, 10194143, 10069881, 10430716, 10509496, 10430735, 10359882, 10482855, 10193729, 10520799, 10561297, 10545432, 10506042, 10543291, 10327908, 10480516, 10030326, 10421300, 10086723, 10438710, 10392982, 10385662, 10572078, 10365803, 10419923, 10588591, 9878641, 10030329, 10515398, 10591382, 10340958, 10517731, 9892313, 10485720, 9872815, 10440143, 10080465, 10074011, 10449701, 10334434, 10091831, 10484950, 10577993, 10372241, 10588965, 10419912, 10334422, 9929524, 10464128, 10547167, 9971865, 9917118, 10228126, 10528035, 10520796, 10072411, 10097929, 10545430, 10643535, 10561205, 10515396, 10385496, 10051505, 10547403, 10493207, 10441602, 10359405, 10362195, 10411845, 10207795, 10097931, 12884888, 10080463, 10099910, 10379018, 10051462, 10094637, 10334425, 10205101, 10080461, 10392981, 10587839, 9989716, 10419922, 10094948, 10636369, 10520780, 10390385, 9872843, 10525491, 10086963, 9989963, 10580024, 10029609, 10480503, 10071274, 9892450, 10401939, 10506617, 10086434, 10437864, 10588598, 10489822, 10482826, 10588996, 10543669, 9935021, 10329064, 10068658, 10360370, 10333932, 10561318, 10102851, 10480824, 10550151, 10480779, 10334433, 10553740, 10071265, 9862851, 10076721, 10066201, 9988249, 10403850, 10520808, 9989964, 10190822, 9922304, 9924052, 10362048, 10460185, 10508811, 10588226, 9973032, 10515399, 10390384, 10466665, 9892449, 10433621, 10440303, 10080605, 9892301, 10327902, 10577294, 10232294, 10200003, 10328256, 10454403, 10333914, 10561173, 9973008, 10604884, 10500075, 9922309, 10465169, 10440141, 10194235, 10453931, 10092302, 10561210, 10075616, 10200005, 10423465, 9892331, 10049056, 10636365, 10574857, 10219066, 10573245, 10445622, 10334441, 10071275, 10489843, 10577848, 10470568, 10202164, 10071003, 10091832, 10391813, 10422996, 10334546, 10372238, 10604887, 10561229, 10561342, 10561349, 10561343, 10441604, 10218748, 10543269, 9927101, 10080558, 10550153, 10480508, 10080462, 9862846, 10545429, 9950657, 10468503, 10189538, 10442189, 10335782, 10514160, 10399998, 10193730, 10450715, 10382694, 10624759, 10508799, 10381910, 10482213, 10361110, 10483964, 10588229, 10195971, 10430731, 10023893, 10369848, 12884889, 10193725, 10080458, 10514159, 10411846, 10506601, 10216104, 10483952, 10516448, 10551383, 10568561, 9933797, 9989537, 10199336, 10488703, 10577295, 10550135, 9918518, 10323626, 10090926, 10096935, 10588623, 10561296, 10334526, 10561363, 10458241, 10071291, 10225812, 10069783, 10216145, 10452767, 10506041, 10338457, 10596684, 10325520, 10325515, 10390425, 10050868, 9872814, 10323625, 10561283, 10359461, 10414418, 10543293, 10568572, 10208785, 10470423, 10051255, 10420027, 10226086, 10462387, 10559610, 10458211, 10231255, 10381711, 10024260, 10591717, 9888904, 10426734, 10074017, 10390455, 10205213, 10574854, 10205197, 10577635, 10550090, 10506039, 10398629, 10577853, 10510989, 10551699, 10334517, 10506633, 10493324, 10080588, 10194425, 10506598, 10458252, 10458212, 10458218, 9927358, 10351924, 10096928, 10442192, 10561291, 10550134, 10506606, 10547391, 10445610, 10561206, 10390399, 10601378, 10469751, 10069785, 10506596, 9862819, 10836333, 10432030, 10520777, 10546697, 10381908, 10591384, 10562579, 10597756, 10029593, 10546696, 10201726, 10213716, 10493325, 9917226, 9862876, 10208784, 9892586, 10517716, 10484540, 10051263, 10393675, 10206381, 10208996, 10577566, 10069869, 10520795, 10597759, 10333895, 10437863, 10437857, 10208143, 10543268, 10559611, 10534462, 10385667, 10471604, 10432323, 9927359, 10484947, 10561182, 10411851, 10489948, 10484534, 10081698, 10561263, 10406187, 10329092, 10385494, 10334524, 10406359, 10591712, 10217079, 10482210, 10205205, 10346769, 10489950, 10528024, 10361111, 10069784, 10459958, 10459957, 10550737, 9927145, 9952201, 10024253, 10414395, 10573247, 10421644, 10199350, 10430726, 10194172, 10051076, 10438709, 10581136, 10591288, 10318656, 10213347, 10433619, 10334412, 10584721, 10452771, 9935007, 10399993, 10329080, 10069876, 10400005, 10208997, 10024259, 10553750, 10588630, 10836329, 10390394, 10376613, 10473995, 10559616, 10228115, 10550087, 10450276, 10836327, 10065696, 9862848, 10365287, 10498596, 10562483, 10588963, 10189324, 10390376, 10390389, 10543292, 10375338, 10591300, 10213349, 10376571, 9893184, 10471456, 10543296, 10543294, 10462363, 10588995, 10643545, 10091815, 9872822, 10446118, 10400846, 10371231, 10408951, 10506087, 10525492, 10329069, 10193727, 9935005, 9869600, 10600959, 10515401, 9922303, 10362181, 10496829, 9935002, 10221945, 10385633, 10416909, 10080464, 10577557, 10486370, 10510985, 10200010, 10416915, 10197825, 10508813, 10347985, 10513708, 10401802, 10450257, 10595949, 10520784, 10430713, 10597763, 10329810, 10515402, 10359885, 10517908, 10561358, 10080612, 9895371, 10561188, 10421597, 10232292, 9862845, 10489946, 10028979, 10502591, 10553743, 10573044, 10607813, 10232298, 9989555, 10096930, 10347132, 10519894, 10482825, 10401446, 10360371, 10518174, 10334751, 10480774, 10099908, 10401941, 10351966, 10577636, 9869598, 10051499, 10550081, 9920950, 9892590, 10493845, 10332014, 10025887, 10568567, 10573515, 10517717, 10480766, 10480775, 10332682, 10408485, 10517426, 10536125, 10357691, 10091825, 10320384, 10075960, 10362041, 10357295, 9918521, 10545428, 10588589, 10458715, 9973007, 10334431, 10506613, 10379020, 10526726, 12892048, 10452746, 9918478, 10489824, 10520790, 10448776, 10404908, 11082090, 10902624, 10877738, 11111188, 11092290, 10676671, 10852998, 10675426, 11096165, 11036118, 10974130, 10974132, 11058673, 10833208, 10816183, 10841872, 10933738, 10841218, 10683053, 10960463, 10891035, 10920469, 10753831, 10665616, 10816186, 10712354, 10807486, 11040176, 10764700, 11074901, 10807891, 11036893, 10707873, 10690382, 10698808, 10839333, 10833209, 10711914, 10673303, 10807482, 10707786, 10807893, 11061615, 10898316, 11052840, 10673248, 10891016, 10927727, 10665619, 10982759, 11088092, 11102253, 11096238, 10841243, 11085962, 10904458, 10761963, 10717010, 11071672, 11079649, 10871971, 10903249, 10891434, 10756219, 10807488, 10915751, 10712326, 10852750, 11069821, 11106119, 11018164, 10807894, 10623703, 10910802, 10931799, 10869264, 11034942, 11061603, 11056096, 10807454, 10987609, 11031332, 10724056, 10801169, 10623705, 11022927, 10831468, 11153582, 10834865, 10903236, 10885423, 10920060, 10653831, 10979796, 10982756, 10719296, 10770306, 10784625, 11036894, 11097979, 10618302, 10731405, 10731406, 10731407, 10758987, 10758988, 10758989, 10761967, 10738049, 10782908, 10655529, 11153592, 10710018, 10875827, 11078488, 11078487, 10985708, 10716475, 10668837, 10987602, 11029334, 10768435, 10764316, 11127443, 10945387, 10831476, 10867779, 10716463, 10988016, 11092282, 10776742, 10776736, 10673065, 10836911, 10737286, 10756223, 11089754, 10866444, 11067784, 10655249, 10933350, 11078770, 11130385, 10807441, 10807442, 10889149, 11029348, 10711913, 11075771, 10809032, 10937511, 11028477, 10891025, 10857960, 10805824, 10801755, 10856103, 10720126, 10714732, 10987605, 11106123, 11001892, 10964871, 10865273, 10937514, 10968501, 10903240, 10735888, 11073017, 11106211, 10725290, 10995804, 10993855, 10715306, 10945384, 10985706, 10985712, 10985714, 10985710, 10869261, 10824072, 10841126, 10747343, 10841242, 11310906, 10901509, 11097959, 10968495, 11106679, 10636263, 11106204, 10769276, 11053072, 10711924, 11023504, 10716461, 11115306, 10607690, 10841227, 10764306, 11283120, 10632230, 10871969, 11112236, 10865301, 10815116, 11283121, 10827161, 11071800, 10915748, 10991635, 10673196, 11020002, 10841825, 11060324, 10934092, 10984370, 10636260, 10705200, 10806134, 10733492, 10637238, 10735395, 10977011, 10759446, 11040177, 10884257, 10924317, 10856142, 11074900, 10950233, 10791373, 10784465, 10968813, 10653877, 10789602, 11053065, 11153576, 10780756, 11087881, 10968433, 10755498, 10950230, 10764307, 10698807, 11042755, 10808172, 10764420, 10733441, 10988131, 11271083, 10988090, 11030295, 10764336, 10611148, 10859289, 10760307, 10933737, 10758046, 10716474, 11053073, 10764368, 11030166, 11082083, 10818026, 11130524, 11074747, 10645924, 11015817, 10739728, 10653826, 10761749, 10963247, 10977833, 10891977, 10952950, 10894871, 10961865, 11001889, 10933100, 10816184, 10710017, 10907942, 10889127, 10758969, 10857963, 10860190, 10875825, 10784638, 11028466, 10841003, 10807453, 10985711, 11153584, 10676668, 11023139, 10952250, 10920067, 10982541, 10898435, 11094033, 10623701, 10832825, 11015162, 10766581, 10963641, 11135780, 10784478, 11092646, 10811496, 10610747, 10952955, 10698876, 10634454, 10872136, 10942739, 10618026, 10964860, 10931801, 11025377, 10964859, 11009140, 10899092, 10929163, 10755499, 11020003, 10716478, 11102252, 10683348, 10859288, 10639305, 10859037, 11113041, 11030854, 10872014, 10872013, 10929164, 10873968, 10937515, 10629468, 10632308, 11004140, 10860192, 10703779, 10952951, 10733540, 10927737, 11089824, 10908209, 10926589, 10877734, 10711927, 10952952, 11145491, 10990104, 11193270, 10675425, 10987596, 10732888, 10764703, 10896910, 10683000, 10678615, 10856147, 10913058, 11023140, 11113039, 10922420, 10986219, 10821361, 10903217, 10911005, 11095259, 10755495, 10683349, 11153591, 11030682, 10979113, 11089744, 10675166, 10703801, 10895854, 10905240, 11081529, 11034935, 11310907, 10944140, 10666187, 10934081, 11056099, 11191537, 11071181, 10840987, 10904510, 10807440, 11015795, 10673253, 11128345, 10838648, 11015798, 10651597, 11147984, 10675118, 11039967, 11085961, 10808173, 10639539, 11191542, 11023935, 10636259, 10942743, 10759443, 11059859, 10826459, 11088095, 10880416, 10714728, 11089748, 10892761, 10779449, 10954759, 10865304, 10889166, 10644314, 10691585, 10831472, 10896637, 10904459, 10675165, 10619824, 10780760, 11071674, 11153594, 10753829, 10868866, 10676669, 10805823, 11130382, 11310905, 11191543, 10683004, 10648452, 11112112, 10665615, 10806139, 10979879, 10885420, 10898421, 10676670, 10920066, 10904461, 10828671, 10780761, 10720857, 11112243, 10744088, 11110737, 10868824, 11067793, 10866439, 11056593, 10770145, 10904462, 10697061, 10984366, 10665554, 10924316, 10831478, 10834422, 11074869, 11036121, 10791526, 10986547, 10871975, 10988110, 11145488, 10963197, 10865302, 10811503, 10758949, 11105182, 10636270, 11044424, 10874061, 10868869, 11095258, 10982755, 10960435, 10636261, 10637204, 11094034, 10789666, 11113045, 10920133, 11089816, 11205461, 11103054, 10770146, 10697060, 10717011, 10972371, 11131448, 10861309, 10710578, 11056590, 11020001, 10694559, 10642233, 11079651, 10711915, 10735887, 10888969, 11029335, 10780766, 11029326, 10963636, 10789612, 10665618, 10885415, 11079647, 10898322, 11022928, 10712324, 10671393, 11078493, 10894863, 10920064, 10688561, 10761763, 11114313, 10910801, 10834419, 10807895, 10676676, 10979111, 10894864, 11001674, 10893283, 10716466, 10964861, 10666351, 10952826, 10859040, 11076818, 11061616, 10688810, 10673519, 10702219, 10761968, 10735896, 10981888, 11092663, 10636257, 10908213, 10807465, 10990114, 10856105, 10716460, 10851205, 10694560, 11075769, 10987607, 11205467, 11030848, 11092645, 11074735, 10607712, 11121460, 10735893, 10758047, 11061604, 10731173, 11069800, 10889591, 10979946, 10634337, 10993854, 11085964, 10752699, 10985709, 10673291, 10700435, 10868864, 11126257, 10828010, 11076820, 10790348, 10989404, 10637255, 10874063, 10857945, 10831521, 10645920, 11096166, 10637198, 10857934, 11052839, 10873918, 10988100, 10756218, 11089761, 10852774, 11292113, 11080706, 11081528, 10784307, 11058672, 10961957, 11120691, 10704165, 10715299, 10636265, 10720131, 10811664, 10696980, 10915728, 10673309, 10734016, 11096234, 10619802, 11023136, 10801757, 10977030, 11006365, 10984365, 10965009, 11087880, 10615885, 10815082, 10911006, 10766680, 10988126, 10744089, 11006366, 10869301, 10706565, 10831487, 11096246, 10827148, 11110676, 10673292, 10770980, 10770979, 11128344, 10716688, 10871974, 10675115, 10644315, 10898414, 10688831, 10834423, 10937510, 10944126, 11094035, 11015339, 10993857, 10720861, 11052841, 10840011, 10716682, 10764302, 10669845, 11074745, 10636276, 11023137, 10898445, 11027739, 11023927, 11112238, 10868865, 10782903, 10889125, 10942231, 10843828, 11049974, 10887107, 11128341, 10655253, 10904087, 10860187, 10940701, 10853852, 10793164, 11079643, 10831474, 10678614, 11079650, 10770301, 10942228, 10636264, 11191541, 10933364, 11015164, 10856145, 10675427, 11034937, 10904465, 10904464, 10647762, 10944134, 10944552, 10885356, 10973836, 10880548, 10669844, 10601063, 11036119, 10887306, 10629456, 10984373, 10634336, 10826461, 11054442, 10904511, 10768702, 10714729, 10647800, 10684911, 10952827, 10894877, 10575270, 10627441, 10984368, 10808170, 10714731, 10611130, 10979060, 10639301, 10972368, 10632236, 10960437, 11080723, 11023932, 10797038, 10781354, 10764298, 11106203, 10678628, 11092289, 11127455, 10852793, 10756221, 10850507, 10944645, 10711911, 10744083, 10731402, 11074742, 11058482, 11104737, 11283130, 10673189, 10706509, 11191538, 10634340, 10903230, 10698809, 10990101, 10659875, 10764365, 10898416, 11015815, 10737284, 10944641, 10719298, 10688560, 10968496, 10834421, 10653675, 10881893, 10610746, 10683057, 10927732, 10963245, 10776745, 10930365, 10908215, 11059861, 10819701, 11127459, 10889124, 10950231, 10801756, 11067788, 10644288, 10807468, 10985713, 10892758, 10811665, 11127445, 11028464, 10808186, 10979067, 10715288, 10898434, 10997806, 10673513, 10856099, 10990103, 10764439, 10650023, 11106715, 11106716, 10634735, 11113085, 10858178, 10868867, 11095257, 11056097, 11085693, 10856108, 10736287, 11106122, 10841002, 10979948, 10811676, 11099326, 10894870, 11013280, 10856098, 10623704, 10623700, 11013273, 10623702, 10944130, 10698828, 11034574, 10768434, 11079667, 10871977, 10976083, 11092281, 10776735, 10826457, 10784481, 10831483, 11052579, 10924298, 10681282, 10735895, 10787361, 10922421, 10811588, 10807457, 11004144, 10636271, 10851204, 10840005, 10845671, 10960456, 11099319, 11089753, 10905597, 10834894, 10715297, 11087879, 10900277, 10699160, 10657332, 10632281, 11110739, 10784636, 10806014, 10716469, 10924313, 10979947, 10910888, 10902623, 10880411, 10648454, 11310903, 10841241, 10683351, 10694547, 11029359, 10811675, 11054434, 11004136, 10934099, 11096237, 10619792, 10791521, 10732887, 10940698, 10765092, 10657327, 10845898, 11118468, 10944563, 10673523, 10801172, 10683002, 11053174, 11039963, 10764367, 10634736, 11030677, 11110735, 10948025, 11053173, 11099284, 11099285, 10764366, 10617523, 11030676, 10761756, 10940699, 10972367, 10688563, 11061730, 10807619, 10623698, 10675381, 10681486, 10715302, 10942742, 10894865, 11052843, 11113043, 10993853, 11113042, 10993856, 11029341, 10613728, 10910797, 10942740, 10784622, 10793106, 10811673, 10894866, 10623706, 10856094, 11023928, 10811493, 10735892, 10704160, 10944557, 10653869, 10801758, 10623692, 10673518, 10655439, 10987586, 10764435, 10653861, 10880550, 10699069, 10893282, 10942744, 11080722, 11003631, 10637236, 10933355, 11007727, 10663279, 10868832, 10952840, 11118463, 11041396, 11113068, 10889153, 10836913, 10932067, 10707859, 11115311, 10896648, 10764156, 10737282, 10807447, 10905242, 10662743, 10764312, 10695689, 10739409, 11102257, 10931808, 10889132, 10895850, 10764301, 11069801, 10973843, 10711928, 10784479, 11087700, 10716459, 10665556, 10673512, 11117910, 10732934, 10811678, 10710579, 10727589, 10807887, 10765098, 10705194, 11061732, 10973767, 11041902, 11310908, 11113067, 11032589, 11102255, 10811587, 10838647, 10856143, 11142481, 10933104, 10690389, 11072938, 10761956, 10858176, 10601051, 10706488, 10988107, 10716467, 10789609, 10627439, 10999974, 10668836, 10869286, 11028492, 10986206, 10905235, 10934078, 10898408, 10977009, 10933353, 10834891, 10678627, 10803508, 10673175, 11106218, 10926587, 10601068, 10984382, 10965008, 10673249, 11081531, 10711925, 10678612, 11028483, 11071802, 11089822, 11086366, 11124171, 10636278, 10801752, 10960446, 10829042, 10744590, 10715293, 10826458, 11069791, 10639541, 10735891, 10791524, 10944137, 11023929, 11120696, 11053067, 10732933, 11052580, 11032592, 10950232, 10702196, 10735611, 10655440, 10683055, 11090092, 10907947, 11074738, 10826454, 10732883, 10985707, 10933361, 10707787, 10759437, 10749962, 10874062, 10766682, 11096167, 10852765, 11029368, 10933082, 10575269, 10924301, 10903223, 10707793, 11093724, 10675424, 11044422, 10839336, 11112128, 10898413, 10676687, 10852763, 10847261, 10857943, 10933349, 11054390, 11115310, 10843823, 10905596, 11119395, 10716471, 10871979, 10764713, 10938172, 11004139, 10903213, 10639542, 10891022, 10678632, 11112113, 10977012, 10676679, 10653825, 10665617, 10834424, 10715308, 10666180, 10988013, 10807483, 10934074, 11072942, 10619812, 11052845, 11086369, 11310904, 10694522, 10974131, 10707784, 10758957, 10725288, 10770982, 11066186, 11052582, 11013281, 10995002, 11000645, 10712318, 10791374, 11118461, 11071801, 11115305, 11075768, 10984564, 10950228, 10836915, 10960460, 11079654, 11029345, 10707876, 10885414, 11076823, 10695692, 10765090, 10834413, 10834412, 10898425, 10735900, 11035891, 10735604, 10673514, 10864908, 10716458, 11145492, 11142488, 11085838, 11030804, 10639540, 10898419, 10977039, 11034934, 11092294, 10961868, 10903238, 10934090, 10885352, 11680444, 11408300, 11133742, 11455971, 11547716, 11685307, 11794192, 11575289, 11156812, 11281559, 11481167, 11742269, 11742270, 11398877, 11762807, 11534565, 11677198, 11913480, 11685297, 11483123, 11136647, 11431228, 11729030, 11481623, 11606503, 11264156, 11343232, 11709513, 11431744, 11342423, 11485506, 11174186, 11679455, 11401123, 11329400, 11545671, 11525706, 11487536, 11735841, 11283865, 11521785, 11231831, 11423507, 11759645, 11371418, 11484687, 11211889, 11481625, 11176767, 11559309, 11704394, 11484951, 11403810, 11287372, 11511121, 11228275, 11386264, 11231744, 11447080, 11712935, 11382723, 11157716, 11313303, 11493132, 11304774, 11176769, 11686668, 11208622, 11719300, 11679451, 11705818, 11606149, 11520725, 11438132, 11344343, 11315837, 11266371, 11716884, 11230464, 11134190, 11739201, 11481622, 11176768, 11150359, 11179128, 11463590, 11734428, 11375376, 11146706, 11315838, 11502317, 11313302, 11115818, 11751188, 11387347, 11313304, 11499722, 11282864, 11300442, 11698275, 11532742, 11296098, 11568067, 11730442, 11153772, 11738299, 11300428, 11425770, 11751195, 11583199, 11182837, 11358776, 11343441, 11673199, 11524396, 11520775, 11300448, 11733393, 11254519, 11686667, 11315821, 11315826, 11289465, 11213870, 11222470, 11300425, 11405516, 11719356, 11413076, 11176735, 11352694, 11734230, 11465968, 11296091, 11418458, 11499745, 11691500, 11497535, 11210994, 11172190, 11557704, 11684627, 11345366, 11448369, 11316648, 11293640, 11293641, 11463597, 11500348, 11751182, 11739158, 11716885, 11600473, 11520523, 11704374, 11136837, 11704367, 11691528, 11245649, 11177119, 11468195, 11547741, 11315833, 11498212, 11401114, 11587207, 11568032, 11479249, 11691689, 11522739, 11697832, 11398690, 11576036, 11387182, 11308399, 11265953, 11309635, 11732958, 11230751, 11679972, 11511139, 11728147, 11177313, 11171785, 11280651, 11171786, 11350844, 11369685, 11754675, 11297702, 11419424, 11238151, 11238150, 11549529, 11214127, 11684212, 11739159, 11304782, 11237545, 11488317, 11762816, 11236936, 11575978, 11213882, 11181776, 11463592, 11419425, 11253966, 11571242, 11242428, 11290635, 11495620, 11315832, 11557703, 11255384, 11345371, 11527647, 11410192, 11571221, 11369630, 11342473, 11445099, 11673197, 11729034, 11397745, 11578996, 11146707, 11560537, 11334121, 11177120, 11245646, 11454386, 11466099, 11179125, 11752357, 11739299, 11890650, 11343523, 11211903, 11417474, 11250997, 11810130, 11757505, 11738102, 11575287, 11401877, 11413083, 11673340, 11430552, 11570941, 11587992, 11282700, 11413085, 11527645, 11136842, 11216954, 11549300, 11520714, 11673321, 11843242, 11484954, 11532838, 11398878, 11481357, 11304776, 11520717, 11181656, 11510809, 11115824, 11559318, 11464037, 11384898, 11431240, 11733394, 11251000, 11511559, 11230476, 11673326, 11136956, 11448376, 11296075, 11794169, 11273059, 11591611, 11386892, 11263603, 11435249, 11343737, 11463605, 11527638, 11558482, 11723015, 11549539, 11356439, 11425369, 11356434, 11386263, 11427138, 11704596, 11377645, 11309632, 11387349, 11520726, 11476835, 11578152, 11425411, 11527612, 11513909, 11306519, 11280671, 11564483, 11387176, 11567700, 11575982, 11591613, 11289473, 11738309, 11282765, 11743836, 11405514, 11346808, 11502614, 11238152, 11229669, 11551575, 11529210, 11230471, 11386927, 11159619, 11748094, 11172164, 11434826, 11300427, 11434828, 11289350, 11431296, 11264206, 11718591, 11529805, 11587974, 11520724, 11709454, 11154618, 11732953, 11176912, 11491155, 11308434, 11247549, 11695935, 11526531, 11228276, 11511562, 11352878, 11277825, 11396283, 11587995, 11709518, 11519503, 11747380, 11440493, 11171788, 11181473, 11387359, 11516093, 11329233, 11289347, 11282762, 11744561, 11352697, 11565518, 11419884, 11259720, 11504771, 11810123, 11551875, 11549528, 11280672, 11447379, 11567703, 11520521, 11181464, 11479247, 11231700, 11177057, 11751349, 11679464, 11597664, 11406520, 11343248, 11913478, 11231747, 11500334, 11136953, 11410193, 11216977, 11729013, 11172346, 11236773, 11355117, 11231932, 11322207, 11483144, 11172176, 11295959, 11457747, 11386980, 11602481, 11210993, 11463593, 11434846, 11747915, 11729012, 11691705, 11398072, 11194244, 11729015, 11797027, 11172150, 11547718, 11216952, 11499744, 11579010, 11691700, 11445103, 11386930, 11344347, 11315834, 11398691, 11250991, 11149991, 11438489, 11730394, 11432629, 11677200, 11181777, 11516109, 11822763, 11584378, 11497140, 11343482, 11691758, 11371411, 11280620, 11583888, 11401128, 11587988, 11313239, 11391543, 11705559, 11584376, 11451176, 11519502, 11693741, 11455982, 11484695, 11333290, 11194214, 11759644, 11150109, 11562389, 11331320, 11251010, 11134196, 11295650, 11306521, 11535566, 11794149, 11794148, 11413082, 11377600, 11675056, 11484961, 11568045, 11719362, 11511120, 11532842, 11473079, 11491175, 11277826, 11213881, 11136839, 11719353, 11149996, 11398073, 11375359, 11430539, 11583752, 11315820, 11289469, 11208636, 11403809, 11677204, 11416115, 11181472, 11487548, 11431729, 11322206, 11583189, 11488316, 11597289, 11331315, 11756577, 11387366, 11434728, 11497156, 11403068, 11692097, 11388815, 11734596, 11176737, 11558483, 11171827, 11237056, 11476655, 11251012, 11369627, 11390337, 11208680, 11213866, 11738300, 11332151, 11568049, 11134208, 11504743, 11375357, 11510790, 11571237, 11529282, 11334124, 11300445, 11315836, 11222362, 11352963, 11289483, 11401608, 11728145, 11723020, 11520815, 11157017, 11673331, 11408508, 11304773, 11322841, 11176963, 11689582, 11574431, 11748098, 11583883, 11280679, 11313245, 11368733, 11549530, 11549532, 11177333, 11704602, 11398082, 11545670, 11600601, 11594896, 11157010, 11794168, 11574432, 11208835, 11682037, 11682036, 11408506, 11413109, 11159890, 11704586, 11342474, 11300450, 11377597, 11280632, 11401938, 11240956, 11692092, 11560454, 11401117, 11313301, 11157692, 11133794, 11255425, 11704378, 11451294, 11159567, 11146708, 11464036, 11566828, 11606504, 11731509, 11735758, 11447381, 11207351, 11216978, 11150012, 11691524, 11208838, 11352886, 11544459, 11208681, 11559718, 11268267, 11136681, 11172151, 11368732, 11315819, 11448378, 11214126, 11430325, 11435237, 11592603, 11347747, 11729022, 11343480, 11157012, 11451296, 11794191, 11300443, 11187414, 11560538, 11709512, 11704584, 11572739, 11240946, 11742271, 11282901, 11387364, 11463600, 11500333, 11208637, 11704585, 11482924, 11580151, 11325322, 11481349, 11434842, 11194245, 11430324, 11731389, 11343739, 11412051, 11695951, 11594897, 11287447, 11309436, 11171680, 11583861, 11673330, 11401884, 11342422, 11719309, 11568065, 11177112, 11728555, 11679459, 11712936, 11762813, 11738097, 11343529, 11675332, 11157525, 11742047, 11438134, 11716166, 11182836, 11479245, 11544467, 11496850, 11430537, 11759643, 11483142, 11568036, 11323056, 11216970, 11153777, 11535506, 11719310, 11350109, 11719297, 11172189, 11673215, 11153737, 11684630, 11691690, 11375363, 11559654, 11496232, 11207085, 11213884, 11755608, 11283143, 11176812, 11739137, 11208843, 11738277, 11345379, 11405809, 11265951, 11319192, 11689577, 11115819, 11822766, 11432886, 11466098, 11575286, 11274622, 11587994, 11481387, 11263615, 11583749, 11451264, 11375230, 11216961, 11435337, 11468200, 11468201, 11251005, 11484963, 11181673, 11709567, 11408507, 11181662, 11454883, 11502700, 11606146, 11343440, 11350112, 11401883, 11579011, 11296070, 11208829, 11729017, 11401111, 11419426, 11368699, 11377599, 11371413, 11511119, 11156873, 11705560, 11282702, 11468148, 11691515, 11453713, 11159657, 11375232, 11157685, 11181465, 11254520, 11730396, 11709566, 11547717, 11822750, 11153773, 11401051, 11296099, 11274623, 11570939, 11251007, 11521801, 11333990, 11500337, 11297958, 11596587, 11499735, 11810122, 11133211, 11408497, 11156874, 11451268, 11242425, 11673338, 11396282, 11520781, 11141144, 11450675, 11405527, 11731997, 11583862, 11528199, 11159875, 11149993, 11401053, 11533101, 11694150, 11245647, 11704371, 11214131, 11559726, 11333992, 11583750, 11179160, 11521809, 11597291, 11744565, 11532845, 11532844, 11463679, 11408301, 11739221, 11451784, 11302905, 11179154, 11691759, 11358775, 11576977, 11679387, 11348910, 11423506, 11431299, 11431298, 11290639, 11843249, 11709448, 11416112, 11435336, 11579113, 11419885, 11560848, 11697833, 11489769, 11588025, 11487547, 11479250, 11177066, 11579006, 11709562, 11309437, 11521804, 11352955, 11432888, 11179104, 11340285, 11532743, 11322842, 11300437, 11352967, 11343496, 11689579, 11177061, 11567990, 11231701, 11451173, 11208820, 11732951, 11282766, 11689583, 11697835, 11435335, 11331253, 11300446, 11238259, 11211888, 11255520, 11435251, 11728564, 11794170, 11454878, 11230490, 11723089, 11488319, 11250852, 11701572, 11401940, 11347755, 11581138, 11762805, 11216965, 11571244, 11208630, 11520524, 11268266, 11423497, 11694151, 11535502, 11511952, 11527621, 11565517, 11425410, 11673219, 11558484, 11495621, 11730397, 11350098, 11401073, 11255445, 11300330, 11208823, 11345378, 11822760, 11249050, 11230478, 11222463, 11496849, 11680441, 11405513, 11734429, 11431238, 11428852, 11304780, 11451263, 11323042, 11413116, 11231941, 11171824, 11149999, 11425416, 11705489, 11526545, 11439963, 11181775, 11829098, 11250995, 11685304, 11259471, 11822764, 11171823, 11575284, 11675061, 11481350, 11709564, 11156811, 11333294, 11230472, 11157688, 11447078, 11522582, 11463595, 11495047, 11679448, 11282761, 11468204, 11735775, 11743835, 11222469, 11498488, 11757504, 11585480, 11240955, 11425414, 11377644, 11685350, 11194215, 11150007, 11438499, 11136838, 11581136, 11673341, 11704369, 11263625, 11114277, 11157015, 11177331, 11315840, 11575280, 11396434, 11352951, 11718589, 11157686, 11432623, 11343530, 11368700, 11738312, 11691521, 11435293, 11560455, 11329407, 11606148, 11156638, 11346327, 11408334, 11710890, 11181655, 11698565, 11283119, 11483141, 11213869, 11153778, 11176959, 11177086, 11529281, 11843243, 11405507, 11739135, 11559644, 11473065, 11329231, 11346806, 11527646, 11401110, 11386896, 11371410, 11716168, 11423492, 11742045, 11485511, 11565519, 11282760, 11345387, 11401052, 11487483, 11493164, 11510804, 11211891, 11738278, 11510780, 11306897, 11320385, 11176732, 11603909, 11252116, 11388817, 11695932, 11704395, 11590377, 11520776, 11535508, 11794219, 11194219, 11375358, 11692096, 11601838, 11222419, 11216968, 11418151, 11463010, 11741625, 11520780, 11668132, 11245644, 11300432, 11723014, 11551576, 11176729, 11156644, 11171825, 11240954, 11304763, 11413078, 11642232, 11171831, 11172139, 11236774, 11159568, 11557646, 11491148, 11136631, 11445102, 11474662, 11448297, 11578994, 11520836, 11602495, 11522698, 11388814, 11520711, 11450676, 11576031, 11307743, 11529283, 11316640, 11248153, 11157022, 11468198, 11583866, 11308436, 11346805, 11457746, 11231746, 11156542, 11308437, 11283125, 11528196, 11691516, 11306524, 11591618, 11463411, 11313314, 11172188, 11673318, 11532741, 11171790, 11684628, 11557651, 11434791, 11377642, 11551574, 11136682, 11434796, 11297959, 11786584, 12356631, 11863094, 12377647, 12215081, 11821508, 12466507, 11777998, 12447516, 12444178, 12110735, 11907291, 12470183, 11948271, 12351470, 12401756, 12365878, 12090818, 11863117, 11982448, 11866001, 12475448, 11919122, 12191747, 12417879, 12377644, 11925292, 12196372, 12419751, 11874821, 11772696, 12153372, 11869857, 12362007, 12427785, 12475455, 12142115, 12020483, 11919129, 12045127, 12358344, 12234503, 12122526, 12117888, 12123334, 11772963, 12181240, 11904653, 12010890, 12297844, 12050336, 11755281, 12150647, 11877270, 12454831, 11823075, 11897643, 11983755, 12055582, 12453982, 12176866, 12181245, 12181246, 12119223, 12096085, 12198662, 11926939, 12361444, 12415457, 11919132, 11861264, 12038949, 12226148, 12196430, 12401759, 12444837, 12359680, 12020186, 11823086, 12039480, 12038883, 12360462, 11842294, 12359855, 12123332, 12095385, 12077091, 12427650, 12453973, 12078888, 12086760, 12106935, 12392825, 11967598, 11754709, 12145231, 11823078, 12204508, 12415443, 12091332, 12030726, 11821466, 12160582, 12123331, 11870016, 11919130, 12359687, 11886009, 12377961, 11956267, 11956266, 11926785, 12086759, 12087021, 12297843, 12020142, 12063522, 12105162, 12393590, 12234947, 12076241, 12089090, 11812555, 12212960, 11907287, 12093058, 11978332, 12042011, 11792137, 11925310, 12412687, 11799031, 11912111, 12090977, 11901038, 12370218, 11879862, 12145221, 12414201, 12037148, 12432044, 11779730, 12204869, 12091205, 11966338, 12377643, 12411229, 12379573, 12049862, 12126819, 12163424, 12446070, 12142121, 11815494, 11991878, 12458242, 12505234, 12412686, 12135935, 12142119, 12090814, 12038918, 12480423, 12153960, 12087019, 12204500, 11981006, 12020525, 11956046, 12198699, 12419788, 12464648, 11978337, 12122534, 12425706, 12042193, 11786563, 12351466, 12427411, 12495392, 11832528, 12270853, 12021221, 12127923, 12383572, 12433762, 12040440, 11901047, 12142111, 12429158, 12074792, 12084594, 11821454, 12453969, 11911720, 12108857, 11907289, 12121731, 12470133, 11786452, 11781276, 12360457, 12020263, 12074820, 12021222, 12175635, 11815511, 12417537, 11926853, 11821459, 12063368, 12090862, 11851538, 11937179, 11937178, 12501222, 12038948, 11926787, 11877357, 12103293, 12105163, 11772700, 12464950, 12108850, 11798370, 12503706, 12089229, 11853793, 12387962, 11807147, 12384409, 12118962, 12488411, 11896110, 11830607, 11897434, 11886010, 12032111, 11802748, 12417888, 11923490, 12365877, 12037150, 12377089, 12020485, 12200354, 12419297, 11823268, 12183309, 12450962, 11903027, 12472325, 12091192, 12453950, 12217597, 12196422, 11926786, 12105831, 11870013, 12449165, 11896097, 11982446, 11997270, 12123336, 11804984, 12419298, 11799368, 11896120, 12351463, 12038884, 11806971, 12063523, 12074791, 12153923, 11919304, 12166584, 12490683, 12091330, 11870012, 11929760, 11784875, 12413331, 11874927, 12180224, 12153376, 11874817, 12186794, 12480424, 12192015, 12047964, 11772973, 12145220, 11790656, 11844826, 11784873, 12074410, 12416946, 11956263, 12360456, 12039932, 12020484, 12090747, 11897647, 12103284, 11788216, 12453952, 11772961, 12175666, 12438289, 12204509, 11914249, 11969276, 11879861, 12377083, 11926936, 11741362, 12383573, 12470125, 11839621, 11741360, 12163429, 11802751, 11980684, 11872547, 11967600, 11815493, 12078913, 12176916, 11983451, 12044534, 12149204, 11815495, 12475466, 11827919, 12432041, 12133656, 12087012, 12456232, 11874944, 11997277, 12185439, 11969278, 12038916, 12423985, 12404219, 11904654, 12480854, 12063530, 11956124, 11969274, 11877261, 12142116, 12449158, 12221048, 11866009, 11998988, 11923489, 11874940, 12077088, 12235071, 11879161, 12153826, 12189224, 12177098, 11806983, 11896119, 12118025, 11786587, 11772908, 12095381, 12495391, 11919306, 11991875, 11955537, 12435254, 12020184, 12076871, 12123405, 11997274, 12081985, 12505222, 11897986, 12373475, 11923049, 11854391, 12241873, 11994699, 11927522, 11919119, 12472326, 11911726, 11864920, 12091193, 12204495, 12433513, 11901039, 12438295, 12006327, 12435255, 11779724, 12390947, 12453948, 12399345, 12169075, 12063524, 12490682, 12406823, 12010887, 12473255, 12117879, 11790236, 12127922, 12190367, 11939866, 12105164, 12108885, 12241778, 12020338, 12204499, 12020302, 12209102, 12444863, 12045168, 12503697, 11772907, 12358339, 11861267, 11869844, 12423984, 11879108, 12087008, 11959766, 11809253, 12163427, 12052800, 12044376, 12010875, 11896091, 12114041, 12415444, 12016098, 12020336, 12208797, 12208796, 12186604, 12231498, 11965280, 11943259, 12196421, 12038910, 12424162, 12468478, 12383665, 12379552, 12234231, 12350190, 11978336, 11964340, 11788212, 12270864, 11985913, 12374491, 12425704, 11991913, 12460093, 11829698, 12145222, 12449166, 12076551, 11839712, 12435256, 11822921, 11772188, 12037147, 11901040, 11981007, 11910343, 12241832, 12243636, 12411222, 12153824, 12020337, 12464949, 12403690, 12503709, 11849859, 12153969, 11844507, 12503692, 12460869, 12373467, 12189228, 12133654, 11956122, 12453954, 12421890, 12417542, 12392830, 12401733, 12045167, 12118024, 12065562, 11994249, 12074777, 12034651, 12181401, 12133653, 12445410, 12390953, 11888584, 11898004, 11898005, 11821453, 11980683, 12105157, 11879860, 12038945, 11922646, 12106937, 12386042, 11842297, 11965273, 12183307, 11785993, 11779284, 12470127, 12074773, 12472324, 11900495, 11754710, 11772695, 12153829, 12123399, 11896093, 12105159, 12412691, 12198659, 11940541, 12454840, 11965272, 11799385, 11823073, 11809180, 12105828, 12074779, 12417880, 12091203, 12433766, 12480851, 11904656, 11988242, 12208795, 12362011, 12225715, 12204862, 12186798, 12358335, 12444180, 12390061, 12505224, 12160589, 12020141, 12039927, 12117873, 11790702, 12480357, 12021225, 12243915, 12056707, 11978675, 12057987, 11907288, 12393618, 12030727, 12076217, 11772871, 12030736, 11815428, 12177099, 12075057, 12431971, 12392836, 11889009, 11809642, 12105827, 12036860, 12028981, 12149201, 12186600, 12453852, 12374520, 12444179, 11869839, 11786572, 12425705, 11755292, 11980557, 12169076, 12447868, 12217993, 12449179, 12479768, 12438291, 12239258, 12374874, 12297845, 12065555, 12076869, 12234948, 12185427, 11914248, 12395333, 12231488, 12200550, 12200551, 12364304, 12070114, 11929950, 12153966, 12079902, 12029413, 12362006, 12087018, 12493658, 11996479, 12105833, 12000813, 12202326, 11978684, 12475452, 11850333, 12065553, 12011121, 12021219, 11772893, 12057986, 11812070, 12153835, 11907654, 12365881, 12386035, 11956265, 12209104, 11863352, 11911721, 11871363, 11978685, 12175665, 12063536, 12354441, 11779723, 12196418, 11814370, 11825138, 12016180, 12359681, 11960538, 12411355, 12089223, 12149235, 12039487, 12475451, 12094249, 12384411, 12171964, 12379544, 11779753, 11821457, 12185428, 12011127, 11994701, 11994246, 12196367, 11927516, 12151469, 11978683, 12370217, 12359676, 11786526, 11781274, 11823074, 12376442, 12406842, 12351481, 11870157, 11865815, 11897445, 11874928, 11826422, 12377965, 12393661, 12354473, 12414200, 12166558, 12241831, 12021220, 12123333, 12167681, 12464951, 12056936, 12063371, 11961147, 12176950, 12196433, 11809183, 11911755, 11870242, 12401731, 12419743, 11814372, 12473557, 12475469, 12076550, 12185445, 12202659, 12234505, 12076867, 12044531, 12087138, 12103285, 11788564, 12241650, 12393567, 12196364, 12225709, 12065268, 12198695, 11788220, 12193353, 12383667, 12006323, 12103264, 12084604, 12473256, 11829701, 12454833, 12170269, 12126818, 12074774, 12106936, 12087139, 11804989, 11996608, 12427649, 11777359, 11919228, 11755279, 11863118, 12047962, 11964279, 12479763, 12393621, 12090980, 12399343, 11991872, 12020139, 12466508, 11983754, 12401734, 12039820, 12044126, 12225716, 11927527, 12163426, 12504397, 12118026, 12412675, 12413372, 12114036, 12171961, 12202661, 12118027, 12433761, 12383985, 11986409, 12460092, 11918909, 12175626, 12453951, 11773168, 11896092, 12042192, 11755289, 12177109, 12416944, 11839710, 12209085, 12090863, 12427648, 12076220, 12358327, 12209270, 12447511, 12020178, 11950803, 12447527, 12142108, 12433764, 12117878, 12359649, 12504395, 12042191, 12042201, 12166585, 12081987, 12411225, 11866010, 12011136, 11815507, 12204875, 12475460, 12456849, 12386036, 12186515, 12393614, 12397189, 12165641, 11939868, 12241774, 11804987, 12010906, 12094254, 12379550, 12202657, 11863090, 12038931, 12417543, 12354424, 11773155, 12075744, 12196363, 12107673, 12324553, 11788218, 12427406, 12475454, 12395330, 11998992, 11986955, 12145241, 11869855, 11854116, 11980678, 12149202, 12176795, 12075743, 12202665, 11773179, 11896107, 12377641, 12409326, 12118018, 12149301, 11870177, 11844818, 11870180, 11849865, 11907659, 11812553, 11870017, 12153962, 12225726, 11956118, 12370220, 12373468, 12105160, 11842293, 12446055, 11849868, 12049860, 12049858, 11923038, 11871364, 12457784, 11839708, 12225711, 12418945, 12150649, 12085364, 12098725, 12373267, 11773176, 12029409, 12103287, 12177103, 12142304, 12074399, 12411356, 11822926, 12045164, 12447512, 12243916, 11826416, 11932472, 12171952, 11914254, 12198700, 11982444, 11799376, 12217604, 12039485, 12200684, 11840286, 12055580, 11934726, 12230419, 12028976, 12029403, 11819000, 11907286, 12373472, 12351592, 12354426, 12114234, 11956258, 12183351, 11980997, 11786571, 12045163, 11897644, 12431965, 11804978, 12185425, 12074403, 12365880, 11876663, 12150648, 11988250, 12038935, 11895821, 12226149, 11827924, 12038917, 11829697, 11788219, 11976245, 11955844, 11851576, 12241656, 11991909, 11799030, 12191749, 11923161, 12077033, 12130606, 12183305, 12361896, 12488405, 12351602, 12202662, 11969277, 12447861, 12228196, 12234950, 12379549, 11779741, 12011120, 12359651, 12365879, 11996616, 11870165, 11981766, 12011122, 12231497, 12431972, 11773166, 11830461, 12196359, 12377962, 12488409, 12177096, 11929799, 12431962, 12196361, 11901037, 11755284, 11755291, 12165639, 12359854, 11919241, 11877267, 12075736, 12409329, 12145792, 11919231, 12196334, 12361449, 11785996, 12377959, 11773150, 12011126, 11896100, 12202284, 12149294, 12373268, 11786577, 11869846, 11777361, 12470180, 11988245, 12351482, 12417538, 11786454, 11832527, 12076231, 11984518, 12446059, 12450951, 11854123, 12204014, 12153837, 11997276, 12077039, 12429544, 12039482, 12398829, 12108859, 11773161, 12113263, 12208800, 12356634, 12079900, 12241657, 11985906, 12117397, 12151468, 12117882, 12117883, 11821469, 11827923, 12045171, 12208222, 12242074, 11898006, 12118965, 12377799, 12105840, 12090979, 12412676, 12023995, 11956045, 12386040, 11967597, 12427412, 11804991, 12480850, 11956264, 12029412, 12234955, 12169073, 11779264, 11890840, 12409542, 11991908, 12011124, 12009719, 12029406, 11911756, 11790211, 12209278, 12458987, 11897984, 12354470, 12196335, 12234946, 11965274, 12209274, 12234956, 12377086, 12208225, 12351491, 11889006, 12473551, 11843308, 12215131, 12450955, 11741361, 12351591, 11927515, 12081988, 12353945, 12063520, 12121721, 12360461, 11772706, 11772897, 12020486, 12242072, 12123409, 12091194, 12504399, 11815505, 12093059, 12065558, 12370214, 11986138, 12049861, 12132977, 12169503, 12110818, 11914258, 12020181, 11815513, 12016099, 11842306, 12387959, 12412696, 11943263, 12377957, 12449159, 12453953, 11991884, 12204013, 12166555, 12373264, 12464959, 12417881, 12383565, 11849851, 12110823, 12166571, 12383570, 11923046, 12475462, 12444833, 12070058, 12121726, 12361440, 11994702, 11996473, 11986125, 11869849, 11955846, 11969282, 12127431, 12358325, 12209099, 11956255, 12443589, 12374512, 12069449, 12400562, 11755283, 11772910, 11819002, 11991886, 12241713, 12086757, 12070007, 11960536, 11919227, 12377958, 11826413, 12015391, 11889011, 11967608, 11890845, 12411221, 12460865, 12454841, 12130483, 12473544, 11976244, 11955536, 11806988, 11986408, 12358334, 11773175, 12351464, 11856794, 11788558, 12484708, 12074810, 12196420, 11865814, 12192016, 11876665, 12095383, 12147533, 11802750, 12504396, 12234230, 12009718, 12032117, 12200355, 12032113, 11844850, 12097537, 12351606, 12239138, 11895822, 12119249, 11867107, 11909785, 12118963, 12213943, 11872551, 12401755, 12197792, 12225725, 11792138, 12084605, 12142117, 12110838, 12063369, 12208801, 11915041, 12377810, 12098721, 11807146, 12167683, 12241833, 12324552, 12451007, 11814373, 12030735, 12433510, 12379064, 12147372, 12606177, 12598344, 14673036, 14662554, 14657064, 12930925, 12663564, 12540643, 12556542, 12584366, 12882862, 13679527, 12909068, 12510038, 12610033, 12524411, 12532112, 12727707, 12953084, 12832297, 12761364, 12902363, 12774016, 12840089, 12477703, 12932592, 14627785, 14500439, 12700373, 14623733, 14578869, 12695317, 12944341, 14679271, 12695148, 12657225, 12923621, 12663565, 12562653, 14633951, 12801944, 12727155, 12972483, 12766103, 12953086, 12932590, 12651043, 12957423, 12904519, 12578431, 12928310, 14557148, 12456380, 12767952, 12580689, 12668982, 12488289, 12562658, 12598350, 12853354, 12763375, 12963672, 14602878, 12891541, 12759479, 14662245, 12761365, 12873857, 12957419, 12621133, 12621132, 12890841, 14568269, 14609894, 14614166, 12770854, 12502473, 14551341, 12783932, 12840090, 12406832, 14563575, 12502668, 12695318, 12510037, 14609886, 14563572, 12821233, 12912783, 12766100, 14523142, 12500203, 12897740, 12842953, 12547847, 12876091, 12633543, 12562649, 12609944, 12578487, 12759322, 12642847, 14563595, 12714022, 12551867, 12535831, 12651051, 16946913, 12721247, 12972521, 12594313, 12766094, 14559891, 12614089, 12606734, 12606735, 12654606, 12954574, 14673038, 12810695, 12697882, 12881382, 14645424, 12583945, 14644892, 14557857, 12695269, 12704369, 14662571, 12714526, 14499682, 12767365, 12859157, 12767651, 12832242, 12692051, 12594104, 14615440, 12511452, 14519710, 12810489, 12610044, 12860943, 12798560, 13678916, 14656921, 12706934, 12566370, 12660058, 12591750, 12628944, 12810429, 12766099, 12704348, 12743557, 12957433, 12816740, 12912807, 12860904, 12393680, 12860586, 12944340, 12893645, 12662006, 12732600, 12860772, 12799402, 12912857, 12578870, 14568853, 12583947, 12588271, 12807696, 12615792, 12547874, 12515739, 12668495, 12598069, 12952843, 12562576, 12502673, 13129985, 14519709, 12684359, 12702620, 12932593, 12882841, 12592331, 12939213, 14613735, 12869359, 12821234, 12743579, 12883493, 12882850, 12588269, 12488300, 12912780, 12667722, 12525530, 12765426, 12917228, 12524408, 12714026, 12832241, 12801946, 12743136, 12598214, 14615106, 14563591, 12860899, 12590903, 12660387, 12667024, 14662257, 12742278, 12695272, 14557355, 12802484, 12560228, 14683659, 12628948, 12610030, 12562575, 12860725, 12618501, 14662572, 12762352, 12952839, 12824094, 12915593, 12531794, 12893649, 14633804, 12971959, 12972523, 12947054, 12881389, 14581425, 14604932, 14680736, 14630756, 14568740, 12912716, 12511175, 14570732, 14662633, 14594754, 12636462, 12851867, 12885809, 12706917, 12759324, 12531578, 14581438, 12771115, 14665656, 12583942, 12756159, 12805334, 12663600, 14557150, 12912816, 12610003, 12900300, 14522567, 12589354, 12511176, 12406840, 12729425, 14625336, 12853193, 12595976, 12577151, 14668456, 12947065, 12583944, 12917299, 14609783, 12480560, 14668455, 14568898, 14638565, 12972470, 12774158, 14585938, 14551096, 12535810, 12746252, 14645636, 12946971, 12941725, 12714348, 12867606, 12611838, 14578240, 12840088, 12742287, 12957426, 14563577, 12634227, 12759480, 12584228, 12714472, 12727767, 12906979, 14499225, 14657888, 14610016, 12963676, 12759292, 12730120, 12634224, 12952249, 12891533, 14557154, 12541156, 12695290, 12578430, 12636461, 14613739, 12920042, 12578439, 12610049, 12531809, 12525508, 12806612, 12529346, 12737857, 12842371, 12939228, 12706932, 14673053, 12633544, 12560274, 12570111, 12975252, 12771007, 12570120, 12932605, 12538427, 14504728, 12637459, 12488303, 14644853, 12860783, 12393732, 12805106, 12663340, 14514589, 12488294, 12598351, 12919771, 12712239, 12580681, 12575975, 12729423, 12796070, 14523462, 12600908, 14551680, 12663712, 12393605, 12697878, 12684745, 14607440, 12672309, 14724827, 12592333, 12727150, 14645314, 12538428, 12562652, 14679270, 12639206, 12885748, 12614088, 12746365, 12970142, 14570722, 12922963, 14563580, 12631652, 12912714, 12622671, 12966125, 12637608, 12966127, 12865375, 12668512, 12575969, 12727395, 12842372, 14642682, 12941721, 12660054, 12788569, 12586806, 12538431, 12771113, 12771114, 12952998, 12966123, 12525233, 12719279, 14630439, 12506181, 12814712, 12609940, 12610046, 12672732, 14612479, 12570114, 12847482, 12788572, 12762347, 12859158, 12900341, 14645308, 13129987, 12532114, 12515745, 14621092, 12589351, 12882849, 12724125, 14662581, 12742878, 12925455, 14563343, 12930926, 12589361, 12547542, 14600188, 12774014, 12695287, 12649124, 12970130, 12642046, 12598068, 12767658, 12480610, 12829989, 12560436, 12684358, 12882864, 12521969, 14578226, 14609903, 14645311, 12747879, 14581254, 12689976, 12946968, 14506120, 14597456, 12570122, 14563749, 14514588, 12796071, 14621090, 12876093, 12633548, 12559938, 12566365, 12692056, 12637400, 12859160, 12716811, 14680725, 14504182, 12860578, 13678871, 13678870, 13678869, 13678868, 12709466, 12944338, 14517164, 14519708, 12642637, 14519707, 12963561, 14499232, 12682721, 12713767, 14659770, 12585949, 12684363, 12663237, 12899584, 12456382, 12589536, 14612477, 14578253, 12829670, 12932604, 12783912, 14522530, 14568892, 12766124, 12665996, 12706935, 12531775, 12813116, 12667723, 12598065, 12517464, 12570112, 12832314, 12642846, 14657066, 12713766, 12600913, 12907008, 12847070, 14644854, 12851279, 14670885, 12679215, 12970133, 14602435, 12773645, 14570950, 12869456, 14609883, 13678872, 12633189, 12941675, 12777270, 12532113, 12559939, 12763982, 14614169, 14592997, 12865276, 12577156, 12637625, 14504184, 12540640, 12896934, 12406833, 12878742, 12810426, 12668699, 12525531, 14575968, 12580705, 12771112, 12904517, 14627786, 12832308, 14609896, 12663573, 12919990, 12570118, 12706919, 14507948, 12867607, 14506118, 12766095, 12944570, 12406891, 12871686, 14559955, 14512395, 12834314, 12594112, 12855524, 14625334, 12506030, 12912787, 12876092, 12695153, 14610472, 12892984, 14600187, 12704355, 14657879, 12904324, 14511925, 12743146, 12824211, 14621086, 14534335, 12477770, 12891544, 12897996, 14570719, 12612897, 14530224, 12952272, 12837810, 14680739, 14575972, 14534336, 12809451, 12957421, 12513038, 12813175, 12531808, 12813117, 12915429, 12777279, 14564379, 12657224, 12972528, 12767656, 12651048, 12897990, 12697850, 12849674, 12628946, 12851876, 12560435, 12560434, 12529662, 12975259, 12840087, 12736280, 12805333, 14517188, 12512035, 12570110, 12904518, 14597591, 12915605, 12586622, 12766123, 12697887, 12952257, 14514591, 12742282, 12882865, 12578875, 12562650, 12529459, 12637609, 14523461, 12732605, 14572747, 12426230, 12529344, 12502652, 14585254, 12560431, 12734132, 12885681, 12502651, 12952833, 14615442, 12909072, 14638545, 12896935, 14623806, 12679222, 12707240, 12637460, 12949712, 14514570, 12756157, 14604931, 12923619, 12740337, 12885836, 12411282, 12663595, 12668989, 14557356, 12847067, 12801957, 12801953, 12824205, 12579420, 12665995, 12580687, 12805340, 14581245, 14645637, 12797502, 12939216, 12576315, 12954742, 12704366, 12562581, 12885685, 12709468, 12882842, 13678928, 12610006, 14578244, 14578238, 12663723, 12721256, 12578880, 12528027, 12920037, 12788993, 12716810, 12642049, 12912859, 12585827, 12612903, 12927929, 12506176, 12529343, 12529345, 14499226, 12829678, 14519752, 12663728, 14563582, 14633947, 14530858, 12591745, 12796137, 12665490, 12706922, 14563581, 12573372, 14551301, 13678973, 12885800, 12515744, 12566364, 12729424, 14633959, 12671888, 12972476, 12954579, 12845429, 14530859, 12802025, 12913100, 12763985, 12529349, 12802027, 14633817, 14645423, 12925453, 12546607, 12763376, 12699951, 12575967, 14581268, 12813170, 12732606, 12633545, 12565779, 12871685, 14623802, 14564361, 12705984, 13678971, 12598346, 12727766, 12559941, 12860733, 12777271, 12932382, 14512873, 12551868, 14568987, 12727154, 12601075, 12610022, 12540788, 12531776, 12714021, 12672313, 12557135, 12766091, 12853587, 12893646, 14583577, 12505859, 14499676, 12849654, 14680078, 12829988, 14504114, 12900301, 14667741, 12456381, 14562117, 14657866, 12954578, 12907482, 12623916, 12672860, 12577159, 14676042, 12502670, 14609904, 12917302, 12671882, 12477710, 12716807, 12515743, 12879235, 12765424, 14563743, 14680080, 14514497, 12814710, 12637463, 12885812, 14581436, 12521968, 12556541, 12748244, 12801736, 12559940, 12853194, 12540786, 12510039, 12721260, 12517199, 12944571, 12759389, 12571256, 12947063, 12610195, 12952263, 12610193, 12970783, 12802487, 14500259, 12689847, 12958051, 12881261, 12847065, 12788301, 12806613, 12505809, 14519753, 12830007, 12954743, 12832240, 12589345, 14657874, 12532101, 12505817, 12897994, 14504122, 12941710, 12502672, 14563574, 14630678, 12663588, 12660386, 14644860, 12767733, 13678873, 12622581, 14522476, 12578516, 13678914, 12826636, 12456509, 12954744, 12734133, 12566359, 12826431, 14504060, 12615625, 12783913, 12668365, 12907484, 12577155, 12622686, 14656841, 12766127, 14563578, 14563576, 12814685, 12952258, 12690049, 14607447, 12925454, 14514498, 12663724, 12856119, 12881395, 12506171, 12637464, 12610178, 12721240, 12853351, 12829663, 12697871, 12586799, 12721259, 12663707, 12775735, 12860938, 12743142, 12860964, 12586793, 14581419, 12807937, 14638556, 12851275, 12775734, 12941723, 12547850, 14642690, 12906976, 12912814, 12957441, 12791655, 12831816, 12797494, 12648967, 12897995, 12695226, 12958082, 12796026, 12574046, 12610038, 14578242, 12529091, 12562651, 14662576, 12804927, 12573274, 12610185, 12611835, 12622584, 12697883, 14576731, 12686036, 12628949, 12697798, 12802023, 12525526, 14633812, 12709465, 14500440, 12835212, 12788576, 12663231, 12560428, 12566361, 12796127, 12525515, 12506163, 12912782, 12682719, 12503978, 12730861, 12649101, 12774003, 12742923, 14581272, 12853344, 12885805, 12875938, 12393571, 14662267, 12860945, 12663710, 12875936, 12765427, 12493646, 14585635, 12743147, 14610048, 12775739, 14512398, 12651033, 12663440, 12821551, 12517462, 12535822, 12972485, 14604933, 14644967, 14644966, 12763986, 12774159, 12958109, 12860959, 14662569, 14656913, 12586796, 12874191, 12886239, 12939210, 12502654, 12637462, 14633803, 14598248, 12721239, 14679156, 12525510, 12610040, 12697853, 12721253, 12821546, 12538422, 12663719, 12393429, 12697866, 12637470, 12885837, 12702524, 12829675, 12963704, 12506170, 12902441, 14551299, 14525804, 14610012, 12628943, 14519751, 12915604, 12900339, 12717396, 12525522, 12805326, 12702550, 12805327, 14551293, 12668651, 12767648, 12637461, 12742869, 12610182, 12971972, 14517785, 12947070, 12954577, 12874182, 12796126, 12560443, 12610188, 12837811, 12829674, 14594748, 14551237, 12783911, 12891538, 12902452, 12750166, 12559860, 12821239, 12684362, 14680731, 14563596, 12622605, 12562588, 12958052, 14504183, 12813118, 12885747, 12842377, 12742806, 12575964, 14625264, 14610474, 12665498, 12847496, 12882847, 12885803, 14585251, 12721238, 12830373, 12704354, 12824212, 12697884, 12488296, 12598340, 12837710, 14662570, 12663706, 14563339, 12576316, 12535809, 12970132, 12743129, 12547889, 14612478, 14683655, 12742981, 12789224, 12570939, 12847475, 14593039, 12521974, 14514569, 14585939, 12821232, 12551863, 12796139, 14519754, 12727703, 12832310, 12885807, 12557133, 12743150, 14522473, 14568737, 14582907, 12965981, 12885745, 12610173, 12834313, 12721261, 12480612, 14659772, 12547849, 12699950, 12515749, 14695409, 12569144, 14550694, 14523139, 12515740, 12615634, 12600905, 12525509, 12646667, 12856118, 12882844, 12743562, 12765436, 12873814, 12919776, 12716806, 12802024, 12711467, 12727401, 12763981, 12774010, 12532097, 14662573, 14572748, 12944569, 14585937, 12562577, 12968087, 14598251, 12775730, 12654603, 12695149, 14673047, 12598341, 12590905, 12829672, 12944062, 12515747, 12939207, 14644852, 12860576, 12606175, 12891539, 14525878, 12598347, 13679822, 12965939, 12505808, 12742280, 14643272, 12600912, 12932612, 12767649, 12917230, 14633813, 12557136, 14559889, 12804926, 12610009, 12798559, 12965978, 12860582, 12642834, 14695410, 12899586, 12949718, 14559892, 12742881, 12897739, 12957432, 12608443, 14500804, 14500805, 14642684, 12766097, 14585250, 14633807, 12875940, 14581257, 12788302, 12783161, 12610021, 12906966, 12824459, 14681504, 14638564, 12612902, 14559886, 12874270, 12804929, 14523141, 12512025, 12832248, 12900303, 12932938, 14563573, 12610036, 14499675, 12529661, 12881376, 14578243, 12963590, 12882446, 12671890, 12925458, 12843002, 14581415, 12517808, 12651035, 12824095, 12849673, 12663594, 12528021, 14642699, 12821556, 14604928, 12912865, 14568851, 12562571, 12970204, 12727705, 14557145, 12511172, 12773333, 12456508, 12860778, 12837712, 12660056, 12649125, 12503977, 14532316, 12569142, 12477757, 12697867, 12767646, 12592328, 14614165, 12732601, 12742873, 12621135, 12589529, 14522478, 12774015, 12575962, 12867608, 12816824, 12941676, 12955185, 14724812, 12759295, 12671884, 14656837, 12837711, 14643269, 12679210, 12826434, 12742978, 12547870, 14578246, 14610160, 12610037, 12742999, 12468439, 12642354, 12742979, 12909071, 14550702, 12702522, 12855528, 14514574, 14652238, 14623807, 12829991, 12684357, 12668363, 12832336, 12551865, 12742279, 12668364, 12915606, 12684361, 15215154, 15138203, 15315996, 14988283, 15611489, 14724302, 15163774, 15102998, 15141043, 15111373, 14711909, 15096406, 15358024, 15111514, 15193678, 15349912, 15122768, 15190136, 15148063, 15041116, 15520363, 15082469, 15319242, 15082488, 15591335, 15466769, 15351753, 15111342, 15121646, 14512295, 14679118, 14998841, 15057738, 15300580, 14711911, 14724301, 15249354, 15082593, 15082209, 14726421, 14712285, 15308515, 15014864, 15361383, 14672887, 15479685, 15140776, 15140780, 15479686, 15526058, 14702424, 15564545, 15351750, 15057894, 14665611, 15184222, 15184237, 15123495, 14706957, 14998845, 14672612, 14720531, 15483279, 15284252, 14998609, 14747203, 15385657, 15102997, 15028824, 15226169, 15542294, 15150305, 15014181, 15229304, 14757602, 15534159, 15261920, 15607398, 15288482, 15451153, 15169696, 15121648, 15220227, 15312862, 15065835, 15464318, 15151953, 14724304, 15037535, 15051755, 15066943, 15234403, 15199092, 15310785, 14732754, 15364318, 15464314, 15494348, 15023828, 15611488, 15063441, 14693962, 15542517, 15208587, 14999298, 15569835, 15277416, 14699490, 14699491, 14769785, 15234425, 14987883, 14665609, 14720527, 15184207, 15042289, 15249347, 15143082, 14647884, 15302780, 15172406, 15451902, 14973216, 14751924, 15361496, 15465973, 15282204, 14767435, 15197210, 14757609, 14769686, 15051639, 15093884, 15210591, 15120803, 14699004, 15531767, 15516696, 15064022, 15020604, 14999693, 15514406, 14982911, 14747230, 14757687, 15313950, 15537905, 15093870, 14762036, 15569893, 15537681, 14962967, 15492300, 15276392, 14871913, 15262838, 15342168, 15020290, 15161788, 15023882, 15178637, 15347564, 15332397, 15285981, 14701770, 15362028, 14715180, 14998848, 15313740, 14720530, 15007001, 15262663, 15313956, 15123479, 15278273, 15256423, 15545678, 15175435, 15226323, 15172773, 15037537, 15023883, 15365065, 15364688, 15145629, 15238367, 15583226, 15364675, 15226164, 15014183, 15256389, 14707022, 15514382, 14747227, 15131005, 15152059, 14662708, 15028365, 15492340, 15184276, 15459300, 15361500, 15480981, 15183621, 15269313, 15184286, 15161789, 15356547, 15210738, 14691036, 15569840, 15464320, 15071126, 15492337, 15451915, 14600809, 15176685, 14982865, 15184278, 15548438, 15480327, 15063421, 15262827, 15459218, 15514409, 15039412, 15519006, 15117983, 15237079, 15596636, 15385922, 15064028, 15547180, 15358033, 15520321, 15451764, 15039413, 15351765, 15520361, 15507582, 15514344, 15172774, 15358690, 15533851, 15567011, 15085323, 15143081, 15504763, 15069044, 15169686, 15489085, 15261932, 15172423, 15183620, 15351191, 15094269, 15610805, 15117982, 15325832, 15325831, 14762776, 15302637, 15183623, 15548447, 15514407, 15534360, 15226332, 15213207, 15213206, 14657226, 15071604, 15572757, 14754784, 14998844, 14597482, 15265969, 14757693, 15625331, 15480326, 15023830, 14711748, 15316501, 15519010, 15459145, 15589636, 15262844, 15555666, 15589641, 15321699, 15562216, 14984920, 15539665, 14734000, 14684569, 15028348, 14504090, 15589635, 15063428, 15148146, 15547086, 14742346, 14960519, 15351166, 15277423, 15197209, 15045169, 14993084, 15496623, 14975468, 15113785, 15066880, 15464333, 14738236, 15277331, 15115713, 15466632, 14769678, 15285978, 14723988, 15358015, 14754789, 15351164, 14665607, 15470214, 15470213, 15226327, 15001462, 15258006, 14980983, 15577832, 15525714, 15570084, 15562199, 15356550, 14702269, 15477510, 15148271, 14751699, 14738235, 15083754, 14982910, 15194570, 15365075, 15337732, 15142868, 15326073, 15197051, 15063433, 14738231, 15519009, 15328329, 14752074, 15280342, 15280341, 15383514, 15364188, 15262104, 15451149, 15277317, 15220226, 15231376, 14996673, 15536108, 15262833, 15337700, 15048552, 15208209, 15001463, 15199111, 15466644, 15522471, 15048550, 15066870, 14981007, 15452189, 15536111, 15365066, 14752069, 14972424, 15353532, 14726165, 14724146, 15333479, 15039411, 15313884, 15208586, 14996776, 15173145, 15474134, 15326078, 15240438, 15333452, 15213209, 15351192, 14738791, 14693987, 14996778, 15226168, 15197430, 14987884, 15082202, 15353497, 15329426, 15509817, 15504990, 15293609, 14551147, 14975482, 15238592, 15100667, 15547166, 14684561, 15138163, 14979510, 15297305, 15611487, 15361407, 14993078, 15351756, 15100337, 15033882, 15492339, 15031238, 15210530, 15066879, 15234855, 15197199, 15562127, 15364317, 15172903, 15197018, 14744846, 14993087, 14990394, 15505095, 15302806, 15210599, 14707020, 15016485, 15562223, 15082697, 15066949, 14985966, 15138671, 15504997, 15315998, 15229100, 15492322, 15194584, 14977622, 15044205, 15016484, 15172408, 14960504, 15505016, 14693963, 15158628, 14732755, 15066900, 15326071, 15120888, 15289281, 15607401, 14722635, 15193680, 14734593, 14734700, 15582061, 15111377, 15271690, 15533865, 14718319, 15024568, 15520364, 15218994, 15113814, 15172901, 14996698, 15142885, 15172898, 14693981, 14722645, 15477437, 15082480, 15249567, 15504996, 15451900, 15363566, 14711910, 15265847, 15249568, 15306109, 15337407, 15541448, 15520346, 14563654, 15249566, 15201414, 14993114, 15596605, 15159270, 14747225, 15201136, 15145633, 15337655, 15488216, 14760130, 15288157, 15047656, 15575055, 14722148, 15289365, 15217837, 14990637, 15520997, 15238624, 14551167, 14981006, 15451146, 15238590, 14679124, 15565613, 15131791, 14747232, 14999111, 15467059, 14769684, 15082482, 15043959, 15364687, 15039128, 15237059, 14722639, 15173268, 15149751, 15193674, 15331394, 15363567, 14996675, 14973217, 14984925, 15145107, 15082726, 15514412, 14769702, 15306578, 15169793, 14693964, 15217841, 14962965, 14657228, 14732756, 15066896, 15351163, 15288159, 14683739, 15159414, 15127186, 15315995, 15466674, 15215483, 14657227, 15172900, 15361495, 15020614, 15261923, 15483282, 15205198, 15140531, 15176694, 15254060, 15199090, 15010365, 14990632, 14990633, 15142875, 15237068, 14734329, 15249570, 15191772, 14722628, 15023706, 15054036, 15310784, 14722144, 15542510, 15466635, 15520068, 15249511, 15596631, 14769679, 15464315, 15353498, 15111618, 15269312, 15504999, 15358704, 15596634, 15520052, 14990635, 15470487, 15483020, 15100679, 15542798, 15220228, 15173031, 15358018, 15547093, 15265815, 15249353, 15534166, 15519014, 15145099, 14979503, 15519008, 15069024, 14979508, 15176682, 15302800, 14683738, 15123524, 15351759, 15173147, 15084372, 14747229, 15093869, 14966079, 14762784, 15161785, 15220231, 15277409, 14975462, 15197202, 14726422, 15285973, 15111338, 14747233, 15562184, 14754790, 15051633, 15229059, 15337703, 15023708, 15519020, 15302194, 14718325, 14722203, 14985485, 15111532, 14712346, 15361375, 15479936, 15382120, 15093878, 15582309, 15572533, 14656755, 15218999, 14722206, 14744971, 15044639, 15547164, 15111525, 15451901, 15519629, 14693983, 15007110, 15500893, 14981107, 15197198, 15265788, 15246726, 15358014, 15247339, 15044206, 14709577, 15451222, 15477504, 15051778, 15112033, 15550582, 15365074, 15065832, 15483019, 14656750, 15364319, 15234399, 15194574, 15241356, 15470215, 15081650, 15293606, 15313942, 15480324, 15590952, 15572537, 15066946, 15239087, 15479889, 15218993, 15188169, 15284257, 15238454, 14975474, 15276394, 15304466, 15173273, 15483018, 15531765, 15381645, 15337691, 14667521, 15234410, 15284269, 14679127, 15172464, 15056514, 15374846, 15143088, 15069048, 15356657, 14762035, 15047649, 14739212, 14525770, 15020310, 15342805, 14962524, 14684415, 15479682, 15329425, 14738232, 15140778, 15194252, 15381516, 14722640, 14966096, 14715189, 15238368, 15451795, 15306531, 14734594, 15536109, 15148065, 15041109, 14701781, 15269315, 15565569, 15583115, 14998852, 15020609, 15611507, 15582060, 15332389, 15173146, 15041108, 15337403, 15492298, 14967728, 15226326, 15023707, 15302193, 15238420, 15289374, 15312855, 15083762, 15312856, 15337695, 15289275, 14701778, 15123534, 15557380, 15245813, 15526045, 15249569, 15377574, 14962523, 15505091, 14991092, 15190137, 15078794, 15246205, 15033254, 15533869, 15082581, 15100202, 14685663, 15388612, 15065831, 14767445, 15465995, 14684584, 14702423, 15363569, 15078803, 15557371, 14979498, 14693980, 15536429, 15107485, 14975458, 15451221, 15111341, 15351769, 15313360, 15353506, 14975461, 15084614, 14722034, 15051771, 15353542, 15207952, 14732749, 15345498, 15226337, 15175436, 14966092, 15367413, 15611506, 15602019, 15451899, 15249319, 15316515, 15520425, 15082468, 15026400, 15210571, 15054570, 15452182, 15184205, 15479678, 15625332, 15578510, 15371578, 15057741, 15282351, 15282352, 15336451, 14996676, 15598915, 14671645, 14990643, 15474274, 15007010, 14586494, 14962821, 14757591, 15169798, 14679122, 14701773, 15459217, 15084618, 14722033, 15547181, 15277255, 15249584, 15385923, 15284262, 15197192, 14722035, 15459211, 15084616, 15367414, 15474700, 15169685, 15533855, 15023808, 15545293, 15530627, 15226212, 15128893, 15128894, 15362027, 14767589, 15254051, 14766718, 15262734, 15541838, 15037444, 15569910, 15353499, 15120821, 15120808, 15466766, 15026402, 14992985, 15254053, 15496622, 15065825, 15028356, 15464329, 15163773, 15505001, 15107313, 15516697, 15520341, 15302102, 15150204, 15135594, 15520310, 15607399, 15060765, 15219008, 15229064, 15172395, 15325833, 15381643, 15547162, 15364322, 15028558, 14769704, 14976046, 15285969, 15057739, 15152060, 15567010, 15567009, 15246213, 15226339, 15590950, 14718331, 15172468, 15294856, 15492306, 15036669, 15051770, 15289492, 15364842, 14762779, 14733999, 15470212, 15337642, 15337807, 15306665, 14693671, 15276393, 14996859, 15466785, 15596568, 15220251, 15337803, 15578509, 15371577, 15161783, 14724157, 15082472, 14724129, 15242867, 15564229, 14744821, 14966078, 15126313, 14744822, 14960530, 15604181, 15253920, 15377573, 14703543, 14726370, 15520061, 15070635, 14684587, 14679117, 15531766, 14754783, 15117994, 15226336, 15142880, 15010373, 15365070, 15349901, 15480323, 15197196, 15514373, 15570076, 15611510, 14998850, 14990647, 15254052, 15169807, 15226321, 14679114, 15197195, 15570070, 15547184, 15459210, 15117980, 15356567, 14981005, 15466681, 15310772, 15292384, 15277322, 15237062, 15150303, 15565568, 14722642, 15142871, 14734517, 15313938, 15226325, 15121645, 15630469, 15465981, 15302787, 15337799, 15289368, 15465984, 15169694, 15342174, 15210522, 15480990, 15534165, 15477492, 14998846, 14996777, 14992980, 15220255, 15464183, 15030987, 15345600, 15197140, 15547161, 15326072, 15385655, 15039131, 15465986, 15007003, 15489091, 15047641, 15161773, 15494583, 14992978, 15170529, 15208585, 15084368, 14754791, 14693984, 15356304, 15315964, 15031241, 15136304, 15191774, 15590953, 15241347, 15381650, 15064026, 15380963, 15519660, 14967716, 15238591, 15234416, 15474136, 15197206, 15126252, 15590951, 15364868, 15208597, 14999109, 15056522, 15525713, 15070666, 15459142, 15451148, 15172407, 15479896, 15570081, 15122749, 15289213, 14988829, 15520323, 15500894, 15247338, 15262845, 14720526, 15585732, 14630619, 15123533, 15289245, 15465985, 15121405, 15516665, 15519028, 15364875, 14718266, 15151947, 14972420, 15474697, 15477398, 15306112, 15159285, 14757608, 14751925, 15364316, 15321697, 15308514, 15541449, 14709579, 15173148, 15121650, 15249317, 15185303, 15477438, 15122774, 15536093, 15380964, 15577823, 15100340, 15342804, 15023878, 15328325, 15333483, 15028823, 15150203, 15306594, 15326069, 15284112, 14713918, 15234398, 15234407, 15193686, 15063430, 15572756, 15193677, 15100668, 15028375, 14967774, 14984917, 14706955, 15541832, 14988822, 15313741, 15183553, 14727018, 15247354, 14760111, 15459137, 14767999, 15033251, 15277417, 14742305, 15289215, 15220247, 15047650, 14714106, 15197019, 15148064, 15514398, 15466673, 14749454, 15082207, 15063432, 14711749, 15351752, 15030985, 14993085, 15249351, 14979502, 15138674, 15308513, 15485934, 15036672, 15151958, 15145093, 15136504, 15505128, 15466678, 15220241, 15557412, 15541450, 15197190, 15070662, 15007086, 14738230, 15001324, 15138160, 15474698, 14970111, 15477411, 12907440, 15308521, 14993134, 15480319, 15117843, 15572717, 15006999, 14993128, 15208594, 14985268, 14982912, 15096395, 15226331, 15550579, 15130978, 15130977, 14976099, 15220230, 15489105, 15589640, 15172892, 15199033, 14760122, 15131566, 15601638, 14992983, 15057903, 15277410, 15572534, 15249503, 15351193, 15254055, 15172894, 15464258, 15057896, 15215482, 15285975, 15558712, 15207950, 15474696, 15562200, 15464185, 15016643, 14982884, 15562202, 15300570, 15548777, 15492301, 15556955, 14999696, 15571828, 14960741, 15246645, 15500895, 15006828, 14739186, 15284267, 14747210, 15353533, 15353496, 15127196, 15161787, 14769685, 15565603, 15315997, 14757690, 14999442, 15169706, 15158627, 14693982, 15313947, 15508085, 15158629, 15545669, 15975991, 16198982, 16154018, 16083710, 16263857, 16286164, 16009873, 15983302, 16143117, 16143705, 16382061, 15983299, 16120856, 15990220, 16230074, 15996811, 15855574, 15130899, 16172439, 15774256, 15849222, 15242865, 15867115, 15838676, 16087696, 15782318, 15358623, 15653032, 15615803, 15598949, 15695501, 16020489, 16141449, 15928070, 15640320, 15528283, 15345500, 15998750, 15920039, 16012942, 16249542, 16043680, 15753242, 16287768, 15668285, 16234567, 15598942, 16143742, 15824291, 15657157, 15812073, 15809460, 15213095, 15944423, 15753114, 15867120, 15867114, 15901604, 15735187, 16061916, 16330721, 16108036, 15915460, 16018426, 15710957, 15994719, 15897530, 16325031, 15936605, 15940616, 15860486, 16143120, 16354890, 15734772, 15760929, 15616227, 15930418, 15625221, 16162882, 16260639, 16172458, 15677780, 16126936, 15911704, 15616239, 15929960, 15750041, 15870415, 16186474, 16005336, 16055451, 16198843, 16143741, 16186422, 15708844, 15788496, 16275368, 15738537, 15758000, 16271642, 15893182, 16338449, 15919685, 15939717, 15939920, 15930421, 15992694, 16030275, 15659500, 16314629, 16009795, 16145011, 16175618, 15723979, 16159631, 16083789, 16053970, 16157938, 15659722, 15872201, 15831901, 16135835, 15850631, 15728165, 16186262, 15925082, 16049208, 15817596, 15901862, 15806000, 15923574, 15681524, 16103233, 16118380, 15598977, 16291601, 15888499, 15734615, 16216961, 16115831, 15708698, 16148284, 15754197, 15843667, 16186434, 16159624, 15851401, 15809365, 16109780, 15809403, 16009860, 15732092, 15927965, 16159831, 15563637, 16159829, 16125588, 16168278, 16258083, 16157934, 16157935, 16084255, 15286005, 15867173, 15735198, 15883262, 16034009, 16126716, 16354893, 15750043, 16123483, 15677807, 16217004, 15955903, 15668261, 15843666, 16368945, 16330736, 16314551, 15956137, 16306540, 16123474, 15920062, 16183697, 16306522, 15855578, 15855557, 15908660, 15911729, 16123481, 15767642, 15766995, 15837945, 15958804, 15956037, 15838068, 15754377, 15502112, 16264042, 16325694, 15987918, 15713943, 15734614, 15713944, 16039333, 15668765, 15999255, 15963399, 15908647, 15967846, 15843449, 15696076, 16087950, 15800321, 15677624, 16112299, 16257793, 15993231, 15735214, 15867104, 15661417, 16278358, 16286586, 15703894, 15983290, 16263866, 15486338, 15940132, 15699477, 15738352, 15659723, 15625211, 16195288, 15585538, 16093252, 15939844, 16077050, 15809408, 16186468, 16186437, 15825072, 16012932, 16084254, 15582252, 15867200, 16053952, 16087941, 15951542, 16354891, 15734768, 15933223, 16306563, 16143730, 15618036, 16030027, 15834703, 15735184, 16159849, 15630104, 16365466, 16160130, 16135834, 16314615, 16316966, 15677775, 16160131, 15936869, 15860509, 15668287, 15677777, 15764724, 15741476, 16186436, 16203961, 15950716, 15735185, 16061501, 16325695, 16144892, 15632335, 15774253, 16182897, 16186280, 16145024, 16135832, 16055868, 15800226, 16083704, 15713945, 15930065, 16275867, 16282177, 15687314, 15735183, 15769918, 16264181, 16246948, 15708879, 15716289, 16275811, 16284203, 15723981, 16061764, 16330719, 16120857, 16314648, 16129801, 15635109, 15494430, 16170182, 15987919, 16093465, 15829533, 16192582, 15911727, 15716208, 16306539, 15618054, 16325696, 15699292, 15897341, 15793181, 15860863, 15657339, 15653017, 15625361, 16110022, 16093466, 16043752, 16365212, 15837944, 15684319, 16243090, 15632336, 16112300, 15808751, 15642765, 16382063, 15681520, 15741528, 15716206, 15728647, 15659494, 16361622, 16314640, 15821119, 15665005, 15618034, 15766817, 15625212, 16039775, 15735182, 16186270, 16333028, 15753241, 15920051, 15901634, 15990782, 16135638, 15930077, 15665326, 15863798, 15677608, 15837982, 16192478, 15737962, 15851601, 16338450, 16186596, 16226162, 15591112, 15857851, 15994396, 16271643, 16162880, 16159843, 15800317, 15994003, 16339217, 16249527, 16150741, 15774238, 15855580, 15793171, 16230723, 15545661, 16264054, 15809394, 16214599, 15872029, 16143698, 16210049, 15911730, 16167129, 16195293, 15657326, 15824294, 15671432, 15716561, 15741530, 15831910, 15671428, 16125590, 16023513, 15916468, 16210250, 16365469, 15939927, 15867184, 15809368, 16210048, 15734619, 16083781, 16043737, 15983288, 15657462, 16081445, 15767536, 16009690, 15956632, 16285944, 15766818, 16249547, 16203960, 16014597, 15800139, 15941800, 15936419, 15598978, 15998749, 15642701, 16195287, 15699284, 16301340, 15733717, 15781099, 15738298, 15951574, 15867125, 16168782, 16143696, 15657324, 15924983, 16172203, 16002204, 15708690, 15774776, 15910951, 15753273, 15800330, 15829574, 16159816, 16150764, 15886380, 15778231, 15992643, 15766997, 15997021, 15734608, 15983242, 15557136, 15723448, 16186467, 15517159, 15983390, 15855572, 15855571, 15956121, 15811982, 15735188, 15677610, 15812624, 15994660, 16310551, 15769967, 16043724, 15866309, 15800328, 15735200, 16287956, 15671427, 16291982, 15836887, 15800160, 15812629, 16361638, 16287955, 16286163, 16337459, 15781429, 16143129, 16159618, 15837996, 15625213, 15811981, 16221778, 16246996, 15731291, 15640336, 15531753, 16043733, 16214830, 15794968, 15897310, 15863377, 15630071, 15937679, 16009794, 15758007, 16263860, 16330680, 15978925, 15883267, 16009958, 15767643, 15805998, 16110017, 15837983, 16053953, 16014883, 16014882, 15940460, 16174858, 15791697, 16043738, 15811983, 16332728, 15809384, 15735186, 15681525, 15564311, 16204662, 15837256, 15863796, 16230722, 15699058, 15834056, 15664241, 15741468, 15987706, 15956130, 15692069, 15876471, 15640321, 16210047, 15728645, 15642700, 16219926, 16116154, 16264159, 16339094, 15936423, 15718314, 15937294, 15821120, 15956633, 15860519, 16098419, 16098418, 15923670, 15998952, 15591466, 15677609, 15927974, 16123223, 16022953, 16139656, 16257339, 15908661, 15625369, 15925814, 15735116, 15741225, 15766814, 15830009, 15811868, 15837246, 16198847, 16159821, 15857930, 16085693, 15677559, 15710792, 16132885, 16186281, 15668282, 15677803, 15691863, 15992641, 15735130, 15653019, 16321761, 15863376, 16330739, 15766828, 16040785, 15717206, 15817602, 15764620, 16306520, 16287954, 15893170, 16275936, 15687232, 16105975, 16285948, 15894598, 16304073, 16053954, 15388510, 15867118, 15908668, 15616225, 15753530, 16046440, 15557131, 16126331, 16000354, 16116152, 15718308, 15542794, 15947284, 16293867, 15990777, 15950714, 15668467, 15827314, 16260696, 15769772, 15867121, 16116055, 15708696, 16287792, 16129844, 16126933, 15793191, 15699275, 15774778, 15820998, 15637138, 15741486, 15824079, 15983303, 16286549, 16285942, 15677607, 15963388, 16203942, 15920040, 15795344, 15800312, 16287769, 16022954, 15994714, 15809387, 16014595, 15972451, 15598939, 16043683, 16103252, 15746106, 15738450, 15643700, 15851642, 15710761, 16154019, 16157829, 16361631, 15940143, 16105981, 15947280, 15940615, 16339095, 15677701, 15829577, 16226614, 15825070, 15920045, 16330684, 15753526, 16040619, 15855433, 16046530, 15972565, 16230721, 16000352, 15963401, 16022966, 16110036, 16325044, 15677776, 15972866, 16024131, 16199843, 16371630, 15755765, 16250032, 15741226, 16137822, 15642859, 16139655, 16082736, 15940125, 15994143, 16317669, 15940624, 15939850, 16316969, 15964445, 15655130, 15855226, 15800157, 16186435, 15840623, 16150805, 15765388, 15708886, 16250037, 15271773, 15615797, 16037102, 15843668, 16278360, 16239176, 16135725, 15696079, 16148283, 15900007, 15930042, 15623545, 15982798, 15657341, 15985559, 15930039, 16139122, 15766816, 15677778, 16129815, 16168780, 15695500, 15880608, 15716204, 16258094, 15774493, 16139121, 16192600, 16136168, 15867204, 15987916, 16192479, 15956126, 16034041, 15655136, 16361623, 16286607, 15998890, 16151466, 15677599, 15867182, 16096751, 15964448, 15778243, 16087945, 15723977, 15920042, 16022970, 16199841, 16162034, 16098428, 15657401, 15956631, 16132888, 15914575, 16301359, 16022955, 15677600, 16250039, 15647576, 15860855, 16079371, 15542792, 15994144, 16055865, 16275879, 15800228, 16301343, 16107485, 15857840, 16234523, 15738542, 16110019, 15735119, 16023511, 16043828, 16306519, 16129365, 15893193, 16267322, 15860850, 16291063, 15860853, 15998903, 15946977, 16000353, 16098423, 16182898, 16170158, 15983319, 16159809, 15893183, 15711977, 15871011, 15765387, 16009798, 15911734, 15625210, 15994710, 16199834, 16132897, 16210665, 15708100, 16337447, 16093464, 15734767, 15754200, 16087825, 15911731, 16061734, 15640324, 16195905, 15885294, 15825069, 15774791, 16135469, 15811979, 15837247, 15837244, 15837245, 15805999, 15684204, 15812080, 15684301, 15837702, 15809382, 15897552, 15774788, 15928071, 16105990, 15824252, 15726658, 15973519, 16192581, 15546895, 15929954, 16126738, 16136167, 15723319, 16116148, 15660392, 15987917, 15972867, 15888797, 16083709, 15639293, 16030279, 16344384, 15661415, 15967849, 15710219, 16123473, 15826716, 15955900, 16110025, 15598944, 15598945, 15955899, 16192591, 16051944, 15961759, 16314626, 15860854, 15939922, 15774775, 16275937, 16230677, 16192577, 16314627, 15681529, 16293869, 15939923, 16293863, 16293868, 16172456, 16361616, 15928305, 15452187, 16286173, 16319330, 15625582, 15940147, 15883215, 16246947, 15677604, 16170170, 16330788, 16006443, 16198981, 15659503, 15837249, 16183654, 16186417, 15800310, 16148021, 16099293, 16198768, 15774254, 15990196, 15862409, 15650862, 15608311, 15997019, 16143734, 16192584, 15925816, 16287765, 16275869, 16228176, 16143747, 15826746, 16154016, 16258098, 16144890, 16246997, 16267249, 16159869, 15809409, 15956038, 15677779, 15800019, 15767288, 15618041, 15872036, 16246976, 16192592, 16352794, 16380589, 15863374, 15967567, 15925083, 16258091, 15767638, 16260701, 15774257, 15770007, 15805993, 15604156, 16085692, 15718325, 16022960, 16319343, 15930064, 16108073, 15911864, 15946978, 16110020, 15618066, 15722369, 15888698, 15928285, 15778240, 15758009, 16039331, 15815895, 16009687, 16103032, 16308382, 15615766, 16096304, 15604157, 16183652, 15860827, 16096286, 15911537, 16306060, 15929958, 15774255, 15883209, 15837968, 15908654, 15734771, 15699480, 15967851, 16203930, 16230487, 16230678, 15616226, 16135471, 15994145, 15616230, 16129793, 16256868, 15657405, 15625365, 15800329, 15659491, 15713598, 15817604, 16361624, 15911866, 16135464, 15961764, 16170175, 15908667, 15923572, 16192580, 16110015, 15837997, 15860487, 15681523, 15911865, 15870437, 15625368, 16087956, 15767639, 15825071, 15812075, 16135475, 16051958, 15718316, 16135479, 15718317, 15677625, 15800308, 16027437, 15741571, 15750189, 15860867, 16145047, 16291981, 15961761, 16258088, 16043728, 16170180, 15928300, 15699482, 15781750, 16231970, 16293862, 16275814, 16263208, 15625214, 15625202, 16143126, 15766996, 15728810, 16157604, 15705690, 15956010, 15855581, 15741228, 15839740, 16192448, 15677598, 16186288, 15837248, 16226164, 15893181, 15800144, 16246987, 15851647, 15967861, 15677794, 16382062, 15972728, 16286540, 16136166, 15867103, 15860852, 15951544, 16365207, 16234518, 16234514, 16139140, 15753902, 16087822, 16210253, 16159847, 15863797, 15930063, 15994720, 15914552, 16099292, 16083782, 16204002, 16141440, 15793183, 16306548, 15733716, 15922478, 16043675, 16023510, 16256861, 16310552, 15680454, 16203934, 16275807, 15917384, 16264158, 15757960, 16214598, 15721471, 16216821, 16264046, 15837984, 15774786, 15572587, 15598943, 15708700, 15738534, 16257791, 15894098, 15630062, 15867849, 15687128, 15967762, 15872028, 15738535, 16291984, 15516532, 16246964, 15699265, 15750042, 15824212, 15867181, 16085658, 15625373, 16014884, 16177249, 16182896, 16105989, 15644543, 15735115, 15855594, 16055779, 15716285, 15618072, 15805990, 16325036, 16087795, 15635110, 15684311, 16227416, 16043723, 16275379, 15923569, 16281077, 15850630, 16249417, 15774240, 16129366, 15911536, 15684287, 15992629, 16110023, 16009955, 15647189, 15840878, 15699266, 16288118, 16258087, 16115830, 16344401, 16143732, 15608312, 15829494, 16260697, 15618073, 16093255, 16159645, 15753541, 15917280, 15990772, 16264052, 15968010, 16098435, 15661416, 15738453, 15867404, 15618027, 15753115, 15968009, 16123484, 16139123, 15699479, 15838067, 16081469, 16306551, 15684293, 15860521, 15983301, 16249540, 15983318, 16022951, 15699297, 15710956, 15863811, 15668361, 15367427, 15753264, 15486066, 16123505, 16186278, 15817595, 15618070, 15572589, 15802340, 16087821, 15618067, 15862408, 16287791, 15851648, 15716559, 15929962, 16087818, 15625220, 16239177, 15890685, 15994288, 15716198, 15766834, 15677813, 15800147, 15767619, 15855570, 15817593, 15625362, 15870414, 15976380, 16286543, 16186600, 15994387, 16267077, 16151021, 16236737, 16236738, 16337461, 15992652, 16121218, 15637558, 16199835, 15928281, 15951310, 16330790, 16286541, 15867860, 15728225, 15958805, 16304572, 16107896, 15794970, 15956124, 16043829, 15825065, 16034044, 15388511, 16123472, 15920044, 15944424, 16143731, 15745977, 16325039, 15920071, 16183692, 16157621, 15659490, 15961217, 15963389, 15914548, 15761020, 15860518, 16204601, 15983312, 15983308, 16043677, 15736995, 16186274, 15674354, 15744348, 16051946, 15726659, 15699296, 16330592, 15728228, 15972865, 15685543, 15829527, 15998891, 16032424, 15963391, 16243088, 16166613, 15983311, 15452188, 16087943, 15653024, 15271770, 15809366, 16221780, 16227417, 15701909, 15701910, 16022950, 16043734, 15738536, 17027729, 17012045, 16849762, 16980115, 16628625, 16401736, 16732007, 16524973, 16682384, 16843184, 16525137, 16946176, 16816220, 16757491, 16630997, 17174704, 16401691, 16807413, 16421364, 16567607, 16861254, 16492717, 16401810, 16799983, 16390905, 17003291, 16983056, 16816222, 17003313, 16540554, 16873779, 16825492, 16840741, 16556697, 16670205, 16651506, 17116766, 16352812, 16274834, 16600984, 16603599, 16766588, 16495395, 17135584, 16877662, 16905695, 16980315, 16847013, 16099784, 16339287, 16344278, 16484504, 16704894, 16317014, 16780997, 16585472, 16931634, 16949492, 16949482, 16373904, 16750678, 16440357, 16904224, 17074945, 16476809, 16389204, 16894074, 16192451, 16840727, 16249215, 16971664, 16423848, 17145988, 16908795, 16894061, 17161267, 16943402, 16557539, 16407509, 17087947, 17159011, 16781360, 16390894, 16952543, 16513860, 17101314, 17151157, 16510744, 16648332, 17035649, 16507859, 17010793, 16567600, 16533938, 16611949, 16818837, 16162678, 16239622, 16818924, 16505266, 16150852, 17074942, 16954134, 17045905, 16432672, 16520474, 16446341, 16505432, 16357330, 16443859, 16936143, 16495393, 16782911, 17105795, 16945766, 16873785, 16682309, 16627509, 16410446, 16452593, 16914703, 16837126, 17039234, 16707395, 16426697, 17050891, 16698410, 16820551, 16760210, 17088142, 16954488, 16754923, 17174182, 16534036, 16554313, 16873786, 16732009, 16870658, 17065671, 16950290, 17012684, 16847010, 16446313, 16875985, 16774980, 17015814, 16424444, 16469404, 16461878, 16882959, 16630999, 16461817, 16520326, 16820633, 16648324, 16126801, 16496307, 16507846, 16714187, 17003304, 16732017, 16622259, 16272449, 16950285, 16908790, 16551713, 16522454, 16412856, 16814647, 16697316, 17101637, 16754835, 16520435, 17045910, 16413875, 16357838, 16530578, 16935870, 16401690, 16513861, 16860459, 16966688, 16361020, 16497822, 17010792, 16887854, 16534040, 16322642, 16905220, 16530506, 17124018, 16697307, 16717292, 17159017, 16801459, 16793845, 17015867, 16801628, 16905785, 16490911, 16670135, 16815141, 16750985, 16750984, 16554525, 16310924, 16751008, 16904537, 16707748, 16481636, 16481635, 16672699, 17088509, 16636023, 16791665, 16545638, 16461961, 16601957, 16214831, 16769917, 16807277, 17135583, 16890832, 16782488, 16446328, 16943401, 16943400, 16782912, 16556888, 16567814, 16317024, 16890770, 16877657, 16971718, 16818861, 16567825, 16401473, 16648042, 16873787, 16505499, 16543470, 16510747, 16551966, 16418408, 16949484, 16936184, 16847150, 16531616, 16765759, 16452559, 16882678, 16804151, 16769915, 16818864, 16864762, 16670409, 16801564, 16754934, 17012628, 17114212, 17130189, 16831981, 17038453, 16864756, 16524979, 16455831, 16403928, 16636210, 16382112, 16926353, 16847014, 16682573, 15919678, 16442211, 16571610, 16714767, 16675783, 17174180, 16731539, 16887480, 16476878, 16715326, 17074952, 17146008, 16801571, 16455835, 17174705, 16876666, 16815137, 16537737, 16631000, 17105759, 16820611, 17108343, 16781361, 16895945, 15994216, 16443871, 16877650, 16682725, 16717170, 16966588, 17194197, 17151158, 16728480, 16870915, 16936149, 16675705, 16790698, 16908486, 16418308, 16603634, 16317012, 16914701, 17065639, 16495394, 16567823, 17015865, 16751015, 16622262, 16966367, 16769916, 16507847, 16174762, 17050889, 16551764, 17015404, 16455186, 16377643, 17179478, 17130190, 16820564, 16953000, 16621868, 16687205, 16339157, 16556690, 16697728, 16957145, 16950289, 16505512, 17019539, 16769988, 16896862, 16785477, 16581404, 16760197, 16648499, 16735702, 16877725, 16630938, 16849754, 16513877, 16769912, 16628640, 17088500, 16788808, 16870914, 16224108, 16757723, 16595757, 16757722, 17179058, 17101639, 17141704, 16554527, 16698413, 16818810, 16517051, 16330464, 17040925, 17023435, 16935685, 16732018, 16801461, 16505262, 16675326, 16769748, 17161243, 16630941, 17077374, 16597660, 16782489, 16543297, 16679331, 16912265, 16772624, 16415377, 16822845, 16731999, 16820548, 17161727, 16891328, 16829296, 16740529, 16751018, 16707508, 16793810, 16801577, 17101640, 16458133, 16705107, 16306137, 16750496, 16980380, 17030174, 16478899, 16644639, 16997664, 16413878, 16641119, 16735333, 16461871, 17105798, 17130196, 16782490, 16636211, 16936160, 16790460, 16735334, 16807257, 17142257, 17035647, 16585434, 16684985, 17056608, 16763247, 17090560, 16565124, 16801514, 16966497, 16754825, 16585435, 17045903, 17151159, 16916809, 16826385, 17146023, 16818923, 16772250, 16772251, 16785475, 16567606, 16754827, 16636212, 17006921, 17050873, 16585662, 16702472, 16609086, 17190893, 16373903, 16864724, 16357331, 16636217, 16373910, 16537725, 17084252, 16443860, 16461864, 16510466, 16949486, 16415095, 16443607, 16426990, 16908918, 16488376, 16644634, 16505497, 16877454, 16837676, 16176940, 16413877, 16814643, 16847290, 16979011, 16754727, 16344336, 16831962, 16798391, 16801565, 17003295, 16407508, 17088141, 16464988, 16625008, 16361021, 16439370, 16698412, 16461133, 16890769, 17046465, 17130197, 16908789, 17084249, 17084250, 16339289, 17098084, 16390893, 16290907, 16481389, 16545747, 16505498, 17088514, 16446318, 16606646, 16820547, 17046467, 16567811, 17050586, 16423873, 16389212, 17050890, 16418497, 16952539, 16820589, 16434427, 17045894, 16979005, 17116917, 16957147, 16537665, 16762627, 17133475, 16525138, 17079759, 16428266, 17182989, 16609087, 16968715, 16399150, 16606651, 16899486, 17105853, 16574999, 16291600, 16766116, 16822812, 16968874, 16720830, 16481637, 16982941, 16368878, 16820261, 16757699, 16618403, 17116767, 16704893, 16307026, 17074946, 17172616, 16514112, 16801333, 16816226, 16546538, 17101936, 16401670, 17045906, 16537662, 17124017, 16675486, 17101847, 17151364, 16484584, 16219797, 16791662, 16505510, 16998093, 16648043, 16772623, 16760295, 16507585, 17012630, 16455838, 16904542, 16966504, 16754834, 16750996, 17058231, 16966687, 16641168, 16857993, 16769782, 16495392, 16820580, 17023730, 17145742, 16461125, 16269428, 17074775, 16871570, 16840441, 16894077, 16636167, 16840742, 16505413, 16707747, 17043338, 17130343, 16899775, 16772388, 16682420, 16713070, 16781986, 17088511, 16531614, 16531613, 16820579, 16472588, 16452558, 16814206, 16757701, 16622261, 16750497, 17157654, 16344495, 16574937, 16487622, 17021320, 16373887, 17010798, 16534012, 17133499, 17194188, 16428249, 16510465, 16816224, 17151173, 16957901, 16344276, 16814645, 16567838, 16782719, 16365183, 16446335, 16651498, 17098754, 16361531, 16556892, 16507849, 16443858, 17018460, 17000941, 16373894, 17074943, 17084264, 16644631, 16682531, 16880458, 16390899, 16443885, 16803941, 16540614, 16644785, 16267070, 16377775, 16188920, 16096330, 16618399, 16840744, 16507848, 16216827, 16452592, 16870913, 16798389, 16461865, 16487841, 16611656, 16782927, 16905024, 16452557, 16382125, 16310929, 16557544, 16687712, 17146066, 16728716, 17082779, 16563608, 16520413, 16990384, 16990383, 16825290, 16732029, 16394300, 17061020, 16609069, 16844491, 16820555, 17126719, 17001467, 16713921, 16979010, 16478902, 16476851, 17133471, 16968876, 17116939, 16914458, 16679433, 16946168, 16921042, 16641126, 16709935, 16723614, 17192538, 16391217, 17174201, 16781381, 16887481, 16983120, 16387410, 16443892, 16537575, 16921035, 16308341, 16476713, 16428253, 16782715, 16282339, 16702588, 16801576, 16611657, 17130195, 16651507, 17179457, 16373898, 16953009, 16330517, 16687711, 16899485, 16478882, 16424872, 17000947, 16807438, 17087951, 16534013, 16766594, 17010806, 16449487, 16717173, 16467234, 16467233, 16467232, 16391215, 16567815, 17055945, 16946304, 16540613, 16873668, 16707509, 16772625, 16401468, 17159021, 16954358, 16443886, 16446323, 16895944, 16554526, 16275661, 16644641, 16322118, 16814663, 17088127, 16540546, 17012266, 16936164, 16954519, 16452585, 16675781, 16421365, 16449486, 16469753, 16556691, 16423847, 17075115, 16443872, 16421418, 16389216, 16807414, 17088140, 16510745, 16513871, 16765760, 16622140, 16622139, 16412857, 17151174, 16574546, 16932345, 16627862, 16807259, 16875978, 17030175, 17108342, 16467546, 16707546, 17101319, 16267074, 16387596, 17030655, 16741212, 16520433, 16648504, 16516075, 16608859, 16421419, 16603609, 16971719, 16530576, 16455695, 17027730, 16625009, 16467348, 16630986, 17065669, 16648506, 16352807, 16777857, 16585663, 16754838, 17088135, 16682304, 16790517, 17167137, 16531618, 16971717, 16391299, 17075109, 17093249, 16254269, 16356945, 16079172, 16648508, 16754728, 16423872, 16781376, 16505520, 16941701, 16651490, 16530503, 16890599, 16941685, 16492716, 16822992, 16310932, 16461819, 16513858, 16963731, 16476694, 16807444, 16835378, 16717293, 16636341, 16966690, 17114657, 16809729, 17008686, 16365178, 16505409, 16782922, 16575010, 16782926, 16682727, 16943538, 17075117, 16877734, 16314619, 16549837, 16505427, 16877722, 17135639, 17135646, 17033039, 16311275, 16648511, 16921039, 16782910, 16877719, 16446329, 16410447, 17028105, 16710029, 16412864, 16455834, 16567830, 16809734, 16390890, 16415300, 16517274, 16838151, 16606645, 16837678, 16365182, 16814669, 17030687, 17145722, 16877656, 16621870, 17157651, 16545748, 16386671, 16707516, 16432056, 16543549, 16449474, 16946178, 16382122, 17003312, 17047215, 17008704, 16740528, 16826390, 16627543, 17167134, 16815138, 16894064, 17133492, 17101852, 17040924, 17003293, 17119142, 16501948, 16861255, 16505178, 16697308, 17011942, 16908768, 16603606, 16648502, 16269419, 16516077, 16648512, 16481638, 16449523, 16902151, 16567604, 16754836, 17060336, 16397129, 16825288, 17045888, 17161256, 17003303, 16953007, 16497687, 16714768, 16476804, 16904540, 16782921, 17093248, 16613932, 16735709, 16467544, 16980316, 15972298, 16720619, 16500914, 16339220, 16873425, 16740530, 17151160, 16458130, 17058225, 16953019, 16461867, 16352815, 16849748, 17015810, 16750696, 16702582, 16505439, 17174178, 16849680, 16446331, 16943537, 16670385, 16484699, 17075122, 16446336, 16446342, 17050867, 16877730, 16549833, 17050869, 16371411, 16983112, 16782917, 16648503, 16921047, 16595780, 16882940, 16330675, 16520442, 16946127, 16446330, 17060387, 16344321, 16391155, 16954475, 17000910, 16899475, 16418496, 16575013, 17105848, 16921043, 16254147, 16648318, 16478746, 16908773, 16338019, 16449478, 17021318, 17021319, 17030169, 17012299, 16517548, 17141703, 16737346, 16282244, 16820557, 16490836, 16682575, 17012691, 16962881, 17088316, 16968848, 16437139, 16641127, 16516083, 16549639, 17151163, 16801465, 16949481, 16522836, 16754631, 17053881, 16387583, 17030680, 16319952, 17030521, 17093247, 16843170, 17095725, 16380544, 16449476, 16873669, 16754935, 16651474, 16982943, 16534045, 16801464, 16394299, 16394298, 16630994, 16675812, 16885182, 16980114, 16714186, 16571878, 16396977, 16717175, 16697759, 16873803, 17030231, 16467543, 16520452, 16503464, 16935686, 17040926, 16622266, 17138219, 17005948, 17116941, 16389183, 16888080, 16606972, 16651509, 16550369, 16452103, 16815148, 16585078, 16446322, 16949487, 16513872, 16917115, 17075113, 16983107, 17084257, 16760445, 16493102, 17060388, 16531619, 16971716, 16390895, 16461866, 16368883, 16908767, 16908788, 16150858, 16432076, 16835383, 16507801, 16531621, 16757720, 16864757, 16608860, 16861341, 16644621, 16618947, 16630937, 16782716, 17077105, 17088125, 17179108, 16514109, 17119141, 17119140, 17098085, 16401777, 16760270, 16390901, 16921034, 16873788, 16505417, 16144800, 17110281, 16935946, 16618953, 16421366, 16093267, 16682542, 16525139, 16505513, 16567837, 16432086, 16254268, 16648320, 17000937, 16314338, 16516074, 16990386, 16750701, 17116916, 17043337, 16772244, 15975966, 16801566, 16439720, 16818832, 17003306, 16954359, 16611950, 16415304, 16507855, 16487836, 16840747, 16373890, 16754920, 16682315, 16567812, 16717081, 16439444, 17174195, 16963472, 16427490, 16822094, 16543298, 16843192, 16814646, 16446317, 16458131, 16412876, 16818836, 17098762, 16644630, 16950363, 16905783, 16412866, 17045887, 17010794, 16458136, 16585455, 17038641, 16682305, 16762982, 17105794, 17105757, 16546539, 17046466, 16766587, 16511633, 16567772, 16387937, 16990385, 17130203, 16567820, 17060379, 16424446, 16946177, 16412870, 16648333, 16936159, 16497685, 16162683, 16449522, 16510462, 17101615, 16488895, 16670131, 16627548, 17079762, 17015818, 16697324, 17179056, 16606763, 16360972, 16449473, 16505505, 16978997, 16434136, 16219709, 17174179, 16818865, 16791663, 16796402, 17006925, 16729326, 16567610, 16908915, 16675324, 16820546, 16750681, 17019548, 16949494, 16860698, 16616557, 16641396, 16501947, 16968847, 16418463, 16474028, 16670132, 17188959, 17146025, 16490909, 17449504, 17975184, 17398307, 17634488, 17208639, 17551146, 17392551, 17466225, 17267907, 17202560, 17502536, 17267790, 17229769, 17846399, 17272357, 17287478, 17573387, 17336650, 17351283, 17568029, 17652648, 17485161, 17846273, 17920906, 17950848, 17110457, 17679728, 17384931, 17084960, 17671288, 16815864, 17140723, 17416453, 17135217, 18021879, 17388667, 16956917, 17114190, 17728216, 16990605, 17678730, 17267789, 17768270, 17403972, 17984403, 17187417, 17256759, 17320744, 17984136, 17698683, 17785765, 17967821, 17328985, 17374742, 17283299, 17532088, 17998490, 17294169, 17202550, 17974932, 17202547, 17363756, 17448371, 17244684, 17372174, 17241861, 17463418, 17475739, 17600039, 17872493, 17502630, 17658395, 17596475, 17671253, 17290041, 16984942, 17475734, 17517761, 17241875, 17161520, 17699546, 17846266, 17192353, 17662390, 17241859, 17299059, 17046964, 17470824, 17971530, 17259481, 17256746, 17890232, 17252006, 17483542, 17349681, 17728419, 17728426, 17159194, 17978289, 18083404, 17525087, 17227978, 17440161, 17442999, 17403977, 17179052, 17170721, 17676985, 17456839, 17204317, 17165022, 17692742, 17984186, 17367658, 17379290, 17050563, 17942579, 17673448, 17320942, 17585106, 17475736, 17389290, 17327325, 17693652, 17568028, 17670909, 17611232, 18056902, 17289685, 17707753, 17690122, 17715164, 17239709, 17478034, 16959916, 17975183, 18056903, 17158523, 17879366, 17702967, 17567623, 17921245, 17292769, 17068328, 17536083, 17993364, 17658394, 17869373, 17971587, 17492431, 17606812, 17371783, 17548750, 17551148, 17671280, 17392302, 17560293, 17336417, 17394957, 17343981, 17604800, 17470670, 18068496, 17107988, 17586747, 17846331, 17719468, 17307761, 17923598, 17442997, 18156031, 17596577, 17353443, 17368152, 17272503, 17846310, 17544845, 17467889, 17515403, 17283347, 17517855, 17237474, 17502530, 17309935, 17341128, 17562667, 17257742, 17324401, 17496229, 17562951, 18025538, 17986493, 17998546, 17251237, 17412741, 17595194, 17848651, 17336720, 17602078, 17141945, 17363838, 17692748, 17240440, 17634458, 17988947, 18068495, 18003960, 17389291, 17666464, 17923601, 17227997, 17686823, 17401004, 17989384, 17607561, 17954800, 17228021, 17303791, 17383417, 17960013, 17925563, 17960012, 17209059, 17050564, 17647293, 17227784, 17664467, 17475941, 17651893, 16807260, 17515459, 17440049, 17170051, 16998094, 17287476, 17825893, 17312289, 17202549, 17327524, 17449505, 17465000, 17909123, 17475733, 17329468, 17499604, 17258855, 17519410, 16973987, 16926184, 17631952, 17548749, 17983871, 17570205, 17028126, 18154961, 17504795, 17404138, 17194902, 17947724, 17712070, 17296878, 17623819, 17341711, 17371885, 17259484, 17283363, 18165667, 17923575, 17063357, 17452544, 18077809, 17426275, 17984395, 17980734, 17199056, 17670917, 18024871, 17325262, 17586744, 17881290, 17192336, 17283264, 17551149, 17559913, 17602080, 17606957, 17869635, 17537764, 17115133, 17984375, 17317857, 17974057, 17704347, 18022023, 17280773, 17848668, 17935765, 17353040, 17267098, 17575270, 18077808, 17290056, 17620445, 17717284, 17283346, 17631387, 17291934, 17682120, 17536077, 17475737, 17630037, 17577004, 17728427, 17148582, 17664469, 17394956, 17327309, 17541059, 17179098, 17687152, 17914039, 17047001, 17675541, 17392352, 17308271, 17804843, 17350452, 17317858, 17484874, 17372175, 17298973, 17314111, 17942873, 17538170, 17482983, 17196507, 17463412, 17496236, 17240287, 17854590, 17353499, 17446306, 17563030, 17592099, 17898345, 17950857, 17170385, 17586303, 17292768, 17909140, 17707751, 17204566, 17563345, 17644511, 17920918, 17306703, 17679016, 17889242, 17988728, 17878421, 17991935, 17261662, 17848650, 18056558, 17699008, 17289744, 17485570, 17324975, 17085459, 17395677, 17938390, 17496227, 17329479, 17157434, 17199066, 17459901, 17626912, 17371886, 17192328, 17446339, 17242322, 17766284, 17911499, 17252021, 17855282, 17259186, 17804441, 17325296, 17339618, 17259189, 17707752, 17631131, 17509485, 17384434, 18073360, 17548243, 17533209, 18068514, 17387132, 17504798, 17387131, 17387129, 17679017, 17686833, 17368154, 18068515, 17878265, 17728425, 17666461, 17244834, 17392295, 17586304, 17290062, 17954801, 17329466, 17916813, 17452390, 17717203, 17156883, 17765963, 17507345, 17384435, 17909129, 17429101, 17595270, 17095745, 17785708, 17876019, 17644512, 17329467, 17473060, 17190867, 17244833, 17556718, 17507344, 17398308, 17200216, 17132678, 17283283, 17283260, 17291931, 17468359, 17620655, 17609490, 17327307, 17353373, 17692744, 17606658, 17353504, 17384437, 17488963, 17291938, 17846285, 17456819, 17387133, 17394963, 17986694, 17468345, 17577003, 17984165, 17872930, 17277036, 17254697, 17658393, 17416261, 17617272, 17560290, 17363401, 17804841, 17267793, 17964037, 17668878, 17467884, 17846260, 17617270, 17617271, 17606657, 17097217, 17652295, 18056556, 17602732, 17484872, 17541049, 18037080, 17531887, 17452676, 17925518, 17980667, 17509486, 17693179, 17071935, 17560446, 17351278, 17208602, 17600040, 18046028, 18046027, 16763053, 17301298, 17272359, 17562672, 17563874, 17456482, 17983800, 17301099, 17323051, 17664472, 17971584, 17998441, 17554116, 17526594, 17452677, 17482880, 17652313, 17582069, 17541052, 17409109, 17562955, 17142385, 17644623, 17984410, 17728357, 17595353, 18073378, 17690257, 17569681, 17335962, 17719471, 17996568, 17513173, 17084512, 17931979, 17980418, 17082513, 17988727, 17483155, 17326152, 17848673, 18025536, 17200148, 17698067, 17339566, 17717285, 17418384, 17519435, 17551129, 17825405, 17919488, 17971401, 17010507, 17538165, 17207911, 17329192, 18036446, 17363767, 17898344, 18073358, 17893311, 17392540, 17420345, 17339550, 18042917, 17434094, 17318498, 17984413, 17848609, 17440171, 17515571, 17998491, 17259641, 17919489, 17898099, 17264340, 18089866, 17196293, 17389338, 17185309, 17768267, 17728213, 17533211, 18056740, 17929300, 17681169, 17947390, 17539025, 17928597, 17456720, 17631129, 17855669, 17484870, 17499425, 17499427, 16960155, 17470822, 17213254, 17681164, 17683197, 17919717, 17964349, 17942872, 17919718, 17942871, 17919634, 17719472, 17322481, 17878474, 17563335, 17666216, 17388666, 17577015, 17513815, 17118950, 17998497, 17687151, 17327338, 17239710, 17954798, 17138955, 17394970, 17556348, 17980248, 17283268, 17306709, 17602075, 17241864, 17698729, 17053207, 17591623, 17337492, 17868802, 17894323, 17540957, 17563395, 17227995, 17560448, 17998498, 17768282, 17611204, 17897708, 17018708, 18036454, 17536071, 17631219, 17251236, 17324398, 17404369, 17698695, 17470697, 17785483, 17909141, 17470830, 17114188, 17470831, 17635889, 17872488, 17785485, 17928091, 17556347, 17954796, 17954538, 17258304, 17980251, 17071931, 17384341, 17582067, 17132720, 17404368, 17310047, 17728418, 17519278, 17429083, 17728218, 17666672, 17476008, 17562958, 17377770, 17950147, 17950856, 17998496, 17687149, 17179021, 17124249, 18048817, 17855668, 17326149, 17968020, 17251533, 17659194, 17326150, 17592080, 18032763, 18032762, 17577027, 17473298, 17890316, 17868806, 17293364, 17804680, 17314110, 17372153, 17301299, 17545185, 17187405, 17596576, 17537770, 17562666, 17903626, 17140647, 17476010, 17989382, 17502627, 17301072, 17928595, 17383080, 17350451, 17964038, 17491099, 17881751, 17298968, 17548728, 17704421, 17312304, 17349881, 17574729, 18036451, 17468120, 17846293, 17890730, 17592102, 17325260, 17925539, 17434095, 17053058, 18025444, 17166799, 17846268, 17296972, 17620494, 17626056, 17602076, 17872903, 17329409, 17634459, 17341712, 17321311, 17321310, 17674440, 17878242, 17392539, 18156032, 17239798, 17920916, 18057338, 17576868, 17482982, 17689147, 17804844, 17525141, 17998547, 17384344, 17301091, 17337504, 17543634, 17761977, 17199068, 17379850, 17011699, 17484873, 17626040, 17484865, 17548746, 17826341, 17971588, 17470850, 17592075, 17540851, 17331969, 17039354, 17728422, 17563333, 17560680, 17720019, 17369599, 17548729, 17239712, 17687131, 17855283, 17898346, 17379291, 17442905, 17394960, 17241860, 17476007, 17110021, 17950142, 17893310, 17470858, 17412737, 17122382, 17935764, 17387127, 17404163, 17634457, 17442659, 17609429, 17532523, 17505072, 17704425, 18089878, 17679661, 17611565, 17515461, 17881750, 17606955, 17468357, 17405996, 17251278, 17470851, 17298965, 17369602, 18160686, 17392561, 17350966, 17350965, 17513805, 17401014, 18039987, 17405834, 17906203, 17498584, 17762000, 17846017, 17625124, 17894303, 17692985, 17604363, 17943989, 17258718, 17560287, 17531660, 17496307, 17446340, 17502632, 17533206, 17554522, 17925548, 16942833, 17327409, 17929306, 17446337, 17629590, 17634482, 17606973, 17909199, 17878480, 17971595, 17761978, 17404152, 17634485, 17235042, 17906207, 17704423, 17351253, 17548839, 17548840, 17442998, 17513814, 17194911, 17264336, 17283364, 17146104, 17698804, 17290061, 17470860, 17634494, 18024869, 17971594, 17975182, 17267099, 17296625, 17548410, 17548731, 17595355, 18021872, 17560678, 17666462, 17451796, 17940319, 17448376, 17405995, 17197435, 17071932, 17592101, 17251530, 17251531, 17405972, 17341661, 17495988, 17698615, 18056526, 17982182, 17127704, 17634486, 17671281, 17301074, 16984929, 17538167, 18083388, 17690120, 17443000, 17132650, 17433950, 17227787, 17259500, 17923721, 17679633, 17192326, 17291851, 17389334, 17244641, 17854593, 17208600, 17208601, 17690340, 17349686, 17671254, 17228023, 17679725, 17372173, 17631211, 18089867, 17634487, 17698733, 17393526, 17971525, 17227996, 17179023, 18160685, 17699816, 17971598, 17556722, 17008536, 17687129, 16698176, 17234331, 17369574, 17485709, 17875971, 17971582, 17984132, 18048822, 17485707, 16934392, 17761981, 17597050, 17142256, 17761995, 17325261, 17404119, 17433955, 17223475, 18071169, 17494926, 17494925, 17210817, 17538164, 17264338, 17664468, 17981316, 17485550, 17634479, 17429084, 17071939, 17356040, 17785705, 17954810, 17261652, 17276176, 17507702, 17975845, 17308275, 17369566, 17374834, 17557948, 17785709, 17470735, 18089865, 17709798, 18048820, 17418289, 17434899, 17876014, 17602081, 18089875, 17110590, 17876013, 17043094, 17264337, 17079694, 17679727, 17296974, 17602086, 17599630, 17513708, 17420341, 17548730, 17513803, 17513813, 17327320, 17470863, 17989110, 17654600, 17634484, 17301297, 17921531, 17498576, 17577032, 17416851, 17475735, 18065731, 17947725, 17502577, 17681958, 17984409, 17935230, 17312332, 17502459, 17878479, 17429085, 17259188, 17954514, 17954515, 17947731, 16984938, 17923633, 17596273, 17599602, 17498580, 18094377, 17954513, 17420340, 18056557, 17363746, 17314338, 17299195, 17993347, 17351252, 17664372, 17785486, 17664470, 17846333, 17309916, 17825709, 17107982, 17507703, 18057337, 17215531, 17620510, 17709802, 17138821, 16882708, 16884844, 17003366, 17132719, 17485579, 17975181, 17420513, 17652651, 17283003, 17551159, 17984166, 17299194, 17329696, 17901415, 17434401, 17426277, 18061060, 17949807, 17980250, 17314337, 17337493, 17408662, 17548760, 17768286, 17673732, 17405999, 17208597, 16935912, 17472992, 17717202, 17556431, 17118949, 17876009, 17430981, 17308269, 17889226, 17438314, 17630036, 17321667, 17303529, 17326161, 17351275, 17562950, 17502531, 17431226, 18039994, 17110643, 17998493, 17384438, 17548761, 18068501, 17145735, 17785618, 17563023, 17141851, 17804683, 17602082, 17997151, 17267326, 18054551, 17562956, 17204725, 17215530, 17392557, 17768289, 17664376, 17670757, 17202559, 17928994, 17040921, 17485422, 17543376, 17321581, 17519409, 16984937, 17405970, 17215529, 17548726, 17686832, 17905149, 17562959, 17242315, 17538084, 17485711, 17538085, 18089864, 17307102, 17502568, 18089874, 17403721, 16899479, 17270340, 17470740, 18156012, 17298975, 17938126, 17851130, 17680654, 18094378, 17895456, 17538086, 17482343, 18003959, 18027874, 17377586, 18036453, 17846332, 17898352, 17050557, 17239711, 17502629, 17457574, 17404349, 17785487, 17351280, 17392542, 17620446, 17377767, 17682119, 17466227, 17563342, 17146635, 17310049, 17905167, 17277040, 17561494, 17141859, 17936154, 17448820, 17932102, 17433949, 17322539, 17560289, 17290039, 17596471, 17485628, 17537772, 17208587, 17919563, 17888840, 17394959, 17459899, 17898353, 17251232, 17290043, 17382827, 17382828, 17606839, 17909125, 17496226, 17389292, 16985182, 17310045, 17548727, 17347187, 17925516, 17630038, 17704418, 17502466, 17884846, 17442904, 17928045, 17418297, 17646669, 17964332, 17405770, 18046717, 17202548, 17451800, 17559152, 17909201, 17329698, 17993363, 17576867, 17526862, 17574730, 17349882, 17456483, 17464992, 17312305, 17606976, 17336652, 17616297, 17570206, 17327324, 17229952, 17291932, 17258387, 17185355, 17954806, 17309918, 17579182, 17604300, 17277042, 17251532, 17693650, 17467513, 17496286, 17765962, 17968021, 17229772, 17470380, 17110451, 17409323, 17974058, 17693178, 17325297, 17389293, 17306441, 17577037, 17692991, 16777920, 17878149, 17159193, 17159195, 18755343, 18784090, 18347003, 18207280, 18836213, 18413705, 18450604, 18550874, 18480196, 18654759, 18725220, 18692886, 18762327, 18621928, 18086726, 18722849, 18621929, 18285602, 18765484, 18420096, 18555897, 18667394, 18504252, 18542921, 18509121, 17681999, 19265453, 17875549, 18272912, 18523025, 18384230, 18448495, 18575843, 18281145, 19007597, 18718419, 18402900, 18296423, 18209013, 18283036, 18567918, 18925641, 18565853, 17989308, 18852400, 18784099, 18697216, 18332291, 18981306, 19029501, 18316661, 18706417, 18316422, 18753646, 18951617, 18848937, 18454510, 18471513, 18302293, 18000175, 18398095, 19064624, 18632147, 18086727, 18579575, 18309945, 18250350, 18180244, 18369591, 18388356, 18391133, 18486741, 18237682, 18504332, 19007589, 18056755, 18716298, 18349029, 18936501, 17909084, 18936500, 18364392, 18212316, 18387444, 18794541, 18334706, 18182617, 18718815, 18083065, 18281266, 18664560, 18809614, 18620949, 17934150, 18762589, 19017587, 18319519, 18056743, 18606913, 18165640, 18280329, 17878209, 18971491, 18192544, 18420965, 18666223, 19050192, 19047026, 18946064, 19092152, 18525041, 18269927, 18506008, 18177775, 18697192, 18602688, 18250251, 17945409, 18468543, 17785764, 18711175, 18212313, 18337600, 18191734, 18427133, 18177758, 18244953, 18955665, 18424309, 17994222, 18753638, 18022173, 18346960, 18535188, 18728281, 18237683, 18281409, 18245177, 19109574, 18848141, 18951618, 18541739, 18772061, 18272891, 18505757, 18678843, 19052124, 18556343, 18332472, 18955670, 18987098, 18421054, 18936475, 19047532, 18765162, 18268073, 18499566, 18703471, 18953051, 17885079, 18753647, 18467723, 19095129, 18458209, 18558645, 17901414, 18256071, 18216054, 17901413, 19001601, 18235052, 18809812, 18202351, 18172173, 17962204, 18550577, 18452785, 18539223, 18250349, 18371557, 18332159, 18922570, 18243498, 18715634, 19029470, 18775429, 18077217, 18617483, 18535254, 17644545, 18398079, 18349391, 18182412, 18441323, 19029466, 19029469, 18287130, 18456558, 18316137, 18355658, 17947611, 18480202, 18242141, 18845330, 18669454, 19013463, 18227370, 18848350, 18519523, 18292286, 18227359, 18687718, 18342223, 18441320, 18332052, 18974308, 18790313, 18474729, 18458145, 18852398, 18276061, 18515805, 18477782, 18796619, 19064746, 18171718, 18395326, 18469091, 18378947, 17673699, 18455221, 18430909, 18182416, 18375998, 18285589, 18824757, 18826988, 18294998, 18635256, 18162494, 18230778, 19047538, 18378631, 18334689, 18789766, 18248876, 17616556, 18669787, 18270335, 18606932, 18390493, 19066253, 18450927, 18309956, 18678836, 18339606, 18482663, 18192453, 18398105, 18953056, 18838655, 18992654, 18779236, 18214425, 18339475, 18854540, 18455961, 18079051, 19038683, 17989119, 19007694, 18955451, 18417532, 18180279, 18838638, 18420663, 18045724, 18838648, 18441005, 18936502, 18413703, 18474762, 18653654, 18508832, 19001472, 18669424, 18787222, 18227526, 18612130, 17980419, 18538469, 19047525, 18227366, 18427130, 18235048, 18503103, 19066382, 18779620, 18502280, 18424324, 19007693, 18263802, 18952612, 18172020, 18342222, 18202421, 18182665, 18845605, 18451321, 18936478, 17977931, 18272913, 18448233, 18625896, 18234715, 18296124, 18509206, 18212092, 18065407, 18195173, 18316662, 18794207, 18445842, 18316794, 18498954, 18355656, 18725489, 18703787, 18824708, 18458144, 18787216, 18498956, 19020325, 18295023, 18065424, 18216051, 18971490, 18039957, 18541793, 18592210, 18375897, 19022155, 18558641, 19088352, 19064829, 18256393, 18242415, 18572080, 18728266, 18083636, 18309940, 18313127, 19073976, 18544724, 18823656, 18823658, 18555912, 18477783, 18984888, 18926277, 18539225, 18669426, 17545193, 17980416, 18640457, 18559700, 18675689, 19088351, 18663167, 18460663, 18283204, 18094013, 18337596, 18328929, 18268067, 18502299, 18358926, 18620950, 18191732, 18572078, 18184907, 18268187, 18398080, 18207017, 18268174, 18757090, 18391135, 18640769, 18235117, 18703470, 18678802, 18511702, 18768414, 18583190, 18207461, 18387931, 18757089, 18534975, 18632543, 19095139, 18000183, 18950853, 18167405, 18697901, 18755762, 18326503, 18243516, 18374841, 18818254, 18577730, 18799552, 18094011, 18332051, 18276664, 18452779, 18827210, 18848553, 19064606, 18296121, 18981485, 18768369, 18071079, 18262041, 18757238, 18765394, 18525036, 18195202, 18502208, 18466804, 18479744, 18084015, 19075206, 18812366, 18539917, 18845667, 18268152, 18413501, 18279739, 18508290, 18485819, 18502300, 18757085, 17947341, 18765397, 19014770, 17962237, 18191684, 18055472, 18395579, 18926569, 18200040, 18940534, 18663165, 18458039, 18970976, 18381567, 18480205, 18450517, 18678610, 18809631, 18192510, 17921185, 18358927, 17967833, 18486740, 18486739, 19036425, 19012954, 18508824, 18177774, 18381903, 19059048, 18653228, 18514303, 18256069, 18424323, 18207016, 19008268, 19000581, 18242413, 18515185, 19064627, 18420662, 18056225, 18676180, 18332474, 18281656, 18172036, 18071002, 18301879, 18490527, 18626047, 18774745, 18086795, 18403102, 18367559, 17962631, 18687253, 18824462, 18057052, 18502301, 18537189, 18390971, 18973936, 18467727, 18505948, 18296421, 18487567, 18678800, 18299491, 18285604, 18458207, 18199863, 18206735, 18617492, 18037627, 18955452, 18782641, 19074218, 18439662, 18525035, 18582928, 18984887, 19029500, 18385202, 18499565, 17768173, 19014765, 18180410, 18762591, 18505951, 17890456, 19066383, 18408224, 18357434, 19001325, 18606949, 18319412, 18430910, 18281410, 18952682, 18180434, 18843118, 17986678, 17965424, 19027483, 18779612, 18711184, 18658112, 19041130, 19017911, 19001195, 18207003, 18250253, 18537187, 18374405, 18757948, 19064822, 18563389, 18614558, 17909090, 18719013, 18319414, 18165645, 19001024, 18757091, 18310262, 18458874, 18809608, 18487480, 18534276, 17938887, 18433918, 18314215, 17989709, 18086798, 18854539, 18089849, 18308250, 18256392, 17869409, 18381485, 18184957, 18316629, 18544723, 18525042, 18625622, 18280326, 18182599, 18207004, 18565968, 18184678, 18281663, 17596275, 18276619, 18467322, 18757324, 18156492, 18413309, 18996930, 18549913, 18285358, 18417521, 18375982, 18687720, 18279736, 18725223, 18297270, 18089850, 18697207, 18666235, 18541827, 18535115, 18809622, 18048644, 18614402, 18838564, 18612151, 18413496, 18565860, 18364505, 18242396, 18445839, 18069698, 17932377, 18055864, 18357436, 18458186, 17644552, 19001202, 18390970, 18206732, 18791166, 18056742, 18187561, 18832751, 18658109, 18474811, 18779447, 18210092, 18381905, 18398094, 18492867, 18539916, 18396107, 18184958, 18502278, 18402899, 18765433, 18482661, 18377975, 18332043, 18541900, 18625864, 18418571, 18299490, 18559698, 18250271, 18423628, 17968976, 18028997, 18178525, 19001508, 18065406, 18779613, 18694978, 18262712, 18562328, 17965059, 18757088, 18198942, 18946061, 18669425, 18198195, 18329126, 18467719, 18227530, 18332475, 17875551, 18596740, 18332471, 18375896, 18203764, 18537181, 18182600, 18502282, 18420099, 18272892, 18268150, 18992651, 18417461, 19095138, 18443258, 18332158, 18752330, 18723437, 18784091, 18268065, 18498953, 18235121, 18403765, 18182669, 18762476, 18316674, 18997198, 18591543, 18677023, 18823986, 18663162, 18626046, 18586174, 18805335, 18603157, 18832244, 18285548, 18794556, 18606930, 18207005, 18702962, 18559704, 19001507, 18784373, 18174038, 18245179, 18463376, 17875547, 18509120, 18502781, 18316488, 18824641, 19074946, 18782843, 18262711, 18519826, 18509204, 18458278, 18923170, 18714059, 19017589, 18468528, 18006539, 18208849, 18362366, 19101390, 18541792, 18280328, 18566340, 19055986, 18287122, 18230792, 18263874, 18765480, 18367240, 18272893, 18814906, 18398097, 18832385, 18539207, 18458204, 18595903, 18538636, 18977178, 18687249, 18627001, 18082451, 18614781, 18591555, 18441375, 18502279, 18555911, 18558642, 18718966, 18398081, 18722869, 18374840, 18158289, 18234321, 18669565, 18268144, 18509207, 18926826, 18567539, 18970966, 18505783, 18235123, 18165641, 18000167, 18519426, 17583416, 18824706, 18272916, 17938425, 18782838, 18765450, 18981357, 18523223, 17666447, 18559876, 19055987, 18503773, 17975791, 17881538, 18212288, 18479743, 18358910, 18358928, 17967831, 18443259, 18297267, 18805963, 18506005, 18695053, 18390968, 18349395, 18574157, 18487568, 18281670, 18375894, 18235122, 18506025, 18794551, 19001323, 18662969, 18509179, 18779611, 18398153, 19001327, 18467316, 18349393, 18029971, 19029421, 18854568, 18281662, 18378568, 18375898, 18309943, 18768432, 18955454, 18177756, 18684843, 18625920, 18629471, 18725484, 18417275, 18846541, 18161700, 18784101, 18617640, 18222355, 18463848, 18848554, 18270353, 18757777, 17925285, 18347350, 19029412, 18779465, 18055486, 18684008, 18672164, 17934156, 18245121, 18250126, 18308687, 18849193, 18707985, 18258986, 18752863, 18606612, 18443191, 18202420, 18539195, 18356189, 17947301, 18279948, 18852401, 18303031, 18981464, 18768944, 18276620, 18400558, 18678838, 18786480, 18502911, 18502912, 19052125, 18555896, 18314473, 18314474, 18427774, 18607562, 18794543, 18442484, 18239086, 18560003, 18653640, 18316672, 18946063, 18669464, 18768943, 18809617, 18467724, 18570306, 18474764, 18506009, 18695132, 18650512, 18482589, 18713809, 19074232, 17898015, 18272546, 18349405, 18565951, 18940887, 18454508, 18955453, 18308157, 18591549, 18762597, 18342224, 19001206, 18802161, 18420964, 19064745, 18332300, 18195176, 18411239, 18505968, 18316792, 18281659, 17947225, 18981465, 18083691, 18936474, 18854562, 18711188, 18281658, 18487565, 18558665, 18669453, 18421053, 18398154, 18458040, 18375893, 18202419, 18809612, 19038680, 18725648, 18304964, 18025439, 18375891, 18936479, 18550554, 18845665, 18606919, 18270213, 18375890, 18981463, 18202422, 18718471, 18086493, 18508296, 18393319, 18467729, 18544744, 18591401, 18061648, 18413554, 18682445, 18812325, 18276741, 18437356, 18711187, 18640939, 18227529, 18450928, 17644537, 18707986, 18334511, 18757465, 18703004, 18471517, 18497320, 17846044, 18364502, 18559703, 18207018, 18802150, 18368070, 18774746, 18077218, 18390799, 18678842, 18565859, 18427147, 18765698, 18387666, 18565884, 18606952, 18206738, 18285545, 18177776, 18799723, 17971487, 18579811, 18579812, 18156154, 18553458, 18328930, 18617486, 18955671, 18718422, 18350270, 18325386, 18503774, 18223031, 18997196, 18614482, 18282805, 18413618, 18436116, 18755342, 18467726, 18640933, 18452778, 18619971, 19064623, 18672400, 18433023, 17959861, 18698064, 18222357, 18191669, 18678792, 18593778, 18647853, 18678611, 18840838, 18824643, 18443351, 18413464, 18612156, 18282804, 18056887, 18172188, 18500419, 18647982, 18714060, 18184901, 18376000, 17875806, 18832239, 18261680, 18226581, 18802155, 18650507, 18702961, 18768415, 18000176, 18840822, 18650514, 18184902, 18096810, 18183660, 18158176, 18402906, 18216062, 18702965, 18565887, 18000649, 18687248, 17906853, 18713788, 18779444, 18583576, 18650513, 18512045, 18155284, 18468672, 19005196, 18923067, 19047545, 18296460, 19010733, 18057049, 18287602, 18086800, 18487550, 18467725, 18006892, 18510961, 18753666, 18314433, 18371559, 19033573, 18281407, 18467717, 18840821, 18323556, 18316660, 18080339, 18753639, 18378520, 18385198, 19052126, 18779616, 18413638, 18782903, 18471512, 18092150, 18474731, 19022840, 18987368, 18492953, 18436293, 18369200, 18175773, 18929239, 18185520, 18302285, 17956874, 18838727, 18276744, 18206149, 18436114, 18166759, 17962632, 18310480, 18703472, 18184893, 18695086, 19091759, 18824758, 18156614, 18931059, 18199700, 18436117, 18405951, 18443265, 18456557, 19067478, 18285565, 18755353, 17916806, 18927173, 18055869, 18198271, 18702964, 18245119, 18829872, 18423977, 17728331, 18532873, 18424307, 18394965, 18356109, 18355913, 18471515, 18079286, 18727929, 19092151, 18815396, 18256391, 17566639, 18297268, 18358909, 17904278, 18398147, 18256325, 18413707, 18794390, 18069117, 18256066, 18177757, 18519824, 18323548, 18378519, 18669816, 18572079, 18344568, 18316673, 18385498, 18339977, 18508828, 18765391, 18174609, 18272504, 18556716, 18535196, 18768436, 18244958, 18554712, 18514729, 18783988, 18096708, 18172039, 18191322, 18486724, 18328909, 18039954, 18421055, 18305085, 18198394, 18596271, 18305265, 18242412, 18270352, 18332267, 19013107, 18182621, 18625626, 18922041, 18997197, 18602687, 18420499, 18819960, 18375417, 18635428, 18574086, 18316791, 18440315, 18762599, 18184631, 18711172, 19574441, 19892392, 19878986, 19729344, 19027013, 19738118, 19625715, 19433702, 19920268, 19213793, 19652123, 19776408, 19135997, 19473751, 19531579, 19797280, 19846844, 19561037, 19181826, 19179549, 19129205, 19585653, 19524353, 19240066, 19460767, 19636533, 19451183, 18647851, 19406340, 18775942, 18633127, 19541717, 19245970, 19652108, 19255389, 19109560, 19281920, 18952082, 19188646, 19136368, 19282098, 19470696, 19567451, 19114685, 19884613, 19474053, 19520905, 19786682, 19433697, 19755574, 19812401, 18835953, 19884591, 19329822, 19502645, 19657122, 19657121, 19717646, 19213683, 19136369, 19958962, 19188136, 19189907, 19174452, 19652099, 19748665, 18853144, 19528367, 19880844, 19952143, 19264239, 19332467, 19620483, 19124807, 19293413, 19738134, 19704066, 19264972, 18413439, 19324944, 18701556, 18684743, 19201775, 18801760, 19064978, 19665644, 19481079, 18285398, 19884222, 19906561, 20004966, 19439741, 19303137, 19433686, 19273708, 19223344, 19298914, 19920051, 19637289, 19047294, 19903804, 19636035, 19368965, 19597054, 18945924, 19364996, 19398690, 19752319, 19933930, 19346521, 19181730, 19776406, 19608997, 19864673, 19139383, 19129278, 19736332, 19502623, 19439743, 19380621, 19892237, 19657123, 19554082, 19948642, 19410576, 19752338, 19602503, 19641202, 19237406, 19427269, 19060001, 19293071, 19329003, 19147693, 19124815, 19858398, 19349313, 19196819, 19351691, 19615730, 19130931, 19202157, 19926008, 19251798, 19761936, 19389560, 19667308, 19717851, 19179550, 19497441, 19824078, 19917838, 19720927, 19304990, 19126811, 18853138, 19232896, 19717185, 19915220, 19805650, 19179203, 19176440, 19474112, 19386610, 19679693, 19809023, 19196674, 19293069, 19303012, 19126633, 19279301, 19786657, 19339358, 19920236, 19153121, 19422079, 19414635, 19366774, 19555836, 19188504, 19204305, 19723701, 19047297, 19282246, 19755362, 19933961, 18978177, 18854973, 19812130, 19339720, 19410716, 19549707, 19368963, 19487635, 19587369, 19717847, 19787809, 19380477, 19196673, 19846212, 19318651, 20008376, 19124698, 19616720, 19846851, 18485575, 18931097, 18930988, 19926006, 19147636, 19996038, 19124524, 19748656, 19597023, 19364980, 19786683, 19617576, 19805702, 19228872, 19351943, 19884606, 19454639, 19369319, 19470928, 19766640, 19318652, 19242495, 19501932, 19651921, 19900993, 19509031, 19884620, 20008701, 19776407, 19366978, 19147478, 19389820, 19529912, 18977552, 19366755, 19106380, 19264678, 19861667, 19221377, 19884541, 19761935, 18945928, 19884468, 19429632, 19724045, 19896184, 19196894, 19687490, 19246357, 19065672, 19129293, 19367397, 18838485, 19168868, 19487706, 19901118, 19273705, 18375537, 19058760, 19329820, 19304578, 19778669, 19332722, 19208715, 19204212, 19171726, 19826133, 19346228, 19366977, 19233181, 19717844, 19966341, 18931330, 19195772, 19826132, 19118302, 19812399, 19671655, 19671656, 19822837, 19597081, 18524792, 19520908, 18795253, 19188334, 19692689, 19111551, 19208352, 19364976, 19075284, 19410693, 19720208, 19948980, 18406045, 19605829, 19294365, 19364966, 19103988, 19124693, 19506112, 18824461, 19150955, 19297314, 19204201, 19029416, 19147639, 19767092, 19349548, 18662933, 19336625, 19516032, 19349559, 19567452, 19065670, 19015209, 19647862, 19446324, 19164458, 19064982, 19151786, 19171852, 19531784, 18823693, 19458363, 19332455, 19255381, 19773329, 18842612, 19473749, 18783871, 19150702, 19815583, 19279300, 19417194, 19398687, 19959590, 19755572, 19398689, 19818690, 19932356, 19376452, 19451554, 19506137, 19336502, 19581564, 19458364, 19244090, 19213680, 19884596, 19231632, 19884550, 19687339, 19433701, 19465233, 19841453, 19211470, 19318702, 19129280, 19815268, 19398471, 19747918, 19826023, 19289615, 19920000, 19564474, 19435954, 19958964, 19608026, 19570573, 19744615, 19232907, 19700470, 19712802, 19196814, 19724043, 20082927, 19201654, 19147637, 19729522, 19416992, 19155454, 19738090, 19171717, 19837254, 19477351, 19103729, 19584345, 19127975, 19066370, 18486308, 19643310, 19083199, 19875426, 20009055, 19487605, 19451556, 19153265, 19515415, 19647607, 19451347, 19684100, 19208703, 19732949, 19589822, 19640941, 19349629, 19443914, 19901116, 19917849, 19602539, 19208714, 19364999, 19181729, 19923205, 19622553, 19477503, 19231633, 19255395, 19542011, 19721018, 19690306, 19723707, 19741188, 19339045, 19920270, 18819961, 19528561, 19211468, 19164356, 19690345, 19289636, 19738091, 19218306, 19808924, 19171808, 19592624, 19351942, 19188677, 19936044, 19874992, 18957532, 19922995, 19436015, 18495735, 19755697, 19809026, 19853906, 19700774, 19409693, 19234106, 19406871, 19246588, 19269895, 19265025, 19717850, 19549737, 19131469, 19460913, 19770382, 20008695, 19770395, 19204301, 19561338, 19570717, 19723695, 19762075, 18931095, 19046761, 19819004, 19246088, 19428297, 19015206, 19015207, 19351941, 19628124, 19095497, 19289857, 19652065, 19644045, 18250112, 19528368, 19581560, 19643320, 19230772, 19118724, 19129277, 19153116, 19767078, 19586656, 19505424, 19451428, 19386683, 19074913, 19389561, 19273704, 19996048, 19859531, 19070889, 19136486, 18794178, 19188505, 19564557, 19380451, 19213681, 19091394, 19213787, 19605828, 19717345, 19255394, 19797436, 19733905, 19439742, 19201650, 19704068, 19155456, 19620495, 19940281, 19366957, 19443659, 19573904, 19858385, 19671919, 19270260, 19224846, 19349325, 19446909, 19075102, 19291787, 19204218, 19767074, 19713451, 19196813, 19900934, 19263034, 19915217, 19398668, 19372000, 19652172, 19675206, 19577798, 19920054, 19824074, 19786634, 19605849, 19389558, 19010983, 19818685, 19884557, 19114683, 19428097, 19726772, 19465426, 19276452, 19237716, 19858174, 19185975, 19144937, 19752173, 19581556, 19556260, 19778672, 19692680, 19429918, 19118304, 20040554, 19276396, 19092145, 19948978, 19560810, 19066176, 19150703, 19590022, 19822836, 19072827, 19451440, 19273701, 19901194, 19299497, 19255328, 19303160, 19270316, 19433757, 19349317, 19767089, 19776405, 19201016, 18955565, 19585548, 19996400, 19850219, 19362361, 19423623, 19786675, 19696440, 19470837, 19339719, 19540162, 18992248, 19273725, 19380868, 19841299, 19451435, 19189904, 19724041, 19875843, 19786664, 19364997, 19171813, 19131458, 19679248, 19676125, 19001475, 19917887, 19736358, 19720914, 19103995, 19307511, 19139435, 19917842, 19875425, 19670459, 19556519, 19429874, 19738137, 19414681, 19451431, 19106196, 19135998, 19075279, 19130936, 18713759, 19805701, 19366752, 19070356, 19224928, 18839137, 19815581, 19346328, 19329498, 19351624, 19805698, 19767384, 19444870, 19720913, 19348923, 19575366, 19656558, 19188654, 18930987, 19155081, 19109962, 19744614, 19246544, 19345145, 18718986, 19414665, 19726771, 19487394, 19366972, 19336639, 19592632, 19592630, 19483114, 19528337, 19846848, 19729520, 18946661, 19364964, 19176240, 18661120, 19318384, 19358940, 19828532, 19249153, 19620163, 19773226, 19208649, 19454638, 19276198, 19097942, 19934423, 19221370, 19524577, 19915222, 19020859, 19529915, 18353529, 19375849, 19771409, 19371824, 19884549, 19028780, 19487623, 19646443, 19654097, 19724042, 19560259, 19805679, 19786658, 19304577, 19636019, 19692688, 19011153, 19690341, 19349322, 18678766, 19515413, 18819705, 18678578, 19406886, 19573727, 19909879, 19139391, 19273782, 19103732, 19033260, 19651711, 18669576, 19748319, 19433655, 19244174, 19901139, 19307526, 19508995, 19720939, 18824499, 19874989, 19171716, 19770374, 19717347, 19019888, 19386609, 19690310, 19539144, 19118301, 19767093, 19255334, 19289454, 19321868, 19955524, 19581544, 19539142, 19704100, 19470936, 19217992, 19638505, 19054552, 19520613, 19264686, 19264687, 19852040, 19369318, 19880616, 19901104, 19273709, 19797281, 19597072, 19349314, 19729663, 19297565, 19336459, 19345831, 18388160, 19700435, 19770386, 19596010, 19805692, 19752340, 19770393, 19581567, 19218249, 19726226, 19014944, 19477358, 19047289, 19850207, 19801201, 19141765, 19064965, 19822825, 19506172, 19559476, 19690081, 19699738, 19901110, 19547952, 19682735, 18853141, 19211442, 19409854, 19660462, 19761938, 19796802, 19664895, 19414708, 20117364, 19237660, 19767090, 19910033, 19843899, 18832520, 18957534, 19884558, 19270315, 19196887, 19890125, 19778673, 19470987, 19638648, 19690349, 19164186, 19204202, 19258557, 19414694, 19075271, 19053049, 19139320, 19717184, 19729418, 19826022, 19460609, 19487381, 19164205, 19052139, 19422982, 19487593, 19420364, 19135415, 19778663, 19237720, 19901144, 19139382, 19549706, 19136484, 19346225, 19228863, 19625712, 19687336, 19683639, 19228612, 19996037, 19435740, 19176454, 19710500, 19369231, 19139427, 19917850, 19380449, 19556318, 19652072, 19380445, 19139433, 19826135, 19636011, 19720908, 19139439, 19380443, 19433689, 19720922, 19858379, 19652068, 19075260, 19433683, 19289630, 19273714, 19075278, 19720897, 19581539, 19332727, 19307500, 19805683, 19188680, 19164456, 19254942, 19720909, 19349543, 19704064, 19687335, 19581537, 19196886, 19064988, 20082930, 19401543, 19364975, 19933962, 19139323, 19282347, 18813910, 19132338, 19349323, 19414675, 19704057, 19892235, 19588122, 19781847, 19232899, 19915221, 19435959, 19363221, 19443528, 19652059, 19752406, 19447518, 19581636, 19564531, 19255380, 19592625, 19211454, 19564902, 19249633, 19661244, 19720911, 19371823, 19237657, 19223438, 19164187, 19770376, 19269519, 19571281, 19487235, 19059639, 19620490, 19487639, 19281922, 19383732, 19491183, 19118720, 19129289, 19595348, 19135235, 19797133, 19147609, 19349544, 19858400, 19221039, 19213790, 19332468, 18957487, 19349306, 19581545, 20004781, 19903918, 19362676, 19047288, 19752363, 19386684, 19687359, 19244158, 19091821, 19433681, 19273715, 19273702, 19380444, 19641203, 18653484, 19765815, 19805651, 19362552, 18987031, 19736359, 19359034, 19351690, 19451574, 19687165, 19590019, 19228620, 19546404, 19828373, 19282468, 19286422, 19805678, 19858392, 19636001, 19755551, 18848382, 19474425, 18593759, 19465422, 19114687, 19349549, 19632716, 19136303, 18810388, 19805710, 19324253, 19171853, 19539141, 19502544, 19996018, 19738111, 19610055, 18948425, 19833787, 19752409, 19064985, 19687337, 19561024, 19597031, 19923168, 19770378, 19457937, 19307503, 19687332, 19289623, 19858396, 19047285, 19273696, 18783876, 18783874, 19506159, 19064973, 19237632, 19704058, 19204207, 19171708, 19786668, 19139440, 19451425, 19884554, 19103990, 18718892, 19556262, 19085908, 19075276, 19590021, 19318632, 19632710, 19289640, 19541792, 19411369, 19651710, 19720642, 19738125, 19687128, 19366965, 19752378, 19939445, 19587338, 19650216, 20050018, 19281927, 19155060, 19567910, 19528562, 19124701, 19528195, 19329177, 20082923, 19349560, 19587364, 19468011, 19651918, 19171735, 19720635, 19047319, 19497447, 19493864, 19329178, 19282342, 18949456, 19571282, 19587362, 19298916, 19586960, 19202155, 19414836, 19523672, 19349552, 19736331, 19364963, 19291790, 19380853, 19907042, 19667239, 19029129, 19483252, 19786678, 19329361, 19805690, 19282274, 18388156, 19567453, 19940299, 19411100, 19539361, 19716961, 19716960, 19796737, 19501900, 19332456, 19553646, 19130937, 19042161, 19647866, 19546387, 19042080, 19167925, 19286091, 19493565, 20016688, 19297566, 19487613, 19654384, 19553269, 19501587, 19223439, 19243886, 19244175, 19282241, 19174453, 19447249, 19179556, 19721013, 19933935, 18990678, 19164351, 19657112, 19745163, 19542476, 19509011, 19487710, 19570931, 19153001, 19255393, 19470941, 19171723, 19324974, 19208350, 19560257, 19164343, 19282833, 19651919, 18795255, 19933933, 19297384, 18807008, 19114612, 19688036, 19713235, 19451442, 19451573, 18996379, 19695955, 19664956, 19386548, 19641522, 19414832, 19255379, 19581631, 19505878, 19846849, 19850525, 19168537, 19406981, 19556517, 19843565, 19465470, 19704072, 19782873, 19797474, 19071061, 19675193, 19542207, 19962185, 19303414, 19249634, 19729114, 19470885, 19828704, 19403903, 19403902, 18799512, 19918088, 18955563, 19074911, 18818699, 19336462, 19477353, 19920001, 19285788, 19570929, 19353740, 19264245, 19138567, 19786674, 19494215, 19506174, 19707579, 19707575, 19429875, 19468030, 19336497, 19858437, 19349551, 19168870, 19652948, 19205663, 19679245, 19478198, 19884223, 19324916, 18807007, 19013011, 19233855, 19800193, 19858439, 19297248, 19837741, 19570933, 19162029, 19833260, 19723787, 19124705, 19483109, 19700005, 18987654, 20004779, 19850703, 19398575, 19161878, 19717846, 19414713, 18783877, 19581546, 19324954, 19443532, 19070418, 19318634, 20082922, 19864035, 19074978, 19596659, 19289619, 19917839, 19786670, 19141764, 19553647, 19349308, 19845037, 19564636, 19470838, 18502028, 19366970, 19745215, 19858438, 19349630, 19046971, 19584344, 19508994, 18854549, 18294761, 19920269, 19822770, 19494217, 19782872, 19221371, 19448187, 19620162, 19217154, 19683638, 19843557, 19297572, 19369667, 19549996, 19332730, 19096005, 19687338, 19608718, 19139319, 19477893, 19770373, 19115219, 19273781, 19179490, 19451353, 19116389, 19066368, 19858394, 19395079, 19858413, 19217991, 19017773, 18625625, 19411662, 19675330, 19179316, 19445938, 19720808, 19581542, 19901122, 19805689, 19362675, 21091061, 20870099, 20417052, 20045278, 20682679, 20200301, 20810376, 20616218, 20484178, 20978060, 19622592, 20231324, 20152561, 20413516, 20538165, 20660848, 20150293, 20627974, 21135328, 20498212, 20479282, 20231298, 20697044, 19940012, 20430261, 20089960, 20089952, 20194829, 20206437, 20800340, 20116842, 20093351, 19608589, 20551461, 19643937, 20511606, 20679587, 20152559, 20837847, 20484124, 21041592, 20351239, 19605504, 19880501, 20159247, 19628570, 19622596, 20393176, 20860505, 20101008, 20860506, 20008945, 20818876, 20660401, 20462632, 20008941, 20581422, 20056900, 19833819, 20142523, 20513595, 20581420, 20802250, 20410514, 20637202, 20200385, 20920764, 20956208, 20009091, 20406930, 20038728, 20385929, 20472590, 20089951, 20430262, 20888994, 19962227, 20410466, 20368559, 20138037, 20823433, 21121833, 20837948, 20937992, 19581281, 20592293, 20864251, 19633051, 20807874, 19628566, 20479407, 20159820, 20600013, 20600017, 20601389, 19965667, 20810472, 20841532, 20621700, 20625026, 20032275, 20811491, 19481335, 19633042, 19889649, 20879879, 20639252, 20703256, 20100958, 20237130, 20579537, 20460620, 21179434, 19875685, 20947884, 20805256, 21135325, 20879881, 19828470, 20137805, 20554983, 20942666, 20942667, 20091678, 20938912, 21175312, 20206776, 20810159, 20525844, 20511610, 20705755, 21062666, 20197530, 20828648, 21050973, 21135365, 20335572, 20679159, 19896902, 20850381, 20624648, 20008943, 21064034, 20085939, 20833658, 20622033, 20298928, 20038732, 20498392, 21029874, 20513728, 20026804, 20551460, 20833738, 20231532, 20688574, 19819617, 20525836, 20368567, 20483969, 19825849, 20212252, 20855823, 19850944, 19910613, 20538634, 20564380, 20044997, 20697028, 20150324, 20516378, 20472597, 20236700, 19844694, 20237760, 20823406, 20368561, 21146205, 20739218, 20940200, 20498215, 21051656, 20048226, 20298924, 20600032, 20682320, 20940383, 20308641, 20189881, 20308671, 19713248, 20516443, 19933908, 21087711, 21073365, 20951323, 20620720, 20189239, 20109864, 20693270, 20033055, 20638563, 20100966, 21071270, 20067974, 21115882, 19875337, 20505152, 20691689, 20054046, 21156747, 21148220, 20833591, 20375404, 19446951, 20458051, 19901115, 20493653, 20670761, 20085937, 20525840, 20537753, 20385930, 20610442, 20513828, 20335070, 20363764, 20925534, 19846796, 20883926, 20736471, 20576697, 20376430, 20805112, 21190455, 20097780, 20947887, 20008946, 20622031, 20202971, 20083678, 19949016, 19570764, 21123342, 20530082, 20877714, 20460331, 20159292, 19756505, 20691553, 20103757, 20212251, 20522795, 20200382, 20232047, 20138809, 20505177, 20566487, 20079528, 19297346, 20923715, 20124563, 20194850, 20631341, 20595145, 20071701, 20554978, 20883925, 20436049, 20061012, 20818864, 20427350, 20032123, 20138810, 20498402, 20643988, 19949136, 20439295, 20521025, 20413036, 21106625, 19903683, 20591841, 20713865, 20578138, 20157135, 20688811, 20103758, 20406865, 20139218, 19740900, 20679589, 20368491, 20038515, 19933991, 20818863, 20133925, 21098435, 20048271, 20404381, 20427397, 19901117, 21147728, 21059484, 20163990, 20153888, 20439832, 20861007, 20566676, 20525995, 19155234, 19918017, 20132969, 20921122, 20729270, 20483968, 21060033, 19897551, 20019087, 20130241, 20956707, 19808926, 20079596, 20817301, 20581167, 21067382, 20194883, 21105792, 20413026, 21135341, 20625068, 20864406, 20075385, 19949019, 19917596, 19819914, 21045096, 20138043, 20858857, 20418013, 20975066, 20164484, 20841613, 20357371, 20511615, 20805115, 20080830, 20817281, 21081728, 20436046, 21080780, 20231231, 20215461, 20493191, 20533022, 20472939, 20177025, 20480136, 20463176, 20206179, 20683945, 20818875, 20150302, 20060783, 20573925, 20888993, 20647201, 20407057, 20818887, 21173411, 20160168, 20019086, 21126973, 20664019, 20123835, 20935338, 21156950, 20207411, 20921123, 20457737, 20682933, 20643989, 20716738, 21059972, 21087898, 20819983, 20442386, 20547990, 20424250, 20733097, 20825986, 20609968, 19363022, 20959577, 20357281, 20510211, 20110554, 20185740, 20606148, 20959284, 20458090, 20542554, 20961244, 20107214, 20215608, 21118874, 20103550, 20594588, 20668152, 20048204, 21131038, 20673995, 20228402, 20816546, 20863761, 20067964, 20159249, 20880898, 20400483, 20357382, 20841534, 20231251, 20009090, 21056464, 20009095, 20194881, 20207412, 20179126, 20818901, 20628129, 20332353, 20150300, 20228403, 20435345, 21083385, 20362507, 20501583, 21081600, 19815809, 20664017, 20124143, 21144971, 20522656, 21159375, 20927359, 20923841, 20068255, 20124230, 20832366, 20139214, 20049907, 20688209, 20357377, 20587585, 20685806, 20682932, 20065126, 20501479, 21071074, 20541828, 20587725, 21098771, 20065132, 20621562, 21115589, 20227347, 19969339, 20335369, 20228404, 20566677, 20979470, 19854713, 19734132, 20935337, 21149757, 20332357, 20107105, 20101011, 20060830, 20585005, 20571015, 20228401, 20451523, 19846798, 20639560, 20587587, 19962744, 20394870, 19921150, 20236788, 21070920, 20101007, 20554319, 20863951, 19880586, 20573755, 20417857, 20728932, 20932805, 20625110, 20801496, 20488885, 20580422, 19747792, 20067958, 20691641, 20360317, 20378080, 20807892, 19357113, 20008644, 19918980, 21078810, 20223367, 20048210, 20231034, 20211953, 19965813, 20124144, 20705516, 20692693, 20573636, 19879276, 19959398, 20133929, 20044806, 20089449, 20045700, 20048184, 20231319, 21072246, 20692031, 20692030, 20519663, 20530557, 19963436, 20444845, 21071584, 20348368, 20736470, 19855955, 20947881, 20625111, 21062875, 20551457, 20600033, 20026785, 20382982, 20382983, 20439791, 20439792, 20937922, 20386003, 20921442, 20049753, 20801542, 20427682, 20089562, 20679619, 20368566, 20670756, 20547163, 20332140, 21109113, 20231536, 20493771, 20843249, 20124118, 20150289, 19717399, 20439627, 20959578, 20200383, 21098467, 20368520, 20215457, 20308616, 20581392, 20445180, 19703922, 20424219, 20101013, 20385794, 20547904, 20621900, 20933466, 20525999, 20142594, 20211180, 20142572, 20067954, 21045097, 19837071, 20004661, 20395260, 19940014, 20497977, 20147743, 20178792, 20660806, 20177030, 21131037, 20382075, 20837925, 20203244, 20860504, 19635718, 20573923, 20537493, 20139154, 20231684, 20357372, 20232036, 20495781, 20573926, 20022809, 20606091, 20103782, 20970382, 20413602, 20538738, 19933225, 20825316, 20177043, 20805258, 19846793, 20376431, 20444749, 20406937, 20165941, 20605316, 20189241, 20368481, 20655105, 20937978, 20185731, 20118174, 20801495, 19875684, 20884297, 20723545, 20038724, 19940005, 20075055, 20970380, 20683947, 20124113, 20800922, 20530316, 20884893, 20427683, 20299485, 20007944, 20818888, 20196118, 20581169, 21175313, 20007927, 20018365, 20736456, 20026597, 20980048, 20679165, 20067963, 20466791, 20693353, 20547926, 20150206, 20439798, 20466200, 20139221, 19880433, 20202514, 20362386, 20303646, 20585099, 19933744, 20667790, 20592248, 20407035, 19587388, 20525992, 20659956, 20620742, 20200309, 20357376, 20660805, 20478960, 20673983, 20689926, 19797259, 19850637, 20620737, 20805433, 20846599, 20185036, 20843575, 20679551, 20816836, 20413522, 20393175, 20022872, 20089518, 20386004, 20728265, 20655106, 20832963, 20724648, 19684105, 20592050, 20584776, 20921542, 21106618, 21139110, 20458045, 20713848, 20818902, 20395554, 21067805, 20338496, 20153039, 20522796, 20679548, 20447955, 20124127, 21098442, 19778914, 20679166, 20124231, 20610444, 20004617, 20708966, 19574331, 19717476, 20805113, 20801500, 20846991, 19640853, 20028938, 20179283, 20621711, 20145228, 20194853, 20937977, 20181970, 20117443, 20413514, 20876454, 19853510, 20231232, 20538001, 20427390, 20548010, 21126970, 20801122, 20439345, 20417856, 20092882, 19793768, 20488910, 20124114, 20519679, 20530280, 20934206, 21190965, 20733096, 20837894, 20543994, 20109743, 20951320, 20299535, 20007932, 19643946, 21079217, 20048236, 20237125, 19877169, 19717481, 20034729, 19717534, 20142410, 20697092, 20688033, 20117965, 20385996, 20484396, 20548096, 20684981, 20876406, 20679528, 19651631, 20650358, 20147717, 19933916, 20409606, 20089558, 20619475, 20605202, 20709992, 20442215, 20513659, 19643944, 20170821, 20357033, 20413028, 20724402, 21144969, 20110289, 19841042, 20554982, 20463338, 20497967, 20864405, 21135366, 21098770, 20513826, 19965659, 20448107, 20729148, 20810155, 20921440, 20124139, 20371785, 20542736, 21135293, 20451454, 20227758, 20494730, 20504899, 20056901, 20705223, 20038723, 20032498, 20154213, 20434199, 19608584, 20434204, 20685676, 20598634, 20823391, 20159816, 20647200, 21060031, 19917571, 20625015, 21060104, 20538329, 21098443, 19625343, 20929341, 20818886, 20625011, 20434400, 21060024, 20818904, 19901111, 20113825, 20308670, 21142534, 20843245, 20439824, 20189458, 20164485, 19717477, 20525993, 20876405, 20357369, 20083683, 20305034, 20805274, 20194812, 21118875, 20124229, 20101009, 20080983, 20601414, 20332355, 20170790, 20418251, 20557927, 20647285, 20421448, 20339140, 20008947, 21060071, 20609969, 20463178, 20064514, 20430429, 20400762, 20299481, 20610322, 20124183, 20606147, 20124218, 20951424, 20089954, 20185734, 20220184, 20542324, 20451040, 20817247, 19717479, 20457157, 20798393, 20564351, 20107216, 21144968, 20530001, 21126639, 20924011, 20100959, 20150572, 20606094, 20519680, 19687327, 20194353, 20494434, 20660803, 19293160, 20679600, 20558431, 20689928, 19875752, 20100962, 20007996, 19852964, 19909752, 20308602, 20498395, 20102716, 20689921, 20933321, 20637539, 20119647, 20522709, 20937921, 20385995, 20530282, 20512995, 20631375, 20660830, 20921467, 20351325, 20606083, 20065174, 20142595, 20733134, 20479419, 20212249, 20837956, 20606093, 20625120, 20498403, 20212250, 20124186, 20516439, 20385998, 20530281, 21079147, 20567019, 20351334, 20368558, 20368553, 20733132, 20498389, 19901102, 20625123, 20008622, 20508212, 20150294, 20427778, 19996196, 21060036, 20651284, 20068208, 20587704, 20823436, 20937929, 19909743, 20494902, 20587720, 20606118, 20452823, 20609970, 19766643, 20444847, 20212256, 20830787, 20142447, 20525842, 20159245, 19854729, 20888992, 21091279, 20876412, 20693354, 21177506, 20837928, 20071702, 20516435, 20227168, 19815652, 19926748, 20054045, 20065185, 20951321, 20357374, 19741029, 20308618, 21098426, 20851460, 20522788, 20502866, 20516155, 20185423, 19828561, 20409752, 20231682, 20439630, 20008640, 20697079, 20034973, 20101752, 20644020, 20081092, 20200306, 20802205, 20805454, 20447535, 19853987, 19910499, 20580483, 19541675, 20801120, 20048227, 20179125, 19875683, 20558628, 20133204, 20625131, 20638899, 20837834, 19679600, 20335368, 20444751, 19897418, 20956620, 20234040, 20378636, 20921118, 20332352, 20625129, 20670755, 20118171, 20117456, 20601395, 20816187, 20708331, 21038410, 19827166, 20733135, 20547984, 20038734, 19815651, 20837952, 20921462, 19906761, 20194854, 20530276, 20038730, 19933915, 19841330, 19808912, 20048183, 20124187, 20434505, 20124169, 20226618, 20683847, 20567002, 20837914, 20634482, 20194880, 20530307, 20805116, 20056957, 20100963, 21087706, 19917841, 20826719, 20713879, 20921465, 19852962, 19926749, 20448287, 20207689, 20423964, 20845544, 20845530, 20848748, 20007948, 20920772, 20338501, 20832848, 20818855, 20497980, 20048237, 20421547, 20236696, 20479421, 20413397, 19825809, 20610445, 20520636, 20644019, 20606124, 20150290, 20189026, 20837926, 20170948, 21093036, 19846407, 20554627, 20471156, 20501928, 20101012, 20133927, 19952006, 20194878, 20157180, 20008620, 20855825, 20103547, 20479459, 20823434, 20303352, 20335583, 20709233, 21147743, 20231321, 20439641, 20023215, 20194844, 20647199, 20647198, 20400552, 20038733, 20925544, 21067381, 19808911, 20022873, 20404379, 20026779, 20202516, 20004464, 20497961, 20595412, 20007923, 20363190, 21109302, 20980427, 20723799, 19913323, 20018364, 20018367, 20508026, 20097702, 20837852, 21082868, 20439572, 20638901, 20660832, 19643947, 20921349, 19671542, 20940193, 20060578, 20888162, 21055801, 20150299, 20858878, 20863759, 20855664, 20568303, 20675691, 20117403, 20538327, 20925543, 20484128, 20019345, 20190331, 20668151, 20460548, 19850639, 19304567, 20147716, 20855800, 20206581, 20818862, 20581391, 20028939, 20921347, 20142600, 20422150, 19734131, 20237316, 20385988, 20920765, 20339154, 20147715, 20505173, 20197425, 20439790, 20713790, 20624650, 20670760, 21134997, 20153030, 20215126, 20802206, 20332388, 20628153, 20124509, 20511018, 20980426, 20231678, 20457158, 20445161, 20409751, 19451137, 20074791, 20332351, 19460761, 20186843, 21149761, 20942668, 20472937, 20570343, 20542963, 20375406, 20484125, 20619448, 21080835, 20603462, 21041576, 20855843, 20600025, 20709444, 20733102, 20950166, 21098355, 20888520, 19155235, 20413513, 20828645, 20399059, 20061358, 19887674, 20332354, 20884700, 20226302, 20061360, 19825505, 20573750, 20439575, 20231565, 19841897, 21135294, 20920766, 20332506, 19828469, 19965647, 20304805, 20650926, 21041593, 19364728, 20167847, 19756506, 20516379, 20683930, 20044810, 19508966, 20332358, 20551163, 19965682, 21098449, 20223838, 20137821, 20855784, 20494961, 20805430, 21060072, 20802246, 21135327, 20457948, 21109115, 20185426, 20979471, 20479425, 19942604, 20697090, 20048189, 20516432, 20961243, 20940381, 19913351, 20728210, 20595411, 20117379, 20051465, 20801085, 20060161, 20060162, 20478877, 20643762, 20089970, 20427348, 20185037, 19800680, 20837917, 19917869, 19942479, 20699432, 20004013, 20876410, 20816189, 20346534, 20501476, 20097417, 20220185, 19949010, 19965693, 21115766, 20206777, 20633818, 20570559, 20713903, 20701962, 20018913, 20679620, 20721883, 20215360, 20625021, 20580423, 20375405, 20620713, 20679559, 21148134, 21060068, 20956710, 20650362, 20139217, 20194251, 20716770, 21987195, 21777974, 22027547, 21514083, 21620467, 21492926, 21852252, 22029980, 22047557, 21355098, 21300730, 21727288, 21447661, 21960587, 21482191, 21916638, 21440548, 21266516, 22087077, 21377715, 21536689, 21798429, 21173013, 21765808, 21248161, 21483712, 21285389, 21641635, 21402616, 21252171, 21636861, 22204725, 21212157, 21372124, 21321086, 21226577, 21540430, 21296481, 21520194, 21186077, 21470815, 21165734, 20562124, 21804100, 21358807, 21317176, 21232669, 21329837, 20870757, 21190996, 21991280, 21233311, 21377034, 21390262, 21900148, 21145828, 21464382, 20870753, 21646584, 21753188, 20696676, 21330337, 20732932, 21059637, 20837824, 21788663, 21828126, 22025101, 21768610, 21810636, 21220660, 21514637, 21306277, 21502541, 21392640, 21825259, 21762974, 21791453, 21200038, 21879897, 22082239, 21419704, 22077909, 20944071, 21612468, 21742734, 22180732, 21774708, 21098740, 21751905, 21256670, 21209123, 21406514, 21641636, 22065255, 21813427, 21502546, 21641868, 21247627, 21991949, 21041710, 21875911, 21199980, 21623000, 21385856, 21591943, 21397567, 21236483, 21189395, 21788567, 21482992, 21550483, 21135055, 21257787, 21237167, 21835135, 20833737, 21321298, 21220656, 21561348, 21714999, 22047559, 21665264, 21488067, 21354234, 21309657, 22077144, 21870978, 21780946, 21393063, 22090661, 21282540, 21784729, 21623001, 22018631, 21183500, 21135313, 22187278, 21211684, 21969011, 21145803, 21813548, 21320923, 21836140, 21106985, 21777972, 21951683, 21406717, 21415269, 21282546, 21146458, 21963186, 21304082, 21306953, 21864166, 21333599, 21699484, 21816315, 21478189, 21224456, 21593294, 21715517, 21844504, 21115602, 21357794, 21920260, 21670455, 21875860, 22090660, 21622587, 21059830, 21159892, 21449783, 21612471, 21849487, 21724462, 20805294, 20603439, 21995387, 21199966, 21907863, 21070781, 21676993, 21474646, 21403011, 21709199, 21709064, 21458058, 21540198, 22110104, 21383294, 22025143, 21831418, 21844495, 21606084, 21982313, 22088800, 21357827, 21492764, 22074713, 21415050, 21300933, 21502544, 21785107, 21804104, 21321292, 21498674, 21570111, 21550310, 21367761, 21940214, 22123795, 21810649, 21467544, 21775756, 22000135, 21489882, 21640651, 21807405, 21862730, 21139020, 22078420, 21914628, 21474517, 21788632, 21239701, 21185507, 21376050, 21934055, 21572163, 21727253, 21676990, 21873705, 22090167, 21911632, 21646579, 21398178, 21606094, 21282130, 21685374, 22056152, 21768544, 21536692, 21753226, 21768582, 21212381, 21911629, 22078721, 21199975, 21546309, 21813428, 21041707, 20603436, 22056247, 21844080, 21403042, 21986350, 22085317, 21893650, 21111555, 21334061, 21697170, 21571593, 21149658, 21690555, 21402611, 21536937, 22053315, 21255828, 20833822, 21963002, 22056739, 21921285, 20959555, 21719756, 21836137, 21527567, 21300709, 21463150, 21211651, 21112898, 21931077, 21949643, 21719096, 21931037, 21464378, 21805159, 22168642, 21788542, 21277016, 21652750, 21242364, 21652683, 21410371, 21606413, 21481708, 21200007, 21551462, 22111718, 21816980, 21434843, 21149651, 21884956, 21531741, 21357809, 21976185, 21343383, 21353695, 21169471, 22180403, 21211644, 21047696, 21211688, 21636122, 21824940, 21670398, 21705068, 21515838, 21282282, 21515916, 21633086, 21068104, 21242235, 21601352, 21464362, 21366472, 21975270, 21803749, 21747085, 21972410, 21196073, 21149662, 21856480, 22088980, 21354977, 22082198, 21872918, 21030144, 21679712, 22042952, 22010913, 21484081, 21406458, 21388992, 21723220, 21403073, 20847081, 21714640, 21879896, 21145807, 21939829, 22084333, 21862744, 21565921, 21526229, 21696814, 20833820, 20929988, 21571733, 21216860, 21750293, 21612469, 21199957, 21914755, 21216000, 21680723, 21335621, 22089242, 21610123, 21768541, 21406510, 21741309, 21093145, 21705063, 21636633, 21621834, 21947297, 21954478, 21346183, 20805297, 22084195, 21851883, 21537042, 21885537, 21998346, 21098340, 21732835, 22065184, 21245085, 21343579, 21636796, 21247310, 21493672, 22078686, 21282693, 21642681, 21176950, 21596735, 21576533, 22131908, 21636031, 21145284, 21317148, 21771987, 21804130, 21292711, 21724564, 22085316, 22108262, 21521847, 21574174, 21628364, 21515164, 20975012, 21646588, 21680622, 21896611, 21810633, 21487057, 21357819, 21220657, 21474675, 21791454, 21836103, 21536693, 21873560, 21986283, 21190995, 21646583, 21296237, 21139088, 21378215, 21343580, 22028630, 21836134, 21996389, 21372193, 21868779, 21958949, 21057005, 21481449, 21994424, 21810630, 21215550, 21281870, 21444887, 21727276, 21875858, 21406465, 21788624, 21545947, 22089718, 20843978, 21816979, 21199958, 21586714, 22104550, 21771568, 22115874, 21228335, 21486974, 21255957, 21775755, 21296403, 22082672, 21216197, 21503940, 21109557, 21281874, 21111681, 21636116, 21798658, 21458153, 21889410, 21209062, 21410393, 21871706, 21211643, 21211642, 21846852, 21624649, 21865087, 21068096, 21992121, 21593041, 21621716, 21263078, 22123804, 21420555, 21245182, 21317434, 21323540, 21482925, 21288094, 21262997, 21237715, 21529927, 21784047, 21925126, 21329971, 21536947, 21990399, 21831711, 21149743, 20739054, 21131631, 21419769, 22041948, 21282548, 21439962, 21976613, 21073363, 21376385, 21783417, 21562038, 21144850, 21968126, 21422407, 21270204, 22042959, 21565848, 21986347, 21048039, 21208974, 20716625, 21958884, 21306238, 22119496, 21334736, 22018294, 21885624, 21480316, 21693709, 21752462, 21639806, 21285388, 21996391, 20921216, 21926186, 21277076, 22068990, 21723781, 21699777, 21800340, 21699794, 21288078, 21898496, 22007715, 21169060, 21288774, 21646500, 22010012, 21992852, 21216670, 22030144, 21561347, 21050812, 22180399, 21593296, 21768458, 21482968, 22080794, 21300940, 22074851, 21903052, 21454917, 21531174, 21969502, 21924547, 22084374, 21482928, 21163921, 21809362, 21411505, 21216851, 22070475, 21200004, 21270184, 21334262, 21631324, 21658839, 21459975, 21449786, 21820163, 21467283, 21958882, 21969017, 21875859, 22123802, 21345487, 22147778, 21665265, 20833659, 21403017, 21310311, 21196444, 21439960, 21445641, 21347869, 21215445, 20854819, 21747013, 21482964, 21812672, 21527519, 21333346, 22029979, 21274882, 21427372, 21571149, 21571150, 21125215, 22000683, 21172891, 21266519, 21821621, 21374656, 21651392, 21486975, 21675889, 21168369, 21153402, 21149672, 20924555, 21555684, 21576654, 21439754, 21515841, 21646616, 21406472, 21632496, 21718908, 21747002, 21382896, 21690465, 20980412, 21885625, 21444883, 22123808, 22051332, 21996470, 21270188, 21715522, 21639808, 21737560, 21741344, 21239052, 21300712, 22123798, 20929999, 21949219, 21562320, 21515846, 21803850, 22204724, 21383263, 21616970, 21383276, 21233014, 21969008, 21392641, 21990403, 21383272, 21550314, 21617110, 21289042, 20966170, 21478216, 20937671, 21262998, 21208101, 21788629, 21529928, 21274861, 21270174, 21396703, 21593292, 21747052, 20837822, 22118442, 22077236, 21304925, 21304923, 21078826, 21641867, 21502557, 21546450, 21518939, 21488763, 21878431, 21127322, 21148540, 21545942, 21737787, 21868284, 22012809, 21830966, 21565557, 21931078, 21189381, 21856483, 21558517, 21705493, 21069290, 21639810, 21628399, 21335007, 21256669, 21345850, 21220683, 21658561, 21112655, 21586712, 21719599, 21593096, 21884958, 21812663, 21382893, 22104549, 21576110, 21542741, 21471106, 21626431, 22168590, 21775759, 21824948, 21626525, 21366475, 21632666, 21555700, 21422412, 22075451, 22036019, 21777745, 21366473, 21454798, 21148229, 22025606, 21990405, 21969009, 21444867, 21610124, 21843494, 21398619, 21109517, 21821474, 21403038, 21325445, 21705273, 21893583, 21445642, 21281875, 21334402, 21867844, 21572992, 21270185, 21483003, 21211686, 22204723, 21177297, 21676992, 21856268, 21129375, 21419770, 21199965, 21628381, 21907861, 21911720, 21414843, 21613310, 21525480, 21690592, 21835317, 21878677, 21300930, 21515905, 21958585, 21602430, 21982309, 21747089, 21855209, 21324954, 22065532, 21791491, 21257657, 21831714, 21615299, 21855126, 21429799, 21115860, 21232664, 21422418, 21940742, 21837752, 21969505, 21483000, 22087680, 21606394, 21463153, 22187187, 22110169, 21844082, 20595158, 21474643, 22010917, 22041946, 21719097, 21732833, 22085343, 22123796, 21856226, 21364138, 21458191, 21374657, 21471093, 20978101, 21136039, 21680944, 22150036, 21502572, 21788625, 21163569, 21715526, 21775749, 20622036, 21374690, 22047971, 21859685, 21995388, 21041245, 20970246, 21388309, 21536936, 20693243, 21852264, 21593290, 20837825, 21636798, 21953799, 21991950, 21610239, 21844547, 21900193, 21958886, 21321152, 21852344, 21703676, 21511783, 21402619, 21859995, 21377716, 21807773, 20880999, 21670447, 21670453, 21159889, 21236730, 20595155, 21482966, 21893645, 21422390, 21098343, 21149742, 22157640, 21835957, 21324992, 21775748, 21268724, 21351116, 21724623, 21748765, 22078433, 21463154, 21865084, 21700651, 21444866, 21447587, 21911782, 21505208, 21489609, 21247998, 21048041, 21803011, 21994429, 21498786, 20929990, 21680720, 20855467, 21300929, 21931019, 21931035, 21135270, 21220618, 21149659, 21911726, 21810677, 21196441, 21084429, 21135267, 21282542, 21263100, 21444871, 21098317, 21300931, 20819978, 21428766, 21571362, 21900191, 21991893, 21415069, 21875913, 21561346, 21768546, 21593301, 21193696, 21145839, 21333347, 21402563, 21190790, 21145862, 21606391, 21453832, 21900084, 21149498, 21167235, 21733570, 21683078, 21315654, 21596621, 21385882, 21382868, 22064591, 21816977, 21357805, 21684068, 21268722, 21767103, 21848460, 21272992, 21873708, 22071279, 21906798, 21199978, 21515839, 21732834, 20724576, 21610860, 21982529, 21145834, 21606427, 21704360, 21199963, 21768545, 21839579, 22084369, 21536860, 20716629, 21345849, 21658558, 21537051, 21908768, 21149654, 21310875, 21969500, 21334136, 21454456, 21709061, 21410370, 21300923, 21252061, 21321290, 21487107, 21224458, 21470671, 21542742, 21751904, 21187295, 21933752, 21471566, 21317170, 21454449, 21415104, 21262918, 21593252, 21514084, 21810659, 21173348, 21292644, 21272743, 21400557, 20439262, 21147188, 21263103, 21199979, 21633690, 21383275, 21464376, 21690475, 21555689, 21859991, 20696677, 21606430, 21502556, 22010015, 22025157, 21768463, 21747084, 21189384, 21135284, 21217084, 21709202, 20682548, 21632509, 21860000, 21278220, 21969495, 21228246, 20693429, 21378213, 21081550, 21502571, 21282539, 22042887, 21480321, 21410369, 21991951, 21463149, 21911723, 21502549, 21123833, 21212385, 21343556, 21844506, 21969509, 21576636, 21483004, 21768453, 21990410, 21555686, 21464406, 21464401, 21526923, 22094316, 21145856, 21730307, 21699796, 21703969, 21056535, 21148720, 21297074, 21041606, 21237227, 21714641, 21135266, 20929998, 21816309, 21551507, 21609974, 21307037, 21849660, 21844075, 22051331, 21576655, 21708823, 21852542, 21303799, 21075774, 21916639, 21709297, 21270442, 21193619, 21851876, 22047561, 21900088, 21658562, 21464363, 21383283, 21990397, 20849805, 21208106, 21763936, 21763935, 21551506, 21273347, 21576658, 22084332, 21176949, 21830957, 20851929, 21748770, 20971747, 21878434, 22011408, 21951682, 21908036, 21570355, 21782519, 21486976, 21502577, 21931024, 21798883, 21709060, 21752460, 22031728, 21173357, 21420908, 21591944, 22051739, 21540202, 21933908, 21156335, 21903473, 22042958, 21903745, 21355070, 20813884, 22025430, 21444869, 22045673, 21680726, 21440505, 21109344, 21551508, 21742556, 21383291, 21670457, 21622669, 21131468, 21349913, 21550313, 21722952, 21212155, 21330457, 21824950, 21825268, 21251705, 21406646, 21899409, 21209124, 21300711, 22104547, 21270183, 21220730, 20934771, 21115863, 21507715, 21281959, 21903477, 21306237, 21757112, 21483008, 21398582, 22180731, 21596740, 21173349, 21802126, 21821865, 21518924, 21951630, 21518942, 21926189, 21911633, 21699786, 21696307, 21034744, 21664867, 21254162, 21294143, 21719095, 21616527, 21386088, 21555634, 21525439, 21475252, 21316752, 21873710, 21788540, 21036173, 21255955, 21308360, 21939822, 21266648, 21270182, 21986346, 21835316, 21211691, 20693248, 21285133, 21403043, 21820166, 21996393, 21593295, 21719078, 21551510, 21663949, 21859765, 21856481, 21415052, 21616282, 22051330, 21655317, 21439633, 21545948, 21972411, 21281871, 20693247, 21317177, 21273492, 21875862, 21177840, 21340655, 21684281, 21990966, 21355071, 21737584, 22150035, 20935058, 21211641, 21709065, 21685437, 21847649, 21075773, 22010914, 21636120, 21807250, 21428765, 21659439, 21422420, 21115872, 21570352, 20643863, 20643862, 22147842, 21639811, 20864577, 21263080, 21357811, 20962323, 21226576, 21145848, 21357906, 21263092, 21962214, 21911721, 21768611, 21354370, 21628377, 21256809, 21830967, 21304921, 22144108, 21646329, 21459430, 21300952, 21741700, 22105823, 22018299, 21324520, 21093024, 21138825, 21518946, 21385806, 21285135, 21778294, 22074711, 21145578, 21632504, 21622774, 20650985, 21330640, 21383284, 21282537, 21471085, 21169065, 21285132, 21788633, 21480317, 21270118, 21422438, 21990298, 22084331, 21216855, 20693299, 21821622, 21893621, 21449785, 21270189, 21335417, 21464374, 21315441, 20802165, 22041945, 21377930, 21342915, 22999730, 22828481, 22362859, 22748821, 22958957, 23177293, 22397653, 22796132, 22586006, 21464158, 22193531, 22706327, 22375971, 22084312, 22716974, 22186709, 21965475, 21937705, 22284328, 22695239, 22999717, 22266605, 22418065, 22237175, 23093587, 22357106, 22314425, 22006541, 23136909, 22377563, 22328739, 21464154, 22223819, 22561965, 22157329, 23194941, 22935569, 22357257, 22294639, 22431865, 22210571, 22958958, 22818065, 22764361, 22341737, 22269589, 22269592, 22634125, 22213771, 22507696, 21889833, 23158530, 22689176, 22782331, 22357252, 22280855, 22015451, 22915637, 22127418, 22375972, 22520250, 22494121, 22064874, 22998339, 22435369, 22699288, 22551104, 22464259, 22787177, 22864465, 22128226, 22386286, 22700995, 22905352, 22190678, 23234472, 22998340, 22939385, 22504093, 22995653, 22551128, 22149921, 22507978, 22508814, 22323414, 22398174, 22067384, 21948812, 23109700, 22609619, 22965967, 23247938, 21998121, 22062358, 22326435, 22949147, 23047822, 22851554, 22502942, 22187070, 22493463, 22104610, 22226517, 22105826, 21859900, 22954508, 21750118, 22575589, 22160383, 22965994, 22215753, 22452896, 22965962, 23147172, 22267243, 22732511, 23122650, 23122652, 22361399, 22637938, 22199016, 22337680, 21930686, 22584133, 22316443, 23036134, 22455413, 23134837, 22683257, 22431837, 22554931, 23190222, 22784038, 22784037, 22493422, 23095282, 22251482, 22572202, 22305462, 23060426, 22651863, 21989542, 22621626, 21835953, 22234683, 23062534, 22393088, 22719000, 22497823, 22456864, 22090163, 23008288, 22389634, 21987393, 22986783, 22241898, 23288372, 22915639, 22575317, 22203756, 22261200, 22104603, 22362846, 23113833, 22082673, 22423353, 23169917, 22268137, 22901887, 22527075, 22331983, 22745608, 22449317, 22449319, 22432107, 22549092, 22147112, 22771827, 22124101, 23121439, 22337213, 22210576, 22723578, 22276821, 22565005, 22039086, 23168362, 23091097, 22052061, 21933835, 22538854, 22447964, 21890793, 22318095, 22412041, 22802322, 22498745, 22949154, 22236796, 22374636, 22373563, 22253393, 22455412, 22505744, 22282543, 22198714, 23069487, 22586173, 21896539, 21976386, 22374183, 23062542, 22056854, 23121378, 22883507, 22173910, 22265698, 21896540, 22855599, 21828378, 22965965, 22965961, 22318279, 21940785, 22452894, 22475493, 22370326, 22399699, 22806681, 22503032, 22674146, 22646631, 22334369, 22250063, 22496161, 23063316, 22726802, 22691567, 22508825, 23043090, 23008442, 22674920, 22446569, 22851406, 23122534, 22981555, 22966146, 22147903, 22885164, 23109696, 22575312, 22585697, 22417252, 22748590, 22748591, 22082706, 22340966, 23040570, 22446171, 22356323, 22612596, 22853014, 22551107, 22682536, 22581070, 21908494, 22936658, 22945622, 22802737, 22767583, 23117550, 22192488, 22930458, 22824435, 22258482, 22782510, 22968888, 23122801, 22146409, 22405255, 23190905, 22261362, 22154591, 22401913, 22571200, 22532639, 22837377, 22732313, 23168367, 22464340, 22169269, 22516444, 23136163, 22361397, 22830462, 22709688, 22467902, 22608570, 22700860, 22981547, 22566583, 23122802, 22075170, 22365424, 21852332, 22449295, 22773702, 22395644, 22472546, 22735384, 22714001, 23180503, 22160483, 22955728, 22589472, 22364685, 22670901, 22396394, 22093187, 22430269, 22619083, 22120766, 22474203, 23197596, 22734013, 22128223, 22461416, 22323575, 22228446, 22679142, 22885329, 22436955, 22497929, 22581157, 22071326, 22402068, 23045214, 22408266, 22649130, 22042946, 22393129, 22064876, 22523314, 22773036, 22397651, 22442350, 22431675, 22271480, 22492586, 22184381, 22616830, 22564993, 22277570, 22624834, 22297666, 22748820, 22491790, 22456475, 22323482, 22446502, 22128084, 22738096, 22396514, 23059199, 22340672, 22626741, 22732747, 22341825, 22815653, 22618925, 22435370, 23128163, 22633824, 22665103, 22751755, 23212498, 22735431, 22535855, 23142059, 23232895, 22351711, 22782417, 22782416, 22450428, 22228743, 22901620, 21719490, 22610520, 22473435, 22915730, 22267599, 22910755, 23040580, 22751874, 23168823, 22893167, 22431131, 23066166, 22822007, 22965977, 22618923, 22433279, 22215165, 22664795, 22203758, 22692171, 23212500, 22338103, 22550196, 23045213, 22088966, 22482940, 23073950, 23048011, 22248659, 22473769, 22438367, 22938716, 22787176, 23169801, 22584134, 22166903, 22927555, 23089999, 22034645, 22801933, 22157067, 22294737, 22446739, 22474202, 22430275, 22815298, 22781424, 22683134, 22893165, 22797645, 22349588, 22723612, 22706833, 23165659, 22396515, 22454089, 23129008, 22968527, 22447880, 22798322, 23032549, 22494826, 22071248, 23212499, 22539172, 22285055, 22589500, 22797843, 22735103, 22385683, 22430268, 22302780, 22842354, 22767614, 23212497, 22267762, 22614993, 21969363, 23083772, 23165660, 22464260, 22923669, 22408034, 22577186, 23126252, 22446170, 22410815, 22735432, 22851113, 22248661, 22699287, 22869065, 22820788, 22498739, 23129601, 22301766, 22374639, 22665104, 22752356, 22240497, 23103036, 21917649, 22275296, 22617188, 22196945, 23036896, 22277837, 22297244, 22210563, 22036776, 22153890, 23060624, 22015077, 22727733, 22947344, 22851600, 22301126, 22432104, 22936892, 22441743, 22459549, 22683226, 22484118, 23208165, 22323576, 22923662, 22933567, 22748702, 23045575, 22643350, 22405251, 22266602, 22807076, 21949007, 21427067, 22282552, 22216840, 22267768, 23141812, 23141813, 22056021, 22913681, 22971323, 22418740, 22067089, 22416101, 22245666, 21920964, 22130488, 22819864, 22894573, 21996342, 22508732, 22677046, 22538330, 22285168, 22801672, 23008295, 22777514, 22416059, 21917824, 21798891, 22179538, 22064878, 22149876, 22951305, 22327223, 22338102, 22581236, 22337714, 21920963, 22683137, 22858387, 22990269, 22399700, 22565934, 22036871, 23037558, 22674921, 22370325, 23021325, 22422824, 22368231, 23017669, 22366046, 22321770, 22271764, 22459892, 22226929, 22503057, 22778317, 23078958, 22331954, 22539562, 22370314, 23128104, 22904565, 22219013, 22005922, 22095545, 22986757, 22924638, 23113482, 22440819, 22281833, 22951084, 22296077, 22513778, 22512843, 22192731, 22730539, 22955918, 22633322, 22179955, 22538805, 22051697, 22395923, 22773704, 22584796, 22052985, 22432105, 22012970, 22121129, 22459542, 22128083, 22119930, 22818074, 22291207, 22972968, 22751879, 22289887, 22040841, 22095398, 22318926, 22511260, 22250141, 22148098, 21652583, 22939758, 21997547, 22128143, 22187510, 21852334, 22177579, 22738085, 23075127, 22851561, 22858558, 21756847, 23136160, 22851563, 22677045, 22541248, 22898679, 22218098, 22615342, 22260988, 22318278, 22071249, 22258366, 23147173, 21471562, 22128081, 22269590, 22751847, 23017533, 22915643, 22285579, 22232347, 22440214, 22922507, 22829023, 22550156, 22618094, 23070488, 22619082, 23020132, 22570370, 22751757, 21930734, 22460142, 23103663, 22894553, 22169268, 22541389, 22529257, 21525406, 22545024, 21810729, 22436957, 22244150, 21994233, 23083787, 22624833, 22553307, 22945832, 22383680, 22822019, 22417254, 22610452, 22670904, 22527062, 22357140, 23043166, 22521071, 22521072, 22933438, 22683131, 22387529, 22210578, 23104212, 22893166, 22301127, 22872695, 22723577, 22723583, 22931259, 22172305, 22479155, 21885390, 22753922, 22692651, 22851599, 22920912, 22447888, 22336189, 22585689, 22464647, 22335736, 21911655, 23117776, 22297080, 22753904, 22575311, 22192729, 22578914, 22547592, 22859709, 22064877, 22637544, 22942337, 21990263, 21846677, 22735306, 22375970, 22332155, 22782199, 22274684, 22257523, 22257673, 22492697, 22987089, 21997554, 22571201, 22571202, 22527079, 22529180, 22455415, 23075177, 22539783, 22723581, 23212058, 22042967, 23171095, 23140761, 22431673, 22128082, 22355055, 22198974, 22172244, 22308289, 22187085, 22649136, 23008300, 22541245, 22442396, 23150707, 22936795, 23129602, 22533576, 23121403, 22393128, 22108194, 22893265, 22633825, 22889759, 22341744, 23045591, 22541418, 22851598, 23083673, 21987394, 23129488, 22236802, 22901886, 22344613, 22492584, 22711171, 22658702, 23032552, 21749847, 22875911, 22331940, 22552194, 21980193, 22507979, 22984354, 22388325, 22025095, 21828036, 22297243, 22633653, 22135277, 22915615, 22473163, 22665541, 22689805, 22654006, 22001391, 22636824, 22572916, 23162860, 23117775, 22408263, 22215856, 22192670, 22764364, 22997204, 22120765, 22265697, 22276820, 22564997, 22633362, 22885649, 23101118, 22949149, 23268664, 22851566, 22719231, 22716976, 22699418, 22577222, 22479156, 22825283, 22322237, 23122795, 22453654, 23045588, 22412073, 22529265, 22354598, 22389254, 22592900, 22449296, 22452356, 22507886, 22464310, 22018760, 22892337, 22453568, 22307571, 22523305, 22516441, 23008325, 22108830, 22678167, 22464674, 22704583, 22808958, 23075701, 22510874, 22371876, 22473162, 22443460, 22452807, 22075171, 22689807, 22100962, 22565936, 23123071, 22564353, 22088831, 22595799, 22431674, 22383118, 23109689, 22505276, 23008509, 22596166, 23011712, 23075175, 22700854, 22371069, 22767584, 22149875, 22689317, 22083873, 22554598, 22927525, 22203755, 22357255, 22184384, 22331946, 22473155, 22851564, 22341632, 21885393, 21965226, 22370330, 22981558, 22074724, 22652183, 22992073, 22992072, 22375973, 22417253, 22873530, 22427749, 23093664, 22575316, 23117779, 22305678, 22464343, 22269594, 22676937, 22591948, 23084481, 22920930, 21998118, 22361329, 22197676, 23104210, 22331951, 22784040, 22615543, 22256805, 22627104, 22646630, 22493419, 22529255, 22566538, 22307831, 22566517, 21890814, 22556050, 22915657, 22508813, 21969418, 22341824, 22760010, 22341736, 21997545, 22162577, 22377180, 22240495, 22473153, 22751877, 22682553, 22527063, 22078337, 22534058, 23103033, 22228146, 22417251, 22975340, 22128076, 22475593, 22493417, 23151431, 22336221, 22030902, 22753306, 22742399, 22858390, 22808955, 22431703, 22512481, 22889955, 22682555, 22282548, 22308301, 22665534, 22753906, 23043083, 23091096, 21633047, 22753918, 22112969, 22547605, 22508830, 22565002, 22430274, 22162575, 22370319, 22271474, 22124104, 22271472, 22665535, 22868933, 22213436, 22945623, 22421303, 22851558, 23008301, 23252525, 21969420, 22231041, 22184370, 22454414, 22965960, 22915652, 22422823, 22987083, 22949150, 22991449, 22544804, 22414766, 21893487, 22614994, 22377805, 22698488, 22291074, 22614972, 22480758, 22959335, 22688548, 23131066, 22703927, 22688551, 22905354, 23109708, 22307201, 22494956, 23075176, 23062530, 22245896, 23212996, 22560018, 22456770, 23011728, 22880214, 22626743, 23248063, 22971521, 23268518, 23091107, 22075478, 22529236, 22206777, 22857850, 23108941, 22505632, 22822024, 22432114, 22155172, 23070486, 22652344, 23177296, 23091101, 22402056, 22077192, 22998716, 23062232, 22460854, 22463922, 22562021, 22192672, 23051991, 22307942, 22357185, 22892585, 22658127, 22569484, 22677258, 21917822, 22412140, 22649143, 21836029, 22704366, 23040422, 21737561, 22674807, 22835668, 22595313, 22362844, 22335737, 23018640, 22584132, 22291076, 22139751, 22039165, 22658758, 22987084, 22744718, 22100963, 22302316, 22586281, 22178268, 22039087, 22232535, 22438530, 22312103, 22040840, 22952075, 22713626, 22982183, 22240407, 22830463, 22878166, 22285578, 22826271, 22179532, 23236031, 22318635, 22267245, 22124771, 22294641, 22932715, 22412143, 22787172, 22633033, 22213489, 22538329, 22863328, 23159551, 22285278, 22231174, 22225814, 22665030, 22116817, 22231607, 22179530, 22039079, 22951082, 23121323, 22551127, 23159552, 22318199, 22236222, 22884505, 22239915, 22564989, 22791289, 22585692, 22011409, 22090168, 22990273, 21610154, 22253394, 21969362, 22338101, 22012969, 22659218, 22645292, 22619107, 21948942, 22424722, 23109694, 22315282, 22818063, 22316445, 22374408, 22271481, 22405632, 21963761, 22951287, 22901955, 22665533, 22982184, 21827730, 22075488, 22578793, 22877848, 22065252, 22430270, 22986377, 22393219, 22374642, 22932717, 22110122, 22632908, 21960535, 22381428, 22508731, 22425702, 22473672, 23028263, 23153843, 22326464, 22539173, 23128568, 22517917, 22282542, 22196768, 22453648, 22446172, 22761293, 22374696, 22944873, 22496156, 22312173, 22910840, 22071384, 21832285, 22101900, 22284330, 21856681, 22006408, 22450429, 22440219, 22130425, 22096248, 23024023, 22877506, 22831781, 22180438, 22622008, 22716975, 22077816, 22572911, 22789884, 22957521, 21700602, 22938706, 22121130, 22873531, 22894574, 23121377, 22027579, 23113480, 22322126, 22517930, 22945620, 21840117, 21737565, 22443478, 22728514, 22377561, 23020162, 22021897, 22162570, 22873532, 22613001, 22086879, 22932714, 22371872, 22948049, 22128078, 22312175, 22190676, 23252526, 22443479, 22296076, 22296075, 23131078, 22460067, 22619193, 22898076, 22585691, 22156624, 22869325, 22300693, 22695903, 22344610, 22344611, 21975269, 22893231, 23075178, 22344040, 22898678, 22245891, 22025146, 22370318, 22473713, 22751878, 23027801, 22883632, 22345126, 23103035, 22561055, 22234907, 22077070, 22337679, 22932716, 22443427, 22551105, 22412149, 22547591, 21562072, 22878279, 22393132, 22656335, 22730101, 22355040, 23147455, 23177300, 23547079, 22753403, 23871490, 23294853, 23665364, 24140390, 23764181, 24130362, 23517118, 23319069, 23357715, 23213105, 24026257, 22975754, 23348980, 23041585, 23747768, 23268365, 23260188, 23784521, 23575810, 23429913, 23656642, 24251361, 23599435, 22859796, 23439259, 23591789, 23883379, 23040208, 22743678, 23975663, 22902362, 23864098, 24163364, 23791513, 23072780, 22677718, 22717454, 23687259, 23687260, 22760005, 23139262, 23413265, 23128366, 23627345, 22821361, 23292882, 23916928, 23098762, 23297129, 23463624, 23986090, 23002282, 23406728, 23788755, 23242192, 23692173, 23992557, 24251360, 24251363, 23594003, 23688301, 24088091, 23500267, 23436910, 24153411, 23000088, 23104724, 23788751, 23510984, 23757424, 23643110, 24054816, 23944299, 22653766, 23394476, 23902482, 23846759, 23044532, 23478059, 23228172, 23374650, 23509310, 23648948, 24086114, 23420231, 23238657, 23999574, 23640009, 23860204, 23628617, 23564915, 22562983, 23196701, 23295789, 24089054, 23850491, 24058300, 23932548, 24104372, 23246022, 24019551, 23234763, 23715567, 23071237, 23041591, 22857983, 23742877, 23689381, 24126646, 24067488, 23994420, 23992602, 23863050, 23870813, 23884353, 22961727, 23265995, 23541756, 23460053, 23250992, 23315161, 24429126, 23306100, 23081753, 24429201, 23363496, 23995608, 23982301, 23680817, 23389382, 24035250, 23769234, 23831050, 23542898, 24622416, 23454058, 23918952, 23216615, 22984174, 24002506, 23104718, 24369076, 23518156, 23440173, 24012983, 23732713, 23193208, 24461666, 24429244, 23929355, 23315907, 23945905, 23258530, 24089540, 23144444, 22915617, 24019763, 23547084, 24101054, 23658438, 24171516, 23403978, 23223772, 23973694, 23222879, 23569311, 23491275, 24206640, 23598172, 24140184, 23770182, 24206459, 24061784, 23514730, 24321801, 23979005, 23846810, 23433739, 23104720, 23644091, 23835684, 23487517, 23193209, 23233715, 24028813, 23852309, 23312888, 23108952, 23973701, 23684673, 23374649, 23817631, 23349444, 24171490, 23614585, 24081433, 23726159, 23768747, 23905525, 23616624, 24170749, 23504926, 23609186, 24043739, 23980090, 23980084, 23998657, 23369417, 23169517, 24002501, 23721753, 23545757, 23564919, 23412078, 23594786, 23463625, 23358884, 23388213, 23998714, 23583246, 23953384, 23796131, 23219296, 23216616, 23465742, 23819964, 24050808, 23877979, 24016454, 23863909, 23460705, 23471421, 24130356, 23924878, 22898037, 23435160, 23670995, 23474589, 23644090, 23349132, 23656980, 23348977, 23735727, 22589376, 23623280, 23939263, 23407784, 24045740, 23652861, 22778174, 23018904, 22956509, 23676420, 23746666, 23460709, 24162942, 23959152, 23161902, 23602769, 23537606, 23778903, 23821661, 24114306, 22562973, 23838349, 23782178, 23333143, 23803136, 23193217, 23033237, 23514286, 24066743, 24368463, 23219570, 24026523, 23500260, 23550668, 23932438, 23685940, 24206457, 23380743, 23537605, 23623998, 23921906, 23043164, 23400279, 23553060, 23470859, 23490040, 23664370, 23499546, 23927582, 23946328, 23500251, 23623915, 24030420, 24429245, 23065731, 24115912, 23414648, 23414650, 23271794, 23925619, 23474283, 23168366, 23738545, 23439102, 23886915, 24030409, 24162943, 24166522, 23520339, 22733833, 23946263, 23277307, 22700844, 22719022, 22964169, 24043299, 23724913, 23500312, 24128861, 23991661, 23562009, 23363666, 24170747, 23599046, 23896732, 23801734, 23821759, 23940225, 23523674, 24043751, 23002085, 23684680, 23393209, 23412077, 23897038, 23752780, 24067398, 24075361, 23620478, 23683464, 23939297, 23141486, 23382656, 23315603, 23498594, 23871417, 24211163, 24231627, 23223332, 23377641, 23590271, 24195548, 23830355, 23578722, 24101040, 23726041, 24130354, 23514733, 24490266, 23797811, 23722616, 23715754, 23323867, 23688323, 23176897, 23306586, 23644079, 23770132, 23358690, 23689848, 23810152, 23726393, 24209829, 23877591, 24251359, 23409290, 23152362, 23752109, 23478743, 23999456, 23999933, 23549582, 23757082, 24002499, 23779114, 23532241, 23516146, 23033246, 24302090, 22935549, 23385272, 24327037, 24130357, 23388005, 23632722, 23532240, 23549581, 23587564, 24002794, 23695482, 23665370, 23512062, 23695483, 23425564, 23690755, 24108526, 23233651, 23766483, 23821088, 23915882, 23976883, 23916925, 23954450, 23564916, 23757437, 23500254, 24461612, 23299606, 24002280, 23972263, 23652522, 23532242, 23280223, 23500269, 24281460, 24041682, 23380218, 23821397, 24368464, 23321763, 23770175, 24150470, 24026600, 23478662, 23473369, 23340588, 24247554, 23602230, 23385271, 24150466, 23538918, 23423753, 24065010, 23273292, 23592107, 23839751, 23443441, 24108510, 23183941, 23444424, 24281461, 23529771, 23578528, 24014831, 23791822, 24461665, 23247302, 23299607, 23440253, 23530022, 24240932, 23849948, 23683639, 24622263, 24130463, 23344135, 24130350, 24100934, 24048296, 24022033, 23727163, 24622367, 23360891, 24012319, 23303891, 22878873, 23471307, 24136634, 23158882, 24002278, 23690410, 22711752, 24062327, 23866823, 23219284, 24047455, 23740284, 23499390, 24151326, 24108515, 23280226, 23676183, 23404297, 24037226, 24026599, 24065013, 23915885, 23065358, 23564914, 23872605, 23439759, 23230096, 23704674, 23803700, 24144653, 23477676, 23689789, 23483175, 23924660, 23999454, 23644995, 23589097, 23780457, 23500230, 23453283, 23536105, 24042367, 23926207, 23277482, 23684411, 23935011, 23770179, 23139265, 22772328, 23640971, 22984171, 23183529, 23850055, 23158522, 24622318, 23942774, 23590265, 22730470, 23698396, 23536584, 24321802, 23234647, 24321804, 23307827, 23664333, 24622413, 23177515, 24429275, 22730366, 23117073, 23248197, 23727264, 23769296, 23182126, 23896675, 24097128, 23511717, 23704198, 23902481, 23364953, 24099601, 23726161, 23852610, 23142625, 24029165, 23583433, 23260167, 23963895, 24622369, 23583763, 24429129, 23567348, 23780458, 23390923, 23387822, 23193211, 24075051, 23594787, 23732774, 23883922, 23900058, 23295797, 23321216, 23948514, 23338569, 23661292, 24062397, 23500237, 23499726, 23312829, 24043745, 23608924, 23358971, 23141817, 23462665, 23643112, 23588877, 23180584, 23404304, 23485851, 23471469, 23579816, 22875412, 23117245, 23425163, 23485610, 23213075, 23567146, 23246393, 23546617, 23644179, 23944300, 23504636, 23359516, 23484827, 23479062, 23735723, 23863908, 22981787, 23473396, 23193213, 23141049, 23401447, 23733031, 23733779, 24239210, 23358972, 23559149, 23567902, 23935012, 23223405, 23514288, 23666915, 24142148, 24030410, 23902874, 23639612, 23414585, 23980091, 22773551, 23474363, 23467860, 23463857, 23233662, 23460711, 23980081, 24159176, 23448807, 23564922, 23491407, 22984173, 24024838, 23129737, 22966096, 23275372, 23689947, 23117071, 23749153, 23312704, 23413266, 23614586, 23868905, 24019074, 23831445, 24146218, 23894039, 23172975, 23233657, 24062400, 24002795, 23797954, 24076337, 23449426, 24070911, 23630210, 23033239, 24106935, 23339638, 23339639, 23857975, 23740278, 23821089, 24382638, 23664707, 23444397, 23144449, 24162940, 23948351, 23613255, 24101696, 23403476, 23352749, 23324563, 24002279, 23632723, 23312808, 23599320, 23393214, 23770172, 23945981, 23100282, 23995982, 23884439, 23213101, 23757426, 22915624, 23328954, 22814294, 23028039, 24152861, 23650412, 23903454, 23670660, 23277897, 23159770, 23319696, 23894178, 23922063, 23018901, 23525797, 23721957, 23222369, 23186806, 24173657, 23645837, 24131140, 23546562, 23706759, 24105303, 23500234, 23414589, 23341517, 22679301, 22739990, 23801725, 23959380, 24060942, 22753402, 22584648, 23881862, 23175622, 23357349, 23403597, 23700028, 23631796, 22496329, 24170762, 23229890, 23727083, 23103177, 24018647, 23735724, 23782814, 23220919, 23835717, 23732754, 23562090, 23589672, 23370827, 23550669, 23579179, 23541971, 24231273, 23864006, 24011548, 23060625, 23940221, 23466078, 23290547, 23596006, 23374268, 24225155, 23218662, 22661646, 23375965, 22886633, 24429154, 23063567, 23269986, 22858559, 24009303, 23152361, 24068776, 24127770, 23359491, 22619366, 23592708, 23932219, 23395398, 24095300, 23514728, 23599941, 23053173, 23902483, 24225157, 23040886, 23301731, 23321318, 23958540, 23948125, 24026560, 23704675, 23453347, 23288387, 22961576, 23738992, 23963894, 23828828, 24461664, 23492671, 23465517, 23219286, 24130343, 23797469, 23033241, 23661293, 23641006, 24047060, 23224207, 24490265, 24019546, 23677313, 23644088, 23842846, 23948349, 23182993, 24186878, 23040887, 23144450, 22887465, 23419284, 23999449, 24228710, 23564917, 23265346, 24247300, 24622415, 22562974, 23623914, 23306584, 23715753, 23690420, 23742699, 23172751, 23984728, 23682110, 24429155, 23484826, 23589556, 23732968, 23332236, 23358784, 22975755, 23048188, 23579178, 23942586, 23290598, 24084921, 23439632, 23673400, 23846733, 23690414, 24026316, 23232844, 23303315, 23378299, 23599318, 23733781, 24013058, 23578724, 22933433, 23525931, 23602084, 24002519, 23940231, 24126948, 23857075, 23340885, 23821090, 24089542, 24136883, 23139259, 24101048, 23378222, 23982225, 23666250, 23406026, 23433590, 23757762, 23502220, 23872500, 23648697, 23445093, 23477625, 23973689, 23449691, 23835688, 23846809, 23563134, 23281974, 23541975, 23393217, 23541458, 23835715, 23835718, 23843495, 23670096, 23477657, 23432142, 23810097, 23791510, 23021878, 23265344, 23608668, 23306000, 24074642, 24461613, 24429127, 23907700, 23275363, 23512759, 23218813, 23940227, 23562754, 23161899, 23358983, 23808982, 24490264, 24127444, 23739521, 23796998, 23149845, 22980443, 23500298, 23594592, 23391196, 23277105, 23293111, 24063860, 23982273, 23659733, 23206837, 24013512, 23406914, 23296629, 23643139, 23725851, 24024839, 23835707, 23169523, 22695241, 23543483, 24193079, 23643501, 23393144, 23417203, 24248687, 23897954, 23964934, 23343062, 24297189, 23635049, 23514285, 23428214, 23325525, 23953766, 23318559, 24120480, 24085766, 23233713, 22933432, 23704196, 23602601, 23777849, 23777763, 23522911, 23382472, 23108953, 22898035, 23295794, 23233710, 23478060, 23940216, 23690424, 23460714, 23235801, 23816960, 23248256, 23071247, 23592701, 23857972, 23569309, 23569308, 23683751, 23805899, 24022796, 24107292, 23386127, 23238663, 24145346, 23319574, 24007748, 23810874, 23630215, 23498850, 23358966, 23364679, 23429924, 23726851, 24062326, 23747759, 23953385, 24127448, 24262440, 23265328, 24035531, 24231454, 23715562, 23038758, 23683475, 23661722, 24084072, 24026598, 23864239, 23812339, 23965225, 23857233, 24126622, 23991622, 23129133, 23542112, 24005123, 23349296, 24005242, 23483174, 23630211, 23432189, 23230099, 23864097, 23440337, 23363495, 23341518, 24019539, 23410541, 24136890, 23687089, 23414791, 23357717, 22699455, 23940228, 24368465, 24140442, 23457213, 23688302, 23348636, 23610561, 23321257, 23715565, 23707067, 23460707, 23868858, 23136231, 23295793, 23479454, 24101043, 24004988, 23680854, 23863946, 23878298, 23500307, 23890779, 23456778, 23721752, 24129463, 23265334, 23500276, 23766350, 23758233, 23269991, 23703789, 22840353, 22859496, 23332882, 23491524, 23128427, 23212150, 23479138, 22961573, 23622121, 23897967, 23233708, 23569301, 23138961, 23811112, 23775967, 24043742, 23918953, 23340895, 23733756, 23195283, 23690416, 23467813, 24081946, 23733767, 23161898, 23091109, 23589560, 23788750, 23733761, 24101053, 23650416, 23341531, 23233719, 23547081, 23182987, 23129742, 23835708, 23775961, 23593932, 23650429, 23435159, 23396493, 23704089, 23686738, 23645886, 24043733, 23085096, 24101042, 23509322, 23129746, 23991625, 24081940, 23086138, 24200404, 24002495, 23980077, 24220555, 24190112, 23897968, 23713578, 23699763, 23704672, 23541974, 23410544, 23690531, 23530110, 23830226, 23041328, 23628620, 23525934, 23824214, 23735726, 23835709, 23277305, 23897970, 23602776, 23177514, 23234725, 23801735, 23246389, 24014834, 23973688, 23916935, 23223803, 23212720, 23966309, 23690533, 23500314, 23100508, 24368466, 23504694, 23172972, 23715579, 23656644, 23117243, 24002511, 23553752, 24130355, 23572499, 23669281, 23233721, 23715582, 23697514, 24004119, 24140183, 23841729, 23423673, 23775260, 23883377, 23883378, 23927913, 23708054, 23261355, 23403682, 23769235, 23293082, 23960180, 23615461, 23547078, 23388003, 23500262, 23204255, 23150720, 23857320, 23473847, 23736733, 23995984, 23810881, 23391465, 23877988, 23174659, 23123509, 23665341, 23369412, 23713082, 23817699, 23992601, 23999656, 23440308, 23598181, 23737376, 23759326, 23715572, 23318311, 23735514, 23200175, 24127442, 23158886, 23141816, 23816964, 23904552, 22975753, 24159565, 23500245, 23685743, 24041978, 24139639, 23751902, 24141424, 23715755, 23323897, 22878879, 24140677, 23695200, 23315904, 23406672, 23429918, 23742912, 23172749, 23489754, 23323901, 24090588, 23583255, 23764350, 24026317, 23183528, 23982366, 23607593, 23607594, 23499158, 23499440, 22831853, 23739063, 23897964, 24103444, 23358975, 23425947, 23333117, 22315471, 23694965, 23900047, 23343564, 22739993, 23811305, 23382470, 23490041, 23949558, 24075050, 23265698, 23168021, 23633524, 24081937, 23732711, 23248249, 23471803, 22562977, 23506518, 24047061, 23219086, 23583245, 23183521, 23940461, 23816967, 23836616, 23284036, 23268367, 24050956, 24237006, 23718152, 24130358, 23863051, 24622414, 23403680, 23709583, 23361064, 23683600, 23583604, 22964882, 23591273, 23747777, 23018151, 23086750, 23212717, 23835687, 23380890, 23689475, 23536583, 23853552, 22297124, 23060628, 23397296, 24346989, 23689765, 23349548, 23851398, 23193218, 23892201, 23223345, 23002086, 23471512, 23275364, 23160726, 23340894, 24030613, 23765237, 23139373, 23229914, 23589670, 23864130, 23974194, 23602770, 23835693, 23770747, 23485520, 23545598, 23265332, 23384679, 23635051, 23378537, 23500313, 23265340, 22865778, 23144046, 23715097, 23230135, 23920083, 23528626, 23576128, 23389323, 23018908, 23352054, 24055415, 23585513, 23755969, 23589673, 23761134, 24177257, 24174625, 23789889, 23991656, 23312702, 24311990, 23992515, 23182627, 24019545, 23515142, 23294500, 24026258, 23388004, 24132249, 23760393, 24166518, 23512758, 23999980, 23817921, 23281973, 23229846, 23823157, 23761189, 23349407, 23473338, 23534542, 23648946, 24429202, 23395119, 23796987, 23277304, 23453583, 24095299, 23358973, 24026296, 24097234, 22700845, 22967997, 23775972, 23900590, 23415013, 24043732, 23696515, 24328444, 23954314, 23583250, 23821401, 23355624, 23796235, 23860985, 23964933, 23964932, 23600452, 23498849, 22810757, 24130351, 23160722, 23845716, 23501976, 24055414, 22997217, 23979627, 23525929, 23361631, 24461758, 23853057, 23275352, 23973699, 25402400, 24331152, 25145522, 24572077, 24530680, 24311766, 24385534, 24100783, 24311763, 25081901, 24678979, 24973281, 25493978, 24861959, 24114969, 24678939, 25091788, 24780211, 24399428, 25337748, 25089931, 24457205, 24934276, 24595632, 24784832, 23791392, 24113319, 24637560, 23606682, 24048736, 24866029, 25237196, 23434567, 24836312, 25139550, 24631949, 24452751, 25193393, 24215940, 24216285, 24637951, 24618266, 24246407, 24334242, 23904473, 25070546, 24894026, 25168619, 24412457, 24814486, 24667715, 24480899, 24170758, 24211510, 24275909, 24351402, 25064437, 24504445, 24603643, 24256848, 25251616, 24804697, 24802775, 24552317, 24583294, 24988555, 24401049, 24693891, 24556911, 24382064, 24635773, 24848284, 24743802, 24434503, 24255129, 24928832, 24847155, 25186259, 24671165, 24497255, 25386945, 24450889, 24552319, 23716067, 25071075, 25242048, 24632280, 24818763, 24795200, 24725237, 24827123, 24622668, 24534599, 24855210, 24647680, 24799467, 25163905, 24974051, 25304851, 25268438, 24442928, 24825371, 23525574, 23897775, 24067881, 23316080, 24585721, 24168956, 24745696, 25439693, 24881463, 24680359, 25035292, 24906040, 24198300, 24898300, 24439929, 24168957, 24635772, 24568894, 25078310, 24616335, 24501005, 25078304, 24630419, 24906822, 24161333, 25301455, 25004917, 24126705, 24571755, 25017001, 25465642, 24333009, 25399731, 25176015, 24513442, 25066248, 25466242, 24950985, 24119840, 24130347, 24470281, 23461895, 24694531, 24691094, 25034862, 24144788, 25282564, 24323030, 25225424, 25549086, 25069991, 24687165, 24767875, 25156992, 24679062, 24485709, 25014688, 23765873, 24799482, 24915873, 24361242, 25157725, 25190230, 24179073, 24980489, 25173754, 24140630, 25096697, 24449321, 24101699, 24595778, 24613349, 24836205, 25405392, 24717640, 24771721, 24963566, 24924991, 24497345, 25058083, 24565698, 25162920, 25184862, 24742662, 24746000, 25154045, 24674149, 24679060, 24947791, 25111658, 24705615, 25301457, 25125505, 24516018, 24837306, 25208464, 25306557, 25286173, 24637997, 25273342, 24552318, 25332247, 24170388, 24849104, 24291283, 25048382, 25399733, 24530176, 24026563, 24449241, 25446057, 24937643, 24850280, 25179753, 25096689, 24535702, 24996197, 24918373, 24440672, 25090173, 24135831, 24722496, 25071111, 25385735, 24981955, 24799484, 24626435, 24582486, 24731674, 25104521, 24291366, 24135832, 24898552, 25147079, 25161182, 25336745, 25456365, 25234803, 23911555, 24150216, 24439313, 24139497, 25516139, 24718885, 25163906, 24616308, 24888818, 24567516, 24892144, 24139977, 24315520, 25148838, 23974954, 25018116, 24748630, 25443696, 24179072, 25220861, 24473673, 24679061, 24788282, 24493100, 24508320, 25172965, 25157724, 24733277, 24733515, 25218906, 25037139, 24512909, 24997559, 24934962, 24399554, 25265492, 24711548, 25265494, 24985962, 24934845, 24722495, 25403218, 25250660, 24618964, 24682026, 25402398, 23853211, 24934787, 24948511, 24812432, 24968985, 24521108, 25500229, 24821885, 25189288, 25399273, 25011720, 24703832, 24315521, 24577287, 24449243, 23959566, 24361112, 25172769, 24603308, 25114296, 25323260, 25140956, 24742740, 24925924, 24849105, 25465416, 24958624, 25372087, 25085960, 24296850, 24657991, 24480619, 24918372, 24918371, 24859205, 24963567, 24638013, 24982464, 25517706, 24428467, 24656609, 25439692, 25439691, 24979446, 24144654, 24873720, 24963110, 24439238, 24297946, 24948656, 25091790, 24556041, 24603565, 25136078, 25282284, 24184147, 24939817, 25107467, 23932338, 24631162, 24622750, 24844728, 24140678, 24493300, 24760262, 24585267, 24879844, 24077657, 24411639, 24332238, 24567366, 24170757, 24014392, 24449819, 25311218, 24658598, 24590635, 24556040, 24733799, 24618336, 25294887, 24411709, 25381393, 25301459, 24176997, 24783988, 25037988, 25482871, 25006719, 25317759, 24780614, 24076283, 25216506, 24335106, 23904470, 24559581, 24292873, 24464358, 24626738, 25329446, 24311723, 24104875, 24810491, 25409371, 24887018, 24430319, 24549550, 24622752, 25268437, 25145775, 25018120, 25456367, 24461901, 24682069, 24700254, 24505216, 25536255, 24094767, 24797294, 25212646, 25456755, 24060855, 25111880, 24696094, 23942868, 24549548, 25142196, 24912901, 25173516, 24756128, 25536254, 23942869, 24784827, 24867013, 25247517, 25104109, 25058216, 25058218, 24825642, 24969577, 25265449, 24868083, 25117129, 24794367, 24170751, 24746755, 24655729, 25268295, 25003802, 25267796, 25172514, 24794703, 24947106, 25453458, 24865166, 24929428, 25536256, 24687169, 24932893, 25742320, 25127405, 24852376, 25038356, 24502830, 25138332, 25533442, 24825641, 24846036, 24287186, 25005651, 25524333, 24240712, 25042290, 25249453, 25311220, 24836273, 24277055, 25182100, 23396509, 25049325, 24963111, 24912589, 25402757, 25157723, 24938562, 25301463, 25402495, 24866862, 25038357, 24804695, 24419119, 25163699, 24957844, 24756512, 24563518, 24838406, 24381967, 24612754, 24824051, 24616337, 25402403, 25209620, 24821270, 24846035, 24515353, 24718270, 24290286, 25255799, 24589566, 24636210, 25243536, 24846652, 24898834, 24445955, 24473592, 25271544, 24602844, 25014686, 25514303, 23300117, 24296904, 25027140, 25006720, 24240777, 25088437, 25453457, 25178568, 24315706, 25134117, 25018057, 24300437, 25043514, 24170759, 24887304, 24461582, 24993098, 24795252, 24169081, 24781960, 24462735, 24360787, 24780501, 24859203, 24232701, 24942631, 25190523, 24291168, 24099659, 23475983, 24582316, 24569032, 24561548, 24211508, 24877997, 24694983, 24323795, 24879836, 24842985, 24742660, 24795251, 23313811, 23740226, 23696633, 24255061, 25231896, 24881993, 24608196, 24836310, 24641941, 25125506, 25439694, 24507091, 24895454, 24302273, 23361084, 24388010, 23852695, 24482301, 25048383, 24461903, 25072396, 24835833, 25285539, 24522178, 24629994, 23870670, 25155070, 24184132, 24013647, 24999157, 25119609, 24657484, 24740528, 24838360, 25002731, 25549238, 24262278, 24726066, 23625982, 24268590, 25064594, 24892763, 24817188, 24347520, 24071762, 24332218, 25005652, 24768678, 24571754, 25189358, 25057173, 24344222, 23792625, 24161338, 24742658, 24126097, 24507377, 25282563, 24722494, 25106761, 23900983, 25348000, 23812324, 24418950, 24101698, 25514302, 24881730, 24907494, 24347519, 24731671, 25127679, 25175099, 24850754, 24581940, 24309370, 24814090, 24815501, 24408401, 23723317, 25456370, 24742739, 25231953, 24731672, 24194508, 24478323, 25376862, 24667460, 24291239, 25104523, 24647464, 24787471, 24315894, 24759852, 23884638, 24257969, 24026542, 24176144, 24613024, 24716681, 24727254, 24827128, 24317176, 24356622, 24718886, 24356620, 24521106, 25402401, 25470694, 25277614, 24700706, 23334213, 24508070, 25328954, 24440473, 24131861, 25088940, 25234206, 25196020, 25287821, 24794375, 25176289, 24798675, 24757229, 24973361, 24735962, 24504441, 24190578, 24598244, 25054836, 24669014, 24950987, 25102853, 24954781, 24852116, 25092781, 24652989, 24291656, 25329201, 24598243, 25011946, 25173752, 25092686, 24929431, 24583059, 25272316, 25142484, 25294786, 24679469, 24856027, 25117155, 24898304, 24804699, 23962455, 24728329, 24658389, 25189213, 24428466, 24817030, 24784879, 24645942, 24510314, 23873583, 24677195, 24667714, 24718568, 24635770, 24894577, 24948586, 24297940, 25337749, 25190674, 25096691, 25125300, 24829362, 24226808, 24055845, 24613084, 24943065, 25513807, 25168125, 24958820, 25066331, 24915259, 24668862, 24582471, 24378475, 24881631, 24450857, 25291578, 25147253, 24982457, 24610807, 24632279, 25440099, 24492859, 25348858, 24184169, 24872540, 24458439, 24316517, 24361322, 25349296, 24858423, 25490549, 24443442, 25239625, 24239662, 25403215, 24275900, 24525446, 24812434, 25423041, 24184245, 25131977, 24513589, 24184254, 24929430, 24031025, 24478320, 25095886, 24982459, 24552320, 24608198, 23524886, 24217336, 24190691, 24616307, 25257633, 24615500, 24685829, 24914244, 25524339, 25225436, 25139884, 25064436, 25366684, 25481829, 24898301, 24290404, 23878167, 24736370, 24904116, 24443059, 24572997, 25184864, 24625200, 25062783, 24795254, 24796339, 23957945, 25005650, 25337750, 24170763, 24366103, 25022831, 24862029, 23897769, 25359348, 24998009, 25175478, 25175097, 25311217, 24831989, 24344215, 24623024, 24993911, 24703531, 25212745, 25247995, 25247709, 24841975, 25262458, 24700332, 24515493, 24161233, 24036024, 24535643, 24012203, 24737258, 24639423, 25271602, 24501094, 25369488, 24831977, 24132190, 24561145, 24835842, 25176136, 24716610, 24356594, 24886787, 25046847, 24829204, 24415445, 25243839, 24722498, 25328940, 25014687, 24868024, 25130998, 24645944, 24720702, 24725238, 24725239, 24302269, 25301831, 25184863, 24806159, 25006718, 25399012, 24824548, 24119318, 23990605, 25011952, 24243886, 25249651, 24139708, 24658390, 24290843, 24435414, 24694530, 24781374, 24569031, 25124692, 24065463, 25171195, 24295873, 23839541, 25236509, 25016974, 24569263, 24807084, 25205802, 23918037, 24523666, 24756515, 24930728, 25401325, 24861828, 25011949, 25264948, 24198302, 25270275, 25282285, 24170221, 24170180, 24914136, 24585722, 25273343, 23740231, 24399553, 24657685, 25320240, 24658769, 25060372, 25122671, 25078901, 24711047, 24240611, 24268105, 25225689, 25199059, 25038431, 24682346, 24898303, 24622715, 24135389, 24269926, 25225698, 25176944, 24395850, 24333184, 24352377, 24245543, 24778393, 24375768, 24831438, 24627416, 24832476, 24461900, 24315907, 25075930, 25001494, 24989152, 24799480, 24953021, 24731535, 25140958, 24247275, 24326741, 24355800, 24722493, 24430917, 24896818, 25003980, 25199624, 24862623, 24862342, 24881803, 24657003, 24794243, 25074507, 24141323, 24356595, 25078900, 25193531, 25465417, 24627552, 23939953, 24947792, 24585265, 24818764, 24737272, 25066329, 25317990, 24638843, 24838102, 25111196, 24115225, 24474434, 24276777, 24290290, 24401022, 24852952, 24882434, 24637998, 23436336, 24698485, 24136330, 24703047, 24897082, 25018121, 25354103, 24247415, 25089860, 24837215, 24669015, 23482473, 25059747, 24419109, 24097439, 25267738, 24497338, 24631948, 25199060, 25140957, 24332514, 25127208, 25192851, 24461574, 25176395, 24786714, 24595629, 24140659, 24552831, 24931572, 24322569, 24757227, 24879841, 25201358, 24928083, 24521107, 25149412, 25267748, 25127231, 24301456, 24739896, 25242045, 24296848, 24650695, 24849084, 25260838, 24638003, 24618153, 25122693, 24769640, 24515422, 24687829, 24713186, 25070545, 24419108, 24687833, 24794721, 25328942, 25281710, 24845609, 25096595, 25399274, 25011722, 23661492, 25399007, 24744272, 25456363, 24333493, 25348002, 24521993, 24626789, 24833354, 24687088, 24428468, 25170121, 24227821, 24450890, 24344211, 25049330, 25048952, 24368400, 24827134, 23979965, 25349302, 24569914, 25049329, 24395863, 24297951, 24419239, 24638015, 25349301, 24401928, 25161294, 24799513, 24297374, 24450858, 24555904, 24183563, 24994622, 25347696, 24502959, 24998674, 24421329, 24130341, 25225693, 24590363, 25287825, 24474383, 25002723, 23661494, 24397981, 24816809, 24131826, 24702835, 24821476, 24970782, 24831979, 24084071, 24779680, 25178809, 23794465, 24433682, 25038355, 25175921, 25332253, 24401930, 24561392, 24573661, 24760263, 24103901, 24554232, 25330017, 24615217, 25450083, 25295709, 24269362, 24478399, 25328863, 24859364, 24530676, 25092278, 25082573, 24663049, 25288141, 24424513, 23900984, 24998121, 24681342, 24687830, 24755007, 25186261, 25287828, 22633317, 23954085, 24485548, 25093754, 25271746, 25268962, 25239590, 25328952, 24916506, 24974383, 25116294, 24711552, 24356636, 25222386, 25139882, 24743971, 23868932, 24597866, 24549549, 24668102, 24001888, 25439688, 24726095, 24094768, 24933332, 25240821, 23665990, 24788619, 24356624, 24669010, 23345601, 24623027, 25267740, 24368224, 24357403, 24419112, 25403211, 25029202, 24590644, 24470003, 25271603, 24449235, 24827127, 24723487, 24366937, 24516038, 25385727, 25154822, 24841974, 25245446, 24297945, 24297950, 25366680, 24934783, 24435025, 25038486, 24591201, 24444658, 25031274, 24827034, 25315207, 25282565, 24728035, 25096607, 24226805, 24590636, 24815499, 24687826, 23847109, 24332263, 25006120, 24888810, 24323035, 24948695, 24509284, 25205139, 24484762, 24560541, 25261199, 24854041, 25182250, 24937305, 24131637, 24140664, 24509273, 24290406, 24646034, 23994382, 24733354, 24170750, 24076296, 25181492, 25265976, 24647726, 23912798, 24371264, 23850254, 24905492, 24194506, 24322378, 24733807, 24613333, 24496805, 24819981, 24720679, 24062325, 24972708, 24700342, 24965569, 24637999, 24595631, 24716641, 24486089, 24224997, 25103176, 24827808, 25154829, 24965823, 24602760, 25372085, 25182247, 24493716, 24382002, 24942277, 25153538, 25002717, 25297013, 25297012, 25209738, 24685276, 25297016, 25142002, 25127173, 24680372, 24525316, 24703208, 25034562, 24508103, 25151225, 25455987, 25087476, 24794369, 24170766, 24929429, 24499812, 25261549, 24297381, 25226476, 25096690, 25108889, 24993530, 23436914, 25236346, 24982456, 24493730, 25007392, 25231095, 24578545, 24954805, 24935082, 24316514, 25291577, 24565955, 24177001, 25323254, 24561149, 24378686, 23385306, 24590634, 24161321, 25172376, 25130995, 24076297, 23910233, 25042199, 24184810, 25078309, 24602923, 24727123, 24907224, 24907225, 24908551, 24667461, 24836125, 24837690, 25268516, 24239324, 24705120, 24369114, 25035291, 25300863, 24897083, 24699939, 23598957, 25191967, 24595630, 24209977, 24795201, 24768112, 25332243, 25002722, 24562449, 25216508, 25222385, 24716680, 24184249, 25075834, 24560453, 24797423, 24637559, 25398492, 24245566, 25080284, 24211309, 25161043, 23735746, 23770005, 25369200, 24853585, 25328943, 24595864, 25285540, 24982463, 24332516, 25405390, 24273046, 25223482, 24576432, 25210016, 24585720, 24678999, 25103718, 25138333, 25372086, 24915612, 24908550, 24201303, 24622716, 24717919, 24982504, 24691606, 24717626, 24360369, 25245439, 23845721, 24323034, 23908187, 24893708, 24331154, 24909499, 24316262, 24067879, 25027139, 24449229, 25098487, 23503044, 24295850, 25203086, 24756520, 24835503, 24382690, 24497539, 24574355, 24914242, 25001475, 24392848, 23979914, 24285642, 24727022, 25193529, 24969648, 24948466, 24639426, 24076487, 25157929, 25156687, 24937476, 24333490, 25458200, 25459211, 23644549, 24722497, 24525910, 24722500, 24682844, 24609919, 24921688, 24622328, 24352484, 24255104, 25048381, 23982436, 24488498, 24573385, 24578358, 24255130, 24567515, 24939657, 25142708, 24356862, 24183564, 24382580, 24561148, 24291273, 24727884, 24420499, 24671945, 24963568, 24297949, 24941177, 24525690, 25008546, 24678937, 24291272, 24509270, 24331381, 24247587, 24666696, 25104527, 24435046, 24705119, 24827132, 24793816, 25332249, 24912899, 24690625, 25338187, 24622667, 24344086, 24146170, 25250737, 24067785, 24720703, 24727841, 24595547, 24757230, 24529697, 25271389, 24284287, 24562444, 24608200, 24752056, 24827136, 25399658, 24519768, 24621680, 25258143, 25164908, 24450891, 24687156, 25229916, 25189359, 23949963, 24747100, 25002178, 24356596, 24647231, 24401051, 25456368, 25022724, 24725468, 24794368, 24727258, 25266222, 24553909, 25127244, 24612661, 24797300, 24247616, 25060370, 24519039, 24798484, 25493974, 24838476, 25135997, 24760259, 24211500, 24064007, 24366359, 25249668, 24668103, 24318743, 23771989, 24760261, 25249652, 24906716, 25252721, 25196117, 24213952, 24470004, 25385729, 24322060, 24442886, 24627538, 26227188, 25835443, 25472864, 26271061, 26271063, 26432775, 25747582, 26055947, 25788231, 25788234, 25461690, 24794075, 25771249, 25693011, 25230593, 25445827, 25923551, 26187183, 25468567, 25632066, 25920401, 26232172, 25575541, 26200166, 26436208, 25972158, 25643304, 26154787, 24748629, 26488693, 25609246, 26352813, 26216383, 26148930, 25281416, 26091808, 26347110, 25458726, 26215946, 25689364, 26082085, 26139054, 25416329, 25676423, 25457497, 25001888, 25596158, 25837041, 26416895, 26341987, 25833957, 26116717, 26347109, 25997818, 25722381, 25467017, 26405092, 25908605, 25538174, 26330419, 25677311, 26547226, 25157500, 25629740, 26551272, 25517348, 26267623, 25830422, 26376136, 26605928, 25406308, 26390245, 26410620, 26132939, 25875257, 26028131, 25693013, 26488691, 25528358, 25998279, 26153496, 25538178, 25524478, 25858497, 26151090, 26405291, 25775048, 26558530, 24412895, 26465985, 26193126, 26448371, 25815420, 25601341, 25319061, 26718674, 25037189, 26436473, 26330412, 26381519, 26580997, 26247543, 26527774, 24817417, 26323938, 25348661, 26549589, 26321237, 26211828, 25608756, 25863562, 25480874, 26040499, 26324372, 26527776, 26417001, 25747273, 25840693, 26206146, 25495490, 26361969, 25592987, 25942722, 26132941, 26596672, 25589191, 26156651, 25892145, 25692915, 25853745, 26002889, 26524571, 26118427, 25981191, 26321593, 26116801, 26153271, 25726502, 25404659, 24443001, 25633661, 26256072, 25657106, 26359900, 25743173, 25906785, 26559317, 26028518, 25403646, 25567115, 26080338, 25468170, 25830421, 26251214, 26505596, 24555998, 25769911, 26265659, 25781440, 25684164, 25372846, 26222559, 26210642, 25605841, 26106009, 26271059, 25012156, 25946280, 25875256, 25770814, 25743855, 26384238, 25819484, 25281353, 25769361, 26551959, 26421634, 25630558, 26714320, 26034956, 26143444, 26181014, 26458124, 25611785, 26408274, 26501506, 26670970, 25726515, 26200980, 25701454, 25923549, 26699167, 25622149, 26369473, 25514556, 25538172, 26118315, 25938655, 25176939, 25851628, 25787199, 25800762, 26360283, 26077237, 25605741, 25638326, 25538173, 26474518, 26014294, 25882029, 25922063, 26022961, 26471805, 26338726, 26324049, 26477635, 25775052, 25878028, 25406300, 25926562, 25449207, 25738670, 26113687, 26385957, 25911172, 26181174, 25637340, 26169621, 25796459, 25677353, 26241598, 25803642, 26116485, 26296950, 25644096, 25835160, 26208996, 26429297, 26201299, 26406150, 25633090, 25877812, 25205142, 26324362, 25573882, 26385956, 26022815, 25715991, 25482145, 25284616, 26493793, 26474517, 26179201, 25539586, 25931244, 25620016, 25605838, 25569128, 24431394, 25795414, 25531343, 25930192, 26065986, 26056125, 26192398, 25411413, 26244877, 25957330, 25596660, 26596670, 25732163, 25882396, 25602496, 26564598, 26047975, 25176942, 25939894, 26059012, 25784660, 25319385, 25800753, 25795409, 25677354, 25785967, 26051236, 26336909, 25869284, 25169728, 26093641, 26282635, 26296562, 26359611, 25384017, 25468164, 25637937, 25499871, 26371113, 25783757, 25957224, 25428216, 26282659, 26414759, 25690005, 25998278, 25681466, 25706340, 25599346, 26062775, 25583754, 26092818, 25749502, 26202811, 24612659, 26398076, 25420207, 25419871, 26056177, 25895016, 26541915, 25496767, 26459273, 25524261, 26433819, 26046730, 25434967, 26122726, 25720622, 25751673, 26072109, 26307603, 26071094, 25754414, 25817472, 26291092, 24733191, 26216386, 25701560, 26347918, 26700834, 25623087, 26271057, 25139667, 25662415, 26559241, 26460306, 25600568, 26282661, 25887358, 26052677, 25790742, 26362172, 24737786, 25542620, 26283655, 25765698, 25788230, 25994742, 25583139, 24706006, 25695403, 26321103, 26037941, 25703086, 25080450, 25311593, 26196502, 26444729, 25489785, 25592197, 25852208, 26049550, 25970252, 26400825, 26144908, 25425475, 26360086, 25815419, 26015558, 26037798, 26489389, 26206073, 25603003, 25806661, 26255043, 26264550, 25016597, 26041765, 25457496, 25851385, 25858265, 24297379, 25772594, 25867075, 26291768, 25743937, 25416687, 25813692, 25897156, 25660917, 26527781, 26446728, 25472980, 25646891, 26360284, 25352655, 26062881, 26062880, 26476467, 25534388, 25908066, 25862439, 26575060, 26342731, 24786301, 26167637, 26465983, 25851383, 26461996, 25712456, 25862039, 25800768, 25660925, 25403645, 25971288, 25687352, 26529159, 26305648, 25803346, 26165396, 26444692, 26421861, 26156108, 25919526, 26010632, 25863524, 25593053, 25463545, 26075751, 25921377, 26303558, 26206075, 26403339, 26346155, 25722380, 25483598, 26551304, 25688779, 25668262, 26348751, 26010418, 26393847, 26301603, 25622303, 25499545, 25888263, 26547464, 26325557, 25935875, 26392095, 26479077, 25182248, 24667900, 24566799, 26362496, 25533966, 24368514, 26556051, 25179768, 25359354, 26441179, 26441180, 26393848, 26070589, 25918291, 26206144, 26624824, 26165398, 26179504, 25499543, 24366914, 26444879, 25573410, 26305649, 26720026, 25688781, 25898050, 26172895, 26559572, 26049552, 26460398, 26024502, 25567765, 25189602, 25684586, 25734732, 26241603, 25734733, 26338956, 26052984, 26501862, 26022006, 26137956, 26433318, 26372583, 26637812, 26114161, 25688780, 26197184, 26547357, 25972575, 25907157, 26418813, 25735317, 26372470, 25883244, 26111066, 25605469, 26201510, 26181634, 25792206, 25700386, 25527456, 24631409, 25798990, 26360083, 25526597, 25852206, 26018333, 26228031, 25732273, 25402214, 26536057, 24721903, 25898052, 26035291, 25401463, 26458258, 26303560, 25813773, 26282640, 25195547, 25788164, 25592198, 25853746, 26188003, 25500202, 26262795, 25784662, 25986447, 26423182, 26248676, 25467591, 25467560, 25589326, 25687353, 25773378, 24550168, 24951103, 25882325, 26486192, 25468945, 25655160, 25715416, 25773607, 26070913, 26231885, 25587645, 26089386, 25913272, 25758769, 25867538, 25873635, 24625625, 25820612, 25533774, 24834925, 26231288, 25892679, 26338971, 25651246, 26061837, 25365753, 25710923, 25916341, 26188394, 26363701, 25915102, 25499546, 26071347, 25771069, 26057703, 25907158, 25498847, 26261007, 26284720, 26511956, 25174863, 25975632, 25465108, 25500425, 25987694, 25498732, 25614171, 26248087, 26333778, 25448925, 25171902, 25234807, 26142048, 25795076, 25900782, 25897569, 26027881, 26077087, 26304905, 25157964, 26304875, 25819691, 26035255, 25669730, 26231455, 25851629, 25847941, 25919527, 25271206, 26378978, 26650153, 26547465, 25671797, 26092673, 25636341, 25011935, 25883095, 26416999, 25554404, 26458034, 25792638, 26381515, 26452709, 25668557, 26482278, 25421877, 25179766, 25367713, 25751701, 25822572, 26457558, 26156323, 26044854, 25799402, 24255546, 25308290, 25271207, 25988460, 25355716, 26441187, 26315981, 25887096, 26224811, 26003169, 25888086, 26039521, 25482832, 25482425, 25865864, 25361552, 25468160, 26022813, 26220945, 26164097, 26351345, 25857665, 26342427, 26141208, 25691677, 25627664, 25836986, 26114410, 26378637, 26105600, 24081439, 25713437, 26177074, 26216382, 25740286, 25605843, 25908603, 25403578, 26338525, 26039600, 25728933, 24285491, 26333474, 25179764, 26232170, 25982014, 26371134, 26610874, 26159065, 25862743, 26431823, 26488565, 26025128, 25769357, 25605861, 24395557, 25387477, 25738668, 26456905, 25909356, 25216510, 26537182, 25336746, 26014298, 25765696, 25434303, 26239086, 25851630, 26113648, 26333337, 26022239, 25702876, 25344361, 25209598, 25869405, 25980660, 25981908, 26008841, 25825410, 25428224, 26595748, 25919012, 25255696, 26379095, 26231624, 26065565, 24448345, 25784665, 26522334, 25656287, 26444221, 26639149, 26698878, 25964246, 25796362, 26072396, 26518765, 26050650, 25195169, 25817374, 25498218, 26387142, 26388534, 25832102, 26342424, 26093980, 25739878, 25577661, 25646809, 25125508, 24872377, 26304900, 25018037, 25718355, 25918346, 26227186, 25219348, 25361992, 26216931, 26541921, 26014295, 26261259, 25092775, 26486190, 26523821, 26541919, 25399551, 25877813, 25539672, 24942382, 25972574, 25531167, 25697494, 26001387, 25573500, 26318714, 26255045, 26358285, 26314532, 26493786, 25867659, 25545858, 25523407, 26275735, 25863654, 26304663, 26431718, 26180109, 26253730, 25282520, 25633662, 26308684, 26192873, 25488965, 26464212, 26084343, 25687344, 24818633, 25923552, 25701561, 26630142, 26088909, 26503196, 26429700, 26200978, 26392096, 25406306, 25987659, 25680558, 25436448, 26022817, 26112885, 26398070, 25925203, 25226850, 26026936, 26549586, 26057285, 25143342, 26280534, 25677355, 25491659, 25616646, 25677309, 26549714, 25784666, 26441181, 26209030, 25779558, 25683116, 25533656, 26014292, 25863559, 25846144, 25985734, 25773757, 25671254, 26482279, 25629739, 26398071, 25760354, 25499165, 25413377, 25488963, 25795408, 26512041, 25414155, 26630143, 26363985, 26107752, 25852210, 25179761, 25672894, 26377054, 25411159, 24366918, 25738667, 26393854, 26077235, 25760561, 25583761, 25183203, 25705872, 25748117, 25453443, 25399542, 25773268, 25823738, 25744003, 25938990, 25981758, 25934242, 25660225, 26483244, 26016823, 25550337, 26361971, 25794457, 26670971, 25862517, 26124485, 26408865, 25935877, 26650152, 25910800, 26255208, 25646186, 25620670, 26315978, 25918350, 26002193, 25467566, 25998582, 25961184, 26365989, 26370380, 26157076, 26084342, 25605660, 25791513, 26067687, 26028120, 25359382, 26033801, 26460660, 25414152, 25686304, 26241597, 25645353, 25833966, 26062774, 25922062, 26061836, 25305506, 25361981, 26342236, 25785968, 26239090, 26093979, 24534756, 26188211, 25512675, 25284802, 26392433, 26150444, 25383558, 26282654, 26373676, 26516121, 25732157, 25760804, 26501533, 25504995, 26405294, 26313558, 26045340, 25701171, 26522337, 26254683, 26538423, 26272770, 25467016, 25488186, 25844993, 25975474, 25515657, 25150159, 26084341, 25882987, 25891304, 25452452, 26062776, 25399552, 25795410, 26412456, 26028407, 26406148, 26053341, 25473939, 25414039, 26367349, 25468946, 26515660, 26282643, 26022945, 26330422, 26377300, 25481791, 25837829, 25706092, 25839406, 25864104, 25539730, 26164096, 26009229, 25609193, 26624850, 25636947, 25556937, 25847979, 25631070, 25842223, 26030264, 25668261, 26289639, 26189433, 25988461, 26135706, 25504992, 25701170, 25074688, 25773919, 26387144, 26066705, 25801579, 26720025, 26182172, 26189067, 26361154, 26382998, 25467586, 26215950, 25732161, 26466202, 26094712, 26094763, 26062777, 26030518, 25596659, 26565927, 25321149, 25273886, 25784663, 26359749, 25657022, 25713015, 26181019, 26092816, 26124478, 25415805, 25882986, 25504030, 25282519, 25261324, 24747867, 25269831, 26115796, 25891173, 26124486, 26112745, 26293315, 25664444, 26095867, 25212278, 26088180, 25992505, 25693012, 25524950, 26358287, 26070406, 26422722, 25766724, 25573533, 25355723, 26109630, 26169611, 25732165, 25605862, 25316259, 26527789, 25691671, 25787915, 25559811, 25624429, 25976288, 25559818, 26116315, 26133966, 26022948, 26466021, 25805645, 25785969, 26436963, 26527790, 25739671, 26314489, 26112886, 26407787, 25827032, 25664620, 25400062, 26314530, 25332252, 26049549, 26266842, 25400162, 25352653, 25901427, 25672274, 25583753, 25269383, 25651247, 26523919, 24870622, 25454611, 25727536, 26518090, 24812287, 25209657, 26404420, 24630685, 25576320, 25989386, 26141263, 26076313, 26002111, 26700832, 25185099, 25822284, 25506771, 25708419, 25372657, 25361982, 26002610, 25995301, 25277272, 26130119, 25368202, 26162609, 26124497, 26239087, 25607833, 26494524, 25832034, 26343840, 26116294, 25792557, 26412349, 25937539, 25583616, 25602358, 26164495, 25713429, 26249300, 26250495, 26383722, 26700836, 26240230, 25176615, 25486131, 25928649, 25791214, 25624432, 26095784, 25877855, 26718672, 26527788, 25665812, 26180108, 26073754, 25667293, 25452437, 25584431, 26438119, 25940724, 25733584, 25512434, 26147154, 26527777, 26202595, 26386123, 25559798, 25669832, 26282650, 25512461, 26077241, 26347107, 25897153, 25584004, 25713428, 26056183, 25515658, 26347106, 25688059, 25452447, 25239078, 25878120, 25908774, 26323937, 25859764, 26333366, 25766941, 25574801, 25970009, 25644279, 26304886, 25041849, 25705822, 25853743, 26422724, 25787197, 25970050, 26063762, 25458910, 26260733, 26282658, 26324329, 26246458, 26351344, 25452446, 26202594, 25395283, 26351350, 25688081, 26043884, 25387891, 25851632, 26091743, 26200977, 25406305, 25981818, 25481378, 26441182, 26359750, 26521770, 26253728, 25835440, 26203062, 26215945, 26436207, 26653621, 25660356, 26149841, 25736990, 26202042, 26376805, 26597128, 25956718, 26112044, 25901426, 26282657, 26321318, 25395036, 25614164, 25187524, 25662413, 25739829, 25923550, 25979364, 25605856, 25629741, 26206451, 25798575, 26318836, 26327130, 25431052, 25937443, 26234554, 26181658, 25676422, 26310692, 26272769, 26272768, 26071796, 25817373, 26257021, 25540891, 26318715, 25504994, 25584002, 25842286, 26296954, 25963890, 26598141, 26645895, 26044855, 26386540, 25726088, 25662592, 25114060, 26088268, 25467848, 26432181, 26432182, 26109202, 26460661, 24550171, 25552421, 25301853, 25788018, 25579833, 26095467, 25547503, 26422723, 26135703, 26699169, 26033508, 24968970, 25795413, 26699168, 24107979, 25301758, 25524951, 26248570, 26414670, 25129681, 25403221, 25465978, 25482330, 25842221, 26160875, 25686165, 26151264, 25935793, 25414157, 26388532, 26188742, 25790882, 25952354, 26449137, 25415807, 26571066, 26575258, 26569658, 25450208, 25937436, 26551263, 26551051, 26049551, 26414968, 26115797, 25691433, 25467562, 26010803, 25601342, 25559324, 25437881, 26432671, 25135867, 26116134, 25908089, 25882376, 26597263, 25981812, 26181254, 26505753, 26129947, 25403587, 25656999, 26408273, 25766947, 26360446, 25844995, 25853744, 25756440, 25800582, 25301760, 25893283, 25952317, 25422488, 25847934, 25174865, 25624439, 26351969, 25918277, 26014293, 25497694, 26414022, 26004375, 26466871, 25722382, 25152207, 25409287, 25205534, 26244854, 25890673, 26244306, 25651245, 25644760, 25112662, 26040806, 25557952, 26653622, 25524798, 25465111, 25605812, 25617215, 25627357, 26268910, 26248510, 25720600, 25851633, 25079365, 26228487, 25468944, 26009595, 25887355, 26407587, 25524401, 25739769, 25398374, 25499773, 25733157, 26424865, 25612858, 26022965, 26429075, 25219423, 25913022, 26222557, 25882510, 25413378, 26573069, 25794890, 26284719, 25573406, 24385202, 26051365, 25431271, 25795406, 25534356, 25304132, 25787196, 25624435, 25647203, 25361980, 25355722, 26272871, 25580725, 25615566, 26237520, 24849310, 25627448, 25981810, 25512454, 25219389, 26106946, 26550795, 25776532, 25774645, 25992746, 25977146, 25724455, 26181252, 25534375, 25769495, 26486191, 25450717, 25450709, 26039792, 25943350, 25614172, 25975659, 25590331, 25617503, 25701273, 26322819, 25588294, 25230595, 25738669, 25560711, 26209642, 26416897, 24763133, 25607727, 26431720, 25445400, 25881936, 26215765, 26106097, 26075752, 25617224, 25359163, 25476069, 25681464, 25800891, 25805811, 26234174, 26535958, 25488686, 25964257, 25500422, 25518957, 26085041, 25403582, 25601789, 25720624, 27626136, 27470449, 27515333, 27050187, 27179402, 26494415, 26813674, 27557300, 26720751, 26720730, 26562047, 27056608, 26626991, 25986943, 26786575, 25765462, 26903081, 26283011, 26980000, 26644172, 27012403, 26930055, 27418381, 26290587, 26782346, 26358288, 27287210, 26861608, 26038091, 27418551, 26916095, 27532829, 26892411, 26685010, 27884241, 26324844, 26786576, 26245756, 26707868, 26364551, 26585431, 25792708, 26782957, 27130705, 27222555, 26602782, 25862143, 26405233, 26681680, 27044933, 27056996, 26483047, 26844840, 26981934, 27412888, 26651376, 27223147, 27557301, 26578734, 27783918, 26760085, 27456299, 26940805, 27074066, 27732819, 26802156, 26802147, 26581915, 27001568, 26858335, 26614823, 27695894, 26272237, 26481928, 26504153, 27029350, 27679583, 26954779, 27532828, 27479847, 27022113, 27618593, 26784863, 26826179, 27806235, 27012405, 27542302, 27602665, 27332902, 27406346, 27406347, 27524393, 26719232, 27313086, 27208343, 26774179, 27576780, 27682034, 26863355, 27613521, 27660192, 27265347, 26527782, 26598744, 27718781, 26969090, 27022111, 27287206, 27325147, 26720308, 26733609, 26646759, 26822398, 27083334, 27084954, 27487479, 26526713, 26858277, 27043774, 26628419, 26984187, 27043165, 26541814, 27575599, 26529126, 27371719, 25792707, 27474214, 27076599, 26906526, 26686313, 26686957, 26598748, 26610858, 27348762, 27248440, 26842679, 26727041, 27418629, 26735993, 27424812, 27665230, 27271183, 27730283, 26792812, 26940689, 27737744, 26603174, 26637834, 26404953, 27486164, 26789727, 26833336, 27872156, 26751506, 27348249, 27760796, 26330424, 26681677, 27322089, 27006510, 27669539, 26746140, 26321238, 26469139, 27595918, 27627782, 27001565, 27271190, 26341275, 26970723, 27440003, 27343257, 27443435, 26522083, 27653939, 26704020, 27028914, 27298404, 25586058, 26315980, 27518321, 26816011, 26655425, 26470053, 27806243, 27208318, 27527848, 26853821, 27609406, 26754759, 26719230, 26976423, 27501767, 26628328, 27515331, 27173037, 26916481, 26892939, 27978944, 26834240, 27066739, 26620248, 26414380, 27039945, 27041480, 27208378, 26628415, 26802160, 27279544, 27155903, 26746631, 26803443, 27382098, 26863353, 27745820, 26700126, 26681720, 26362090, 27289121, 26590384, 27959716, 26476478, 26671818, 27439911, 26764065, 26586780, 26786921, 27234640, 27025186, 27107460, 27530656, 27294447, 27208464, 26644536, 27002107, 26924770, 25550338, 27040132, 27914656, 26269397, 26489704, 27688021, 27634117, 27270348, 26794729, 26520230, 26621483, 26569098, 26988951, 27294570, 27727383, 27532830, 26895675, 27480103, 26453773, 27312053, 25589511, 27622997, 26857999, 27487573, 27183035, 26687300, 27130929, 27002108, 26733110, 27311311, 27806897, 27430374, 27496855, 27417007, 26613768, 27569442, 27532508, 27243899, 27022068, 26374852, 27726951, 27523980, 27161539, 27734108, 26882111, 26481174, 27769562, 26681684, 26334785, 27482008, 27382104, 26668177, 27641781, 27181196, 27091717, 27339115, 26979085, 26764061, 27012401, 27040723, 27352955, 26376350, 27207107, 26503204, 27461440, 26351332, 26764063, 26731191, 26517576, 27571048, 26803397, 27012781, 27042964, 26821623, 26453968, 26822022, 26768165, 27561923, 27046159, 26778538, 27557302, 27705267, 27540041, 26850709, 27208331, 26740634, 27049915, 27281795, 27053444, 27571011, 26993881, 26967681, 27022822, 26422126, 27114587, 27604504, 26888258, 27542303, 26994121, 26989182, 27230046, 26919870, 26481172, 26962728, 26810768, 25187158, 26764260, 26926681, 26609008, 26119561, 26764069, 27190009, 27502729, 27269942, 27572953, 27012407, 27130691, 27311493, 26969758, 27455166, 27380464, 26895652, 27102506, 27751553, 27364049, 26487588, 27050189, 27585757, 27287832, 26975590, 27707496, 27590218, 27609678, 27875542, 27532914, 27283479, 27837602, 27367877, 28027368, 27115264, 27161611, 26864411, 27139058, 27533157, 26836730, 27273731, 27438995, 27765184, 26489809, 27825009, 26995298, 27367889, 27179988, 27120082, 26942922, 27302238, 26864410, 27599328, 26631833, 27267068, 27434441, 26746456, 27136347, 27570866, 27139057, 27785516, 27706466, 26910598, 26242443, 27002446, 27787564, 27879359, 26585988, 27380342, 26975647, 27131100, 27135598, 27601237, 26916176, 27771739, 27187300, 27852621, 27483065, 27179849, 27367876, 27846344, 27547925, 27695824, 26978207, 27893130, 26934259, 26762524, 27432930, 26626993, 27043082, 26967465, 27163986, 26809849, 27002445, 26954408, 27405322, 27245914, 27179847, 26975890, 27482002, 27380343, 27298411, 27404184, 27273839, 27116282, 26914592, 26975498, 27706464, 26813209, 27997653, 27043237, 27458945, 27585384, 26859559, 26859481, 27775503, 27742728, 27428731, 27081016, 27669457, 26842425, 26836729, 27002448, 27825007, 27367818, 26537183, 27271455, 26547100, 27599329, 27163985, 26954409, 26813208, 27654602, 26970721, 26908915, 26292778, 27474376, 27398874, 26857829, 26474809, 27717766, 27294708, 27132075, 26653067, 26827250, 27593504, 27797931, 26857383, 27875528, 26903176, 26704571, 27450474, 27032628, 26680217, 27076578, 27265548, 26220533, 26654125, 27539168, 27483064, 27377542, 27379904, 26162769, 26813210, 27695851, 26555329, 27006514, 27599265, 27653735, 26886521, 26868683, 27058906, 27498098, 27574788, 27816567, 27304433, 27609408, 26797774, 26762525, 27573936, 26454361, 27542322, 26578735, 26774508, 26952945, 27616196, 27493132, 27506222, 26656289, 26681713, 27650977, 27284114, 26965560, 27436275, 27185571, 27365261, 26321288, 26852726, 27030891, 26388169, 26511959, 26947523, 27344296, 26338095, 26293163, 26056119, 27289490, 27863809, 26672064, 26443023, 26275429, 26371143, 27472949, 27098404, 27039291, 27373329, 27115376, 27144848, 26417061, 27367832, 26580307, 27548766, 27521719, 27570178, 26518245, 26821625, 27325850, 27418378, 25995322, 27240903, 27626517, 27132212, 27371977, 27084959, 27373900, 27114593, 26874076, 27537841, 26912641, 26789872, 27299675, 27706483, 27025337, 27580428, 27743617, 27404094, 27599874, 27045127, 26896232, 26811535, 26210894, 26874885, 26540028, 27825638, 26600191, 26621682, 26552421, 27208377, 27777234, 26681715, 27621394, 26703889, 26794930, 26626617, 26405232, 27797291, 27247219, 27639082, 27651331, 27440192, 27459375, 26231884, 27477896, 26930627, 26947602, 26603917, 27232649, 27028913, 27264120, 26861923, 27177859, 26747792, 26606329, 26556618, 27503237, 27217448, 26858331, 27502725, 27216274, 26353789, 28029926, 27771985, 27364043, 26984864, 27203508, 27575024, 26861604, 26962727, 26881417, 27302126, 27509101, 26757463, 25873634, 27908454, 26947331, 27609676, 26822397, 26809813, 27567279, 26811524, 26562046, 26875743, 26223239, 26908914, 26884472, 26394161, 27208320, 26975007, 26673391, 27236344, 27863807, 27456837, 26953637, 27686943, 27029706, 27601539, 26780289, 27064456, 26771737, 27456384, 26480931, 26906014, 27502721, 26891677, 27436880, 28007133, 26325537, 26752109, 27776843, 27509097, 27612500, 28007137, 27212429, 27161966, 27049826, 26670617, 27324967, 26703894, 26740639, 27339116, 26968359, 28002714, 27184532, 27132052, 27132053, 26655421, 26673811, 27720136, 26564381, 27371185, 27291994, 26652797, 26719058, 27893068, 27029707, 26585409, 26359449, 26916483, 27777294, 27576774, 27297994, 27358434, 27052688, 27780198, 27143684, 26946925, 26516172, 27502703, 27151347, 27115286, 26585938, 27569726, 26915630, 26859081, 27181606, 26945047, 27777180, 27686945, 26850983, 27562972, 26796541, 26946343, 26374849, 27417000, 26858332, 27458234, 27838224, 27493136, 27745818, 26786577, 27163694, 27181456, 27136493, 27292104, 27411698, 26622051, 27484801, 26760084, 27276234, 26861924, 26764182, 27195814, 26700123, 26520703, 26976420, 26913614, 27019334, 26784110, 27022118, 26757276, 26681725, 26351971, 27865535, 27686346, 27085624, 26794722, 27388752, 26684607, 26491109, 27059887, 27335319, 27074067, 26784672, 26628478, 27098150, 26928567, 25670810, 26907736, 27788847, 27402145, 26899778, 26500139, 26742777, 27166670, 27705084, 27898672, 26672686, 27295427, 26283736, 26608256, 27538259, 27789196, 26884473, 26884592, 26837842, 27006512, 27810043, 27612501, 26777773, 27211272, 25757867, 27064975, 26464170, 26940688, 27686345, 27089068, 27802493, 27322798, 26486789, 27090975, 26890472, 26614104, 26712220, 26830377, 26924470, 25425655, 27325862, 27161018, 27009070, 26550687, 26757466, 26552942, 26944421, 27261067, 26856620, 27567239, 27592224, 26833209, 26569624, 26802176, 26947328, 27556593, 26840133, 26632520, 27456835, 26674653, 27118453, 26769742, 26708675, 27480145, 26527775, 26747333, 26903580, 26491112, 26905278, 27459502, 25355728, 26462728, 26467666, 27569526, 27469583, 26869049, 27007062, 26615328, 27653564, 26836220, 26874901, 27078022, 26767384, 27686347, 26507180, 26701362, 27913408, 27838646, 27116918, 26944026, 26556616, 26822326, 27717299, 27686343, 27718784, 26786573, 27217453, 26825000, 27348075, 27373812, 26599983, 27634581, 27299859, 27166622, 27345636, 26861603, 27283862, 27978942, 27410922, 27291997, 27824606, 27701661, 27311491, 27245485, 27358435, 27502710, 26725544, 26869247, 27806900, 27573032, 27276373, 26827074, 27119237, 26903390, 26988359, 27585503, 26474811, 26935273, 26863265, 26539881, 26707054, 26811525, 27617661, 27091810, 27144850, 27959613, 26573073, 26631116, 27199056, 26786744, 27273602, 27626365, 26686960, 26912557, 27168434, 27068858, 26739688, 27718847, 26712084, 27052652, 26908949, 27810312, 27712774, 27569438, 26321236, 27532780, 27144849, 26637987, 27289122, 27007578, 27559041, 26463318, 27056606, 25830326, 26852075, 27299809, 27458288, 27400949, 26976426, 27177865, 27458307, 27400947, 27573560, 27138575, 26755711, 26822749, 26785394, 27155741, 27573562, 26493792, 26886418, 27465265, 27071387, 27269946, 27756783, 27581531, 26965534, 27178479, 27133819, 27654344, 26673560, 27083332, 27407100, 27214731, 26802153, 27160473, 27622507, 27222510, 27576777, 26033153, 27443429, 26965540, 27682885, 26483255, 27221664, 26963689, 27067478, 27067479, 26938706, 27222508, 26645190, 26364263, 26628417, 26627366, 27043416, 26712905, 26719947, 26582645, 26141262, 26599707, 26723083, 26998789, 26785406, 27959713, 26903532, 27576775, 26906747, 27029708, 26508309, 26988955, 26811519, 27743922, 27287207, 27908347, 26796394, 26787231, 27717298, 27824882, 26640973, 26641557, 27780197, 27345639, 26487585, 27601545, 27344436, 27012778, 26898856, 26999485, 27998215, 27773665, 26606200, 27753609, 27283863, 27029704, 26620368, 26747859, 27143676, 27871759, 27050206, 26729431, 26608067, 26551550, 27765757, 25533646, 26577417, 27573938, 26185283, 27069071, 27289127, 26644535, 26932802, 27325848, 26951309, 26915677, 26472752, 27091875, 27702799, 27217446, 27085251, 27733377, 27185843, 27507876, 27998236, 27044935, 27022112, 27177863, 27354480, 26487578, 27400948, 26700124, 27208335, 28199143, 26598750, 27082942, 27192672, 26426458, 27959712, 26378963, 26834067, 26943128, 26962902, 27028911, 26644526, 26886419, 27509100, 27358437, 26223345, 27217449, 27358385, 27298414, 26884585, 27082062, 27217459, 26474810, 27043047, 26972498, 27289387, 26869678, 26775631, 26719225, 27173030, 27046160, 26821621, 27777279, 26530103, 27325864, 27788856, 27727279, 27055601, 25660991, 26577415, 26796396, 25935574, 27585605, 26774608, 27528177, 27114605, 27238752, 27686349, 26803476, 26485620, 27852684, 27717303, 27418379, 26968357, 27298402, 26817957, 26562292, 27080498, 27626518, 26712227, 26795874, 27474375, 26915674, 26724840, 27103127, 27641143, 26832963, 26840134, 27133971, 26640036, 26981935, 26858122, 27247226, 27959766, 26879279, 27543447, 27302362, 26892409, 27567243, 26547499, 26283739, 26757788, 27751554, 27765756, 27243803, 27751846, 26947719, 26601738, 27380540, 27156934, 27496680, 26879276, 26725450, 27139805, 27092831, 27394191, 27161257, 27579634, 27702796, 27665514, 27296693, 27160475, 27665515, 27622558, 26859076, 27451345, 27633186, 26547101, 26677940, 27269740, 26338825, 26500339, 27579635, 26949137, 27626622, 27729323, 27355532, 27177493, 27058908, 26903039, 27208375, 27919865, 26976418, 27733375, 28002709, 27749954, 26531865, 27012397, 27009916, 26799692, 26152318, 27598678, 27099151, 26940686, 27673310, 26449391, 26903575, 26822324, 27151177, 27310176, 26541531, 27575534, 27712772, 26417059, 26809111, 27573564, 26884590, 27312051, 27288520, 26988949, 27473760, 26590673, 27367766, 27247220, 26868687, 26103979, 27287209, 27481956, 26364288, 26933848, 26628508, 27573561, 27174742, 25475116, 27458809, 26560249, 26865601, 27015909, 26538525, 26644527, 27117531, 25550339, 27283332, 27444687, 27681865, 27026689, 26617202, 27252245, 27213646, 27686946, 27025586, 25800784, 25596179, 27305192, 27573034, 26659927, 26896734, 26861922, 26727163, 26502000, 27117749, 26657945, 27654449, 26400827, 27520594, 26646758, 27351380, 25795907, 26926656, 27058907, 26398704, 26742001, 26084406, 26139005, 26633571, 27071072, 26896460, 27056738, 27006332, 27258431, 26285584, 27056776, 26483049, 27504014, 26837684, 26340411, 27046162, 27160892, 26716735, 26483177, 27155740, 26960245, 26952547, 26511996, 27059729, 26559482, 27789470, 26757748, 27397040, 26948484, 27053442, 27040324, 27311495, 26757749, 27339398, 27598679, 27217455, 27509102, 27602507, 26962903, 26729895, 26986446, 27197690, 26729437, 27518661, 27690741, 27161974, 27040451, 26550689, 26684923, 27530741, 26831676, 26628416, 27103382, 27398991, 27737957, 27325857, 27181367, 27504637, 27212247, 26884557, 26900902, 26546548, 27081014, 27426247, 27959607, 27638357, 26921136, 27058909, 27151345, 26091907, 26553744, 26965717, 26892413, 27028912, 26699917, 27149033, 27806902, 26673352, 26540136, 26755507, 26935876, 27002117, 26933849, 27704151, 26846517, 27172277, 26790922, 26223240, 27575820, 27324280, 28183505, 28122895, 29097166, 27806919, 29100702, 29103847, 29236639, 27939058, 28209343, 29136001, 28742797, 29171821, 28625832, 28475678, 27959709, 27959715, 28885054, 27736154, 27624791, 29059253, 28453702, 28626220, 27899357, 27654851, 26150601, 28189771, 26905864, 28657870, 27507905, 28314755, 27811070, 28478399, 28087506, 28213566, 26801885, 28637670, 26318384, 27466231, 27803006, 27872058, 28135843, 28659038, 27826098, 28049139, 28950297, 28419193, 28327896, 27416912, 27773929, 27742394, 27831503, 28100019, 29059273, 28327907, 28426104, 28950305, 28625806, 28108117, 28790131, 28614691, 28464041, 29191321, 27531072, 28578639, 28578607, 28437520, 28600210, 29091568, 28470676, 28854066, 27513822, 28445659, 28832876, 28300506, 28479233, 26674360, 27811072, 28973066, 28564564, 28253390, 28976791, 29129443, 28661759, 28891408, 28031175, 28891423, 28581356, 28732650, 28278333, 27787547, 28604926, 27283216, 28514618, 28426106, 28877914, 28952891, 28624576, 28049170, 28501140, 28586279, 28645737, 28370357, 28830881, 27918762, 28476871, 28453701, 28950289, 28012564, 27602993, 28528561, 28595700, 28645853, 28398846, 28904070, 27307305, 27998967, 28845751, 28249150, 28527704, 27489241, 28213565, 28453721, 28584075, 28916443, 27584578, 28662808, 28683960, 28657417, 28057693, 28804124, 28851729, 28011037, 27942720, 28384828, 28477440, 28719683, 28292744, 28319243, 28006055, 28404658, 27812689, 27737436, 27560549, 28241237, 28619195, 28566338, 27979383, 28126898, 27117700, 28687275, 27749094, 27880977, 28199805, 27689735, 28199814, 28342911, 28327893, 28373701, 27611807, 28231946, 28209296, 28335000, 28438473, 28121498, 28459958, 28284557, 28654954, 29135993, 27190095, 29117336, 28797349, 28936678, 28402237, 28844201, 28249141, 28278391, 28379800, 28961845, 28111466, 28017406, 28423145, 28475456, 28418267, 27889574, 28131786, 28576675, 28468518, 28259608, 28753384, 28426123, 28199818, 28814431, 28605608, 28081827, 28605291, 28301264, 28025189, 28538111, 27803120, 28304242, 28794054, 28335841, 28843768, 28025582, 27298379, 28855115, 28644774, 29261655, 28602779, 27165179, 28982497, 28450350, 28380313, 27522164, 28052977, 28153828, 28525305, 27988142, 28855239, 29133529, 28636854, 28289041, 28813214, 28846769, 27746233, 28096223, 28097310, 28213480, 28678849, 28693854, 28705312, 28586856, 28412065, 28113032, 27196588, 28510573, 28115474, 28751548, 28407726, 28591523, 28642008, 28817370, 28125763, 28828487, 28118454, 28636405, 29023215, 28750695, 27918712, 27988144, 27959701, 28592386, 29033099, 28283282, 29096915, 28265678, 28475671, 28958502, 28040671, 27534671, 28945833, 28682681, 29081267, 28550195, 29182634, 29052691, 27998961, 27742650, 28355511, 27771973, 28167214, 28923793, 29084318, 28463633, 28126245, 28651927, 28363637, 28844193, 27831502, 28958328, 28600309, 28885881, 28408018, 27522159, 28291393, 28605290, 28634219, 28196255, 28692728, 28821576, 28146243, 28459925, 28975305, 28197680, 28973115, 27612178, 28430829, 28045890, 27918780, 28196254, 29114833, 27902836, 28609532, 28806791, 28655015, 28613956, 28228428, 28459931, 28973227, 29164255, 28264080, 28810024, 28027338, 28632867, 29279933, 28697253, 28558104, 28898379, 28787506, 28492910, 27913306, 28329240, 28655025, 28535235, 28787505, 29260226, 28241357, 28873161, 28505217, 28118453, 28254171, 28672318, 28322414, 28146251, 29112701, 28655016, 28654972, 28657784, 28763550, 28097356, 28097355, 28241354, 29059263, 28632865, 28055065, 27918764, 27767328, 28829877, 28973620, 29141087, 27893032, 29209721, 28600189, 28719693, 28528348, 27893064, 28973220, 28973344, 28926288, 28291891, 28672316, 28672317, 28166324, 28512184, 28834483, 28322416, 28768334, 28510679, 28535237, 28334365, 28787507, 29234808, 28601891, 29136444, 28030702, 28476766, 28359749, 28975307, 28609534, 28973363, 28241153, 28468794, 28306757, 28880972, 28829876, 28063795, 28743190, 27893018, 29183074, 28418482, 29164254, 28510680, 28763548, 28829884, 29049653, 29084312, 28873160, 28975304, 28973516, 28672319, 28241271, 29067426, 28973619, 28330649, 27797925, 28522311, 27965257, 27978579, 29101402, 28531241, 28973065, 29100704, 28241231, 28687830, 27893884, 28520838, 29067428, 29096949, 28350928, 29260225, 28402745, 28437795, 29136445, 28350929, 28262598, 28746708, 28809935, 29112963, 28419095, 28241215, 28192551, 27720838, 28892496, 29049469, 27432356, 27650174, 27669524, 28189751, 28264082, 28672291, 28847206, 29049577, 28619659, 28424359, 28030741, 28114607, 28029308, 28973535, 28787504, 28869987, 27797930, 28013329, 28910237, 28846767, 28335624, 28459932, 29141784, 27914822, 28899222, 28242304, 28539196, 28539194, 27903568, 28468770, 28017403, 28844990, 28473423, 28478975, 27672124, 27658762, 27130908, 28605603, 28818994, 28428368, 29074099, 28390867, 28750582, 28344112, 28385659, 28760384, 28461207, 27856432, 29107679, 27117698, 28584187, 27864024, 28882235, 28238711, 27654854, 28864027, 28390869, 28328346, 28614554, 28628363, 28471726, 28012485, 29233854, 28444114, 28240966, 28700805, 28395936, 27918778, 28636855, 28838366, 27939076, 28570730, 28854065, 28500214, 28700791, 28705454, 27889312, 28256747, 28167660, 28691557, 27913576, 29262276, 28389389, 28723333, 28089180, 28450313, 27477525, 28091362, 28137739, 28514624, 28056205, 28209728, 28132019, 28412295, 28304224, 28720592, 28359513, 27795558, 29031778, 28043872, 28531910, 27941065, 28068170, 28219690, 28356319, 28483786, 28389435, 28701367, 27838347, 28586116, 28113021, 27993829, 27993828, 27993816, 28355112, 28628702, 28096109, 28373523, 28967485, 28781171, 27246522, 28082379, 27302659, 28813218, 27814453, 26976734, 28700844, 28317428, 29171818, 28423305, 28375647, 27507903, 28126349, 28335622, 28103714, 28968167, 28882536, 28538136, 28768627, 28096535, 29088928, 28715532, 28128852, 28600913, 28077460, 28688129, 28844816, 28648402, 28838862, 28760403, 28162999, 29129441, 28453703, 28574779, 28385948, 29126212, 28124736, 28100438, 28692301, 28082387, 28499583, 29161260, 28187920, 28608719, 28007348, 28346581, 28641792, 28219612, 28368672, 28139405, 28295463, 28291882, 28454674, 28444267, 28542506, 28029312, 27965285, 28375706, 29088237, 27687308, 27497762, 29138273, 28809926, 29045529, 28034064, 28128967, 28459989, 28727491, 27491641, 28303301, 28335835, 27400939, 28715535, 28526493, 28329235, 28838362, 28335012, 28043903, 28444112, 28306389, 28427286, 29133014, 28830875, 27321436, 29169472, 28225674, 28039187, 28317438, 28327932, 28628707, 28947569, 28384682, 28393258, 29059277, 28431003, 28052915, 28356246, 27773851, 28319997, 27604532, 28576285, 28146651, 27269294, 27856497, 28094141, 28834453, 27993800, 28800861, 28359784, 28549783, 27765690, 28135143, 28859790, 29054911, 28551073, 27553214, 28045625, 28320223, 28514613, 28012663, 28679741, 28029317, 28579538, 28094198, 29203564, 28715907, 28426350, 28379796, 27821644, 29073947, 29211658, 28320259, 28316276, 28304227, 28854085, 28188240, 28376151, 29141164, 28068780, 27916480, 29072977, 28418763, 28467729, 28478972, 28606961, 28273028, 28902590, 28911510, 29175902, 27797928, 28877913, 28430910, 27881565, 28973211, 28424223, 28973174, 28522450, 28654363, 28654682, 27582509, 28094194, 28611080, 27825963, 28241185, 28389316, 28376149, 28119329, 28069279, 28414061, 27506951, 28193797, 27745812, 28898378, 28893134, 28514601, 28010989, 27685251, 28500216, 28384657, 28644114, 27870567, 27923814, 28355113, 28238742, 28097324, 28580882, 28968163, 28120005, 28095414, 27687307, 28240967, 27893331, 28494974, 28380315, 28595694, 28228427, 28231950, 28784312, 28347733, 28707987, 29146401, 28403383, 28671857, 28827410, 28892431, 27881556, 28734759, 27932067, 29023213, 28651929, 27864127, 27523435, 28034891, 28529147, 29051184, 29091570, 28976863, 28796588, 28131794, 28002679, 28002688, 28029326, 28520519, 28578601, 29103871, 28754483, 27609658, 28389393, 28389391, 28027332, 29121226, 28190762, 28530840, 28880867, 26848182, 28958678, 27959700, 28398499, 29236628, 29045520, 28889792, 28081824, 28844200, 28007457, 28705314, 27998910, 28734830, 27630212, 28661758, 28808144, 28902593, 28902580, 27609242, 28114648, 28798049, 27799159, 28012849, 28327934, 29083953, 27911566, 28010994, 28168303, 28212060, 28988646, 29191325, 27810347, 28163000, 28241247, 28672306, 28027333, 28533290, 27927683, 29211671, 29020589, 27998964, 28076709, 28368437, 28445097, 28384065, 28135150, 28489511, 28809610, 28034079, 28902534, 28958324, 28637881, 28114683, 27997805, 28471697, 28142273, 27827824, 28280233, 28223407, 28637287, 28687377, 28687375, 28398845, 28774885, 28213610, 28596023, 28404657, 27864015, 28100606, 28341782, 28715540, 28031173, 27821724, 28282501, 28110412, 28271319, 28328347, 28351897, 28972004, 28483500, 28687477, 27607734, 28882233, 28847895, 28389572, 28236583, 28135140, 28343976, 28686535, 28632487, 27544575, 28376214, 27815101, 27893880, 28624580, 28100551, 28982501, 28657793, 28427580, 28329389, 28330794, 28146658, 28404647, 27543430, 28546260, 28407727, 28711629, 27856208, 28424222, 28794001, 28081369, 27554812, 29045553, 28418767, 28825853, 28774880, 28402747, 28486043, 28221862, 28862883, 27993804, 28383633, 28437161, 27523961, 27893326, 28423407, 28296582, 27631964, 27748623, 27898215, 28034081, 27918718, 29149418, 28632486, 28473136, 28533209, 27914900, 28602200, 28449038, 28869989, 28936675, 27881555, 28687232, 28376188, 27932229, 27432355, 28780512, 28209654, 28759347, 29130845, 27937096, 28216660, 28336937, 28334999, 27775818, 28007576, 28958504, 27457511, 28423301, 28550194, 28408020, 27884679, 28953447, 28668386, 28316279, 28892526, 28844192, 28892457, 28392237, 27916398, 29074595, 27838190, 28216232, 28437539, 28642125, 29066163, 28522181, 28460892, 29084313, 28320635, 28460889, 28229892, 28139349, 28216244, 28129985, 28174194, 28159608, 28974553, 28318984, 28533093, 28483414, 28017399, 28060545, 28365225, 28985239, 28606591, 27955995, 28828489, 28238601, 29136037, 28363636, 28742880, 27084245, 28626088, 27582421, 27169431, 29045535, 28468840, 29114781, 28492898, 28329315, 28246236, 28245280, 28384752, 28324081, 27611637, 28296618, 27770561, 28158487, 28302292, 27912982, 27965111, 28399154, 27641510, 28855173, 28564569, 28220512, 27687820, 26503631, 26657900, 28531281, 28729151, 28327975, 28184431, 28729250, 29050384, 28637880, 28154006, 27836885, 28396144, 28274941, 29069568, 28304219, 29241487, 28097317, 28898239, 28898283, 28135846, 28502609, 29113968, 28158398, 28489508, 28742911, 27893087, 28691885, 28975241, 27918724, 28780236, 27545497, 28362562, 28467880, 28241356, 28241355, 29099344, 29099333, 28877011, 29050397, 28606970, 27899497, 28742913, 28487294, 28399129, 28835465, 28584189, 28733374, 27988143, 28389582, 28350796, 28006059, 28241154, 27196574, 28687711, 28325784, 27744739, 27997679, 28802565, 28768327, 28118559, 28081952, 27959702, 28402245, 27840336, 27959717, 28118549, 28596043, 28241159, 28893843, 28385346, 28877027, 28029329, 28389552, 28467869, 28209624, 29045207, 28130206, 29045212, 29105594, 28007428, 28363222, 27774838, 27663604, 28526536, 28343975, 28526538, 28056202, 28525302, 28249134, 27993811, 28236469, 27842383, 28729154, 27788026, 28538134, 28657871, 28564557, 28328324, 28745999, 27658562, 29059426, 29206996, 27964843, 28188086, 28012977, 27939035, 28379802, 29145948, 28317415, 28662222, 28605779, 28317458, 28209298, 28949973, 27832260, 28945839, 28302288, 27825616, 28259610, 28028552, 28369340, 28118659, 29136032, 28422514, 27978548, 28285844, 28027334, 28650204, 28522338, 28631003, 29211857, 27974550, 29023915, 28833331, 28969438, 29224502, 29241357, 28329231, 29231133, 29231094, 29142011, 29222139, 27802155, 29133606, 29242349, 29246950, 29174928, 29084737, 28535964, 28479329, 29021322, 29030102, 29277560, 28053054, 28945120, 28970212, 29242350, 29151359, 29130810, 28056206, 28946761, 29136387, 29191844, 29232153, 28689794, 29215955, 29104121, 29232171, 29281101, 28507219, 29122523, 29198583, 29187423, 29129157, 29208679, 29142012, 29262271, 28552588, 7030908, 1906029, 8440446, 4162666, 23737460, 367494, 10381939, 1551558, 7615217, 11357858, 11777372, 11926807, 1688499, 23761185, 1397897, 19318629, 11785469, 11759664, 1698172, 1499487, 2019385, 2676686, 9340299, 1985058, 18485319, 25888112, 16344081, 9824337, 2788118, 23761192, 9024321, 8440447, 4916451, 5694972, 11313329, 23737438, 9288003, 3500103, 10702217, 9098033, 9797392, 1679735, 11396433, 11324633, 11926809, 11926808, 2197211, 16237869, 1451990, 16344085, 11054401, 4195118, 2205560, 1985059, 2583680, 4135556, 2013394, 12806641, 3286465, 25911449, 2114337, 6253688, 8462814, 8440449, 8419257, 4883650, 7615216, 11759662, 11900514, 2044914, 9207302 |

Note: PMID order by year of publication.
