# Supplementary material for: Global mapping of randomised trials related articles published in high-impact-factor medical journals: a cross-sectional analysis
Source: Trials. 2020 Jan 7;21:34. doi: 10.1186/s13063-019-3944-9 (PMC6947860; doi:10.1186/s13063-019-3944-9)
Supplement: Supplementary file 5 — Additional file 5. List of excluded articles. [file 13063_2019_3944_MOESM5_ESM.docx]

**Additional file 5. List of excluded articles.**

| **No.** | **PMID** | **Title** | **Year** | **Journal** | **Cause** |
| --- | --- | --- | --- | --- | --- |
| 1 | 14057 | Sulphasalazine in asymptomatic Crohn´s disease. A multicentre trial. | 1977 | Gut | No record in WoS |
| 2 | 29035093 | Gastric Banding Surgery versus Continuous Positive Airway Pressure for Obstructive Sleep Apnea: A Randomized Controlled Trial | 2018 | Am J Resp Crit Care | No record in WoS |
| 3 | 26115379 | Correction: Efficacy of Pneumococcal Nontypable Haemophilus influenzae Protein D Conjugate Vaccine (PHiD-CV) in Young Latin American Children: A Double-Blind Randomized Controlled Trial. | 2015 | PLoS Med | Corrections and/or errata |
| 4 | 26106226 | Erratum. Efficacy and Safety of Dulaglutide Added Onto Pioglitazone and Metformin Versus Exenatide in Type 2 Diabetes in a Randomized Controlled Trial (AWARD-1). | 2015 | Diabetes Care | Corrections and/or errata |
| 5 | 26404929 | Erratum. Vitamin D supplementation and the effects on glucose metabolism during pregnancy: a randomized controlled trial. | 2015 | Diabetes Care | Corrections and/or errata |
| 6 | 27258028 | Correction: Initiating Antiretroviral Therapy for HIV at a Patient´s First Clinic Visit: The RapIT Randomized Controlled Trial. | 2016 | PLoS Med | Corrections and/or errata |
| 7 | 27760145 | Correction: Supported Telemonitoring and Glycemic Control in People with Type 2 Diabetes: The Telescot Diabetes Pragmatic Multicenter Randomized Controlled Trial. | 2016 | PLoS Med | Corrections and/or errata |
| 8 | 28420696 | Erratum. Benefits of LixiLan, a Titratable Fixed-Ratio Combination of Insulin Glargine Plus Lixisenatide, Versus Insulin Glargine and Lixisenatide Monocomponents in Type 2 Diabetes Inadequately Controlled on Oral Agents: The LixiLan-O Randomized Trial. | 2017 | Diabetes Care | Corrections and/or errata |
| 9 | 28420697 | Erratum. Sitagliptin Attenuates the Progression of Carotid Intima-Media Thickening in Insulin-Treated Patients With Type 2 Diabetes: The Sitagliptin Preventive Study of Intima-Media Thickness Evaluation (SPIKE). A Randomized Controlled Trial. | 2017 | Diabetes Care | Corrections and/or errata |
| 10 | 28615238 | Erratum. Gestational Diabetes Mellitus Can Be Prevented by Lifestyle Intervention: The Finnish Gestational Diabetes Prevention Study (RADIEL). A Randomized Controlled Trial. | 2017 | Diabetes Care | Corrections and/or errata |
| 11 | 28887408 | Erratum. Application of Zone Model Predictive Control Artificial Pancreas During Extended Use of Infusion Set and Sensor: A Randomized Crossover-Controlled Home-Use Trial. | 2017 | Diabetes Care | Corrections and/or errata |
| 12 | 28452409 | Ketamine versus midazolam in bipolar depression with suicidal thoughts: A pilot midazolam-controlled randomized clinical trial. | 2017 | Bipolar disorders | Indexing error (ineligible journal) |
| 13 | 7392709 | Sulfinpyrazone in the prevention of sudden cardiac death after myocardial infarct. | 1980 | Med Clin (Barc) | Indexing error (ineligible journal) |
| 14 | 21253791 | Chemoprevention of hepatocellular carcinoma in chronic hepatitis C. | 2011 | Recent Results Cancer Res | Indexing error (ineligible journal) |
| 15 | 20585979 | Endoscopic therapy for peptic ulcer hemorrhage: practice variations in a multi-center U.S. consortium | 2010 | Dig Dis Sci | Indexing error (ineligible journal) |
| 16 | 19846309 | The PedsQL in pediatric patients with Spinal Muscular Atrophy: feasibility, reliability, and validity of the Pediatric Quality of Life Inventory Generic Core Scales and Neuromuscular Module. | 2009 | Neuromuscul Disord | Indexing error (ineligible journal) |
| 17 | 10228190 | Infliximab for the treatment of fistulas in patients with Crohn´s disease | 1999 | N Engl J Med | Duplicate |
| 18 | 1568716 | Interferon-Alpha in Acute Posttransfusion Hepatitis-C – A Randomized, Controlled Trial | 1992 | Hepatology | Duplicate |
| 19 | 17335962 | Extracorporeal magnetic stimulation is of limited clinical benefit to women with idiopathic detrusor overactivity: A randomized sham controlled trial | 2007 | Eur Urol | Duplicate |
| 20 | 19766640 | Colectomy Rate Comparison After Treatment of Ulcerative Colitis With Placebo or Infliximab | 2009 | Gastroenterology | Duplicate |
| 21 | 19195772 | Dapoxetine for the Treatment of Premature Ejaculation: Results from a Randomized, Double-Blind, Placebo-Controlled Phase 3 Trial in 22 Countries | 2009 | Eur Urol | Duplicate |
| 22 | 23020132 | Improved Survival with MEK Inhibition in BRAF-Mutated Melanoma | 2012 | N Engl J Med | Duplicate |
| 23 | 1434838 | Long-Term Effects of Prolonged Maintenance and of Very Early Intensification Chemotherapy in AML – Data from AMLCG | 1992 | Leukaemia | Duplicate |
| 24 | 3500103 | Prevention of variceal rebleedling by propranolol: should it work? does it work? | 1987 | Hepatology | Duplicate |
