# Supplementary material for: Global mapping of randomised trials related articles published in high-impact-factor medical journals: a cross-sectional analysis
Source: Trials. 2020 Jan 7;21:34. doi: 10.1186/s13063-019-3944-9 (PMC6947860; doi:10.1186/s13063-019-3944-9)
Supplement: Supplementary file 6 — Additional file 6. Exploratory analysis of topical data. [file 13063_2019_3944_MOESM6_ESM.docx]

**Additional file 6. Exploratory analysis of topical data**

| **Figure. Word cloud for the frequency of terms.**    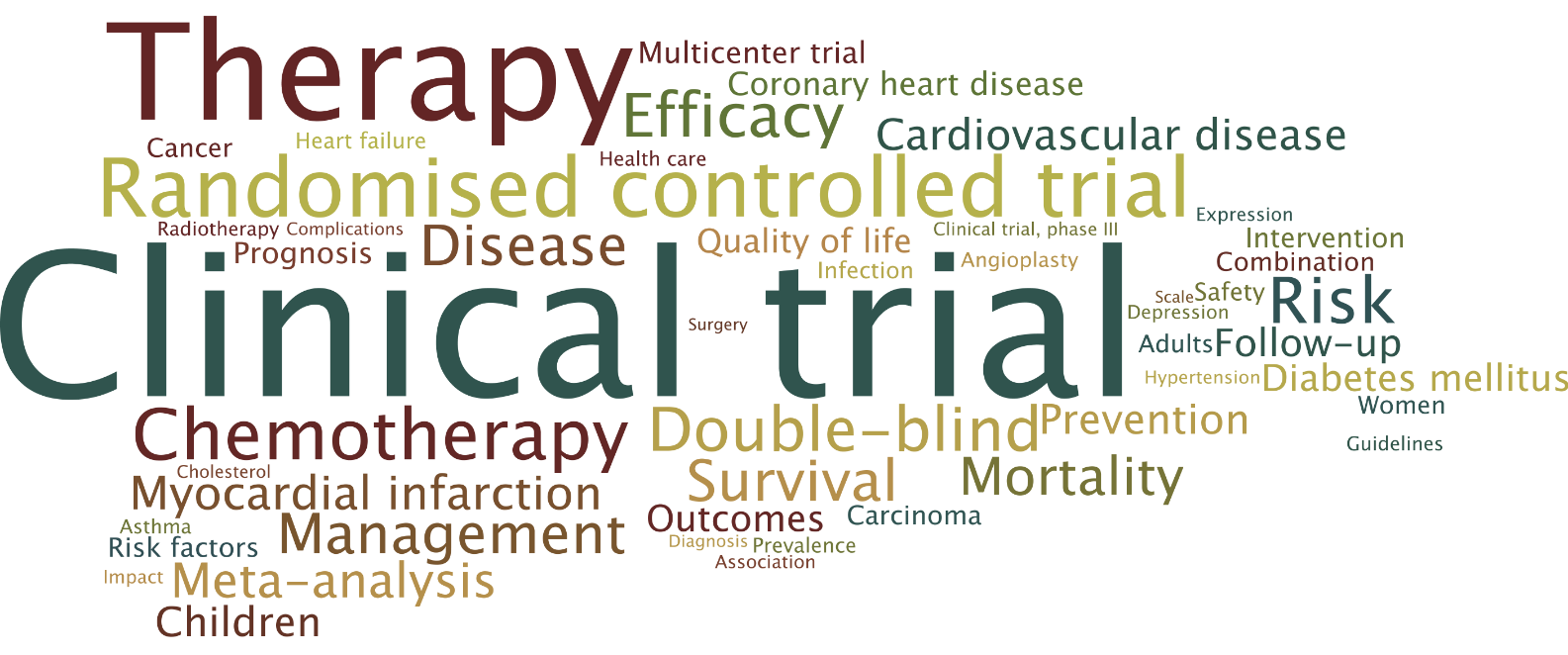  Note: Most frequently used keywords (at least 500 times).  Based on most frequently used keywords (with at least 500 articles), a word cloud was created using Wordle (http://www.wordle.net/), which is free-software that generates “word clouds” from text that the user provides and places more emphasis on words that appear with greater frequency in the source text. Commentary: The most commonly used article keywords were “clinical trial” (16.1%; n=6332 papers), followed by “therapy” (10.8%; n=4267), “randomised controlled trial” (6.6%; n=2587), “chemotherapy” (5.6%; n=2224), “risk” (5.1%; n=2026), “efficacy” (4.9%; n=1933), and “double-blind” (4.9%; n=1929).  **Figure. Co-words network of the keywords.**  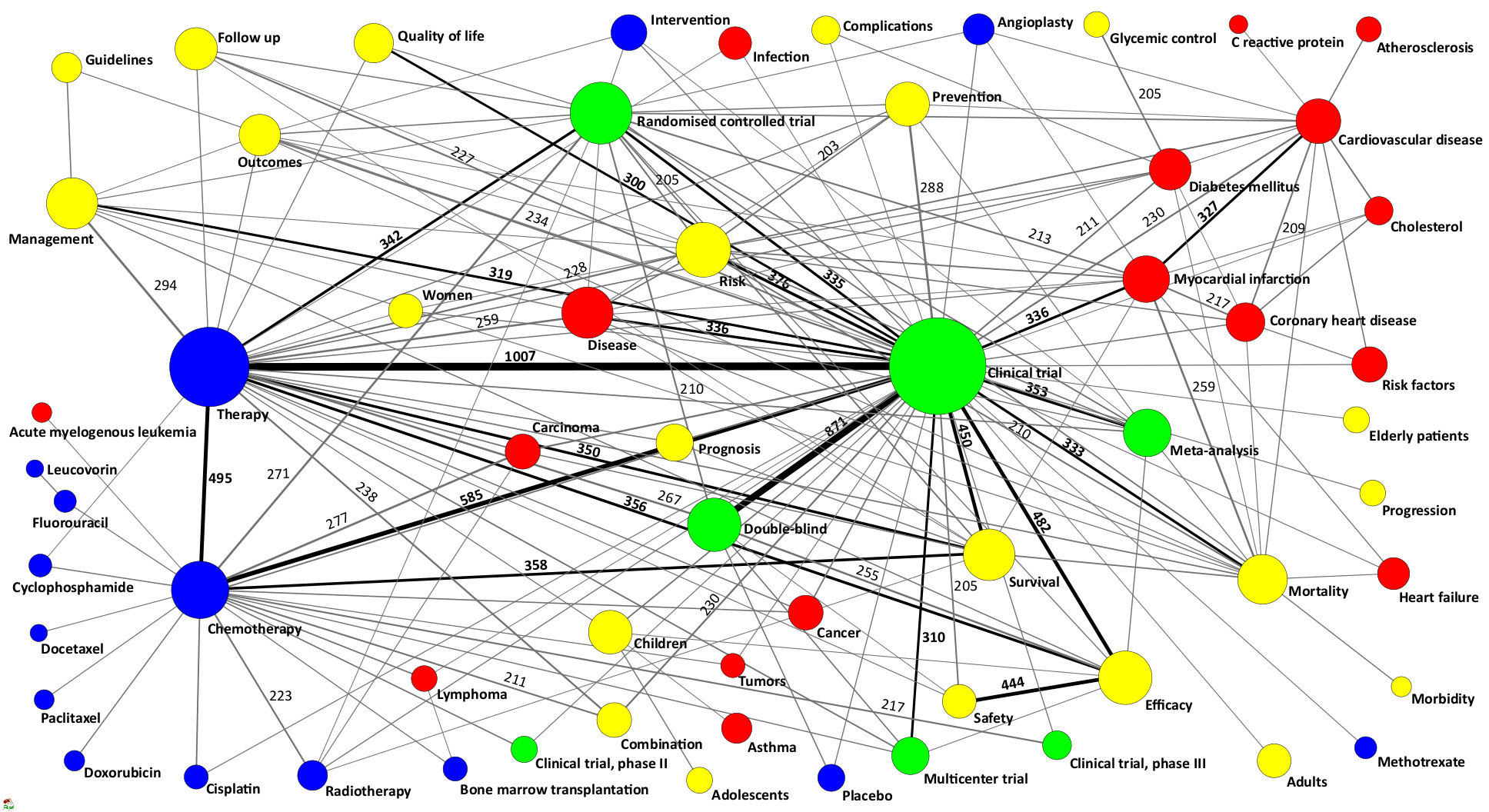  Note: Node sizes are proportional to the number of papers and line thicknesses are proportional to the number of co-occurrences of words. Numbers are presented for co-occurrences on ≥ 200 words. Node colors: blue = words related to interventions; green = words related to methodology/clinical trials; red = words related to diseases/disorders, signs, symptoms and biomarkers; yellow = words related to general terms.  A “co-words network” of keywords was created to illustrate the co-occurrence phenomenon of highly frequent words in the articles to map the associations between keywords in textual data (applying a threshold of 100 articles in collaboration). Commentary: “Clinical trial” occupied a central position in the entire co-word network. Co-words analysis showed some associations of keywords forming intense groupings of terms, such as “clinical trial” and “therapy” with either “randomised controlled trial”, “double-blind”, “chemotherapy”, “efficacy”, “risk”, “mortality” or “survival”. Within diseases, we noted a cluster of keywords related to “cardiovascular disease”, such as “myocardial infarction”, “coronary heart disease”, “cholesterol”, “atherosclerosis” and “C reactive protein”, but also with “diabetes mellitus”. Within therapies, we showed associations of keywords forming a star network or groupings of multiple antineoplastic therapies directly connected to “chemotherapy” (as a common central term). |
| --- |
